# Spatiotemporal Pattern of a Macrofungal Genus *Phylloporia* (*Basidiomycota*) Revealing Its Adaptive Evolution in China

**DOI:** 10.3390/jof10110780

**Published:** 2024-11-10

**Authors:** Xue-Wei Wang, Li-Wei Zhou

**Affiliations:** 1 State Key Laboratory of Mycology, Institute of Microbiology, Chinese Academy of Sciences, Beijing 100101, China; xuewei_wang1995@im.ac.cn; 2 University of Chinese Academy of Sciences, Beijing 100049, China

**Keywords:** adaptive evolution, environmental variable, *Hymenochaetaceae*, macrofungi, net diversification rate

## Abstract

The understanding of distribution and the evolutionary scenario is crucial for the utilization and conservation of biological resources; nevertheless, such explorations rarely focus on macrofungi. The current study selects a macrofungal genus, *Phylloporia,* and explores its spatiotemporal pattern in China. A total of 117 available occurrence records of *Phylloporia* in China were summarized for the current analyses. Ensemble modeling supports the highly suitable habitat of *Phylloporia* concentrated in southern, especially southeastern, China, where the ancestor of *Phylloporia* originated 77.74 million years ago and then dispersed to other parts of China. Benefitting from the available suitable habitats, *Phylloporia* rapidly diversified after its divergence in Southeast China. Then, the net diversification rate slowed down when the rapidly diversifying species filled available niches in Southeast China and the dispersed species in other parts of China inhabited the less suitable and unsuitable habitats. During adaptive evolution, precipitation, temperature and the host plant are the major environmental variables that shape the spatiotemporal pattern of *Phylloporia*. In conclusion, the current study reveals the adaptive evolutionary scenario of *Phylloporia* and provides the first exploration of the spatiotemporal pattern of macrofungi.

## 1. Introduction

Biodiversity is closely linked to human well-being in various ways [1], and thus innumerable organisms have the potential to be strategic biological resources [2]. Thus, recognizing organisms and exploring their distribution and evolutionary scenario are crucial for the utilization and conservation of these resources [3,4]. In addition, the hypotheses of related biological and ecological phenomena are central topics in diversification and evolutionary biology [5,6,7,8].

Today, species diversity and the distribution of animals and plants are well recognized all over the world, and the corresponding evolutionary and spatiotemporal patterns of some biological groups have been accordingly explored [9,10,11,12,13,14]. Generally, species diversification is considered to be associated with the adaptation of biological and ecological functional traits along with their niche change over time and space. However, the adaptive evolution of different organisms in certain regions may be shaped by differentiated niche specialization. For example, environmental temperatures dominated the diversification of lizards [15], while the evolution of angiosperm flora in China was related to the boundary between humid–semi-humid and arid–semi-arid areas indicated by the modern 500 mm isoline of annual precipitation [16]. Nevertheless, the current evolutionary theory and spatiotemporal pattern are derived mainly from animals and plants, and whether they are applicable to other life forms, like macrofungi, is largely unknown.

Macrofungi, well known as mushrooms, are a group of fungi producing sporocarps for sexual reproduction and visible to the naked eye, but they also exist for a long time as an asexual mycelial form normally invisible to the naked eyes [17,18]. This unique life history makes describing and recording macrofungi more difficult than plants or animals. Indeed, it is postulated that the number of macrofungi and other fungal groups awaiting being newly described is much higher than that of known species [19,20]. Therefore, the data of macrofungi may be not comprehensive to support spatiotemporal analyses at a large taxonomic and geographic scale. Alternatively, a well-studied fungal group in taxonomy may serve as a typical exemplar for preliminarily exploring fungal spatiotemporal evolution.

*Phylloporia* Murrill is a macrofungal genus, which belongs to *Hymenochaetales* Oberw., *Basidiomycota* R.T. Moore. Some species in this fungal genus are plant pathogens, and several species have been utilized as medicines in Chinese folklore [21]. Therefore, *Phylloporia* attracts wide attention from fungal chemists for extracting natural products with various medicinal properties [22,23]. Since the publication of Zhou and Dai [24] that first studied the taxonomy of this fungal genus in China from both morphological and phylogenetic perspectives, species diversity of *Phylloporia* has been extensively explored in China. Today, a total of 34 species in this fungal genus are distributed in China [25]. From the evolutionary perspective, the biological traits of *Phylloporia*, especially the parasitic trophic mode, represent the most crucial innovative dynamics for species diversification in the order *Hymenochaetales* [26]. Therefore, knowledge of the spatiotemporal patterns of *Phylloporia* may provide insights into the conservation of the evolutionary potential of *Hymenochaetales* in China.

On the basis of current comprehensive recognition of *Phylloporia* in China, this study aims to clarify the evolutionary history of *Phylloporia* in China by exploring (1) the current potential distribution, (2) the possible historical distribution, and (3) the spatiotemporal pattern of *Phylloporia* in China, and the relationship between spatiotemporal pattern and environmental variables.

## 2. Materials and Methods

### 2.1. Species Occurrence Records

The occurrence records of *Phylloporia* in China were summarized from published papers [21,24,25,27,28,29,30,31,32], which resulted in a total of 117 records (Table S1). The geo-coordinates of these fungal records either came from field labels or were determined according to the sampling locations via Google Maps.

### 2.2. Environmental Variables

A total of 19 current bioclimatic indicators and corresponding altitude data (Table 1) were downloaded from the WorldClim version 2.1 database (https://www.worldclim.org/data/worldclim21.html; accessed on 15 December 2023). These environmental variables from the climate data for 1970–2000 at a spatial resolution of 30″ (approximately 1 km^2^) [33] were used to predict the current geographic distribution of *Phylloporia*.

Due to the parasitism of *Phylloporia*, the host plant was also considered one of the most important covariates determining the growth of *Phylloporia* [21,24,27,28,29,30,31,32,34]. Therefore, the distributions of the host plants of *Phylloporia* (Table S1) were retrieved from the Global Biodiversity Information Facility (https://www.gbif.org/occurrence/download/; accessed on 15 December 2023) as one of the variables (Table 1). The distribution of each species or genus of the host plant was downloaded with an accuracy to the county level, and the maximum number of downloaded items was set to 1000. For fungal species, whose host information was not identified to a genus level but indicated as angiosperms, the host information was filtered out in subsequent analyses. Finally, the number of host plants on each coordinate was converted to raster data by ArcGIS v.10.8 at a spatial resolution of 30″ (approximately 1 km^2^) for predicting the current geographic distribution of *Phylloporia* (Figure S1).

### 2.3. Modeling Procedure

**Figure 1:**
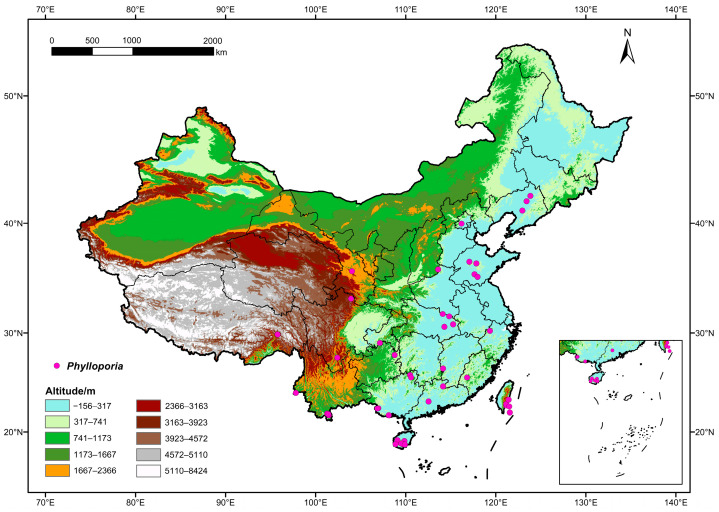
Known geographic distribution of *Phylloporia* indicated by the purple point in China. Map adapted from the National Geomatics Center of China (http://bzdt.ch.mnr.gov.cn/; accessed on 18 December 2023; review drawing number: GS(2019)1822).

To avoid data redundancy of spatial autocorrelation, if the sampling locations of occurrence records were distributed within 10 km, these records were treated as replications. After keeping only one record from replications, 44 of 117 occurrence records were filtered for predicting the geographic distribution of *Phylloporia* (Figure 1).

Previous studies indicated that a serious multicollinearity problem exists among various bioclimatic variables [35,36]. Thus, to avoid over-fitting induced by the multicollinearity of variables, Pearson’s correlation coefficient (r) analysis method was used to verify the correlation between primary environmental variables using ENMTools v.5.26 [37]. When |r| > 0.8, two environmental variables were considered to be autocorrelated and only one variable was randomly retained for further analyses (Figure S2). Eventually, Bio4, Bio6, Bio13, altitude and host plant were selected as the modeling factors (Table 1).

Ensemble modeling [38,39] was used to conduct the prediction with four different algorithms, viz., Generalized additive model [40], Generalized boosted model [41], Generalized linear model [42] and Random forests [43]. All model-building processes were conducted by the R package biomod2 v.4.2.5 [44].

Specifically, we first used random sampling methods to generate 500 pseudo-absence data points for the entire research area. This process was repeated three times to reduce the uncertainty caused by random sampling. Second, each of the four models was tuned by the function “bm_ModelingOptions”. Third, the four models were evaluated and built separately by the function “BIOMOD_Modeling”. A total of 75% of the 44 filtered occurrence records were randomly selected for model calibration and the remaining 25% for model testing with 10 replications to reduce the uncertainty. Thus, a total of 120 models generated from the four models, three-time replications of pseudo-absence data points and 10 cross-validation runs were built in this step. Furthermore, the accuracy of each model was evaluated using the values of the Area Under the receiver operator characteristic Curve (AUC) [45], and the True Skill Statistic (TSS) [39] values. The model is considered to perform well when the value of the AUC is higher than 0.8 and the value of TSS is not lower than 0.7. Fourth, ensemble modeling was generated by the function “BIOMOD_EnsembleModeling”. Committee Averaging (CA) and Weighted Mean (WM) methods were used to mix all 120 models to perform ensemble modeling. Each method was evaluated by both AUC and TSS values. Finally, ensemble modeling was projected by the function “BIOMOD_EnsembleForecasting”, which produced the raster map of distribution probability. Jenks’ natural breaks method was used to divide the potential habitat into four levels following the work of Zhao et al. [46].

### 2.4. Divergence Time and Historical Distributions

BEAST v.2.7.5 [47] was used to estimate the divergence time of each Chinese species in *Phylloporia*. The analyses included all Chinese species of *Phylloporia* and the representative Chinese genera belonging to *Hymenochaetaceae* Donk as ingroup taxa, while *Xylodon subflaviporus* Che C. Chen & Sheng H. Wu and *Lyomyces crustosus* (Pers.) P. Karst. from *Schizoporaceae* Jülich were chosen as outgroup taxa [48,49]. The ITS, nLSU, mtSSU, tef1α and rpb2 regions of these fungal species were downloaded from GenBank (https://www.ncbi.nlm.nih.gov/genbank/, Table S2). In addition, four newly generated sequences of *Phylloporia fontanesiae* L.W. Zhou & Y.C. Dai and *P*. *lonicerae* W.M. Qin, Xue W. Wang, T. Sawahata & L.W. Zhou were also supplemented (Table S2). Briefly, crude DNA was extracted from basidiomes of dry specimens using the FH Plant DNA Kit (Beijing Demeter Biotech Co., Ltd., Beijing, China), and then directly used as template for PCR amplifications. The primer pairs ITS1F/ITS4 [50] and LR0R/LR7 [51,52] were selected for amplifying ITS and nLSU regions, respectively, and PCR amplification followed the procedures of Wang and Zhou [49]. The PCR products were sequenced with the same primers in PCR amplifications at the Beijing Genomics Institute, Beijing, China, and then the sequences were submitted to GenBank.

Each region was separately aligned using MAFFT v.7.110 [53] under the “G-INS-i” option [54], and then concatenated as a single alignment (File S1). jModelTest v.2.1.10 [55,56] under Akaike’s information criterion corrected (AICc) was used to estimate the best-fit evolutionary model for the BEAST procedure. The lognormal relaxed molecular clock model and the Yule speciation prior were set to evaluate the divergence times and their corresponding credibility intervals. The offset age with a prior Gamma distribution (scale = 20, shape = 1) for *Hymenochaetaceae* was set as 125 million years ago (Mya) for calibration following the work of Wang et al. [57]. This time point was indicated by the minimum age of *Quatsinoporites cranhamii* S.Y. Sm., Currah & Stockey, a fossil poroid species collected from Apple Bay on Vancouver Island [58,59]. Trees were sampled every 1000th generation from a total of 100 million generations. The resulting log file was checked for chain convergence using Tracer v.1.7.1. After discarding the first 25% sampled trees, the remaining 75% samples were summarized to a single tree by TreeAnnotator [60].

To reconstruct the possible historical distributions of *Phylloporia* in China, we extracted the *Phylloporia* lineage as well as the *Fulvifomes* Murrill lineage as the outgroup from the time-calibrated phylogenetic tree generated by BEAST. The APE package implemented in RASP v.4.2 [61,62] was used for the reconstruction. The current geographic distributions of *Phylloporia* in China were divided into four areas, viz., Northeast, Northwest, Southeast, and Southwest, according to the Heihe–Tengchong line (proposed by Chinese geographer Huanyong Hu) that delimits the western and eastern parts of China, and the other Qinling–Huaihe extension line (proposed by Chinese geographer Xiangwen Zhang) that delimits the northern and southern parts of China. To avoid the bias caused by outgroup taxa, the distribution state of the outgroup *Fulvifomes* lineage was defined as “Null”. After pre-validation of the three models in APE package, viz., equal-rates (ER) model, all-rates-different (ARD) model and symmetrical (SYM) model, the ER model with the highest likelihood value was selected.

### 2.5. Spatiotemporal Pattern

To explore the spatiotemporal pattern of *Phylloporia* in China, we integrated the spatial distribution data with the time-calibrated phylogenetic tree generated by BEAST.

Maps of China used in this study were adapted from standard maps released by the National Geomatics Center of China (http://bzdt.ch.mnr.gov.cn/; accessed on 18 December 2023; review drawing number: GS(2019)1822). We divided the map of China into 100 km × 100 km grid cells. The divergence time of each species was represented by its stem age. The mean divergence time (MDT) of all species of *Phylloporia* occurring in a single grid cell was calculated by integrating spatial distribution data with the dated phylogenetic tree following the methods of Lu et al. [16]. The MDTs of *Phylloporia* in grid cells were classified into four levels by Jenks’ natural breaks method. Then, the MDTs of grid cells comprising species of *Phylloporia* were divided into four groups following their suitability as the potential habitat of *Phylloporia*. The differences between the groups represented by the MDTs of grid cells were compared using the Kruskal–Wallis non-parametric statistical test [63].

### 2.6. Net Diversification Rate

The Bayesian Analysis of Macroevolutionary Mixtures (BAMM) [64] method was used to estimate net diversification rate heterogeneity across lineages and through time, as well as to detect shifts in net diversification rates. The time-calibrated phylogenetic tree generated above by BEAST v.2.7.5 was used as the input tree file. The analysis was run for 100 million Markov chain Monte Carlo (MCMC) generations using four independent chains and sampling parameters every 10,000 generations. Ten thousand of the posterior samples were stored, with 2500 discarded as burn-in, and 7500 remained for subsequent analysis. The convergence of MCMC was checked with an effective sample size above 200 by the R package CODA v.0.19.4 [65]. The R package BAMMtools v.2.1.10 [64] was used to estimate speciation and extinction priors with the “setBAMMpriors” function, and to evaluate the outputs. The diversification analysis was run three times, each generating almost identical results, and thus only the results of the first run are shown.

## 3. Results

### 3.1. Modeling Accuracy Evaluation

For ensemble modeling, the average AUC and TSS values of the CA method were 0.885 ± 0.060 and 0.761 ± 0.122, respectively, while the average AUC and TSS values of the WM method were 0.935 ± 0.036 and 0.752 ± 0.127, respectively. Therefore, both methods supported the accuracy of the predicted modeling (Figure S3).

### 3.2. The Current Potential Distribution of Phylloporia in China

Bio4 (temperature seasonality), Bio6 (min temperature of the coldest month), Bio13 (precipitation of the wettest month), altitude and host plant are the crucial environmental variables for the current potential distribution of *Phylloporia* in China. Among these variables, Bio4, Bio13 and altitude have the highest importance scores of 0.45, 0.44 and 0.34, respectively, to ensemble modeling (Table 1).

**Figure 2:**
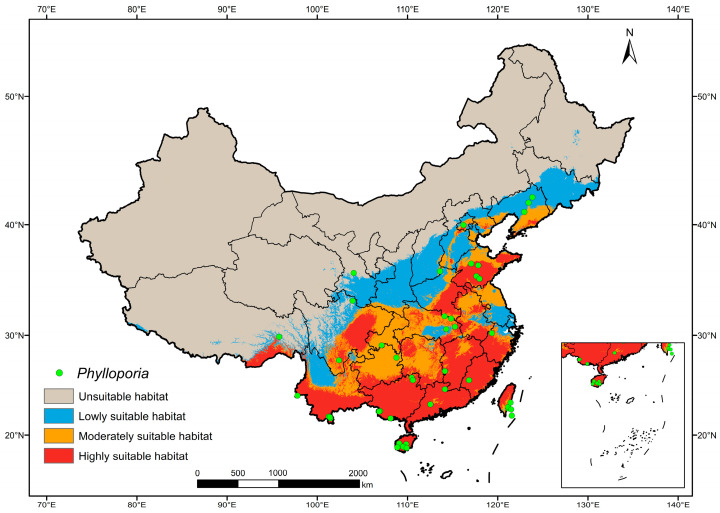
The current potential distribution of *Phylloporia* in China predicted by ensemble modeling. The green point represents the known distribution of *Phylloporia*, while the colored region in the map indicates the suitability of habitat for *Phylloporia* at four levels. Map adapted from National Geomatics Center of China (http://bzdt.ch.mnr.gov.cn/; accessed on 18 December 2023; review drawing number: GS(2019)1822).

The distribution suitability of *Phylloporia* was unequally classified into four groups using Jenks’ natural breaks method. The unsuitable habitat (0–0.18) occupies 608.421 × 10^4^ km^2^ (63.25%), while the lowly (0.18–0.45), moderately (0.45–0.68) and highly (0.68–1) suitable habitats occupy 110.613 × 10^4^ km^2^ (11.50%), 99.664 × 10^4^ km^2^ (10.36%) and 143.184 × 10^4^ km^2^ (14.89%), respectively. The main distribution of highly suitable habitats concentrates in the southeastern China, including Hainan, Taiwan, Guangdong, Guangxi, Fujian, Jiangxi, Zhejiang, southern Yunnan, southern Guizhou and southeastern Sichuan (Figure 2). In addition, highly suitable habitats are also scattered in Anhui, Hebei, eastern Henan, Hubei, Hunan, Liaoning, southeastern Shandong, and a small part of southeastern Xizang (Figure 2).

### 3.3. The Divergence Time and Possible Historical Distributions of Phylloporia in China

**Figure 3:**
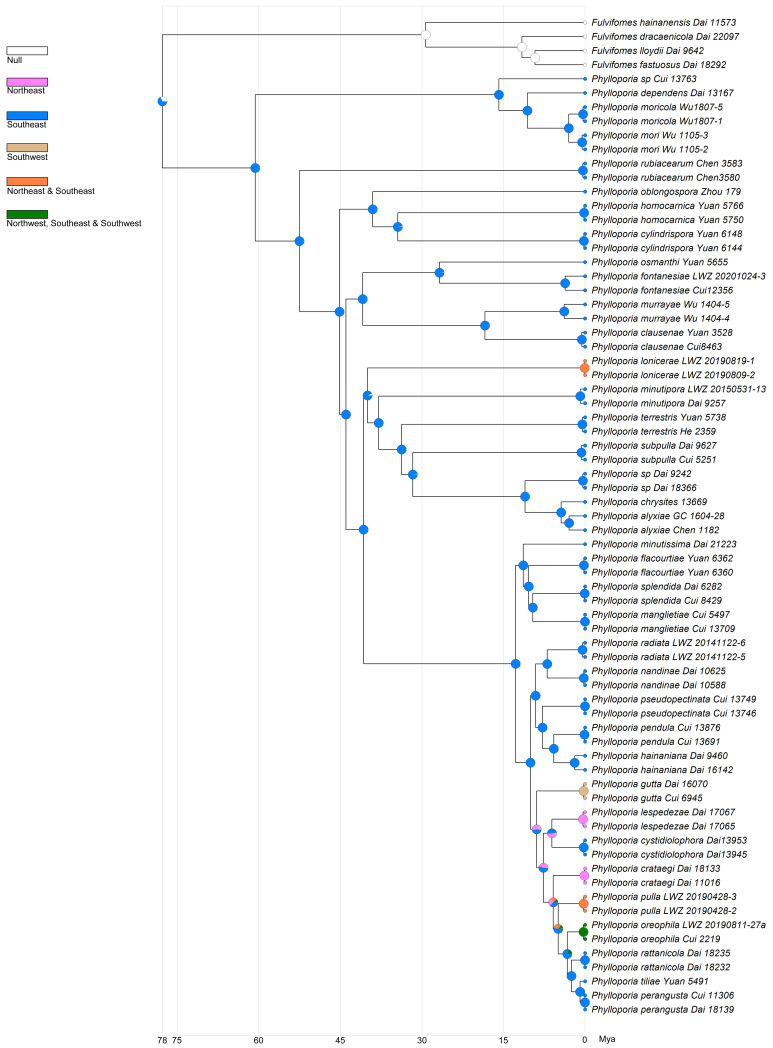
The possible historical distributions of *Phylloporia* in China. The *Fulvifomes* lineage, as the outgroup, was excluded from the reconstruction progress of historical distributions.

A total of 125 collections with 67 of *Phylloporia* in China were used to generate the time-calibrated phylogenetic tree (Table S2). The dataset generated a concatenated alignment of 4633 characters with GTR + I + G as the best-fit evolutionary model. According to the results generated by BEAST and RASP, the Chinese ancestor of *Phylloporia* was evaluated to originate and diversify in Southeast China since 77.74 Mya (stem age) with a 95% highest posterior density (HPD) of 69.22–86.82 Mya (Figure 3 and Figure S4). Until about 40 Mya, the genus dispersed to other geographic parts of China (Figure 3).

### 3.4. Spatiotemporal Pattern of Phylloporia in China

**Figure 4:**
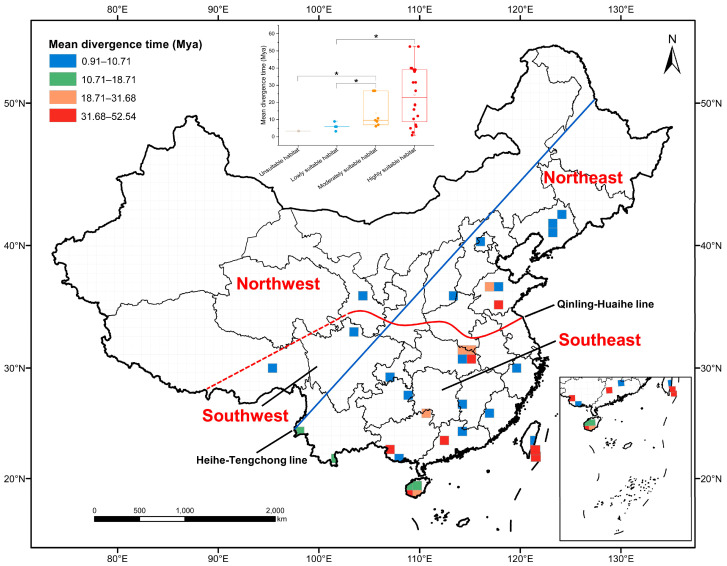
Spatiotemporal pattern of *Phylloporia* in China and point box line diagram of mean divergence times of *Phylloporia* in the four groups of grid cells classified by the suitability of habitat for *Phylloporia*. The map of China is divided into 100 km × 100 km grid cells and four geographic parts according to the Heihe–Tengchong line (blue) and the Qinling–Huaihe extension line (red). The mean divergence times of *Phylloporia* in grid cells are classified into four levels by Jenks’ natural breaks method. The asterisk indicates significant differences between two groups (Kruskal–Wallis non-parametric statistical test, *p*-value < 0.05) in ensemble modeling. Map adapted from National Geomatics Center of China (http://bzdt.ch.mnr.gov.cn/; accessed on 18 December 2023; review drawing number: GS(2019)1822).

After mapping the MDT of *Phylloporia* in each grid cell to map of China, we found that the grid cells with higher MDTs of *Phylloporia* are mostly distributed in southeastern China (Figure 4). Furthermore, we found that the grid cells grouped in the highly suitable habitat have the highest mean MDT of *Phylloporia* (24.39 Mya, Figure 4). Moreover, the Kruskal–Wallis non-parametric statistical test indicates that the group of grid cells in the highly suitable habitat has significantly higher MDT than the group in the lowly suitable habitat, and the group of grid cells in the moderately suitable habitat has significantly higher MDT than the groups in the unsuitable and lowly suitable habitats (*p*-value < 0.05, Figure 4).

### 3.5. Net Diversification Rate of Phylloporia in China

**Figure 5:**
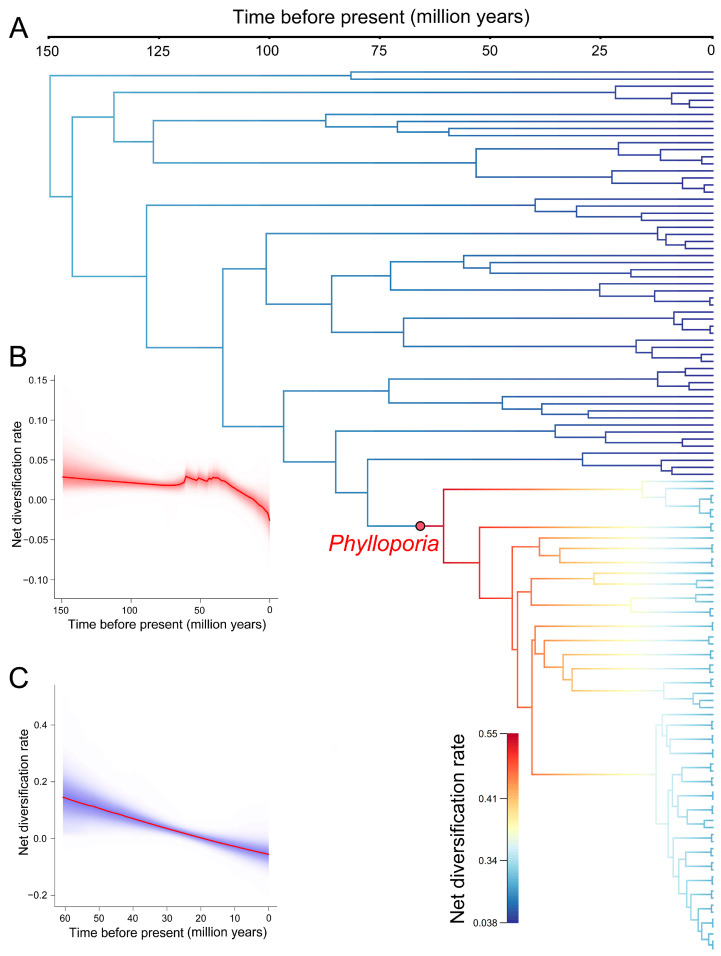
Net diversification rate inferred from the time-calibrated phylogenetic tree of representative Chinese genera in *Hymenochaetaceae*. (**A**) Per-branch net diversification rate averaged across posterior samples. The significant rate shift represented by the red dot indicates the lineage of the ancestor of *Phylloporia*. (**B**) Net diversification rate (red line) through time of representative Chinese genera in *Hymenochaetaceae*. Shaded red areas are 95% quantile ranges. (**C**) Net diversification rate (red line) through time of the lineage of *Phylloporia*. Shaded blue areas are 95% quantile ranges.

The per-branch net diversification rate was estimated to be between 0.038 and 0.55 per million years (Figure 5A). While the net diversification rate of the whole tree gradually slowed down through time, a significant rate speedup was inferred from the ancestor of *Phylloporia* (Figure 5A,B). The rate shift occurred when the ancestor of *Phylloporia* originated, approximately in 70 Mya, and was followed by a slowdown of the net diversification rate (Figure 5A,C).

## 4. Discussion

In the current study, a well-documented macrofungal genus *Phylloporia* was selected to explore its spatiotemporal pattern in China. This exploration helps to clarify the adaptive evolutionary scenario of *Phylloporia* in China.

### 4.1. The Highly Suitable Habitat of Phylloporia Mainly Concentrated in Warmer and More Humid Southeastern China

Ensemble modeling, which integrates data from separate models fitted with various modeling techniques [38,39], was used to predicted the current potential distribution of *Phylloporia* in China. In addition to providing relative measurement value for each predictor across all candidate models, ensemble modeling eliminates, or at least limits, model selection bias by avoiding the selection of a single best model [36,66].

The ensemble modeling method predicted that the highly suitable habitat mainly concentrated in southeastern China (Figure 2), where the climate is generally warmer and more humid than in other parts of China [67,68]. Moreover, from southeastern China onwards, the suitability for *Phylloporia* generally decreased. This phenomenon corresponds to the known distribution of *Phylloporia* in China [21,24,27,28,29,30,31,32]. In association with the environmental variables contributing to the distribution of *Phylloporia* (Table 1), we have a preliminary understanding that low temperature and drought restrict the growth of *Phylloporia*.

### 4.2. The Chinese Ancestor of Phylloporia Originated from Southeast China

We further explored the origin and dispersal of *Phylloporia* in China. Previously, the divergence times of *Hymenochaetales* were repeatedly estimated to be approximately 200 Mya [48,57]. However, no such study focusing specially on *Phylloporia* was performed. For the first time, the ancestor of *Phylloporia* in China was evaluated to originate in 77.74 Mya; meanwhile, the divergence time of each Chinese species of *Phylloporia* was also estimated (Figure S4). When mapping these times to the geographic distribution of *Phylloporia* in China, it is shown that the species of *Phylloporia* located in Southeast China have higher MDTs than other species (Figure 4), which also corresponds to the geographic suitability of *Phylloporia* (Figure 2); namely, the Chinese species of *Phylloporia* located in the moderately and highly suitable habitats have higher MDTs than other species (Figure 4). This phenomenon indicates that the species of *Phylloporia* in China may originate from Southeast China and later disperse to other geographic parts, which is also supported by the reconstruction of ancestral geographic distribution (Figure 3). Ensemble modeling indicates that the host plant, namely angiosperm trees for almost all species of *Phylloporia* (Table S1), is one of the restricting variables to the distribution of *Phylloporia* (Table 1). Previous research on the evolutionary history of Chinese angiosperm flora suggested that southeastern China has more ancient angiosperms [16]. Therefore, it is reasonable to postulate that the evolutionary history of *Phylloporia* at least partially corresponds to the spatiotemporal pattern of their host plants.

### 4.3. The Spatiotemporal Pattern of Phylloporia in China Is a Result of Adaptive Evolution

The net diversification rate indicates the rate of speciation minus that of extinction. This rate is a critical determinant of species diversity and may reflect the adaptive level of a lineage to special niches [69]. In macrofungi, a study at a large taxonomic scale, viz., mushroom-forming fungi, revealed that the net diversification rate was positively related to the expansion of gymnosperms and the increase in paleotemperature [70], which provides partial evidence for the adaptive evolution of mushrooms. Another study also suggested that the net diversification rate explosion of ectomycorrhizal fungi in *Agaricomycetes* resulted from their coevolution with angiosperms [71]. To test whether the spatiotemporal pattern of *Phylloporia* in China is a result of adaptive evolution, the net diversification rate per branch and through time was determined. After the divergence of the ancestor of *Phylloporia*, the net diversification rate of this lineage suddenly had an increase and then gradually slowed down (Figure 5). This kind of speedup and slowdown is often identified when suitable niches are available and rapidly diversifying species fill available niches during adaptive evolution, respectively [72,73]. Moreover, this rate change fits well with the evolutionary scenario that the ancestor of *Phylloporia* originated in southeastern China with highly and moderately suitable habitats and thus presented rapid diversification, and then the net diversification rate decreased after the early diversified species of *Phylloporia* occupying the suitable habitat in southeastern China and dispersing to other parts of China with lowly suitable and unsuitable habitats. It suggests that the diversification of *Phylloporia* may have been limited by niche suitability. During this evolutionary process of *Phylloporia*, its parasitism on angiosperms and pileate fruitbodies with poroid hymenophores may represent the most important adaptive biological traits to occupy the newly generated suitable habitats according to our previous study [26]. So, it is concluded that the suitability of habitat, including precipitation, temperature and host plant, indeed shapes the spatiotemporal pattern of *Phylloporia*.

## 5. Conclusions

In summary, we predicted the current potential distribution of *Phylloporia* in China using ensemble modeling, which suggests that the highly suitable habitat mainly concentrated in southeastern China. Furthermore, it is concluded that the Chinese ancestor of *Phylloporia* originated in 77.74 Mya from Southeast China, where the lineage of *Phylloporia* rapidly diversified with the aid of the highly and moderately suitable habitats for the growth of *Phylloporia*. Then, after occupying the available suitable niches during adaptive evolution, the diversification of *Phylloporia* in Southeast China gradually slowed down. In addition, due to the lowly suitable and even unsuitable habitats, the lineage of *Phylloporia* dispersal to other parts of China also diversified with a low rate. This adaptive evolutionary scenario of *Phylloporia* provides the first understanding for the spatiotemporal pattern of macrofungi, which hopefully will attract more attention to the macroevolution of macrofungi.

## Figures and Tables

**Table 1:** Environmental variables used to predict the current potential distribution of ***Phylloporia*** and their importance scores to ensemble modeling.

Variable	Description	Unit	Score
Bio1	Annual mean temperature	°C	–
Bio2	Mean diurnal temperature range	°C	–
Bio3	Isothermality (Bio2/Bio7) (×100)	%	–
Bio4	Temperature seasonality (standard deviation ×100)	°C	0.45
Bio5	Max temperature of the warmest month	°C	–
Bio6	Min temperature of the coldest month	°C	0.11
Bio7	Annual temperature range (Bio5-Bio6)	°C	–
Bio8	Mean temperature of the wettest quarter	°C	–
Bio9	Mean temperature of the driest quarter	°C	–
Bio10	Mean temperature of the warmest quarter	°C	–
Bio11	Mean temperature of the coldest quarter	°C	–
Bio12	Annual precipitation	mm	–
Bio13	Precipitation of the wettest month	mm	0.44
Bio14	Precipitation of the driest month	mm	–
Bio15	Precipitation seasonality (coefficient of variation)	%	–
Bio16	Precipitation of the wettest quarter	mm	–
Bio17	Precipitation of the driest quarter	mm	–
Bio18	Precipitation of the warmest quarter	mm	–
Bio19	Precipitation of the coldest quarter	mm	–
Altitude	Altitude	m	0.34
Host plant	Host plant	tree/km^2^	0.01

The dash (–) means that the environmental variable is not included for modeling the geographic distribution due to autocorrelation.

## Data Availability

The original contributions presented in this study are included in the article/supplementary material. Further inquiries can be directed to the corresponding author.

## Supplementary Materials

The following supporting information can be downloaded at: https://www.mdpi.com/article/10.3390/jof10110780/s1, Figure S1: Density of the host plants of *Phylloporia* distributed in China. Map adapted from National Administration of Surveying, Mapping and Geoinformation of China (http://www.ngcc.cn/; review drawing number: GS(2019)1822); Figure S2: Correlation of the 21 environmental variables (Table 1) used for predicting the current potential distribution of *Phylloporia* in China; Figure S3: The evaluation scores of ensemble model by the Committee Averaging (CA) and Weighted Mean (WM) methods separately according to the value of the area under the receiver operator characteristic curve (AUC) and the true skill statistic (TSS); Figure S4: Time-calibrated phylogenetic tree of representative Chinese genera (each genus is represented by its corresponding species) in *Hymenochaetaceae*, with all species of *Phylloporia* in China (highlighted in orange color). The divergence time of *Phylloporia* in China (labeled with a red pentagram) was estimated to be 77.74 Mya (stem age) with a 95% highest posterior density (HPD) of 69.22–86.82 Mya; File S1: The alignment used for generating the time-calibrated phylogenetic tree; Table S1: Known records of *Phylloporia* used for predicting the current potential distribution of *Phylloporia* in China; Table S2: Information of collections used for generating the time-calibrated phylogenetic tree.
