# Supplementary material for: Spatiotemporal Pattern of a Macrofungal Genus Phylloporia (Basidiomycota) Revealing Its Adaptive Evolution in China
Source: J Fungi (Basel). 2024 Nov 10;10(11):780. doi: 10.3390/jof10110780 (PMC11595563; doi:10.3390/jof10110780)
Supplement: Supplementary file 1 [file jof-10-00780-s001.zip › File S1.pdf]

File S1 The alignment used for generating the time-calibrated phylogenetic tree.

>Lyomyces_crustosus_LWZ_20170815-23
ATGAATTTGCA----------AGTGAGA------------------------------CTTGATGCTG--
GCAGT---------------GTAATG-CTGCA-TGTG-CTCGGT------------------------------
--TTC--------CGTCAAATCC--A--CAT-----------------TA-CACC--TGTGCA-
CTTTCAAA--
---------------------------------GCTCTGTAGT-----------------------CCTCCGT--
-------------------------AATGGGAGCC---

---------------------GCGGCTGT---

---------------------------AATGGC-TGCTGGGGCTACACC-TTTGC----CCATACAA-
TTAC-------------AA---ACTCTGTATTTTGTCTTGTAGAAT-GT---CT-AATGGC---CT-TT--
--RTTGGCTTAAC------
ATAATACAACTTTCAACAACGGATCTCTTGGCTCTCGCATCGATGAAGAACGCAGCGAAATGCG
ATAAGTAATGTGAATTGCAGAATTCAGTGAATCATCGAATCTTTGAACGCACCTTGCGCTCCTTG
G-TATTCCGAGGAGCATGCCTGTTTGAGTGTCATTAAATCATCAATTCTC--------AAC-
TTCTGTAAT------------------GGTTGTTG------TTGAATTGGAC-TTGGGA----------
CTTGCTGAT-TCTA---------------------ATGTTGAACTGGCTTCCCTCAAATGCATTAGC-
TGAAGTCTTA----------------C-AA---GCA-GCATAT------ATGCGGTGTGATAATATC---
----------------TTCACTG-----CAT-TGCTC--TCGTT-GAATTCGGC-TTC-----
TAATGGTCCTTCAAT-------------TGGAC-AA---------------------------------
TACTTTGACAATTTGACCTCATCCCCTAGTAACTGCGAGTGAAGCGGGAAGAGCTCAAATTTAA
AATCT-G-GCG-GTTT----CACCGTCCGAGTTGTAATCTGGAGAAGCATTTT-CTGCGTTG-
GACCGTGTACAAGTCTCTTGGAATAGAGCGTCATAGAGGGTGAGAATCCCG-
TCCATGACACGG-AC-TACCAGTGC-
TTTGTGATATGCTCTCAAAGAGTCGAGTTGTTTGGGAATGCAGCTCAAAATGGGTGGTAAATTCC
ATCTAAAGCTAAATATTGGCGAGAGACCGATAGCGAACAAGTACCGTGAGGGAAAGATGAAAA
GCACTTTGGAAAGAGAGTTAAACAGTACGTGAAATTGTTGAAAGGGAAACGCTTGAAGTCAGTC
GCGTTGTCCGGGACTCAGCCTTGCT---ATTTGCTTGGTGTACTTCCTGG-T---
TGACGGGTCAACATCAA-TTTT-GGC-TGGTGGATAAAGGCTGAGAGAAGGTGGCATCTTC----
GGATGTG-TTATAGC-TCTTGGTT-GCAT-ACGCTGGCTGGGATTGAGGT-CTGCAGCACGCCT--
----TT-T-TGGCC-------GGGGT---------------------------TC-GCCCACG-T-
AACGTGCTTAGGAT-
GTTGGCATAATGGCTTTAAGCGACCCGTCTTGAAACACGGACCAAGGAGTCTAACATGCCTGCG
AGTATTAGAGTGGAAAACTCTTGTGCGAAATGAAAGTGAAA-GTTGGGAC-----TCTT--------
----------TGCGG--AG----GCACCAACGCCCGGCCCTGAAG-----
TTTACTGACGGTGCTGCGGTAGAGCATGTATGTTGGG----
ACCCGAAAGATGGTGAACTATGCCTGAATAGGGTGAAGCCAGGGGAAACTCTG-
GTGGAGGCTCGTAGCGATTCTGACGTGCAAATCGATCGTCAAATTTGGGACGGTGTGTC--AT--

-
CGTTGATAAAGTAAGGGAAAAGAAATGATATTACCTTACTACTAGTGTCGTCCAAGTACTCGTG
CCAGAAGACTCGGTAAGGCGAGAGACGCAAACGTTAGTCGTCTTAATCAGGCGTAAAGGGTTT
GTAGGCGGCTTTAAGTATTTTA--AGTATTTTAAGTATTTT--------------------------------
AAGTATTTATT---

ATATTTAAAATCAATTAAAGCTAGAATCAAAAAGAGGTCACATTAAATAATACTTGGT--
GTAAGGTTGGTATCTTAAAACCCCAAGTAGAATAC-TAACGGCGAAGGC-
TTTTTTCCAATAATGATTGACGCTGAGAAACTAAGGTGAGGATAGAAAAAAGGATTAGAGACCC
TCGTATCCCTCACTGTCAACGATGAATGGTAGTTACTAGATAAATAATTATTAT-------
TTAATTTTTTTAAATAATTACAAATCTAGAGACGATGTTAACACGAAAACCATTCCGCCTTGTGA
GTACAACTGCAAAGTTGAAAACAAAAAAATTAGTCGGTTTCGGAG--CAAACGAAGTGAAGC-
ATGTTATTTAATGGGTACTGGTGAATTCGAGGCTGGTATCTCCAAGGATGGCCAGACTCGCGAA
CACGCTCTTCTCGCTTTCACTCTCGGTGTGCAGCAACTGATCGTTGCTGTCAACAAAATGGATTC
CACTAAGGTCAGTTTGAGATCTTGG--------CCATGCACATTCTCGCTA--ACGTTCG--------
GTTCTGTAGTGGTCTGGCCCTCGTTTCGAGGAAATCGTCAAGGAAGTCAGCGGCTTCATCAAGA
AGGTCGGTTACAACCCGAAGAATGTCGCCTTCGTCCCCATCTCTGGGTGGCATGGTGACAACAT
GATCGAGGAGTCCGCGAAGTGAGTGATCGCG-TTCTGTC--------TTTGAATA-----
TTACTTATCTAACGAGAG-
TGCAGCATGCCTTGGTACAAGGGTTGGTCCAAGGAAGTCAAGGGCG---------
GCGGTGCCGTC-
ACCGGCAAGACTCTTCTTGAGGCGATCGACGCGATCGAGCCGCCAGTACGGCCGACTGACAAG
CCCCTCCGCCTTCCCCTCCAG------
ATCTCTGTTGGTTCTTATTCGGCCCCTGTCATTGACTTCTTGGAGGAATGGGGTCTGGAAGGATT
AGAAGAGAACGCGCATTCCTCTCCCG------
GACTAACGAAGGTGTTCGTCAATGGGGTCTGGATGGGTATTCACAGGGAGTCGTCCAATCTATT
GGAGACAATCAGGAAACTGCGGCGTCGAG---
ACGATATCAGTCCCGAGGTCTCTGTGGTGCGCGACATTCGTGAGCGC----------------------
----

>Xylodon_subflaviporus_Dai_16413
CTGAGTTTTGA------------GTGGG--------------------------------CTTGATGCTG--
GCATC----------------TCTCGA--TGCA-TGTG-CTCAGC------------------------------
--CCC--GCT----CTCAAATCC--A--CTC-----------------AA-CACC--TGTGCA-
CTTTCAAA--
-------------------------------------GTTGTAGT------------------------CCTCCGT---
-------------------------AATGGGAGTC--

--------------------GCAAGTCTTT---

--------------------------ATTGGC-TTGTTGGGACTGCAC-TTTGC------CATTTA-TCAC-
------------AA---ACGATTTATATTGTTTTGTAGAAT-G-----T-CTA-GCCT-CT-TA----
GTAGGTGAAAC------
TTAATACAACTTTCAACAACGGATCTCTTGGCTCTCGCATCGATGAAGAACGCAGCGAAATGCG
ATAAGTAATGTGAATTGCAGAATTCAGTGAATCATCGAATCTTTGAACGCACCTTGCGCTCCTTG
G-TATTCCGAGGAGCATGCCTGTTTGAGTGTCGTGAAATTATCAACTCCC--------ATT-
CTTTGTTGA--------------------------TTGG-TGAGCTTGGAC-TTGGGGA-C---------
TGCTGGT-GC----------------------------AAATCAGCTTCCCTTGAATGAATTAGC-
TGGAATTTGA----------------T-TC---GCA-GCATAT------ATGCGGTGTGATAATGTC---
----------------GTCACTG---------TGTAGC-TCGGA-TTGTCTGGC-TTC-----
TAATCGTCCTTCA---------------CGGAC-AA------------------------------------
CTTGATCTTTTGACCTCATCCCCTAGTAACTGCGAGTGAAGCGGGAAAAGCTCAAATTTAAAATC
T-G-ACA-GTTT----CACTGTCCGAGTTGTAATCTGGAGAAGCATTTT-CCGTGTCG-
GACCGTGTACAAGTCTCTTGGAATAGAGCGTCATAGAGGGTGAGAATCCCG-
TCCATGACACGG-AC-TACCGATGC-
TTTGTGATATGCTCTCGAAGAGTCGAGTTGTTTGGGAATGCAGCTCAAAATGGGTGGTAAATTCC
ATCTAAAGCTAAATATTGGCGAGAGACCGATAGCGAACAAGTACCGTGAGGGAAAGATGAAAA
GCACTTTGGAAAGAGAGTTAAACAGTACGTGAAATTGTTGAAAGGGAAACGCTTGAAGTCAGTC
GCGTTGATTGAGACTCAGCCTTGC----TTT-GCATGGTGTACTTCTCAG-T---
TAACGGGTCAACATCAA-TTTC-AGT-TGGTGGATAAAGGCATTGGGAATGTGGCACCTTC----
GGGTGTG-TTATAGC-CCTTTGTT-GCAT-ACACTGACCGGGATTGAGGA-ACGCAGCACGCCA-
-----CT-A-AGGCC-------AGGGA----------------------TT---CG-TCCCATG-T-
AACGTGCTTAGGAT-
GTTGGCATAATGGCTTTAAGCGACCCGTCTTGAAACACGGACCAAGGAGTCTAACATGCCTGCG
AGTATTAGGGTGGAAAACCCTTGTGCGTAATGAAAGTGAAA-GTTGGGAC-----CTCT-------
-----------CGCGA--GG----GCACCGACGCCCGGCCCTGAAG-----
TTTACTGACGGCGCTGCGGTAGAGCATGTATGTTGGG----
ACCCGAAAGATGGTGAACTATGCCTGAATAGGGTGAAGCCAGGGGAAACTCTG-
GTGGAGGCTCGTAGCGATTCTGACGTGCAAATCGATCGTCAAATTTGGGAAGGTGTGTC--AC--
-
TTTTGATAAGGTAAAGGAAAATAAATGATATTACTTTACTATTAGTGTCGTCCAAGTACTCGTGC
CAGAAGACTCGGTAAGGCGAGAGACGCAAACGTTAGTCGTCCTGATCAGGCGTAAAGGGTTTG
TAGGCGGCTTTAAGTATGGATTCCTTCTAAATAACATTAC-------------------------------
-TCTATTTTTAG-----------GGTAACGAGT--

CCTCCCCTATGGGGCCAGGAGCAGAAGGGAAAAATCAATTAAAGCTAGAATCAAATAGAGGTC
AAATTAAATAATACTTGGT--GTAAGATTGGTATCTTTAAAAACCAAGCGGAATAC-
TAACGGCGAAGGC-
TTTTTTCCATTAATGATTGACGCTGAGAAACTAAGGTGAGGACAGAAAAAAGGATTAGAGACCC
TCGTATTCCTCACTGTCAACGATGAATGGTAGTTACTAGATGACAAGATATTGTACTTAAATAAA

TAAGGATATGTCTAAAAAAATCTAGAGACGATGTTAACACGAAAACCATTCCGCCTTGTGAGTA
CGACTGCAAAGTTGAAAACAAAAAAATTAGTCGGTTTCGGAG--CAAACGAAGTGAAGC-
ATGTTATTTAATT------
GGTGAGTTCGAGGCTGGTATCTCGAAGGATGGCCAGACCCGAGAGCACGCTCTTCTTGCCTTCA
CTCTTGGTGTGCGTCAGCTCATCGTCGCCATCAACAAAATGGACACCACCAAGGTTCGTACCGA
GTTTGTCGG------GTACTTGGAAGCTTTCTG--ACATGTC---------
GATGACAGTGGTCTGAGGCCCGTTACGAAGAAATCGTCAAGGAAACCTCCAACTTCATCAAGA
AGGTCGGATACAACCCGAAGCAGGTTGCCTTCGTTCCGATTTCGGGATGGCACGGTGACAACA
TGTTGGAGGAGTCCTCCAAGTAAGTTTTGTCT-TCTCGAG-----TGCGGAGAGTT-----
TTGAAACTGACGACTCCG-AACAGCATGCCGTGGTACAAGGGCTGGAAGAAGGAGACCAAGG-
-----------CCGGTGAAGTC-AAGGGCAAGACCCTCCTTGAGGCCATCGATGCC-----------
----
ATTTCCGTCGGTTCTTACTCGGCACCTGTCATCGAATTCTTGGAAGAATGGGGTTTGGAAGGTTT
GGAGGAGAATTCACG---GTCCTCCAG---
CGGCTTGACCAAGGTGTTCGTCAATGGTGTTTGGATGGGTGTTCATCGAGACCCCGCAAATCTC
GTCCGGACTATACGAACTCTGCGTCGTCGTG---
ATGATATCAGTCCCGAAGTGTCCGTCGTCCGTGACATTCGAGAACGGGAGTTGAGGATTTACAC
AGACGCCGGCCGTGTCTGTCGACCACTCTTCGTGGTGGAGGATCACGACCTCAAGCTGACCCG
AAGCCACGTCAGGTGGTTGCAGCAGAAGTACAGGG-----------------------------------
----

AGTCGGATCCGGCGGGCAAAGCCTACACCTGGGGATCGCTCGTCAAGGAGGGTATCGTCGAGT
ATCTTGACGCGGAAGAAGAGGAAACCATTATGATTTGTATGACTCCTGACGATCTCCTCGAAAC
ACGACTTCTCAGGCAAGGTATCAACCCGCG---
CGCCGACATGGAATTAGAGCCTTCTGCACGGCTCAAACCACCACCTACT------
GCTCACACCTGG--
>Phylloporia_alyxiae_GC_1604-28

TCCCCTAGTAACTGCGAGTGAAGCGGGAAGGGCTCAAATTTGTAATCT-G-GCG-GCC--
TCTGGCCGTCCGAGTTGTAATCTGTAGAGGCATTGT-CCGCATCA-
GCCCGTGTACAAGTCTCCTGGAATGGAGCGTCATAGAGGGTGAGAATCCCG-
TCCGTGACATGG-AC-CACTGATGC-
TTTGTGAGGTGCCCTCAAAGAGTCGCGTTGTTTGGGAATGCAGCGCAAAATGGGTGGTAAATTC
CATCTAAGGCTAAATATTGGCGAGAGACCGATAGCGAACAAGTACCGTGAGGGAAAGATGAAA
AGCACTTTGAAAAGAGAGTTAAACAGTACGTGAAATTGTTGAAAGGGAAACGCTTGAAGTCAGT
CGCGTCTTGAGGCACTCAGCCTTAC----TTCGGTTTGGTGTACTTGCCTT-A---
GGACGGGTCAACATCAA-TTTT-GGC-
TGGTGGAGAAGGATGAGGGGAATGTAGTGTCGTTTCGACGATGTG-TTATAGC-CTCTTGTC-
GAAT-ATGCTGGCTGGGATTGAGGAGCTGCAGCACGCCC-----CTT-G-TGGCC-------
AGAGG---------------------TT---CG-CCTCATG-T-AACGTGCTTAGGAT-
GTTGGCATAATGGCTTTAAGCGACCCGTCTTGAAACACGGACCAAGGAGTCCAACATGCTTGCG
AGTGTTCGGGTGGAAAACCCTTGCGCGTAATGAAAGTGAAA-GTTGGGAG-----CCTC-------
-----------TGTAA--GGAGGTGCACCGACGCCCGGCTCTGACG-----
TTCTCTAATGGTGCCGCGGTAGAGCAAGTGTGTTGGG----
ACCCGAAAGATGGTGAACTATGCCTGAATAGGGCGAAGCCAGAGGAAACTCTG-
GTGGAGGCTCGTAGCGATTCTGACGTGCAAATCGATCGTCAAATTTGGG------------------
----

>Phylloporia_alyxiae_Chen_1182

TCCCCTAGTAAATGCGAGTGAAGCGGGAAGGGGTCGAATTTGTAATCT-G-GCG-GCC--
TCTGGCCGTCTGAGTTGTAATCTGTAGAGGCATTGT-CCGCATCA-
GCCCGTGTACAAGTCTCCTGGAATGGAGCGTCATAGAGGGTGAGAATCCCG-
TTCATGACATGG-AC-CACTGATGC-
TTTGTCAGGTGCCCTCAAAGAGTCGCGTTGTTTGGGAATGCAGCGCAAAATGGGTGGTAAATTC
CATCTAAGGCTAAATATTGGCGAGAGACCGATAGCGAACAAGTACCGTGAGGGAAAGATGAAA
AGCACTTTGAAAAGAGAGTTAAACAGTACGTGAAATTGTTGAAAGGGAAACGCTTGAAGTCAGT
CGCGTCTTGAGGCACTCAGCCTTAC----TTCGGTTTGGTGTACTTGCCTT-A---
GGACGGGTCAACATCAM-TTTT-GGC-
TGGTGGAGAAGGATGAGGGGAATGTAGTGTCGTTTCGACGATGTG-TTATAGC-CTCTTGTC-
GAAT-ATGCTGGCTGGGATTGAGGAGCTGCAGCACGCCC-----CTT-G-TGGCC-------
AGAGG----------------------TT---CG-CCTCATG-T-AACGTGCTTAGGAT-
GTTGGCATAATGGCTTTAAGCGACCCGTCTTGAAACACGGACCAAGGAGTCCAACATGCTTGCG
AGTGTTCGGGTGGAAAACCCTTGCGCGTAATGAAAGTGAAA-GTTGGGAG-----CCTC-------
-----------TGTAA--GGAGGTGCACCGACGCCCGGCTCTGACG-----
TTCTCTAATGGTGCCGCGGTAGAGCAAGTGTGTTGGGCTTAACCCGAAAGATGGTGAACTATGC
CTGAATAGGGCGAAGCCAGAGGAAACTCTG-
GTGGAGGCTCGTAGCGATTCTGACGTGCAAATCGATCGTCAAATTTGGG------------------

----

>Phylloporia_chrysites_13669
ACGAGCCTTGA-----A-AG-TGTAAAG---------------------------------CCTGATGCTG--
GCGCG----------------GAGACG--CGTA-TGTG-CACGGC-------------------------------
---TTT--ACG-----CTCAATCT--A--CTT-------------------AA-CCCT--TGTGCA-
CTTTTATA---AGG----------------------
---------------------------------------CGAGTAGG------------------C-----TTTTGCTC--
-----------------------------TTTGAAG-------------CAACCAAGTGGTGAGTAACGCGAAC-
---GTGC-------------------------------------

------------------------------GTGA--

-------------------------GCATTAGTA-----------------GCCTGAA--------AGCGAGGG---
--------------------ACCTTCATTT------------AGAG---AGCATCGTCGAAAGC-TTTAA----
-CTTTTT----AT-------------CA---ACCTTTTGT-ATGTCTTGTGAAT--G-----T-GTT-GC-
T-CC-TC----GTGGGCGAAAG-------
AAATACAACTTTCAACAACGGATCTCTTGGCTCTCGCATCGATGAAGAACGCAGCGAAATGCGA
TAAGTAATGTGAATTGCAGAATTCAGTGAATCATCGAATCTTTGAACGCATCTTGCGCCCTCTGG

-TATTCCGTAGGGCATGCCTGTTTGAGTGTCATGCTAATCTCAAACCTC--------AAG-
CCTTTCA--------------TAA----GGCG-----CTG-ATGGTTTGGAG-TTGAAGG-C--------
TTTGCTGAT-GGGCA--------------CCTTT--AGGTGTGTCAGCTCCTTTGAAAAGCATTAGC-
TGGACTTTGG----------------T-TC---GCA-TGG-----------ATGGTGTAATAATAA-----
----------AGTG-TTCAC----CAGAAG-TACATT-CCTAA-TGGGTCTGC-TTC-----
TAATCGTCTT------------------CGGAC-AA--------GG-TCC---TTTGT-GAC----C-
TTTGCTTGACTGATTGACCTCA---

>Phylloporia_clausenae_Yuan_3528

TCCCCTAGTAACTGCGAGTGAAGCGGGAAGAGCTCAAATTTGTAATCT-G-GTG-GTC--
TCTGGCCACCCGAGTTGTAATCTGTAGAGGCGTCGT-CTGCGTCG-
GCCCGTGTACAAGTCTCCTGGGATGGAGCGTCATAGAGGGTGAGAATCCCG-
TCCATGACACGG-AC-CACCGATGC-
TCTGTGAGGCGCCCTCAAAGAGTCGCGTTGTTTGGGAATGCAGCGCAAAGCGGGTGGTAAATT
CCATCTAAGGCTAAATATTGGCGAGAGACCGATAGCGAACAAGTACCGTGAGGGAAAGATGAA
AAGCACTTTGAAAAGAGAGTTAAACAGTACGTGAAATTGTTGAAAGGGAAACGCTTGAAGTCA
GTCGCGTCTCGAGGCACTCAGCCTTGC----TTCGGCTCGGTGTACTTGCCTT-A---
GGACGGGTCAACATCAA-TTTT-GGC-
CGGTGGACAAGGGCGAGGGGAATGTAGCGTTGTTTCGGCGACGTG-TTATAGC-CCCTTGTC-
AGAT-ACATTGGCTAGGATTGAGGA-CTGCAGCACGCCC------TT-G-TGGCC-------
GGGGG----------------------TT---CG-CCTCACG-T-AACGTGCTTAAGAT-
GTTGGCATAATGGCTTTAAGCGACCCGTCTTGAAACACGGACCAAGGAGTCTAACATGCTTGCG
AGTGTTCGGGTGGAAAACCCTTGCGCGTAATGAAAGTGAAA-GTTGGGAG-----CCTC-------
-----------TGTAAAGAGGGGTGCACCGATGCCCGGCTCTGACG-----
TTTTCTGACGACGCCGCGGTAGAGCAAGCATGTTGGG----
ACCCGAAAGATGGTGAACTATGCCTGAATAGGGCGAAGCCAGAGGAAACTCTG-
GTGGAGGCTCGTAGCGATTCTGACGTGCAAATCGATCGTCAAATTTGGG------------------
----

>Phylloporia_clausenae_Cui8463
GGGCCTCTTGAGCTTGA-C---GTGGGG--------------------------------TCTGCTGCTG--
GCATA---------------AAAACA--CGCA-TGTG-CACGGC-------------------------------
---TCC--ATG-----CTCGATCC--A--TTC------------------AA-CCTC--TGTGCA-
CCTTTGCA---AAG---------------------
---------------------------------------CATAGTAGG------------------C------TTCGCTC-
------------------------------TTTGAA--------------GTGTTTAGTAATGAGTAAGGCGAGG-
---GTGC-------------------------------------

------------------------------GCGAGCATC----------------------------GATAGT-
CTGAAAGTGA------------------------------------AAGGTCT---
TCACCATTTGAAGTGCGTTCGGTAGCAAAGTAGGGTTTGAAGGTGCGCGCAAGCCACCAGATG
GCCTGAAAGCGAGAATGGACTTTGTTTACTGTTTGAAGTGACC-AGTAGCAAGTAA-GGTT----
---------GCGTGAGCG----------------GCCTGAA-------AGCGAACG-------------------
----TCCTTCATTT-----------GGAGGG-AGCGCAGTCGAAAGC-TTTGA------TCTTT----
AT--------------TA---ACTTGTCGT-ATGTCTTGTGGAT--GGTATAT-ATT-GC-T-CC-TG----
GTGAGCGAAATGA----
ATAATACAACTTTCAACAACGGATCTCTTGGCTCTCGCATCGATGAAGAACGCAGCGAAATGCG
ATAAGTAATGTGAATTGCAGAATTCAGTGAATCATCGAATCTTTGAACGCACCTTGCGCCCTTTG
G-TATTCCGAAGGGCATGCCTGTTTGAGTGTCATGTTAACCTCAAACCGC--------AGG-
CCTTTTG---------------TTA----GGCT-----CGA-GCGGTTTGGAT-TGGAGGA-T----------

-TGCTGGT-GCTT---------------GCTCT--GCATGCGTCGGCTCCTCTGAAAGGCATTAGC-
TGGACTTTGG----------------T-TC---GCG-CGG-----------ATGGTGTAATAGTAA-----
-----------CGTG-TTCGC----CAGAGG-CGCCTA-CCTAA-GGGGTCTGC-TTC-----
TAATCGTCTT------------------CGGAC-AA--------GG-TCC---TTTGTCGAC----C-
TTTGCTTGATCTTTTGACCTCATCCCCTAGTAACTGCGAGTGAAGCGGGAAGAGCTCAAATTTGT
AATCT-G-GTG-GTC--TCTGGCCACCCGAGTTGTAATCTGTAGAGGCGTCGT-CTGCGTCG-
GCCCGTGTACAAGTCTCCTGGGATGGAGCGTCATAGAGGGTGAGAATCCCG-
TCCATGACACGG-AC-CACCGATGC-
TCTGTGAGGCGCCCTCAAAGAGTCGCGTTGTTTGGGAATGCAGCGCAAAGCGGGTGGTAAATT
CCATCTAAGGCTAAATATTGGCGAGAGACCGATAGCGAACAAGTACCGTGAGGGAAAGATGAA
AAGCACTTTGAAAAGAGAGTTAAACAGTACGTGAAATTGTTGAAAGGGAAACGCTTGAAGTCA
GTCGCGTCTCGAGGCACTCAGCCTTGC----TTCGGCTCGGTGTACTTGCCTT-A---
GGACGGGTCAACATCAA-TTTT-GGC-
CGGTGGACAAGGGCGAGGGGAATGTAGCGTTGTTTCGGCGACGTG-TTATAGC-CCCTTGTC-
AGAT-ACATTGGCTAGGATTGAGGA-CTGCAGCACGCCC------TT-G-TGGCC-------
GGGGG----------------------TT---CG-CCTCACG-T-AACGTGCTTAAGAT-
GTTGGCATAATGGCTTTAAGCGACCCGTCTTGAAACACGGACCAAGGAGTCTAACATGCTTGCG
AGTGTTCGGGTGGAAAACCCTTGCGCGTAATGAAAGTGAAA-GTTGGGAG-----CCTC-------
-----------TGTAAAGAGGGGTGCACCGATGCCCGGCTCTGACG-----
TTTTCTGACGACGCCGCGGTAGAGCAAGCATGTTGGG----
ACCCGAAAGATGGTGAACTATGCCTGAATAGGGCGAAGCCAGAGGAAACTCTG-
GTGGAGGCTCGTAGCGATTCTGACGTGCAAATCGATCGTCAAATTTGGG------------------
----

GGTACCGGTGAATTCGAGGCTGGTATCTCAAAGGACGGTCAGACCCGCGAGCACGCTTTGCTT
GCGTTCACCCTCGGTGTGCGTCAGCTGATTGTTGCCGTGAACAAGATGGACACGGCTAAGGTTA
GT-GGCGCCATTGA--CCTCT-CTCTGACGAAATTAAATC--CCGTTTG-------
AATACTCCAGTGGTCTGAGGAACGCTTCAACGAAATTGTTAAGGAGACGTCGATCTTCATTAAG
AAGGTCGGCTACAACGCTAAGGCCGTAGCTTTTGTCGCTATCTCTGGATGGCACGGTGACAACA
TGCTTGAGGAATCGACCAAGTTCGTTTGACTT-TACCC-----TACAATATAAGCG-----
TGAGCTGACGTGACATCT-TTCAGTATGTCTTGGTACAAGGGTTGGTCGAAGGAGACGAAGG--
----------GTGGTGTCGTT-
AAGGGAAAGACACTTCTCGATGCCATTGATGCCATCGAACCCCCCGTCCGTCCAGAGAACAAG
CCCTTGCGACTTCCTCTCCAGGATGTGATTTCCGTTGGTTCTTTATCCGCTCCGGTACTCGAATTC
TTGGAGGAATGGGGGTTGGAGAGCCTCGAGGAAAATGCACA---AGCTGCAA------
ATTTAACCAAAGTTTTCGTGAATGGAGTATGGCTAGGAGTACATCGAGACCCGCACACTCTGGT
CAACACTCTTCGAACGCTTAGGAGAAAGG---

AGGATCTGAACGCCGAGGTTTCAATCGTTCGTGACATACGTGAAAAGGAGGTCCGTATTTATAC
CGACGCAGGACGCGTTCTTCGACCGCTTTTCATTGTCGAGGACAATAGATTAAAGCTGAGACGT
CAGCACCTGCGCTGGCTCCAGGCGAAGTGGAGGATT-------
GAGGAGAGGCCCAAAGACAGAGTCCT------------
GGATGAAGGCGAAGAATCAGCAGGCGAGACAGTAACTGAGCTAGAAGACGATGGGAAGCCAA
TAATACGGGAAGGGGTTCGAATGAAGAAGAAGAGGAAACCGGTCACCTGGAAAACGTTGTTCA
GCGACGGCGTTATTGAATACCTTGACGCAGAGGAGGAGGAAACGGTCATGATCTGCATGACAC
CAGAGGATTTGGTCGAATCTCGGTTAATGAGGCAAGGCCTGGATCCTCG---
AGCTGACACGGAGTTCGATGCGTCTACGCGTCTCAAGCCTGTACCGACC------
GCTCATAACTGGACACATTGCGAGATTCATCCCAGCATGATTCTAGGTATATGTGCAAGTATCAT
TCCGTTCCC
>Phylloporia_crataegi_Dai_11016

ACTGCGAGTGAAGCGGGAAGAGCTCAAATTTGTAATCT-G-GCG-GTC--
TCTGGCCGTCCGAATTGTAATCTGTAGAGGCGTCGT-CCGCGTCG-
GCCCGTGTACAAGTCTCCTGGGATGGAGCGTCATAGAGGGTGAGAATCCCG-
TCCATGACACGG-AT-CATCGATGC-
TTTGTGAGGCGCCCTCGAAGAGTCGCGTTGTTTGGGAATGCAGCGCAAAATGGGTGGTAAATTC
CATCTAAGGCTAAATATTGGCGAGAGACCGATAGCGAACAAGTACCGTGAGGGAAAGATGAAA
AGCACTTTGAAAAGAGAGTTAAACAGTACGTGAAATTGTTGAAAGGGAAACGCTTGAAGTCAGT
CGTGTCCCGTGGCACTCAGCCTTGC----TTTGGCTTGGTGTATTTGCCTT-G---
GGATGGGTCAACATCAA-TTTT-GGC-
CGGTGGATAAGGGTGAGGGGAATGTAGCGTTGTTTCGGCAACGTG-TTATAGC-CTCTTGTC-
AAAT-ACGTCGGCTGGGATTGAGGA-CTGCAGCACGCCC------TT-G-TGGCC-------

GGGGG----------------------TT---CG-CCCCATG-T-CACGTGCTTAGGAT-
GTTGGCATAATGGCTTTAAGCGACCCGTCTTGAAACACGGACCAAGGAGTCTAACATGCTTGCG
AGTGTTCGGGTGGAAAACCCTTGCGCGTAATGAAAGTGAAA-GTTGGGAG-----CCTC-------
-----------CGTGA--GGGGGTGCACTGACGCCCGGCCCTGATG-----
TTCTCTGACGGTGCTGCGGTAGAGCAAGTATGTTGGG----
ACCCGAAAGATGGTGAACTATGCCTGAATAGGGCGAAGCCAGAGGAAACTCTG-
GTGGAGGCTCGTAGCGATTCTGACGTGCAAATCGATCGTCGAATTTGGG------------------
----

>Phylloporia_crataegi_Dai_18133
ATGGGTTTTTG-----A-A---GCAGAG------------------------------TCTGCTGCTG--
GCGTG---------------GAAACG--CGCA-TGTG-CACGGC----------------------------
---CCT--GTG-----CTCGATCC--A--CTC-----------------AA-CCCC--TGTGCA-
CCTTTGCA---AAG-------------------
------------------------------------CGAGTAGG----------------C------TTCACAC-
---------------------------TTTGAAGG-----------
CGGGCCAGCGGCGAGTAAGGCGAGC---
GTGC---
--GTGA---------

---GCACCAGTA------
---------GCCTGAA-------AGTGG--C---------------------TCTTTCATTC-----------
GAAG---AGTGCGGTCGAAAGC-TTTGA------TTTTT----AT-------------CA---
ACCCTTTGT-ATGTCATGTGAAT--G-----T-GTT-GC-T-CC-TC----GTGGGCAAAGC-------
AAATACAACTTTCAACAACGGATCTCTTGGCTCTCGCATCGATGAAGAACGCAGCGAAATGCGA
TAAGTAATGTGAATTGCAGAATTCAGTGAATCATCGAATCTTTGAACGCACCTTGCGCCCTTTGG
-TATTCCGAAGGGCATGCCTGTTTGAGTGTCGTGTTTATCTCAAACCCC--------AAA-
CCTTTTG--------------TTA----GGTTT----CGG-ATGGTTTGGAC-TTGGAGG-C--------
TTTGCTGGT-GCGT---------------GCCCT--GTGCGCGTCGGCTCCTCTGAAAGGCATTAGC-
TGGACTCTGG----------------T-TC---GCA-AGG-----------ATGGTGTGATAGTAA-----
----------CGTA-TTCGC----CAGAAA-TGCTTG-CCTGA-TGGGTCTGC-TCC-----
TAATCGTCTT------------------CGGAC-AA--------GGTTCC---TTTGT-GAC----C-
TTTGCTTGACCGTTTGACCTCATCCCCTAGTAACTGCGAGTGAAGCGGGAAGAGCTCAAATTTGT
AATCT-G-GCG-GTC--TCTGGCCGTCCGAATTGTAATCTGTAGAGGCGTCGT-CCGCGTCG-
GCCCGTGTACAAGTCTCCTGGGATGGAGCGTCATAGAGGGTGAGAATCCCG-
TCCATGACACGG-AT-CATCGATGC-
TTTGTGAGGCGCCCTCGAAGAGTCGCGTTGTTTGGGAATGCAGCGCAAAATGGGTGGTAAATTC
CATCTAAGGCTAAATATTGGCGAGAGACCGATAGCGAACAAGTACCGTGAGGGAAAGATGAAA
AGCACTTTGAAAAGAGAGTTAAACAGTACGTGAAATTGTTGAAAGGGAAACGCTTGAAGTCAGT
CGTGTCCCGTGGCACTCAGCCTTGC----TTTGGCTTGGTGTATTTGCCTT-G---
GGATGGGTCAACATCAA-TTTT-GGC-
CGGTGGATAAGGGTGAGGGGAATGTAGCGTTGTTTCGGCAACGTG-TTATAGC-CTCTTGTC-
AAAT-ACGTCGGCTGGGATTGAGGA-CTGCAGCACGCCC------TT-G-TGGCC-------
GGGGG----------------------TT---CG-CCCCATG-T-CACGTGCTTAGGAT-
GTTGGCATAATGGCTTTAAGCGACCCGTCTTGAAACACGGACCAAGGAGTCTAACATGCTTGCG
AGTGTTCGGGTGGAAAACCCTTGCGCGTAATGAAAGTGAAA-GTTGGGAG-----CCTC-------
-----------CGTGA--GGGGGTGCACTGACGCCCGGCCCTGATG-----
TTCTCTGACGGTGCTGCGGTAGAGCAAGTATGTTGGG----
ACCCGAAAGATGGTGAACTATGCCTGAATAGGGCGAAGCCAGAGGAAACTCTG-
GTGGAGGCTCGTAGCGATTCTGACGTGCAAATCGATCGTCGAATTTGGGTTGATGTGAC--AT--
-CGAGGATAACGTAAGGGAAAAT-
AATGATATTACCTTACTATTAGTGTCGTCCAAATGCTCGTGCCAGAAGACTCGGTAAGGCGAGA
GACGCAAACGTTAGTCGTCTTGATCAGGCGTAAAGGGTTTGTAGGCAGCTTTAAGTATCCAA-
TTTAATGTTTATTATTAT-------------------------------AATATTTACA--------------
----

AGTTATAATATTTATTAAAGGATCAATTAAAGCTAGAATCTAGAAGAGGTGA----
ATACAATGCTTGAG-CTTAGGAGTACTAACCGCAAACATCAAGTAGAGTGC-
TAAGGGCGAAGGC-
AGTTTACCAATAAAGATTGACGCTGAGAAACGAAGGTGAGGATAGAAAAAAGGATTAGATACC
CTTGTATCCCTCACTGTCAACGATGAATGGTAGTTACTAGGGAGAA----------------------
----
ATGACCTAGAGGCGATGTTAACACGTAAACCATTCCGCCTTGTGAGTACGACTGCAAAGTTGAA

AACAAAAAAATTAGTCGGTTTCGGAG--CAAACGAAGTGAAGC-
ATGTTATTTAATTGGTACTGGTGAGTTCAAGGCTGGTATCTCGAAGGACGGTCAGACCCGCGAG
CACGCTCTGCTGGCTTTCACCCTCGGTGTGCGTCAGCTGATCGTTGCTGTGAACAAGATGGACA
CGGCTAAGGTTGGT-GGTGCCATTGA--C--CT-CCGTAACGAAGCTGAATT--GCGTTTG------
-
AATACTCCAGTGGTCTGAGGAACGATTCAACGAAATTGTTAAGGAGACATCGAACTTCATTAAG
AAAGTCGGCTACAACGCCAAGGCCGTACCTTTCGTCCCGATTTCTGGATGGTGCGGTGACAACA
TGCTTGAGGAGTCGAGCAAGTATGTTTGATTT-TGCCC-----TACAATGCAAGCG-----
TGTGCCGACGTGACGTCT-TTCAGTATGACTTGGTACAAGGGCTGGACCAAGGAGACGAAGG--
----------GCGGTGTCGTC-
AAGGGAAAGACTCTTCTCGATGCCATTGATGCCATCGAGCCCCCTGTCCGCCCAGAAAACAAA
CCCTTGCGACTTCCTCTCCAGGATGTGATTTCCGTTGGATCATTATCCGCTCCGGTACTCGAATT
CTTGGAGGAATGGGGCTTGGAGAGCCTCGAGGAAAATACGCA---AGCTGCAA------
ATTTGACCAAAGTTTTCGTGAATGGGGTATGGCTCGGAGTACACCGAGACCCGCACACCCTTGT
CAACACTCTTCGAACGCTCAGGAGAAAGG---
AGGATCTCAACGCTGAGGTTTCCATTGTTCGTGACATACGCGAAAAGGAAGTTCGCATCTACAC
GGACGCAGGACGCGTTCTTCGACCGCTCTTCATCGTCGAGGACAATAGATTAAAGCTGAGGCG
TCAGCACCTGCGCTGGCTTCAGGCGAAGTGGAGGATT-------
GAGGAGAGGCCCAAAGACAGGGTCTT------------
GGATGAAGGCGAAGAATCAGCAGGCGAGACAATAACTGAGGTAGAAGATGATGGGAAGCCAA
TAATACGGGAAGGGGTTCGAATGAAGAAGAAGAGGAAACCTGTCACCTGGAAGACATTGATTA
GCGACGGCGTTATTGAATACCTTGATGCAGAGGAGGAGGAAACGGTCATGATCTGCATGACAC
CAGAAGATCTGGTCGAATCTCGATTAATGAGGCAAGGCCTGGATCCTCG---
AGCAGACACGGAGTTCGATGCGTCTACCCGTCTCAAGCCTGTAGCAACT------
GCTCATAACTGGACACATTGCGAGATTCATCCTAGCATGATTCTAGGTATATGTGCAAGTATCAT
TCCTTTCCA
>Phylloporia_cylindrispora_Yuan_6144

TCCCATAGTAACTGCGAGTGAAGTGGGAAAAGCTCAAATTTGTAATCT-G-ATG-GCC--
TCCAGCCGTCCGAGTTGTAATCTGTAGAGGCGTCAT-CTGCGTCG-
GCCCATGTACAAGTCTCCTGGAATGGAGCCTCCTAGAGGGTGAGAATCCCG-
TCTGTGACATGG-AC-TACCGATGC-
TCTGTGAGGTGCCCTCGAAGAGTCGCGTTGTTTGGGAATGCAGCGCAAAATGGGTGGTAAATTC
CATCTAAGGCTAAATATTGGCGAGAGACCGATAGCGAACAAGTACCGTGAGGGAAAGATGAAA
AGCACTTTGAAAAGAGAGTTAAATAGTACGTGAAATTGTTGAAAGGGAAACGCTTGAAGTCAGT
CGTGTTCTGTGGCACTCAGCCTTGC----TTCGGCTCGGTGTACTTGCCTT-A---
GGATGGGTCAACATCAA-TTTT-AGC-
TGGTGGACAAGGGTGAGGGGAATGTAGCGTCGTTTTAGCGTCGTG-TTATAGC-CTCTTGTC-
AAAT-ACATCAGCTGGGATTGAGGA-CTGCAGCATGCTC------TT-G-TGGCG---
CCCGGGGGT----------------------TT---TG-CCCCACGTT-AATGTGCTTAGGAT-
GTTGGCATAATGGCTTTAAGCGACCCGTCTTGAAACACGGACCAAGGAGTCTAACATGCTTGCA
AGTGTTTGAGTGGAAAACTCTTGCGCGTAATGAAAGTGAAAGTTTGGGAG-----CCTC-------
-----------TGTGA-AGGGGGTGCACCGACGCCCGGCCCTGATG-----
TTTTCTGATGGTGCTGCGGTAGAGCAAGTATGTTGGG----
ACCCGAAAGATGGTGAACTATGCCTGAATAGGGTGAAGCCAGAGGAAACTCTG-
GTGGAGGCTCGTAGCGATTCTGACGTGCAAATCGATCGTCAAATTTGGG------------------
----

>Phylloporia_cylindrispora_Yuan_6148

TCCCATAGTAACTGCGAGTGAAGTGGGAAAAGCTCAAATTTGTAATCT-G-ATG-GCC--
TCCAGCCGTCCGAGTTGTAATCTGTAGAGGCGTCAT-CTGCGTCG-
GCCCATGTACAAGTCTCCTGGAATGGAGCCTCCTAGAGGGTGAGAATCCCG-
TCTGTGACATGG-AC-TACCGATGC-
TCTGTGAGGTGCCCTCGAAGAGTCGCGTTGTTTGGGAATGCAGCGCAAAATGGGTGGTAAATTC
CATCTAAGGCTAAATATTGGCGAGAGACCGATAGCGAACAAGTACCGTGAGGGAAAGATGAAA
AGCACTTTGAAAAGAGAGTTAAATAGTACGTGAAATTGTTGAAAGGGAAACGCTTGAAGTCAGT
CGTGTTCTGTGGCACTCAGCCTTGC----TTCGGCTCGGTGTACTTGCCTT-A---
GGATGGGTCAACATCAA-TTTT-AGC-
TGGTGGACAAGGGTGAGGGGAATGTAGCGTCGTTTTAGCGTCGTG-TTATAGC-CTCTTGTC-
AAAT-ACATCAGCTGGGATTGAGGA-CTGCAGCATGCTC------TT-G-TGGCG---
CCCGGGGGT----------------------TT---TG-CCCCACGTT-AATGTGCTTAGGAT-
GTTGGCATAATGGCTTTAAGCGACCCGTCTTGAAACACGGACCAAGGAGTCTAACATGCTTGCA
AGTGTTTGAGTGGAAAACTCTTGCGCGTAATGAAAGTGAAAGTTTGGGAG-----CCTC-------
-----------TGTGA-AGGGGGTGCACCGACGCCCGGCCCTGATG-----
TTTTCTGATGGTGCTGCGGTAGAGCAAGTATGTTGGG----
ACCCGAAAGATGGTGAACTATGCCTGAATAGGGTGAAGCCAGAGGAAACTCTG-
GTGGAGGCTCGTAGCGATTCTGACGTGCAAATCGATCGTCAAATTTGGG------------------

----

>Phylloporia_cystidiolophora_Dai13945

TCCCTTAGTAACTGCGAGTGAAGTGGGAAGAGCTCAAATTTGTAATCT-G-GCG-GTC--
TTTGGCCGTTCGAGTTGTAATCTGTAGAGGCGTCGT-CCGCGTCG-
GCCCATGTATAAGTCTCCTGGAATGGAGCGTCATAGAGGGTGAGAATCCCG-
TCCATGACATGG-AC-CACCGATGC-
TTTGTGAGGTGCCCTCGAAGAGTCGCGTTGTTTGGGAATGCAGCGCAAAATGGGTGGTAAATTC
CATCTAAGGCTAAATATTGGCGAGAGACCGATAGTGAACAAGTACCGTGAGGGAAAGATGAAA
AGCACTTTGAAAAGAGAGTTAAACAGTACGTGAAATTGTTGAAAGGGAAACGCTTGAAGTCAGT
CGTATCTTGGGGCACTCAGCCTTGC----TTTGGCTTGGTGTACTTGCCTT-G---
GGATGGGTCAACATCAA-TTTT-GGC-
CGGCAGACAAGGGTGAGGGGAATGTAGCGTCATTTTGGTGATGTG-TTATAGC-TCCACATC-
AAAT-GTGTCGGCTGAGATTGAGGA-TCGCAGCATGCCC------TT-G-TGGTC-------
AGAGG-----------------------TT---CG-CCTCATG-T-AATGTGCTTAGGAT-
GTTGGCATAATGGCTTTAAGTGACCCGTCTTGAAACACGGACCAAGGAGTCTAACATGCTTGCA
AGTGTTTGGGTGGAAAACCCTTGCGCGTAATGAAAGTGAAA-GTTGGGAG-----CCTC-------
-----------CGTGA--GGGGGTGCACCGACGCCCGGCCCTGATG-----
TTCTCTGGTGGTGCTGCGGTAGAGCAAGTATGTTGGG----
ACCCGAAAGATGGTGAACTATGCCTGAATAGGGCGAAGCCAGAGGAAACTCTG-
GTGGAGGCTCGTAGCGATTCTGACGTGCAAATCGATCGTCAAATTTGGG-------------------
----

>Phylloporia_cystidiolophora_Dai13953

TCCCTTAGTAACTGCGAGTGAAGTGGGAAGAGCTCAAATTTGTAATCT-G-GCG-GTC--
TTTGGCCGTTCGAGTTGTAATCTGTAGAGGCGTCGT-CCGCGTCG-
GCCCATGTATAAGTCTCCTGGAATGGAGCGTCATAGAGGGTGAGAATCCCG-
TCCATGACATGG-AC-CACCGATGC-
TTTGTGAGGTGCCCTCGAAGAGTCGCGTTGTTTGGGAATGCAGCGCAAAATGGGTGGTAAATTC
CATCTAAGGCTAAATATTGGCGAGAGACCGATAGTGAACAAGTACCGTGAGGGAAAGATGAAA
AGCACTTTGAAAAGAGAGTTAAACAGTACGTGAAATTGTTGAAAGGGAAACGCTTGAAGTCAGT
CGTATCTTGGGGCACTCAGCCTTGC----TTTGGCTTGGTGTACTTGCCTT-G---
GGATGGGTCAACATCAA-TTTT-GGC-
CGGCAGACAAGGGTGAGGGGAATGTAGCGTCATTTTGGTGATGTG-TTATAGC-TCCACATC-
AAAT-GTGTCGGCTGAGATTGAGGA-TCGCAGCATGCCC------TT-G-TGGTC-------
AGAGG----------------------TT---CG-CCTCATG-T-AACGTGCTTAGGAT-
GTTGGCATAATGGCTTTAAGTGACCCGTCTTGAAACACGGACCAAGGAGTCTAACATGCTTGCA
AGTGTTTGGGTGGAAAACCCTTGCGCGTAATGAAAGTGAAA-GTTGGGAG-----CCTC-------
-----------CGTGA--GGGGGTGCACCGACGCCCGGCCCTGATG-----
TTCTCTGGTGGTGCTGCGGTAGAGCAAGTATGTTGGG----
ACCCGAAAGATGGTGAACTATGCCTGAATAGGGCGAAGCCAGAGGAAACTCTG-
GTGGAGGCTCGTAGCGATTCTGACGTGCAAATCGATCGTCAAATTTGGGTATATGTGTC--ATA-

-TGAGGCTAACGTAAGGGAAAAT-
AATGATATTACCTTACTATTAGTGTCGTCCAAATGCTCGTGCCAGAAGACTCGGTAAGGCGAGA
GACGCAAACGTTAGTCGTCTTGATCAGGCGTAAAGGGTTTGTAGGCAGCTTTAAGTATCCAA-
TTTAATGTTTACTATTAT----------------------------------AATATTTACA----------------
----

TGTTATAATATTTATTAGAGGATCAATTAAAGCTAGAATCTAAAAGAGGTGA----
ATACAATGCTTGAG-CTTAGGAGTACTAACCGCAAACATCTAGTAGAGTGC-
TAAGGGCGAAGGC-
AGTTTTCCAATAAAGATTGACGCTGAGAAACGAAGGTGAGGATAGAAAAAAGGATTAGAGACC
CTTGTATCCCTCACTGTCAACGATGAATGGTAGTTACTAGGGAGAA------------------------
----
ATGACCTAGAGGCGATGTTAACACGTAAACCATTCCGCCTTGTGAGTACGACTGCAAAGTTGAA
AACAAAAAAATTAGTCGGTTTCGGAG--CAAACGAAGTGAAGC-
ATGTTATTTAATTGGTACTGGTGAGTTCGAGGCTGGTATCTCGAAAGACGGTCAGACCCGCGAG
CACGCTCTGCTTGCGTTCACCCTCGGCGTGCGTCAGCTGATCGTTGCCGTGAACAAGATGGACA
CAGCCAAGGTTGGT-GGCGCTATTGA--CCTCT-CCGTAATGAAGCTGAATC--GCGTTTG-----
--
AATACTCCAGTGGTCTGAGGAGCGATTCAACGAAATTGTTAAGGAGACATCGACCTTCATTAAG
AAAGTCGGCTACAACGCCAAGGCCGTACCTTGCGTCCCGATCTCCGGATGGCACGGTGACAAC
ATGCTTGAGGAGTCGACCAAGTACGTTTGATTT-TGCTC-----TACAATGTAAGCG-----
TGTGCCGACGTGACATCT-TTCAGTATGACTTGGTACAAGGGCTGGTCCAAGGAGACAAAGG--
----------GCGGTGTCGTT-
AAGGGAAAGACTCTCCTCGATGCCATTGATGCCATTGAGCCCCCTGTCCGCCCAGAGAACAAG
CCTTTGCGACTTCCCCTCCAGGATGTGATTTCCGTTGGATCATTATCCGCTCCGGTACTCGAATT
CTTGGAGGAATGGGGCTTGGAGAGCCTCGAGGAAAATGCACA---AGCTGCAA------
ATCTGACCAAAGTTTTCGTGAATGGGGTATGGCTCGGAGTGCACCGAGACCCGCACACTCTTGT
CAACACTCTTCGAACACTCAGGAGAAAGG---
AGGATCTCAACGCTGAGGTTTCCATTGTTCGTGACATCCGCGAAAAGGAAGTTCGCATCTACAC
GGACGCAGGACGCGTTCTTCGACCGCTTTTCATTGTCGAGGACAATAGATTAAAGCTGAGACGT
CAGCACCTGCGCTGGCTTCAGGCGAAGTGGAGGATT-------
GAGGAAAGGCCCAAAGACAGGGTCCT------------
GGATGAAGGCGAAGAATCAGCAGGCGAGACAATAACCGAGCTAGAAGACGATGGGAAGCCAA
TAATACGGGAAGGGGTTCAAATGAAGAAGAAGAGGAAGCCGGTCACCTGGAAGACGTTGATTA
GCGACGGCGTTATTGAATACCTTGATGCAGAGGAGGAGGAAACGGTCATGATCTGCATGACAC
CAGAAGATCTGGTCGAATCTCGGTTAATGAGGCAAGGCCTGGATCCTCG---
AGCAGACACGGAGTTCGATGCGTCTACGCGTCTCAAGCCTGTACCAACT------
GCTCATAATTGGACACATTGCGAGATTCATCCCAGCATGATTCTTGGTATATGTGCAAGTATCAT
TCCGTTCCC
>Phylloporia_dependens_Cui_13763
ATGAGTTTTGA-----A----ATCGGTG-------------------------------TCTGCTGCTG--
GTGCG----------------GAAACG--CACA-TGTG-CACGGC-----------------------------
---TCC--GTG-----CTCAATCC--A--CTC------------------AA-CACCCTTGTGCA-
CTTTGTCGAGGCGAGTAAGCTCTTCGTCGGTTCGGTCTAGTAACCAGTAAGGCGAAGGCACTTT

AGAGGTGTTGGTAGCTTGAACGCGAACGTCAATCTTTTGTTAGTCGGTCTAGTAGTCGGTAAGG
CGAAGGCAC-----GTTCGTGTCGGTAGCTTGAAGGTGAACGTCGACCGGTTTGAAAGG------
----TTGGTTAGTAGTTAGTAAGGCGAAGGCA----------------------------------
CGTCTGTGTTGGTAGCTTGAAGGGTGAACGTCGACCTTTTGTTGGTTCGGTCCGGTAACTAGTAA
GGCAAAGGCAAAGGCATTTTGGTGTTGGTAGCTTTTGGTGTCAGCAGCCT-----------------
------------GAACGCGAACGTCAACCTTTTG-----
TTAGGTTGGTCTAGTAGTTAGTAAGGTGAAGGCACGTTTGAGTCGGGGGGCAGCCTGAACGTG
AAGGTCGACCTTTTGTTAGGTT--GGTCTAGTAGTTAGTAAAG---TGAAGGCATGTCCGTG----
TCGATAGCTTGAATGTGAACGG----TCAACACTTTGTTGGG-TTGGTCTAGTAACTAGTAA-
GGCGG--AGGCACGTCCGTGTCGATA---------------GCTTGAA-------CGCGAACGTCAA-
--------------------CCTTTT------------------TGAAGGGCTGAAAGC-TTTGAAC------
-----TTAC-------------AA---ACGATTTGT-ATGTCCTGTGAAC--G-----T-GTT-GC-T-
CC-TT----GTGGGCGAAAT-------
AAATACAACTTTCAACAACGGATCTCTTGGCTCTCGCATCGATGAAGAACGCAGCGAAATGCGA
TAAGTAATGTGAATTGCAGAATTCAGTGAATCATCGAATCTTTGAACGCACCTTGCGCCCCTTGG
-TATTCCGAGGGGCATGCCTGTTTGAGTGTCATATTAATCTCAAACTCC--------GAG-
CCTTTCGTT------------------AGGTCTTG------GTGGTTTGGAA-TTGGGAA-------
GGGGTTGCTGGC-GTCGACCTT------GGGTTGTTG--
ACTTGACCGGGCTCCTCTGAAATGCATTAGC-TGGGCTTTGG----------------C-TC---
GCA-TGA-----------ACGGTGTAATAGTCAA--------------CTCA-TTCGCCG-----TAG-
TGCCTG-CCGGG-AGAGTCTGC-TTC-----TAATCGTCTT------------------CGGAC-AG---
---------------------------C---CTTTGGGCCTTTTACCCTTG-
CCCCTAGTAACTGCGAGTGAAGCGGGAAGAGCTCAAATTTGTAATCT-G-GCG-GTC--
TTGGGCCGTCCGAGTTGTAATCTGAAGAGGCATTGT-CCGCGTCG-
GACCGTGTACAAGTCTCCTGGAATGGAGCGTCATAGAGGGTGAGAATCCCG-
TCCATGACACGG-AC-CGCCGATGC-
TTTGTGAGGTGCCCTCGAAGAGTCGCGTTGTTTGGGAATGCAGCGCAAAATGGGTGGTAAATTC
CATCTAAAGCTAAATACTGGCGAGAGACCGATAGCGAACAAGTACCGTGAGGGAAAGATGAAA
AGCACTTTGAAAAGAGAGTTAAACAGTACGTGAAATTGTTGAAAGGGAAACGCTTGAAGTCAGT
CGCGTCCTGTGGTACTCAGCCTTGC----TTCGGCTCGGTGTACTTACCAT-TT--
GGACGGGTCAACGTCAA-TTTC-GAC-
CGGTGGACAAGGGCGAGGGGAATGTAGTGTCGTTTCGGCGGCGTG-TTATAGC-CCCTCGTC-
ACAT-ACACCGGCTGGGATTGAGGA-CTGCAGCACGCCC------TT-GTTGGCC-------
GGGGG----------------------TT---CG-CCCTACG-T-TTAGTGCTTAGGAT-
GTTGGCATAATGGCTTTAAGCGACCCGTCTTGAAACACGGACCAAGGAGTCTAACATGCTTGCG
AGTGTTTGGGTGGAAAACCCTTGCGCGGAATGAAAGTGAAAGGTTGGGAG-----CCTC------
------------CGCGA--GGGGGTGCACTGACGCCCGGCCCTGACG-----
TTCTCTGATGGTGCTGCGGTAGAGCAAGTATGTTGGG----
ACCCGAAAGATGGTGAACTATGCCTGAATAGGGCGAAGCCAGAGGAAACTCTG-
GTGGAGGCTCGTAGCGATTCTGACGTGCAAATCGATCGTCAAATTTGGG------------------
----

>Phylloporia_dependens_Dai_13167

TCCCCTAGTAACTGCGAGTGAAGCGGGAAGAGCTCAAATTTGTAATCT-G-GCG-GTC--
TTGGGCCGTCCGAGTTGTAATCTGAAGAGGCATTGT-CCGCGTCG-
GACCGTGTACAAGTCTCCTGGAATGGAGCGTCATAGAGGGTGAGAATCCCG-
TCCATGACACGG-AC-CGCCGATGC-
TTTGTGAGGTGCCCTCGAAGAGTCGCGTTGTTTGGGAATGCAGCGCAAAATGGGTGGTAAATTC
CATCTAAAGCTAAATACTGGCGAGAGACCGATAGCGAACAAGTACCGTGAGGGAAAGATGAAA
AGCACTTTGAAAAGAGAGTTAAACAGTACGTGAAATTGTTGAAAGGGAAACGCTTGAAGTCAGT
CGCGTCCTGTGGTACTCAGCCTTGC----TTCGGCTCGGTGTACTTACCAT-TT--
GGATGGGTCAACGTCAA-TTTC-GAC-
CGGTGGACAAGGGCGAGGGGAATGTAGCGTCGTTTCGGCGGCGTG-TTATAGC-CTCTTGTC-
GTAT-ACACTGGCTGGGATTGAGGA-CCGCAGCACGCCC------TT-G-TGGCC-------
GGGGG----------------------TT---CG-CCCTACG-T-TCTGTGCTTAGGAT-
GTTGGCATAATGGCTTTAAGCGACCCGTCTTGAAACACGGACCAAGGAGTCTAACATGCTTGCG
AGTGTTTGGGTGGAAAACCCTTGCGCGTAATGAAAGTGAAAGGTTGGGAA-----CCTC-------
-----------CGCGA--GGGGGTGCACCGACGCCCGGCCCTGACG-----
TTCTCTGACGGTGCTGCGGTAGAGCAAGTATGTTGGG----
ACCCGAAAGATGGTGAACTATGCCTGAATAGGGTGAAGCCAGAGGAAACTCTG-
GTGGAGGCTCGTAGCGATTCTGACGTGCAAATCGATCGTCAAATTTGGG------------------
----

>Phylloporia_flacourtiae_Yuan_6360

TCCCCTAGTAACTGCGAGTGAAGCGGGAAGAGCTCAAATTTGTAATCT-G-GCG-GTC--
TCTGGCCGTCCGAGTTGTAATCTGCAGAGGTGTCGT-CTGCGTCG-
GCCCGTGTACAAGTCTCCTGGAATGGAGCGTCATAGAGGGTGAGAATCCCG-
TCCATGACACGG-AT-CACCGAGGC-
TCTGTGAGGCGCCCTCGAAGAGTCGCGTTGTTTGGGAATGCAGCGCAAAATGGGTGGTAAATTC
CATCTAAGGCTAAATATTGGCGAGAGACCGATAGTGAACAAGTACCGTGAGGGAAAGATGAAA
AGCACTTTGAAAAGAGAGTTAAACAGTACGTGAAATCGTTGAAAGGGAAACGCTTGAAGTCAG
TCGCGTCTCGAGGCACTCAGCCTCGC----TTCGGCTTGGTGTACTTGCCTC-G---
GGACAGGTCAACATCAA-TTTT-GGC-
CGATGGAGAAGGGTGAGAGGAATGTGGCGTCATTTCGGTGACGTG-TTATAGC-CTCTTGTC-
GAAT-GCGTCGGCTGGGATTGAGGA-CTGCAGCACGCCC------TT-G-TGGCC-------
AGAGG----------------------TT---CG-CCTCATG-T-AACGTGCTTAGGAT-
GTTGGCATAATGGCTTTAAGCGACCCGTCTTGAAACACGGACCAAGGAGTCTAACACGCTCGCA
AGTGTTCAGGTGGAAAACCTTTGCGCATAATGAAAGTGAAA-GTTGGGAG-----CCTC-------
-----------CGTGA--GGGGGTGCACCGACGCCCGGCCCTGACG-----
TTCTCTGATGGTGCCGCGGTAGAGCGAGCGTGTTGGG----
ACCCGAAAGATGGTGAACTATGCCTGAATAGGGCGAAGCCAGAGGAAACTCTG-
GTGGAGGCTCGTAGCGATTCTGACGTGCAAATCGATCGTCAAATTTGGG------------------
----

>Phylloporia_flacourtiae_Yuan_6362

TCCCCTAGTAACTGCGAGTGAAGCGGGAAGAGCTCAAATTTGTAATCT-G-GCG-GTC--
TCTGGCCGTCCGAGTTGTAATCTGCAGAGGTGTCGT-CTGCGTCG-
GCCCGTGTACAAGTCTCCTGGAATGGAGCGTCATAGAGGGTGAGAATCCCG-
TCCATGACACGG-AT-CACCGAGGC-
TCTGTGAGGCGCCCTCGAAGAGTCGCGTTGTTTGGGAATGCAGCGCAAAATGGGTGGTAAATTC
CATCTAAGGCTAAATATTGGCGAGAGACCGATAGTGAACAAGTACCGTGAGGGAAAGATGAAA
AGCACTTTGAAAAGAGAGTTAAACAGTACGTGAAATCGTTGAAAGGGAAACGCTTGAAGTCAG
TCGCGTCTCGAGGCACTCAGCCTCGC----TTCGGCTTGGTGTACTTGCCTC-G---
GGACAGGTCAACATCAA-TTTT-GGC-
CGATGGAGAAGGGTGAGAGGAATGTGGCGTCATTTCGGTGACGTG-TTATAGC-CTCTTGTC-
GAAT-GCGTCGGCTGGGATTGAGGA-CTGCAGCACGCCC------TT-G-TGGCC-------
AGAGG----------------------TT---CG-CCTCATG-T-AACGTGCTTAGGAT-
GTTGGCATAATGGCTTTAAGCGACCCGTCTTGAAACACGGACCAAGGAGTCTAACACGCTCGCA
AGTGTTCAGGTGGAAAACCTTTGCGCATAATGAAAGTGAAA-GTTGGGAG-----CCTC-------
-----------CGTGA--GGGGGTGCACCGACGCCCGGCCCTGACG-----
TTCTCTGATGGTGCCGCGGTAGAGCGAGCGTGTTGGG----
ACCCGAAAGATGGTGAACTATGCCTGAATAGGGCGAAGCCAGAGGAAACTCTG-
GTGGAGGCTCGTAGCGATTCTGACGTGCAAATCGATCGTCAAATTTGGG------------------
----

>Phylloporia_fontanesiae_Cui12356
TTGGAGTTTTG-----A-A---GCGAGG------------------------------CCTGCTGCTG--
GCGTG---------------GCAACA--CGCA-TGTG-CACGGC----------------------------
---TCC--GTG-----CTCAATCC--A--CTC-----------------AA-CCTC--TGTGCA-
CCTTTGCA---AAG------------------
------------------------------------CAAGTAGG----------------C------TACACTC-
---------------------------TTTGAA-------------GCATTTAGTAATGAGTAGGGTGATC-
--GTGC---------------------------------

---------------------------GTAAGCACC--------------------------GGTCGC-
CTGAAAGTGA---------------------------------AAGGGCT---TCATTGTTTGAA-------
----
-GGTGGTC-AGTAGTGAGTAA-GGTGA--AAGCGTGCGAGCGTAAGTA---------------
GCCTGAG-------AGTGAATG--------------------TCCTTCATTC------------AAAAG-
AGCGCGGTTGAAAGC-TTTGA------TCTTT----AT-------------CA---ACCTTTTAT-
ACGTTTTGTGAAT--G-----T-GTT-GC-T-CT-TC----GTGGGCAAAAT-------
TAATACAACTTTCAACAACGGATCTCTTGGCTCTCGCATCGATGAAGAACGCAGCGAAATGCGA
TAAGTAATGTGAATTGCAGAATTCAGTGAATCATCGAATCTTTGAACGCACCTTGCGCCCTTTGG
-TACTCCGAAGGGCATGCCTGTTTGAGTGTCATGTTAATCTCAAACCCT--------AAG-
CCCTTTG--------------TTG----GGCT-----CGG-ATGGTTTGGAC-TTGGAGG-C--------
TTTGCTGGC-ATGTG--------------CTTCT--GCGCACGTCGGCTCCTCTGAAAAGCATTAGC-
TGGACTTCGG---------------T-TT---GCG-TGG-----------ATGGTGTAATAGTAA-----
----------TGTG-TTCAC----CGGAAG-TGCTTG-CCTAA-TGAGTCCGC-TTC-----
TAATTGTCCT-----------------CGGAC-AA--------GG-TCC---TTTGT-GAC----C-
TTCGCTTGACCCTTTGACCTCATCCCCTAGTAACTGCGAGTGAAGCGGGAAGAGCTCAAATTTG
TAATCT-G-ACG-GTC--CCTGGCTGTCCGAGTTGTAATCTGTAGAGGCGTCGT-CTGCGTCG-
GCCCGTGTACAAGTCTCCTGGAATGGAGCGTCATAGAGGGTGAGAATCCCG-
TCCATGACACGG-AC-CACCGATGC-
TCTGTGAGGTGCCCTCGAAGAGTCGCGTTGTTTGGGAATGCAGCGCAAAATGGGTGGTAAATTC
CATCTAAGGCTAAATATTGGCGAGAGACCGATAGCGAACAAGTACCGTGAGGGAAAGATGAAA
AGCACTTTGAAAAGAGAGTTAAACAGTACGTGAAATTGTTGAAAGGGAAACGCTTGAAGTCAGT
CGTGTCTCGAGGCACTCAGCCTTGC----TTCGGCTTGGTGTACTTGCCTT-G---
GGATGGGTCAACATCAA-TTTT-GGT-
CGGTGGATAAGGATGAGGGGAATGTAGCGTTGTTTCGGCGACGTG-TTATAGC-CCCTTGTC-
GAAT-ACGTCGACTGGGATTGAGGA-CTGCAGCACGCCC------TT-G-TGGCC-------
GGGGG---------------------TT---CG-CCTCACG-T-AACGTGCTTAGGAT-
GTTGGCATAATGGCTTTAAGCGACCCGTCTTGAAACACGGACCAAGGAGTCTAACATGCTTGCG
AGTGTTCGGGTGGAAAACCCTTGCGCGTAATGAAAGTGAAA-GTTGGGAG-----CCTC-------
-----------TGTGA--GGGGGTGCACCGACGCCCGGCCCTGACG-----
TTCTCTGACGGTGCTGCGGTAGAGCAAGTATGTTGGG----
ACCCGAAAGATGGTGAACTATGCCTGAATAGGGCGAAGCCAGAGGAAACTCTG-
GTGGAGGCTCGTAGCGATTCTGACGTGCAAATCGATCGTCAAATTTGGG------------------
----

>Phylloporia_fontanesiae_LWZ_20201024-3
ATGAGTTTTGA-----A-A---GCGAGG------------------------------CCTGCTGCTG--
GCGTG---------------GCAACG--TGCA-TGTG-CACGGC----------------------------
---TCC--GTG-----CTCAATCC--A--CTC-----------------AA-CCTC--TGTGCA-
CCTTTGCA--AAG-------------------
------------------------------------CAAGTAGG----------------C------TACACTC-
---------------------------TTTGAA-------------GCATTTAGTAATGAGTAGGGTGATC-
---GTGC---------------------------------

---------------------------GTAAGCACC--------------------------GGTCGC-
CTGAAAGTGA---------------------------------AAGGGCT---TCATTGTTTGAA-------
----
-GGTGGTC-AGTAGTGAGTAA-GGTGA--AAGCGTGCAAGCGTAAGTA---------------
GCCTGAG-------AGCGAATG---------------------TCCTTCATTC------------AAAAG-
AGCGCGGTTGAAAGC-TTTGA------TCTTT----AT-------------CA---ACCTTTTAT-
ACGTTTTGTGAAT--G-----T-GTT-GC-T-CT-TC----GCGGGCTTAAT-------
TAATACAACTTTCAACAACGGATCTCTTGGCTCTCGCATTGATGAAGAACGCAGCGAAATGCGA
TAAGTAATGTGAATTGCAGAATTCAGTGAATCATCGAATCTTTGAACGCACCTTGCGCCCTTTGG
-TACTCCGAAGGGCATGCCTGTTTGAGTGTCATGTTAATCTCAAACCCT--------AAG-
CCCTTTG--------------TTG----GGCT-----CGG-ATGGTTTGGAC-TTGGAGG-C--------

TTTGCTGGC-ATGTG--------------CTTCT--GCGCACGTCGGCTCCTCTGAAAAGCATTAGC-
TGGACTTCGG----------------T-TT---GCG-TGG-----------ATGGTGTAATAGTAA-----
----------TGTG-TTCAC----CGGAAG-TGCTTG-CCTAA-TGAGTCCGC-TTC-----
TAATTGTCCT------------------CGGAC-AA--------GG-TCC---TTTGT-GAC----C-
TTCGCTTGACCCTTTGACCTCATCCCCTAGTAACTGCGAGTGAAGCGGGAAGAGCTCAAATTTG
TAATCT-G-ACG-GTC--CCTGGCTGTCCGAGTTGTAATCTGTAGAGGCGTCGT-CTGCGTCG-
GCCCGTGTACAAGTCTCCTGGAATGGAGCGTCATAGAGGGTGAGAATCCCG-
TCCATGACACGG-AC-CACCGATGC-
TCTGTGAGGTGCCCTCGAAGAGTCGCGTTGTTTGGGAATGCAGCGCAAAATGGGTGGTAAATTC
CATCTAAGGCTAAATATTGGCGAGAGACCGATAGCGAACAAGTACCGTGAGGGAAAGATGAAA
AGCACTTTGAAAAGAGAGTTAAACAGTACGTGAAATTGTTGAAAGGGAAACGCTTGAAGTCAGT
CGTGTCTCGAGGCACTCAGCCTTGC----TTCGGCTTGGTGTACTTGCCTT-G---
GGATGGGTCAACATCAA-TTTT-GGT-
CGGTGGATAAGGATGAGGGGAATGTAGCGTTGTTTCGGCGACGTG-TTATAGC-CCCTTGTC-
GAAT-ACGTCGACTGGGATTGAGGA-CTGCAGCACGCCC------TT-G-TGGCC-------
GGGGG----------------------TT---CG-CCTCACG-T-AACGTGCTTAGGAT-
GTTGGCATAATGGCTTTAAGCGACCCGTCTTGAAACACGGACCAAGGAGTCTAACATGCTTGCG
AGTGTTCGGGTGGAAAACCCTTGCGCGTAATGAAAGTGAAA-GTTGGGAG-----CCTC-------
-----------TGTGA--GGGGGTGCACCGACGCCTGGCCCTGACG-----
TTCTCTGACGGTGCTGCGGTAGAGCAAGTATGTTGGG----
ACCCGAAAGATGGTGAACTATGCCTGAATAGGGCGAAGCCAGAGGAAACTCTG-
GTGGAGGCTCGTAGCGATTCTGACGTGCAAATCGATCGTCAAATTTGGG------------------
----

>Phylloporia_gutta_Dai_16070
CTTAGAGATGT-----A-A---GCGAGG-----------------------------TTTGCTGCTG--
GCGTG---------------G-AACG--CGCA-CGTG-CACGGC----------------------------
---TTC--GTG-----CTCAATCC--A--TTC-----------------AA-CCCC--TGTGCA-
CCTTTGCA---AAG------------------
-----------------------------------CAAGTAGG----------------C------TTTGTTC-
--------------------------TTTGAA-------------GTCTTTAGTAATGAGTAAGGCGAGC-
--GTGC--------------------------------

--------------------------GTGAGCATC-------------------------AGTAGT-
CCGAAAGTGA--------------------------------ACGGACT---TCATT---------------
----
TACCTTTTGAAATGCTC-AGCG----GCAA-GGTGA--AAGTGCGCAAGCGCAAGTA----------
-----GTCTGAA-------AGTGGGGG--------------------CGTTTCACTT-----------
GGAG---AGTGAGGCCGAAAGC-TTTGT------TCTTT----AT-------------CA---
ACCCTTTGT-ATGTCTTGTGAAT--G-----C-ATT-GC-T-CC-TT----GTGGGCGAAAT-------
AAATACAACTTTCAACAACGGATCTCTTGGCTCTCGCATCGATGAAGAACGCAGCGAAATGCGA
TAAGTAATGTGAATTGCAGAATTCAGTGAATCATCGAATCTTTGAACGCACCTTGCGCCCTTTGG
-TATTCCGAAGGGCATGCCTGTTTGAGTGTCATGTTAATCTCAAACCTC--------GAG-
CCTTTTG--------------TTA----GGCT-----CGA-ACGGTTTGGAC-TTGGAGG-C--------
TTTGCTGGT-GAGCG--------------CCTCT--
GCATGCGCCGGCTCCTCTAAAAGGCATTAGC-TGGACTTTGG----------------T-TC---
GCA-TGA-----------ATGGTGTGATAGTAA---------------TGTG-TTCAC----CGGAAG-
TGCTTG-TCTGA-TGGGTCCGC-TTC-----TAATCGTCTT------------------CGGAC-AA----
----GGTTCC---TTTGC-GAC----C-
TTTGCTTGACCGTTTGACCTCATCCCCTAGTAACTGCGAGTGAAGCGGGAAGAGCTCAAATTTGT
AATCT-G-GCG-GTC--TCTGGCCGTCCGAGTTGTAATCTGTAGAGGCGTCGT-CCGCGTCG-
GCCCGTGTACAAGTCTCCTGGAATGGAGCGTCATAGAGGGTGAGAATCCCG-
TCCATGACACGG-AT-CACCGATGC-
TCTGTGAGGTGCCCTCGAAGAGTCGCGTTGTTTGGGAATGCAGCGCAAAATGGGTGGTAAATTC
CATCTAAGGCTAAATATTGGCGAGAGACCGATAGCGAACAAGTACCGTGAGGGAAAGATGAAA
AGCACTTTGAAAAGAGAGTTAAACAGTACGTGAAATTGTTGAAAGGGAAACGCTTGAAGTCAGT
CGCGTCTCGAGGCACTCAGCCTTGC----TTCGGCTTGGTGTACTTGCCTT-G---
GGATGGGTCAACATCGA-TTTT-GGT-
TGGTGGACAAGGGTGAGGGGAATGTAGCGTCGTTTCGGTGACGTG-TTATAGC-CTCTTGTC-
GAAT-ACGCCAGCTGGGATTGAGGA-CCGCAGCACGCCC------TT-G-TGGCC-------
GAGGG----------------------TT---CG-CCCTACG-T-AACGTGCTTAGGAT-
GTTGGCATAATGGCTTTAAGCGACCCGTCTTGAAACACGGACCAAGGAGTCTAACATGCTTGCG
AGTGTTCGGGTGGAAAACCCTTGCGCATAATGAAAGTGAAA-GTTGGGAG-----CCTT-------
-----------CGTA---GGGGGTGCACTGACGCCCGGCCCTGACG-----

TTCTCTGATGGTGCTGCGGTAGAGCAAGTATGTTGGG----
ACCCGAAAGATGGTGAACTATGCCTGAATAGGGTGAAGCCAGAGGAAACTCTG-
GTGGAGGCTCGTAGCGATTCTGACGTGCAAATCGATCGTCAAATTTGGGTTGATGTGAC--AT--
-CGAGGATAACGTAAGGGAAAAT-
AATGATATTACCTTACTATTAGTGTCGTCCAAATGCTCGTGCCAGAAGACTCGGTAAGGCGAGA
GACGCAAACGTTAGTCGTCTTGATCAGGCGTAAAGGGTTTGTAGGCAGCTTTAAGTATCCAA-
TTTAATGTTTACTATTAT--------------------------------AATATTTACA---------------
----

AGTTATAATATTTATTAGAGGATCAATTAAAGCTAGAATCTAGAAGAGGTGA----
ATACAATGCTTGAA-CTTAGGAGTACTAACCGCAAACATCTAGTAGAGTGC-
TAAGGGCGAAGGC-
AGTTTTCCAATACAGATTGACGCTGAGAAACGAAGGTGAGGATAGAAAAAAGGATTAGAGACC
CTTGTATCCCTCACTGTCAACGATGAATGGTAGTTACTAGGGAGAA----------------------
----
ATGACCTAGAGGCGATGTTAACACGTAAACCATTCCGCCTTGTGAGTACGACTGCAAAGTTGAA
AACAAAAAAATTAGTCGGTTTCGGAG--CAAACGAAGTGAAGC-
ATGTTATTTAATGGGTACTGGTGAGTTCGAGGCTGGTATCTCGAAGGACGGTCAGACCCGCGAG
CATGCTCTGCTTGCGTTCACCCTCGGTGTGCGTCAGCTGATCGTTGCCGTGAACAAGATGGACA
CTGCTAAGGTTGGT-GGCGCTATTGA--CCTCT-CCGTGTCGAAGCTGAATA--GCGTTTG------
-
AACAATACAGTGGTCTGAGGAACGATTCAACGAAATTGTAAAGGAGACATCGAACTTCATCAA
GAAAGTCGGCTACAACCCCAAGGCCGTACCTTTCGTCCCGATCTCCGGTTGGCACGGTGACAA
CATGCTTGAGGAGTCGTCAAAGTATGTTTGATTT-TGCCC-----TACAATGTAAGCG-----
TATGCCGATGTGCCATCT-TTCAGTATGACTTGGTATAAGGGCTGGTCCAAGGAGACGAAGG---
---------GCGGTGTCGTT-
AAGGGAAAGACGCTTCTCGATGCCATTGATGCCATCGAGCCCCCTGTCCGCCCAGAGAACAAA
CCCTTGAGACTTCCTCTCCAGGATGTGATTTCCGTTGGATCATTATCCGCTCCGGTACTCGAATT
CTTGGAGGAATGGGGCTTGGAGAGCCTCGAGGAAAATGCACA---AGCTGCAA------
ATTTGACCAAAGTTTTCGTGAATGGGGTGTGGCTTGGAGTGCACCGAGACCCGCACACCCTTGT
CAACACTCTCCGAACGCTCAGGAGAAAGG---
AGGATCTCAACGCTGAGGTTTCCATTGTTCGTGACATACGCGAAAAGGAAGTTCGCATCTACAC
GGACGCAGGACGCGTTCTTCGACCGCTTTTCATTGTCGAGGACAATAGATTGAAGCTGAGACGG
CAGCACCTGCGCTGGCTACAGGCGAAGTGGAGGATT-------
GAGGAGAGGCCCAAAGACAGGATCCT------------
GGATGAAGGCGAAGAATCAGCAGGCGAGACAATTACTGAGCTAGAAGACGACGGAAAGCCAA
TAATACGGGAAGGGGTCCGAATGAAGAAGAAGAGGAAACCTGTCGCCTGGAAGACTTTGATTA
GCGACGGCGTGATTGAGTACCTTGATGCAGAAGAGGAGGAAACAGTTATGATCTGCATGACAC
CAGAAGATTTAGTCGAATCACGGTTAATGAGGCAAGGCCTGGATCCTCG---
AGCGGATACAGAGTTCGATGCTTCTACACGTCTCAAACCTGTACCGACT------
GCGCATAACTGGACACATTGCGAGATTCATCCCAGTATGATTCTGGGAATATGCGCCAGTATCA
TTCCGTTCCC
>Phylloporia_gutta_Cui_6945
ATGAGACTTGA-----A-A---GCGAGG------------------------------TTTGCTGCTG--

GCGTG---------------GAAACG--CGCA-CGTG-CACGGC----------------------------
---TTC--GTG-----CTCAATCC--A--TTC-----------------AA-CCCC--TGTGCA-
CCTTTGCA---AAG-------------------
-------------------------------------CAAGTAGG----------------C------TTTGTTC-
---------------------------TTTGAA-------------GTCTTTAGTAATGAGTAAGGCGAGC-
--GTGC----------------------------------

---------------------------GTGAGCATC---------------------------AGTAGT-
CCGAAAGTGA----------------------------------ACGGACT---TCATT----------------
----
TACCTTTTGAAATGCTC-AGCG----GCAA-GGTGA--AAGTGCGCAAGCGCAAGTA----------
-----GTCTGAA-------AGTGGGGG---------------------CGTTTCACTT-----------
GGAG---AGTGAGGCCGAAAGC-TTTGT------TCTTT----AT-------------CA---
ACCCTTTGT-ATGTCTTGTGAAT--G-----C-ATT-GC-T-CC-TT----GTGGGCGAAAT-------
AAATACAACTTTCAACAACGGATCTCTTGGCTCTCGCATCGATGAAGAACGCAGCGAAATGCGA
TAAGTAATGTGAATTGCAGAATTCAGTGAATCATCGAATCTTTGAACGCACCTTGCGCCCTTTGG
-TATTCCGAAGGGCATGCCTGTTTGAGTGTCATGTTAATCTCAAACCTC--------GAG-
CCTTTTG--------------TTA----GGCT-----CGA-ACGGTTTGGAC-TTGGAGG-C--------
TTTGCTGGT-GAGCG--------------CCTCT--
GCATGCGCCGGCTCCTCTAAAAGGCATTAGC-TGGACTTTGG----------------T-TC---
GCA-TGA-----------ATGGTGTGATAGTAA---------------TGTG-TTCAC----CGGAAG-
TGCTTG-TCTGA-TGGGTCCGC-TTC-----TAATCGTCTT------------------CGGAC-AA----
----GGTTCC---TTTGC-GAC----C-TTTGCTTGACCGTTTGACCTCA----------------------
----

TTGATGTGAC--AT---CGAGGATAACGTAAGGGAAAAT-
AATGATATTACCTTACTATTAGTGTCGTCCAAATGCTCGTGCCAGAAGACTCGGTAAGGCGAGA
GACGCAAACGTTAGTCGTCTTGATCAGGCGTAAAGGGTTTGTAGGCAGCTTTAAGTATCCAA-
TTTAATGTTTACTATTAT--------------------------------AATATTTACA---------------
----

AGTTATAATATTTATTAGAGGATCAATTAAAGCTAGAATCTAGAAGAGGTGA----
ATACAATGCTTGAA-CTTAGGAGTACTAACCGCAAACATCTAGTAGAGTGC-
TAAGGGCGAAGGC-

AGTTTTCCAATACAGATTGACGCTGAGAAACGAAGGTGAGGATAGAAAAAAGGATTAGAGACC
CTTGTATCCCTCACTGTCAACGATGAATGGTAGTTACTAGGGAGAA-----------------------
----
ATGACCTAGAGGCGATGTTAACACGTAAACCATTCCGCCTTGTGAGTACGACTGCAAAGTTGAA
AACAAAAAAAATTAGTCGGTTTCGGAG--CAAACGAAGTGAAGC-
ATGTTATTTAATTGGTACTGGTGAGTTCGAGGCTGGTATCTCGAAGGACGGTCAGACCCGCGAG
CATGCTCTGCTTGCGTTCACCCTCGGTGTGCGTCAGCTGATCGTTGCCGTGAACAAGATGGACA
CTGCTAAGGTTGGT-GGCGCTATTGA--CCTCT-CCGTGTCGAAGCTGAATA--GCGTTTG------
-
AACAATACAGTGGTCTGAGGAACGATTCAACGAAATTGTAAAGGAGACATCGAACTTCATCAA
GAAAGTCGGCTACAACCCCAAGGCCGTACCTTTCGTCCCGATCTCCGGTTGGCACGGTGACAA
CATGCTTGAGGAGTCGTCAAAGTATGTTTGATTT-TGCCC-----TACAATGTAAGCG-----
TATGCCGATGTGCCATCT-TTCAGTATGACTTGGTATAAGGGCTGGTCCAAGGAGACGAAGG---
---------GCGGTGTCGTT-
AAGGGAAAGACGCTTCTCGATGCCATTGATGCCATCGAGCCCCCTGTCCGCCCAGAGAACAAA
CCCTTGAGACTTCCTCTCCAGGATGTGATTTCCGTTGGATCATTATCCGCTCCGGTACTCGAATT
CTTGGAGGAATGGGGCTTGGAGAGCCTCGAGGAAAATGCACA---AGCTGCAA------
ATTTGACCAAAGTTTTCGTGAATGGGGTGTGGCTTGGAGTGCACCGAGACCCGCACACCCTTGT
CAACACTCTCCGAACGCTCAGGAGAAAGG---
AGGATCTCAACGCTGAGGTTTCCATTGTTCGTGACATACGCGAAAAGGAAGTTCGCATCTACAC
GGACGCAGGACGCGTTCTTCGACCGCTTTTCATTGTCGAGGACAATAGATTGAAGCTGAGACGG
CAGCACCTGCGCTGGCTACAGGCGAAGTGGAGGATT-------
GAGGAGAGGCCCAAAGACAGGATCCT------------
GGATGAAGGCGAAGAATCAGCAGGCGAGACAATTACTGAGCTAGAAGACGACGGAAAGCCAA
TAATACGGGAAGGGGTCCGAATGAAGAAGAAGAGGAAACCTGTCGCCTGGAAGACTTTGATTA
GCGACGGCGTGATTGAGTACCTTGATGCAGAAGAGGAGGAAACAGTTATGATCTGCATGACAC
CAGAAGATTTAGTCGAATCACGGTTAATGAGGCAAGGCCTGGATCCTCG---
AGCGGATACAGAGTTCGATGCTTCTACACGTCTCAAACCTGTACCGACT------
GCGCATAACTGGACACATTGCGAGATTCATCCCAGTATGATTCTGGGAATATGCGCCAGTATCA
TTCCGTTCCC
>Phylloporia_hainaniana_Dai_9460

TCCCCTAGTAACTGCGAGTGAAGCGGGAAGAGCTCAAATTTGTAATCT-G-GTG-GTC--
TTTGGCCACTCGAGTTGTAATCTGTAGAGGTGTTGT-CTGCGTCG-
GCCCGTGTACAAGTCTCCTGGAATGGAGCGTCATAGAGGGTGAGAATCCCG-
TCCATGATATGG-AC-CACCGATGC-
TTTGTGAGGCACCCTCGAAGAGTCGCGTTGTTTGGGAATGCAGCGCAAAATGGGTGGTAAATTC
CATCTAAAGCTAAATACTGGCGAGAGACCGATAGCGAACAAGTACCGTGAGGGAAAGATGAAA
AGCACTTTGAAAAGAGAGTTAAACAGTACGTGAAATTGTTGAAAGGGAAACGCTTGAAATCAGT
CGTGTCCTGGGGCACTCAACCTTGC----TTCGGCTTGGTGTACTTGCCTT-A---
GGATGGGTCAATATCAA-TTTT-GGC-
CGGTGGATAAGGATGAGAGGAAAGTAGCGTCATTTTGGTGACGTG-TTATAGC-CTCTTGTC-
AAAT-ACATCAGCTGAGATTGAGAA-TCGCAGCACGCCC------TT-G-TGGTC-------
GGAGG-----------------------TT---CG-CCTCATG-T-AACGTGCTTAGGAT-
GTTGACATAATGGTTTTAAGCGACCCGTCTTGAAACACGGACCAAGGAGTCTAACATGCTTGCG
AGTGTTTGGGTGGGAAACCCTTGTGCGTAATGAAAGTGAAA-GTTGGGAG-----CCTC-------
-----------CGTAA--GGGGGTGCACCGACGCCCGGCCCTGACG-----
TTCTCTGATGGCGCTGCGGTAGAGCAAGCATGTTGGG----
ACCCGAAAGATGGTGAACTATGCCTGAATAGGGCGAAGCCAGAGGAAACTCTG-
GTGGAGGCTCGTAGCGATTCTGACGTGCAAATCGATCGTCAAATTTGGG-------------------
----

>Phylloporia_hainaniana_Dai_16142

TCCCTTAGTAACTGCGAGTGAAGCGGGAAGAGCTCAAATTTGTAATCT-ACGTG-GTC--
TTTGGCCACTCGAGTTGTAATCTGTAGAGGTGTTGT-CTGCGTCG-
GCCCGTGTACAAGTCTCCTGGAATGGAGCGTCATAGAGGGTGAGAATCCCG-
TCCATGATATGG-AC-CACCGATGC-
TCTGTGAGGCACCCTCGAAGAGTCGCGTTGTTTGGGAATGCAGCGCAAAATGGGTGGTAAATTC
CATCTAAAGCTAAATACTGGCGAGAGACCGATAGCGAACAAGTACCGTGAGGGAAAGATGAAA
AGCACTTTGAAAAGAGAGTTAAACAGTACGTGAAATTGTTGAAAGGGAAACGCTTGAAATCAGT
CGCGTCCTGGGGCACTCAACCTTGC----TTCGGCTTGGTGTACTTGTCTT-A---
GGATGGGTCAACATCAA-TTTT-GGC-
CGGTGGATAAGGATGAGAGGAAAGTAGTGTCATTTCGGTGACGTG-TTATAGC-CTCTTGTC-
AAAT-ACATCAGCTGGGATTGAGAA-TTGCAGCACGCCC------TT-G-TGGTC-------
GGAGG----------------------TT---CG-CCCTACG-T-AACGTGCTTAGGAT-
GTTGGCATAATGGTTTTAAGCGACCCGTCTTGAAACACGGACCAAGGAGTCTAACATGCTTGCG
AGTGTTTGGGTGGGAAACCCTTGCGCGTAATGAAAGTGAAA-GTTGGGAG-----CCTC-------

-----------CGTAA--GGGGGTGCACCGACGCCCGGCCCTGATG-----
TTCTCTGATGGCGCTGCGGTAGAGCAAGCATGTTGGG----
ACCCGAAAGATGGTGAACTATGCCTGAATAGGGCGAAGCCAGAGGAAACTCTG-
GTGGAGGCTCGTAGCGATTCTGACGTGCAAATCGATCGTCAAATTTGGGTTGATGTGAC--AT--
-CGAGGATAACGTAAGGGAAAAT-
AATGATATTACCTTACTATTAGTGTCGTCCAAATGCTCGTGCCAGAAGACTCGGTAAGGCGAGA
GACGCAAACGTTAGTCGTCTTGATCGGGCGTAAAGGGTTTGTAGGCAGCTTTAAGTATTCAA-
TTTAATGTTTACTATTAT-------------------------------AATAATTACA--------------
----

AGTTATAATATTTATTAGAGGATCAATTAAAGCTAGAATCTAGAAGAGGTGA----
ATATAATGCTTGAG-CTTAGGAGTACTAACCGCAAACATCAAGTAGAGTGC-
TAAGGGCGAAGGC-
AGTTTTCCAATGAAGATTGACGCTGAGAAACGAAGGTGAGGATAGAAAAAAGGATTAGAGACC
CTTGTATCCCTCACTGTAAACGATGAATGGTAGTTACTAGGGAGAA----------------------
----
ATGACCTAGAGGCGATGTTAACACGTAAACCATTCCGCCTTGTGAGTACGACTGCAAAGTTGAA
AACAAAAAAATTAGTCGGTTTCGGAG--CAAACGAAGTGAAGC-
ATGTTATTTAATTGGTACTGGTGAGTTCGAGGCTGGTATCTCGAAGGACGGTCAGACCCGCGAG
CATGCTCTGCTTGCGTTCACCCTCGGTGTGCGCCAGCTGATCGTCGCCGTGAACAAAATGGACA
CGGCCAAGGTTGGTGGATGCTATTGA--CCTTCGCCGTACCGAAGCTGAACC--CC-TTCG-----
--
AATAATTCAGTGGTCCGAGGAACGATTTAACGAAATCGTCAAGGAGACGTCGAACTTCATTAAG
AAAGTCGGCTACAACGCGAAGGCCGTACCTTTCGTCCCGATCTCCGGATGGCACGGTGACAAT
ATGCTTGAGGAGTCGATCAAGTATGTTTGATTT-TGTCCTGCTTTACAGGATAAGAG-----
TGATCTGACGTGATATCT-CCCAGCATGTCTTGGTACAAGGGCTGGTCCAAGGAGACGAAGG--
----------GCGGTGTCGTT-
AAAGGAAAGACACTTCTCGATGCCATTGATGCCATTGAACCCCCTGTCCGTCCGGAGAACAAG
CCCTTGCGACTCCCTCTGCAGGACGTG--

>Phylloporia_homocarnica_Yuan_5750
ATGAGCTTTGA-----A-A---GCAGAG-----------------------------TCTGTTGCTG--
GCATT---------------GAAATG--TGCA-CGTG-CAAGGC---------------------------
---TCT--GTG-----CTCAATTC--A--CTC-----------------AA-CCTT--
TGTGCACCCTTTGCA---AAG-----------

---TGGAGTAGG----------------C------
TGCAC-------------------------------TTGAAGG-----------
ACTTAAAGTAACTGAGGAGGCAAGC--
CTGC---
--GCAAGCATT---
-----------------------AGTAGC-CTTAAAGTAA---------------------------------
AAGGGCT---TCATTGTTGAAA--GTGGTGAGAAGCGAGTAGGG-TGTGTAAGCATGC--------
---TAAGAAATTGT---------
-----ATGCTGGTG---------------ACCAGAA-------AGTGGAACA-------------------
CCCTTTCATTT-----------AA-----AGTGCAGTTGAAAGC-TTTGC------TTTTT-----T-----
--------TA---ACCTTTTGC-ATGTTTTGTAAAT--G-----T-GTT-GC-C-CC-TT----
ATGAGGGAAAT-------
AAATACAACTTTCAACAACGGATCTCTAGGCTCTCGCATCGATGAAGAACGCAGCGAAATGCG
ATAAGTAATGTGAATTGCAGAATTCAGTGAATCATCGAATCTTTGAACGCATCTTGCGCCCTCTG
G-TATTCCAGAGGGCATGCCTGTTTGAGTGTCGTATTAAAATCAAACCCT--------CAC-
ATCTCTG--------------TTA----GGTGTGG-ATGG-ATGGTTTGGACTTTGGAGG-T-------
TTTGCTGCT-GTGTGCTG--------CTCTTTTT--
CTGCATCTCGGCTCCTCTAAAAAGCATTAGCTTAGGCTTTAG----------------C-CT---GCA-
TAG-----------ATGGTGTAATAATAA---------------TGGA-TTCAC----CAGAAG-
TGCTTG-TCTGA-TAGGTCTGC-TTCTAATTTAATCCTTTC------------------TGCTC-AA----
----GG-TCC---TTTGT-GAC----C-
TTTGCTTGACCACTTGACCTCATCCCCTAGTAACTGCGAGTGAAGCGGGAAGAGCTCAAATTTG
TAATCT-A-ATG-GCC--TCTGGCTGTTCGAGTTGTAATCTGTAGAGGCATCGT-
CCGCATCATGGCCATGTCTAAGTCTCCTGGAATGGAGCTTCATAGAGGGTGAGAATCCCG-
TATATGATATGG-AC-CAGTGTTGC-
TCTGTGAGATGCCCTCAAAGAGTCGCGTTGTTTGGGAATGCAGCGCAAAATGGGTGGTAAATTC
CATCTAAGGCTAAATATTGGCGAGAGACCGATAGTGAACAAGTACCGTGAGGGAAAGATGAAA
AGCACTTTGAAAAGAGAGTTAAATAGTACGTGAAATTGTTGAAAGGGAAACGCTTGGAGTCAG
CCATGTTCTGAGGCACTCAGCCTTGC----TTTGGCTTGGTGTATTTGTCTT-A---
GGATGGGTCAACATCAA-TTTT-GGC-
CAGTAGATAAAGGCAAGGGGAATGTGGCATTGCTTTAGTGATGTG-TTATAGC-TCTTTGTC-
AAAT-GTACTGGCTGGGATTGAGGAATTGCAGCATGCCC------TT-G-TGGCT-------
GGAGA----------------------TT---CG-TCTCACG-A-AACATGCTTAAGAT-
GTTGGCGTAATGGCTTTAAGCGACCCGTCTTGAAACACGGACCAAGGAGTCTAACATGCTTGCA
AGTGTTCGGGTGGAAAACCCTTGCGCAGAATGAAAGTGAAA-GTTGGGAG-----CCTT-------
-----------TATAA---GAGGTGCACCGACGCCTGGCCCTGATG-----
TTCTCTGATGGTGCTGCGGTAGAGCAAGTATGTTGGG----
ACCCGAAAGATGGTGAACTATGCCTGAATAGGGTGAAGCCAGAGGAAACTCTG-
GTGGAGGCTCGTAGCGATTCTGACGTGCAAATCGATCGTCAAATTTGGG------------------
----

>Phylloporia_homocarnica_Yuan_5766

TCCCCTAGTAACTGCGAGTGAAGCGGGAAGAGCTCAAATTTGTAATCT-A-ATG-GCC--

TCTGGCTGTTCGAGTTGTAATCTGTAGAGGCATCGT-
CCGCATCATGGCCATGTCTAAGTCTCCTGGAATGGAGCTTCATAGAGGGTGAGAATCCCG-
TATATGATATGG-AC-CAGTGTTGC-
TCTGTGAGATGCCCTCAAAGAGTCGCGTTGTTTGGGAATGCAGCGCAAAATGGGTGGTAAATTC
CATCTAAGGCTAAATATTGGCGAGAGACCGATAGTGAACAAGTACCGTGAGGGAAAGATGAAA
AGCACTTTGAAAAGAGAGTTAAATAGTACGTGAAATTGTTGAAAGGGAAACGCTTGGAGTCAG
CCATGTTCTGAGGCACTCAGCCTTGC----TTTGGCTTGGTGTATTTGTCTT-A---
GGATGGGTCAACATCAA-TTTT-GGC-
CAGTAGATAAAGGCAAGGGGAATGTGGCATTGCTTTAGTGATGTG-TTATAGC-TCTTTGTC-
AAAT-GTACTGGCTGGGATTGAGGAATTGCAGCATGCCC------TT-G-TGGCT-------
GGAGA----------------------TT---CG-TCTCACG-A-AACATGCTTAAGAT-
GTTGGCGTAATGGCTTTAAGCGACCCGTCTTGAAACACGGACCAAGGAGTCTAACATGCTTGCA
AGTGTTCGGGTGGAAAACCCTTGCGCAGAATGAAAGTGAAA-GTTGGGAG-----CCTT-------
-----------TATAA---GAGGTGCACCGACGCCTGGCCCTGATG-----
TTCTCTGATGGTGCTGCGGTAGAGCAAGTATGTTGGG----
ACCCGAAAGATGGTGAACTATGCCTGAATAGGGTGAAGCCAGAGGAAACTCTG-
GTGGAGGCTCGTAGCGATTCTGACGTGCAAATCGATCGTCAAATTTGGG------------------
----

>Phylloporia_lespedezae_Dai_17067

ATGAGTTTTGA-------A---GTGGAG------------------------------
TCTGTTGCTGGTGTGTG---------------GAAACA--CGCA-TGTG-CACGGC--------------
-----------------TCT--GTG-----CTCAATCC--A--CTT-----------------AA-CTCT--
TGTGCA-CCTTTGCA--AAG-----------
--TGAGTAGGC--------------TC------
TTCACCC----------------------------TTTGAA-------------
GTCACTAGTAGTGAGTAAGGTGAGT--GTGC-

---GTGA---------------

---GCACC---A-------------
--GCCTGAA-------TGCGAGTG---------------------TCTCTCATTT-----------
AGAGGG-AGTGTGGTCGAAAGC-TTTGA------TCTTT----AT-------------CA---
ACTCTTTGT-ATGTCATGTGAAT--G-----T-GTT-GC-T-AC-TT----GTGAGCGAAT-------
AAATACAACTTTCAACAATGGATCTCTTGGCTCTCGCATCGATGAAGAACGCAGCGAAATGCGA
TAAGTAATGTGAATTGCAGAATTCAGTGAATCATCGAATCTTTGAACGCACCTTGCGCCCTTTGG
-TATTCCGAAGGGCATGCCTGTTTGAGTGTCATGTTAATCTCAAACCTC--------AAG-
CCTCTTG--------------TTA----GGTT-----TGG-ATGGTTTGGAC-TTGGAGG-C--------
TTTGCTGGT-GTGTG---

TCTCTTAGTAACTGCGAGTGAAGGGGGAAGAGCTCAAATTTGTAATCT-G-GTG-GTC--
TATGGCCATCCGAGTTGTAATCTGTAGAGGCATCAT-CCGTGTCG-
GCCCATGTATAAGTCTCCTGGGATGGAGTGTCATAGAGGGTGAGAATCCCG-
TCCATGACATGG-AT-CACCGATGC-
TTTGTGAGGTGCCCTCGAAGAGTCGTGTTGTTTGGGAATGCAGCGCAAATTGGGTGGTAAATTC
CATCTAAGGCTAAATATTGGCGAGAGACCGATAGCGAACAAGTACCGTGAGGGAAAGATGAAA
AGCACTTTGAAAAGAGAGTTAAACAGTATGTGAAATTGTTGAAAGGGAAACACTTGAAGTCAGT
CGTGTTCTGTGGCACTCAGCCTTGC----TTTGGCTTGGTGTACTTGCCTT-G---
GGATGGGTCAACATTAA-TTTC-GGC-
CGGTGGACAAGGGTGAGGGGAACGTAGTGTTGTTTTGGCAACGTA-TTATAGC-CTCTTGTT-
AAAT-ACACCGGCTGGGATTGAGGA-TCGCAGCACACCT------TT-G-TGGAC-------
AGGGG----------------------TT---CA-CCTCATG-T-AATGTGCTTAGGAT-
GTTGGCATAATGGCTTTAAGTGACCCGTCTTGAAACACGGACCAAGGAGTCTAACATGCTTGCG
AGTGTTTGGGTGGAAAACCCTTGTGCGTAATGAAAGTGAAA-GTTGGGAG-----CCTC-------
-----------TGTAA--GGGGGTGCACCGATGCCTGGCCCTGATG-----
TTCTCTGACGGTGCTGCGGTAGAGCAAGTATGTTGGG----
ACCCGAAAGATGGTGAACTATGCCTGAATAGGGTGAAGCCAGAGGAAACTCTG-
GTGGAGGCTCGTAGCGATTCTGACGTGCAAATCGATCGTCAAATTTGGGTAGATGTGTC--
ATATGATGGCAGCACGTAAGGGAAAAT-
AATGATATTACCTTACTATTAGTGTCGTCCAAATGCTCGTGCCAGAAGACTCGGTAAGGCGAGA
GACGCAAACGTTAGTCGTCTTGATCAGGCGTAAAGGGTTTGTAGGCAGCTTTAAGTATCCAA-

TTTAATGTTTACTATTAT-------------------------------AATATTTACA--------------
----

TGTTATAATATTTATTAAAGGATCAATTAAAGCTAGAATCTAGAAGAGGTGA----
ATACAATGCTTGAG-CTTAGGAGTACTAACCGCAAACATCAAGTAGAGTGC-
TAAGGGCGAAGGC-
AGTTTTCCAATAAAGATTGACGCTGATAAACGAAGGTGAGGATAGAAAAAAGGATTAGAGACC
CTTGTATCCCTCACTGTCAACGATGAATGGTAGTTACTAGGGAGAA----------------------
----
ATGACCTAGAGGCGATGTTAACACGTAAACCATTCCGCCTTGTGAGTACGACTGCAAAGTTGAA
AACAAAAAAATTAGTCGGTTTCGGAG--CAAACGAAGTGAAGC-
ATGTTATTTAATTGGTACTGGTGAGTTCGAGGCTGGTATCTCGAAGGACGGTCAGACCCGCGAG
CATGCTCTACTTGCGTTCACCCTCGGTGTGCGTCAGCTGATCGTTGCTGTGAACAAGATGGATAC
GGCTAAGGTTGGT-GGTGCCATTGA--CCCCT-CCGTAATGAAGCTGAATC--GCGTTTG------
-
AATACTCCAGTGGTCTGAGGAACGATTCAACGAAATTGTTAAGGAGACATCGAACTTCATTAAG
AAAGTCGGCTACAACCCCAAGGCCGTACCTTTCGTCCCGATCTCCGGATGGCACGGTGACAAC
ATGCTTGAGGAGTCGAGCAAGTATGTTTGATTT-TGCCC-----TACAATGTAAGCG-----
TGTGCTGACGTGACATCT-TTCAGTATGACTTGGTACAAGGGCTGGACCAAGGAGACGAAGG--
----------GCGGTGTCGTT-
AAGGGAAAGACTCTTCTCGATGCCATTGATGCCATCGAGCCCCCTGTCCGCCCAGAGAACAAA
CCCTTGCGACTTCCTCTCCAGGATGTGATTTCCGTTGGATCATTATCCGCTCCGGTACTCGAATTT
TTGGAGGAATGGGGCTTGGAGAGCCTCGAGGAAAATGCACA---AGCTGCAA------
ATTTGACCAAAGTTTTCGTGAATGGGGTATGGCTCGGAGTACACCGAGACCCGCACACCCTTGT
CAACACTCTTCGAACGCTCAGGAGAAAGG---
AGGATCTCAACGCTGAGGTCTCCATTGTTCGTGACATACGCGAAAAGGAAGTTCGCATCTACAC
GGACGCAGGACGCGTTCTTCGACCGCTTTTCATTGTCGAGGACAATAGATTGAAGCTGAGACGT
CAGCACCTGCGCTGGCTGCAGGCGAAGTGGAGGATT-------
GAGGAGAGGCCCAAAGACAGGGTCCT------------
GGACGAAGGCGAAGAATCAGCAGGCGAGACGATAACTGAGCTAGAAGACGATGGGAAGCCA
ATAATACGGGAAGGCGTTCGAATGAAGAAGAAGAGGAAGCCGGTGACCTGGAAGACGTTGATT
AGCGACGGCGTTATTGAATACCTTGATGCAGAGGAGGAGGAAACGGTCATGATCTGCATGACA
CCAGAAGATCTGGTCGAATCTCGGTTAATGAGGCAGGGCTTGGATCCTCG---
AGCAGACACGGAGTTCGATGCGTCCACGCGTCTCAAGCCTGTACCAACT------
GCTCATAACTGGACACATTGCGAAATTCATCCCAGCATGATTCTAGGTATATGTGCAAGTATCAT
TCCGTTCCC
>Phylloporia_lespedezae_Dai_17065
ATGAGTTTTGA-----A-A---GTGGAG------------------------------
TCTGTTGCTGGTGTGTG---------------GAAACA--CGCA-TGTG-CACGGC-------------
-----------------TCT--GTG-----CTCAATCC--A--CTT-----------------AA-CTCT--
TGTGCA-CCTTTGCA--AAG----------
---TGAGTAGGC--------------TC------
TTCACCC----------------------------TTTGAA------------
GTCACTAGTAGTGAGTAAGGTGAGT---GTGC-

---GTGA---------------

---GCACC---A-------------
--GCCTGAA-------TGCGAGTG---------------------TCTCTCATTT-----------
AGAGGG-AGTGTGGTCGAAAGC-TTTGA------TCTTT----AT-------------CA---
ACTCTTTGT-ATGTCATGTGAAT--G-----T-GTT-GC-T-AC-TT----GTGAGCGAAAT-------
AAATACAACTTTCAACAATGGATCTCTTGGCTCTCGCATCGATGAAGAACGCAGCGAAATGCGA
TAAGTAATGTGAATTGCAGAATTCAGTGAATCATCGAATCTTTGAACGCACCTTGCGCCCTTTGG
-TATTCCGAAGGGCATGCCTGTTTGAGTGTCATGTTAATCTCAAACCTC--------AAG-
CCTCTTG--------------TTA----GGTT-----TGG-ATGGTTTGGAC-TTGGAGG-C--------
TTTGCTGGT-GTGT--

TCTCTTAGTAACTGCGAGTGAAGGGGGAAGAGCTCAAATTTGTAATCT-G-GTG-GTC--
TATGGCCATCCGAGTTGTAATCTGTAGAGGCATCAT-CCGTGTCG-
GCCCATGTATAAGTCTCCTGGGATGGAGTGTCATAGAGGGTGAGAATCCCG-
TCCATGACATGG-AT-CACCGATGC-
TTTGTGAGGTGCCCTCGAAGAGTCGTGTTGTTTGGGAATGCAGCGCAAATTGGGTGGTAAATTC
CATCTAAGGCTAAATATTGGCGAGAGACCGATAGCGAACAAGTACCGTGAGGGAAAGATGAAA
AGCACTTTGAAAAGAGAGTTAAACAGTATGTGAAATTGTTGAAAGGGAAACACTTGAAGTCAGT
CGTGTTCTGTGGCACTCAGCCTTGC----TTTGGCTTGGTGTACTTGCCTT-G---
GGATGGGTCAACATTAA-TTTC-GGC-
CGGTGGACAAGGGTGAGGGGAACGTAGTGTTGTTTTGGCAACGTA-TTATAGC-CTCTTGTT-
AAAT-ACACCGGCTGGGATTGAGGA-TCGCAGCACACCT------TT-G-TGGAC-------
AGGGG----------------------TT---CA-CCTCATG-T-AATGTGCTTAGGAT-
GTTGGCATAATGGCTTTAAGTGACCCGTCTTGAAACACGGACCAAGGAGTCTAACATGCTTGCG
AGTGTTTGGGTGGAAAACCCTTGTGCGTAATGAAAGTGAAA-GTTGGGAG-----CCTC-------
-----------TGTAA--GGGGGTGCACCGATGCCTGGCCCTGATG-----
TTCTCTGACGGTGCTGCGGTAGAGCAAGTATGTTGGG----
ACCCGAAAGATGGTGAACTATGCCTGAATAGGGTGAAGCCAGAGGAAACTCTG-
GTGGAGGCTCGTAGCGATTCTGACGTGCAAATCGATCGTCAAATTTGGGTTGATGTGAC--AT--
-CGAGGATAACGTAAGGGAAAAT-
AATGATATTACCTTACTATTAGTGTCGTCCAAATGCTCGTGCCAGAAGACTCGGTAAGGCGAGA
GACGCAAACGTTAGTCGTCTTGATCAGGCGTAAAGGGTTTGTAGGCAGCTTTAAGTATCCAA-
TTTAATGTTTACTATTAT--------------------------------AATATTTACA---------------
----

TGTTATAATATTTATTAAAGGATCAATTAAAGCTAGAATCTAGAAGAGGTGA----
ATACAATGCTTGAG-CTTAGGAGTACTAACCGCAAACATCAAGTAGAGTGC-
TAAGGGCGAAGGC-
AGTTTTCCAATAAAGATTGACGCTGATAAACGAAGGTGAGGATAGAAAAAAGGATTAGAGACC

CTTGTATCCCTCACTGTCAACGATGAATGGTAGTTACTAGGGAGAA----------------------
----
ATGACCTAGAGGCGATGTTAACACGTAAACCATTCCGCCTTGTGAGTACGACTGCAAAGTTGAA
AACAAAAAAATTAGTCGGTTTCGGAG--CAAACGAAGTGAAGC-
ATGTTATTTAATTGGTACTGGTGAGTTCGAGGCTGGTATCTCGAAGGACGGTCAGACCCGCGAG
CATGCTCTACTTGCGTTCACCCTCGGTGTGCGTCAGCTGATCGTTGCTGTGAACAAGATGGATAC
GGCTAAGGTTGGT-GGTGCCATTGA--CCCCT-CCGTAATGAAGCTGAATC--GCGTTTG------
-
AATACTCCAGTGGTCTGAGGAACGATTCAACGAAATTGTTAAGGAGACATCGAACTTCATTAAG
AAAGTCGGCTACAACCCCAAGGCCGTACCTTTCGTCCCGATCTCCGGATGGCACGGTGACAAC
ATGCTTGAGGAGTCGAGCAAGTATGTTTGATTT-TGCCC-----TACAATGTAAGCG-----
TGTGCTGACGTGACATCT-TTCAGTATGACTTGGTACAAGGGCTGGACCAAGGAGACGAAGG--
----------GCGGTGTCGTT-
AAGGGAAAGACTCTTCTCGATGCCATTGATGCCATCGAGCCCCCTGTCCGCCCAGAGAACAAA
CCCTTGCGACTTCCTCTCCAGGATGTGATTTCCGTTGGATCATTATCCGCTCCGGTACTCGAATTT
TTGGAGGAATGGGGCTTGGAGAGCCTCGAGGAAAATGCACA---AGCTGCAA------
ATTTGACCAAAGTTTTCGTGAATGGGGTATGGCTCGGAGTACACCGAGACCCGCACACCCTTGT
CAACACTCTTCGAACGCTCAGGAGAAAGG---
AGGATCTCAACGCTGAGGTCTCCATTGTTCGTGACATACGCGAAAAGGAAGTTCGCATCTACAC
GGACGCAGGACGCGTTCTTCGACCGCTTTTCATTGTCGAGGACAATAGATTGAAGCTGAGACGT
CAGCACCTGCGCTGGCTGCAGGCGAAGTGGAGGATT-------
GAGGAGAGGCCCAAAGACAGGGTCCT------------
GGACGAAGGCGAAGAATCAGCAGGCGAGACGATAACTGAGCTAGAAGACGATGGGAAGCCA
ATAATACGGGAAGGCGTTCGAATGAAGAAGAAGAGGAAGCCGGTGACCTGGAAGACGTTGATT
AGCGACGGCGTTATTGAATACCTTGATGCAGAGGAGGAGGAAACGGTCATGATCTGCATGACA
CCAGAAGATCTGGTCGAATCTCGGTTAATGAGGCAGGGCTTGGATCCTCG---
AGCAGACACGGAGTTCGATGCGTCCACGCGTCTCAAGCCTGTACCAACT------
GCTCATAACTGGACACATTGCGAAATTCATCCCAGCATGATTCTAGGTATATGTGCAAGTATCAT
TCCGTTCCC
>Phylloporia_lonicerae_LWZ_20190809-2
ATGAGTTTTTG----AA-A---GTGGAG------------------------------TCTGCTGCTG--
GTGTG---------------GAAACA--TGCA-TGTG-CACGGC----------------------------
---TCT--GTG-----CTCAATCC--G--CTC-----------------AA-CCCA--TGTGCA-
CCTTTGCA--AAG------------------
-----------------------------------CGAGTAGG----------------T------TTCACTT-
---------------------------TTTAGA-------------
CTGACAGGTAGTGAGTAAGGCGAGG---
GTGC---
---TATAG---------

--GCATCAGTA-------
--------GTCTGAA-------AGCGAGCG---------------------TCGTACATTT-----------
GAAG---AGTGTAGTCGAAAGC-CTTGA------TCTTT----AT-------------CA---

ACTTTTTGT-ATGTCTTGTGAAT--G-----T-GTT-GC-T-CT-GT----ATGGGCGAAAT-------
AAACACAACTTTCAACAACGGATCTCTTGGCTCTCGCATCGATGAAGAACGCAGCGAAATGCGA
TAAGTAATGTGAATTGCAGAATTCAGTGAATCATCGAATCTTTGAACGCACCTTGCGCCCTTTGG
-TATCCCGAAGGGCATGCCTGTTTGAGTGTCATGTTAATCTCAAACCTC--------CAG-
TCTTTTG--------------TTA----GGCTTGG-ATGG-ATGGTTTGGAC-TTGGAGG-C--------
TTTGCCGGT-AAGCGC-------------TCTCT--GCGTATGTCGGCTCCTCTGAAAAGCATTAGC-
TGGACCTTGG----------------T-TT---GCG-TGG-----------ATGGTGTAATAGTAA-----
----------TTTG-TTCAC----CGGAAG-GGCTTATCTTGA-CGGGTCTGC-TTC-----
TAATCGTCTT------------------CGGAC-AA--------GG-TC----ATTAC-GAC----C-
TTTGCTTGATCGTTTGACCTCATCCCTTAGTAACTGCGAGTGAAGCGGGAAGAGCTCAAATTTGT
AATCT-G-GCG-ATC--TCTGGTCGTCCGAGTTGTAATCTGTAGAGGCGTCGT-CCGCGTCG-
GCCCGTGTACAAGTCTCCTGGAATGGAGCGTCATAGAGGGTGAGAATCCCG-
TCCATGACACGG-AC-CACCGATGC-
TTTGTGAGGTGCCCTCGAAGAGTCGCGTTGTTTGGGAATGCAGCGCAAAATGGGTGGTAAATTC
CATCTAAGGCTAAATATTGGCGAGAGACCGATAGCGAACAAGTACCGTGAGGGAAAGATGAAA
AGCACTTTGGAAAGAGAGTTAAACAGTACGTGAAATTGTTGAAAGGGAAACGCTTGAAGTCAG
TCGCGTCCTGGGGCATTCAGCCTCGC----TTCGGCTTGGTGTACTTGCCTT-G---
GGATGGGTCAACATCAA-TTTT-GGC-
TGGTGGATAAGGGTGAGGGGAATGTAGCGTTGTTTCGGCGACGTG-TTATAGC-CCCTCATT-
GAAT-ACATCGGCTGGGATTGAGGA-CCGCAGCACGCCC------TT-G-TGGCC-------
GGGGG----------------------TT---CG-CCCTAC--T-CACGTGCTTAGGAT-
GTTGGCATAATGGCTTTAAGCGACCCGTCTTGAAACACGGACCAAGGAGTCTAACATGCTTGCG
AGTGTTCGGGTGGAAAACCCTTGCGCGTAATGAAAGTGAAA-GTTGGGAG-----CCTT-------
-----------CGCGA--GGAGGTGCACCGACGCCCGGCCCTGATG-----
TGCTCTGACGGTGCTGCGGTAGAGCAAGTATGTTGGG----
ACCCGAAAGATGGTGAACTATGCCTGAATAGGGTGAAGCCAGAGG-AACTCTG-
GTGGAGGCTCGTAGCGATTCTGACGTGCAAATCGATCGTCAAATTTGGG------------------
----

>Phylloporia_lonicerae_LWZ_20190819-1
ATGAGTTTTTG-----A-A---GTGGAG------------------------------TCTGCTGCTG--
GTGTG---------------GAAACA--TGCA-TGTG-CACGGC----------------------------
---TCT--GTG-----CTCAATCC--G--CTC-----------------AA-CCCA--TGTGCA-
CCTTTGCA---AAG------------------
----------------------------------CGAGTAGG----------------T------TTCACTT-
---------------------------TTTAGA-------------
CTGACAGGTAGTGAGTAAGGCGAGG---
GTGC--
---TATAG---------

--GCATCAGTA-------
--------GTCTGAA-------AGCGAGCG---------------------TCGTACATTT-----------
GAAG---AGTGTAGTCGAAAGC-CTTGA------TCTTT----AT-------------CA---
ACTTTTTGT-ATGTCTTGTGAAT--G-----T-GTT-GC-T-CT-GT----ATGGGCGAAAT-------
AAACACAACTTTCAACAACGGATCTCTTGGCTCTCGCATCGATGAAGAACGCAGCGAAATGCGA
TAAGTAATGTGAATTGCAGAATTCAGTGAATCATCGAATCTTTGAACGCACCTTGCGCCCTTTGG
-TATCCCGAAGGGCATGCCTGTTTGAGTGTCATGTTAATCTCAAACCTC--------CAG-
TCTTTTG--------------TTA----GGCTTGG-ATGG-ATGGTTTGGAC-TTGGAGG-C--------
TTTGCCGGT-AAGCGC-------------TCTCT--GCGTATGTCGGCTCCTCTGAAAAGCATTAGC-
TGGACCTTGG----------------T-TT---GCG-TGG-----------ATGGTGTAATAGTAA-----
----------TTTG-TTCAC----CGGAAG-GGCTTATCTTGA-CGGGTCTGC-TTC-----
TAATCGTCTT------------------CGGAC-AA--------GG-TC----ATTAC-GAC----C-
TTTGCTTGATCGTTTGACCTCATCCCTTAGTAACTGCGAGTGAAGCGGGAAGAGCTCAAATTTGT
AATCT-G-GCG-ATC--TCTGGTCGTCCGAGTTGTAATCTGTAGAGGCGTCGT-CCGCGTCG-
GCCCGTGTACAAGTCTCCTGGAATGGAGCGTCATAGAGGGTGAGAATCCCG-
TCCATGACACGG-AC-CACCGATGC-
TTTGTGAGGTGCCCTCGAAGAGTCGCGTTGTTTGGGAATGCAGCGCAAAATGGGTGGTAAATTC
CATCTAAGGCTAAATATTGGCGAGAGACCGATAGCGAACAAGTACCGTGAGGGAAAGATGAAA
AGCACTTTGGAAAGAGAGTTAAACAGTACGTGAAATTGTTGAAAGGGAAACGCTTGAAGTCAG
TCGCGTCCTGGGGCATTCAGCCTCGC----TTCGGCTTGGTGTACTTGCCTT-G---
GGATGGGTCAACATCAA-TTTT-GGC-
TGGTGGATAAGGGTGAGGGGAATGTAGCGTTGTTTCGGCGACGTG-TTATAGC-CCCTCATT-
GAAT-ACATCGGCTGGGATTGAGGA-CCGCAGCACGCCC------TT-G-TGGCC-------

GGGGG----------------------TT---CG-CCCTAC--T-CACGTGCTTAGGAT-
GTTGGCATAATGGCTTTAAGCGACCCGTCTTGAAACACGGACCAAGGAGTCTAACATGCTTGCG
AGTGTTCGGGTGGAAAACCCTTGCGCGTAATGAAAGTGAAA-GTTGGGAG-----CCTT-------
-----------CGCGA--GGAGGTGCACCGACGCCCGGCCCTGATG-----
TGCTCTGACGGTGCTGCGGTAGAGCAAGTATGTTGGG----
ACCCGAAAGATGGTGAACTATGCCTGAATA-GGTGAAGCCAGAGGAAACTCTG-GTGGA-
GCTCGTAGCGATTCTGACGTGC-AATCGATCGTC-AATTTGGG---------------------------
----

>Phylloporia_manglietiae_Cui_13709
----------------AGA---

--GAGA-----------

---AGAGCG-
TGGATGGTCGAAAGC-TTTGA------TTCTTC--ATT-------------GA---CCCCTTTGT-
ATGTCTTGTGAAT--G-----T-ATC-GC-T-CC-GG----ATGGGTGAAAT-------
GAATACAACTTTCAACAACGGATCTCTTGGCTCTCGCATCGATGAAGAACGCAGCGAAATGCGA
TAAGTAATGTGAATTGCAGAATTCAGTGAATCATCGAATTTTTGAACGCACCTTGCGCCCTTTGG
-TATTCTGAGGGGCATGCCTGTTTGAGTGTCGTGTTAATCTCGAACCTCTC------AAG-
CCCCCCCCTGCTG------GGGCA----GGCTT----GAA-CAGGTTTGGAC-TTGGAGG-C------
--TTTGCTGGC-GCGT----------------GCTGC--
GCGTGGGTCGGCTCCTCTAAAAGGCATTAGC-TGGACTTTGG-----------------T-TT---
GCA-CGG-----------ATGGTGTGATAGTGA----------------CGTG-TTCAC----
CGGAAGTTGCTTG-CTCGA-TGGGTCTGC-TTC-----CAATCGTCTT-------------------
CGGAC-AA--------AGGTCG---TCTGC-GAC----C-TTTTCTTGA-CGGTTGACCTCA-
CCCCTAGTAACTGCGAGTGAAGCGGGAAGAGCTCAAATTTGTAATCT-G-GCG-GTC--
TGTGGCCGCCCGAGTTGTAATCTGTAGAGGCGTCGT-CCGCGTCG-
GCCCATGTACAAGTCTCCTGGAACGGAGCGTCGTAGAGGGTGAGAATCCCG-
TGCATGACGTGG-AC-CACCGATGG-
TTTGTGAGGCGCCCTCGAAGAGTCGCGTTGTTTGGGAATGCAGCGCAAAACGGGTGGTAAATTC
CATCTAAGGCTAAATATTGGCGAGAGACCGATAGCGAACAAGTACCGTGAGGGAAAGATGAAA
AGCACTTTGGAAAGAGAGTTAAACAGTACGTGAAATTGTTGAAAGGGAAACGCTTGAAGTCAG
TCGCGTCTCGAGGCATTCAGCCTTGC----TTCGGCTCGGCGTACTTGCCTC-AG--
GGATGGGTCAACATCAA-TTTT-GGC-
TGGTGGACAAGGGTGGGGGGAATGTGGCGTCGTTTCGGCGACGTG-TTATAGC-CTCTCGTC-
AAAC-ACGCCAGCTGAGATTGAGGAACTGCAGCACGCCCTT----TT-G-TGGCC-------
AGAGGCC-----------------TTC-TT---CG-CCTCATG-T-GAAGTGCTTAGGAT-
GTTGGCATAATGGCTTTAAGCGACCCGTCTTGAAACACGGACCAAGGAGTCTAACGTGCTTGTG
AGTGTTCGGGTGGAAAACCCTTGCGCGTAATGAAAGTGAAA-GTTGGGAG-----
CCCCCTCTCTTCTCGAAAAAAGAGGGG--GGGGGTGCACCGACGCCCGGCCCTGACG-----
TTCTCTGATGGTGCCGCGGTAGAGCAGGCACGTTGGG----
ACCCGAAAGATGGTGAACTATGCCTGAATAGGGCGAAGCCAGAGGAAACTCTG-
GTGGAGGCTCGTAGCGATTCTGACGTGCAAATCGATCGTCAAATTTGGGACAATGCCGC--GC-
--GCGGGGGGCGGGGAAGGGAAAT-
AAAGATCCTAAATTACTATAAGTGTCGTCCAAATGCTCGTGCCAGAAGACTCGGTAAGGCGAGA
GACGCAAACGTTAGTCGTCTTGATCAGGCGTAAAGGGTTTGTAGGCAGCTTTAAGCATCCAA-
TTTAATGTTTACTATTAT---------------------------------AATATTTACA---------------
----

AATTATAATATTTATTATAGGATTAATTAAAGCTAGAATCTAAAAGAGGCGA----
ATACAATGCTTGAG-CTTAGGAGTACTAACCGCAAACATCAAGTAGAGTGC-
TAAGGGCGAAGGC-
AGTTTTCCAATAAAGATTAACGCTGAGAAACGAAGGTGAGGATAGAAAAAAGGATGTGCGACC
AGAAAAGTTTTTTTAAGTATTGCGGATTTAGTCTGTCTTGTAATCTA------------------------
----
TCTTACACGAGTTCGCCTTAATATGGTAACAATTTTGTCTAAAGCTTAAGGTTTAATTTTAGAAAG

GTTAATAATTAGCTGTTTTCTAAGGTTAGAGAAATTGAATCTATGTAATTAAAATGGCACTGGCG
AGTTCGAGGCTGGTATCTCGAAAGACGGTCAAACTCGCGAGCACGCGCTGCTTGCGTTCACCCT
CGGTGTGCGTCAGCTGATCGTTGCCGTGAACAAGATGGACACGGCCAAGGTTGGT-
GGTGCAATTGA--CCTCT-CCGTAGCGAAGCTGAACC--CCGTTTG-------
AATACTCCAGTGGTCCGAGGAACGATTCAACGAAATTGTCAAGGAGACATCGAACTTCATTAAG
AAAGTCGGCTACAACCCCAAGGCCGTACCTTTCGTCCCGATCTCCGGATGGCACGGTGACAAT
ATGCTTGAGGAGTCGACCAAGTATGTCTAGCTT-TTCCT-----TGCGATGTAAGCG-----
TGAGCTGACGTGACATCT-TCCAGTATGCCTTGGTATAAGGGTTGGTCCAAGGAGACTAAGG---
---------GCGGTGTCGTT-
AAGGGGAAGACTCTTCTCGATGCCATTGATACCATCGAGTCCCCTGTCCGCGCAGAGAACAAG
CCCTTGCGACTTCCTCTCCAGGATGTGATTTCCGTTGGATCATTATCCGCTCCGGTGCTGGAATT
CCTGGAGGAATGGGGCTTGGAAAGCCTCGAGGAAAATGCACA---AGCTGCAA------
ATTTGACCAAGGTCTTCGTGAATGGAGTATGGCTCGGAGTACATCGAGACCCACACACACTGGT
CAATACGCTCCGAACGCTCAGGAGAAAGG---
AAGATCTGAACGCCGAGGTTTCAATTGTTCGTGACATACGTGAAAAGGAGGTCCGCATTTATAC
GGACGCAGGACGCGTTCTTCGACCGCTTTTCATTGTCGAGGACAATAGATTAAAGTTGAGACGT
CAGCACTTGCGCTGGCTTCAGGCGAAATGGAGGATT-------
GAGGAGAGGCCTAAAGACAGGGTTCT------------
GGATGAAGGCGAAGAATCAGCAGGCGAGACAATAACTGAGCTAGAAGACGACGGGATGCCAA
TAATACGGGAAGGGGTCCGAATGAAGAAGAAGAGGAAACCCGTCACCTGGAAGACATTGATTG
GCGACGGCGTTATTGAATACCTTGATGCAGAGGAGGAGGAAACAGTCATGATCTGTATGACAC
CAGAAGATCTGGTCGAATCTCGGTTAATGAGGCAAGGCCTGGATCCTCG---
AGCAGACGCGGAGTTCGATGCGTCTACGCGTCTCAAGCCTGTACCAACT------
GCTCACAACTGGACACATTGCGAGATTCATCCTAGCATGATTCTTGGTATATGTGCAAGTATTAT
CCCGTTCCC
>Phylloporia_manglietiae_Cui_5497

TCCCCTAGTAACTGCGAGTGAAGCGGGAAGAGCTCAAATTTGTAATCT-G-GCG-GTC--
TGTGGCCGCCCGAGTTGTAATCTGTAGAGGCGTCGT-CCGCGTCG-
GCCCATGTACAAGTCTCCTGGAACGGAGCGTCGTAGAGGGTGAGAATCCCG-
TGCATGACGTGG-AC-CACCGATGG-
TTTGTGAGGCGCCCTCGAAGAGTCGCGTTGTTTGGGAATGCAGCGCAAAACGGGTGGTAAATTC
CATCTAAGGCTAAATATTGGCGAGAGACCGATAGCGAACAAGTACCGTGAGGGAAAGATGAAA
AGCACTTTGGAAAGAGAGTTAAACAGTACGTGAAATTGTTGAAAGGGAAACGCTTGAAGTCAG
TCGCGTCTCGAGGCATTCAGCCTTGC----TTCGGCTCGGCGTACTTGCCTC-AG--
GGATGGGTCAACATCAA-TTTT-GGC-
TGGTGGACAAGGGTGGGGGGAATGTGGCGTCGTTTCGGCGACGTG-TTATAGC-CTCTCGTC-
AAAC-ACGCCAGCTGAGATTGAGGAACTGCAGCACGCCCTT----TT-G-TGGCC-------
AGAGGCC----------------TTC-TT---CG-CCTCATG-T-GAAGTGCTTAGGAT-
GTTGGCATAATGGCTTTAAGCGACCCGTCTTGAAACACGGACCAAGGAGTCTAACGTGCTTGTG
AGTGTTCGGGTGGAAAACCCTTGCGCGTAATGAAAGTGAAA-GTTGGGAG-----
CCCCCTCTCTTCTCGAAAAAAGAGGGG--GGGGGTGCACCGACGCCCGGCCCTGACG-----
TTCTCTGATGGTGCCGCGGTAGAGCAGGCACGTTGGG----
ACCCGAAAGATGGTGAACTATGCCTGAATAGGGCGAAGCCAGAGGAAACTCTG-
GTGGAGGCTCGTAGCGATTCTGACGTGCAAATCGATCGTCAAATTTGGG---------------
----

GGCACTGGCGAGTTCGAGGCTGGTATCTCGAAAGACGGTCAAACTCGCGAGCACGCGCTGCTT
GCGTTCACCCTCGGTGTGCGTCAGCTGATCGTTGCCGTGAACAAGATGGACACGGCCAAGGTT
GGT-GGTGCAATTGA--CCTCT-CCGTAGCGAAGCTGAACC--CCGTTTG-------
AATACTCCAGTGGTCCGAGGAACGATTCAACGAAATTGTCAAGGAGACATCGAACTTCATTAAG
AAAGTCGGCTACAACCCCAAGGCCGTACCTTTCGTCCCGATCTCCGGATGGCACGGTGACAAT
ATGCTTGAGGAGTCGACCAAGTATGTCTAGCTT-TTCCT-----TGCGATGTAAGCG-----
TGAGCTGACGTGACATCT-TCCAGTATGCCTTGGTATAAGGGTTGGTCCAAGGAGACTAAGG---
---------GCGGTGTCGTT-
AAGGGGAAGACTCTTCTCGATGCCATTGATACCATCGAGTCCCCTGTCCGCGCAGAGAACAAG
CCCTTGCGACTTCCTCTCCAGGATGTG-------------------------------------
----

>Phylloporia_minutipora_LWZ_20150531-13
ATGAGTTTTGA-------A---GCGGAG------------------------------TCTGCTGCTG--
GCGTATGAAG---------TGAAATG--CGCA-CGTG-CACGAT---------------------------
----TCT--GCG-----CTCAATCC--A--CTC-----------------AA-CCGC--TGTGCA-
CCTTTGCA--GAG-------------------
------------------------------------CGAGTAGG----------------CC----TTTCGTCC-
---------------------------TCTGAG-------------
GCGTTTAGTAACGAGTAAGGCGAGCTGG--------------------------------------
TGTGC--
---GTAAGT-----

--GCACCGGTA----
-----------GCCTGAA-------AGCGAACG---------------------TTGGTTTTAG---------
---AGGGA-GGTGAGGTCGAAAGC-TTTGA-----TTCTTT----AT-------------CA---
ACCTTTTGT-ATGTCTTGTGAATGCG-----T-ATT-GC-T-CC-TG----GCGGGCGAAAT-------
AAATACAACTTTCAACGACGGATCTCTTGGCTCTCGCATCGATGAAGAACGCAGCGAAATGCGA
TAAGTAATGTGAATTGCAGAATTCAGTGAATCATCGAATCTTTGAACGCACCTTGCGCCCTTTAG
-TATTCTGAAGGGCATGCCTGTTTGAGTGTCGTGTTAATCTCAAGCCCC--------GAG-
CCTTTTG-------------TTTA----GGCT-----TGG-ATGGTTTGGTC-TTGGAGG-C--------
TTTGCTGGC-GTGTGT-------------GCTTT--GCGCGCGTCGGCTCCTCTGAAAAGCATTAGC-
TGGACTGTGG----------------T-TT---GCA-TGG-----------ATGGTGTAATAGTAA-----
----------CGTG-TTCAC----CGGAAG-TGCTCGCTTTGA-TGGGTCTGC-TTC-----
CAATCGTCTT------------------CGGAC-AA--------GG-TCC---TTTGG-GAT----C-
TTTGCTTGACCGTTTGACCTCATCCCCTAGTAACTGCGAGTGAAGCGGGAAGGGTTCAAATTTGT
AATCT-G-GCG-GTC--CTCGGCCGTCTGAGTTGTAATCTGTAGAGGTGCCAT-CCGCGTCG-
GTCCGTGTACAAGTCTCCTGGAATGGAGCGTCATAGAGGGTGAGAATCCCG-
TCCATGACATGG-AT-CGCCGATGC-
TTTGTGAGGTGCTCTCGAAGAGTCGCGTTGTTTGGGAATGCAGCGCAAAATGGGTGGTAAATTC
CATCTAAGGCTAAATATTGGCGAGAGACCGATAGCGAACAAGTACCGTGAGGGAAAGATGAAA
AGCACTTTGGAAAGAGAGTTAAACAGTACGTGAAATTGTTGAAAGGGAAACGCTTGAAGTCAG
TCGCGTTTCGAGGCACTCAGCCTCGC----TTCGGCTTGGTGTACTTGCCTC-G---
AGATGGGTCAACGTCAA-TTTT-GGT-
TGGTGGAGAAGGGCGAGGGGAATGTTGCGTCGTTTCGACGATGTG-TTATAGC-CCCTTGTC-
GAAT-GCGYCAGCTGGGATTGAGGA-CTGCAGCACGCCC------TC-G-TGGCC-------
AGAGG----------------------TT---CG-CCTCATG-TGAATGTGCTTAGGAT-
GTTGGCATAATGGCTTTAAGCGACCCGTCTTGAAACACGGACCAAGGAGTCTAACACGCTTGCG
AGTGTTTGGGTGAAAAACCCTTGCGCGTAATGAAAGTGAAA-GTTGAGAG-----TCTT--------

----------CGTAG--GGAGACGCATCGATGCCCGGCCCTGATG-----
TTCTCTGACGGTGCCGCGGTGGAGCAGGCGTGTTGGG----
ACCCGAAAGATGGTGAACTATGCCTGAATAGGGCGAAGCCAGAGGAAACTCTG-
GTGGAGGCTCGTAGCGATTCTGACGTGCAAATCGATCGTCAAATTTGGGTCGATATCAC--AT--
-CGAGGATAACGTAAGGGAAAAG-
AATGATATTACCTTACTATTAGTGTCGTCCAAATGCTCGTGCCAGAAGACTCGGTAAGGCGAGA
GACGCAAACGTTAGTCGTCTTGATCAGGCGTAAAGGGTTTGTAGGCAGCTTTAAGTATCCAA-
TTTAATGTTTACTATTAT---------------------------------AATATTTACA---------------
----

AGTTATAATATTTATTAGAGGATCAATTAAAGCTAGAATCTAGAAGAGGTGA----
ATATAATGCTTGAG-CTTAGGAGTACTAACCGCAAACATCAAGTAGAGTGC-
TAAGGGCGAAGGC-
AGTTTTCCAATAAAGATTGACGCTGAGAAACGAAGGTGAGGATAGAAAAAAGGATTAGAGACC
CTTGTATCCCTCACTGTCAACGATGAATGGTAGTTACTAGGGAGAG----------------------
----
ATGACTTAGAGGCGATGTTAACACGTAAACCATTCCGCCTTGTGAGTACGACTGCAAAGTTGAA
AACAAAAAAATTAGTCGGTTTCGGAG--CAAACGAAGTGAAGC-
ATGTTATTTAATAGGTACTGGTGAGTTCGAGGCTGGTATCTCGAAAGACGGTCAGACTCGCGAG
CACGCTCTGCTTGCGTTCACACTCGGTGTGCGTCAGCTGATCGTTGCCGTGAACAAGATGGACA
CGGCCAAGGTTGGT-GGCGCTATCGA--CACCT-CCGTAGCAAAGCTGAACC--CCGTTTG----
---
AATACTCCAGTGGTCCGAGGAACGATTCAACGAAATTGTCAAGGAGACATCGAACTTCATTAAG
AAAGTCGGCTACAACCCCAAGGCCGTACCTTTCGTCCCAATCTCCGGATGGCACGGTGACAAC
ATGCTTGAGGAGTCGACCAAGTATGTCTGACTT-TTCCT-----TACAATGTAAGCG-----
TGAGCTGACGTGACATCT-CCCAGTATGTCTTGGTGCAAGGGCTGGTCTAAGGAGACGAAGG--
----------GCGGTGTCGTT-
AAGGGGAAGACTCTTCTCGATGCCATTGACGCCATCGAGCCCCCTGTCCGCCCAGAGAACAAG
CCCTTGCGACTTCCCCTCCAGGATGTG--

>Phylloporia_minutipora_Dai_9257

TCCCCTAGTAACTGCGAGTGAAGCGGGAAGGGTTCAAATTTGTAATCT-G-GCG-GTC--
CTCGGCCGTCTGAGTTGTAATCTGTAGAGGTGCCAT-CCGCGTCG-
GTCCGTGTACAAGTCTCCTGGAATGGAGCGTCATAGAGGGTGAGAATCCCG-
TCCATGACATGG-AT-CGCCGATGC-
TTTGTGAGGTGCTCTCGAAGAGTCGCGTTGTTTGGGAATGCAGCGCAAAATGGGTGGTAAATTC
CATCTAAGGCTAAATATTGGCGAGAGACCGATAGCGAACAAGTACCGTGAGGGAAAGATGAAA
AGCACTTTGGAAAGAGAGTTAAACAGTACGTGAAATTGTTGAAAGGGAAACGCTTGAAGTCAG
TCGCGTCTCGAGGCACTCAGCCTCGC----TTCGGCTTGGTGTACTTGCCTC-G---
AGATGGGTCAACGTCAA-TTTT-GGT-
TGGTGGAGAAGGGCGAGGGGAATGTTGCGTCGTTTCGACGATGTG-TTATAGC-CCCTTGTC-
GAAT-GCGCCAGCTGGGATTGAGGA-CTGCAGCACGCCC------TC-G-TGGCC-------
AGAGG----------------------TT---CG-CCTCATG-TGAATGTGCTTAGGAT-
GTTGGCATAATGGCTTTAAGCGACCCGTCTTGAAACACGGACCAAGGAGTCTAACACGCTTGCG
AGTGTTTGGGTGAAAAACCCTTGCGCGTAATGAAAGTGAAA-GTTGAGAG-----TCTT--------
----------CGTAG--GGAGACGCATCGATGCCCGGCCCTGATG-----
TTCTCTGACGGTGCCGCGGTGGAGCAGGCGTGTTGGG----
ACCCGAAAGATGGTGAACTATGCCTGAATAGGGCGAAGCCAGAGGAAACTCTG-
GTGGAGGCTCGTAGCGATTCTGACGTGCAAATCGATCGTCAAATTTGGG------------------
----

>Phylloporia_minutissima_Dai_21223

TCCCTTAGTAACTGCGAGTGAAGCGGGAAGAGCTCAAATTTGGAATCT-G-ACG-GTC--
TCTGACCGTCCGAGTTGTAATCTGTAGAGGCATCGT-CCGCGTCG-
GCCTGTGTGCAAGTCTCCTGGAACGGAGCGTCGTAGAGGGTGAGAATCCCG-
TCCGTGACACAG-AC-CACCGATGC-
TCTGTGAGGTGCCCTCGAAGAGTCGCGTTGTTTGGGAATGCAGCGCAAAATGGGTGGTAAATTC

CATCTAAGGCTAAATACCGGCGAGAGACCGATAGCGAACAAGTACCGTGAGGGAAAGATGAA
AAGCACTTTGGAAAGAGAGTTAAACAGTACGTGAAATTGTTGAAAGGGAAACGCTTGAAGTCA
GTCGCGTCCCGTGGCACTCAGCCTTGC----TTCGGCTTGGTGTACTTGCTTT-G---
GGATGGGTCAACATCAA-TCTT-GGA-
CGGTGGATAAGGGCGAGGGGAATGTAGCGTCGTTTCGACGACGTG-TTATAGC-CCCTCGTC-
GAAT-ACGCCGGCTGGGATTGAGGA-TTGCAGCACGCCC------TT-G-TGGCC-------
AGGGGGT--------------------TC---GC-CCTCATG-T-AACGTGCTTAGGAT-
GTTGGCATAATGGCTTTAAGCGACCCGTCTTGAAACACGGACCAAGGAGTCTAACATGCTTGCG
AGTGTTTGGGTGGAAAACTCTTGCGCGTAATGAAAGTGAAA-GTTGGGAG-----CCTC-------
-----------TGTGA--GGGGGTGCACCGACGCCCGGCCCTGAAG-----
TTCTCTGACGGTGCTGCGGTAGAGCAAGCATGTTGGG----
ACCCGAAAGATGGTGAACTATGCCTGAATAGGGTGAAGCCAGAGGAAACTCTG-
GTGGAGGCTCGTAGCGATTCTGACGTGCAAATCGATCGTCAAATTTGGG------------------
----

>Phylloporia_mori_Wu_1105-2

TCCCCTAGTAACTGCGAGTGAAGCGGGAAGAGCTCAAATTTGTAATCT-G-GCG-GTC--
TTGGGCCGTCCGAGTTGTAATCTGAAGAGGCATTGT-CCGCGTCG-
GACCGTGTACAAGTCTCCTGGAATGGAGCGTCATAGAGGGTGAGAATCCCG-
TCCATGACACGG-AC-CGCCGATGC-
TTTGTGAGGTGCCCTCGAAGAGTCGCGTTGTTTGGGAATGCAGCGCAAAATGGGTGGTAAATTC
CATCTAAAGCTAAATACTGGCGAGAGACCGATAGCGAACAAGTACCGTGAGGGAAAGATGAAA
AGCACTTTGAAAAGAGAGTTAAACAGTACGTGAAATTGTTGAAAGGGAAACGCTTGAAGTCAGT
CGCGTCTTGTGGTACTCAGCCTTGC----TTCGGCTTGGTGTACTTACCAT-TT--
GGACGGGTCAACGTCAA-TTTC-GAC-
CGGTGGACAAGGGCGAGGGGAATGTAGCGTCGTTTCGGCGGCGTG-TTATAGA-CTCTTGTT-
GTAT-ACACCGGTTGGGATTGAGGA-CCGCAGCACGCCC------TT-G-TGGCC-------
GGGGG----------------------TT---CG-CCCTACG-T-TTGGTGCTTAGGAT-
GTTGGCATAATGGCTTTAAGCGACCCGTCTTGAAACACGGACCAAGGAGTCTAACATGCTTGCG
AGTGTTTGGGTGGAAAACCCTTGCGCGTAATGAAAGTGAAAGGTTGGGAA-----CCTC-------
-----------CGCGA--GGGGGTGCACCGACGCCCGGCCCTAACG-----
TTCTCTGACGGTGCTGCGGTAGAGCAAGTATGTTGGG----
ACCCGAAAGATGGTGAACTATGCCTGAATAGGGTGAAGCCAGAGGAAACTCTG-
GTGGAGGCTCGTAGCGATTCTGACGTGCAAATCGATCGTCAAATTTGGG------------------
----

>Phylloporia_mori_Wu_1105-3

TCCCCTAGTAACTGCGAGTGAAGCGGGAAGAGCTCAAATTTGTAATCT-G-GCG-GTC--
TTGGGCCGTCCGAGTTGTAATCTGAAGAGGCATTGT-CCGCGTCG-
GACCGTGTACAAGTCTCCTGGAATGGAGCGTCATAGAGGGTGAGAATCCCG-
TCCATGACACGG-AC-CGCCGATGC-
TTTGTGAGGTGCCCTCGAAGAGTCGCGTTGTTTGGGAATGCAGCGCAAAATGGGTGGTAAATTC

CATCTAAAGCTAAATACTGGCGAGAGACCGATAGCGAACAAGTACCGTGAGGGAAAGATGAAA
AGCACTTTGAAAAGAGAGTTAAACAGTACGTGAAATTGTTGAAAGGGAAACGCTTGAAGTCAGT
CGCGTCTTGTGGTACTCAGCCTTGC----TTCGGCTTGGTGTACTTACCAT-TT--
GGACGGGTCAACGTCAA-TTTC-GAC-
CGGTGGACAAGGGCGAGGGGAATGTAGCGTCGTTTCGGCGGCGTG-TTATAGA-CTCTTGTT-
GTAT-ACACCGGTTGGGATTGAGGA-CCGCAGCACGCCC------TT-G-TGGCC-------
GGGGG-----------------------TT---CG-CCCTACG-T-TTGGTGCTTAGGAT-
GTTGGCATAATGGCTTTAAGCGACCCGTCTTGAAACACGGACCAAGGAGTCTAACATGCTTGCG
AGTGTTTGGGTGGAAAACCCTTGCGCGTAATGAAAGTGAAAGGTTGGGAA-----CCTC-------
-----------CGCGA--GGGGGTGCACCGACGCCCGGCCCTAACG-----
TTCTCTGACGGTGCTGCGGTAGAGCAAGTATGTTGGG----
ACCCGAAAGATGGTGAACTATGCCTGAATAGGGTGAAGCCAGAGGAAACTCTG-
GTGGAGGCTCGTAGCGATTCTGACGTGCAAATCGATCGTCAAATTTGGG------------------
----

>Phylloporia_moricola_Wu1807-1

TCCCCTAGTAACTGCGAGTGAAGCGGGAAGAGCTCAAATTTGTAATCT-G-GCG-GTC--
TTGGGCCGTCCGAGTTGTAATCTGAMGAGGCATTGT-CCGCGTCG-
GACCGTGTACAAGTCTCCTGGAATGGAGCGTCATAGAGGGTGAGAATCCCG-
TCCATGACACGG-AC-CGCCGATGC-
TTTGTGAGGTGCCCTCGAAGAGTCGCGTTGTTTGGGAATGCAGCGCAAAACGGGTGGTAAATTC
CATCTAAAGCTAAATACTGGCGAGAGACCGATAGCGAACAAGTACCGTGAGGGAAAGATGAAA
AGCACTTTGAAAAGAGAGTTAAACAGTACGTGAAATTGTTGAAAGGGAAACGCTTGAAGTCAGT
CGCGTCTTGTGGTACTCAGCCTTGC----TTCGGCTTGGTGTACTTACCAT-TT--
GGACGGGTCAACGTCAA-TTTT-GAC-
CGGTGGACAAGGGCGAGGGGAATGTAGCGTCGTTTCGGCGGCGTG-TTATAGA-CTCTTGTT-
GTAT-ACACCGGTTGGGATTGAGGA-CCGCAGCACGCCC------TT-G-TGGCY-------
GGGGG----------------------TT---CG-CCCTACG-T-TCGGTGCTTAGGAT-
GTTGGCATAATGGCTTTAAGCGACCCGTCTTGAAACACGGACCAAGGAGTCTAACATGCTTGCG
AGTGTTTGGGTGGAAAACCCTTGCGCGTAATGAAAGTGAAAGGTTGGGAA-----CCTC-------
-----------CGCGA--GGGGGTGCACCGACGCCCGGCCCTAACG-----
TTCTCTGACGGTGCTGCGGTAGAGCAAGTATGTTGGG----
ACCCGAAAGATGGTGAACTATGCCTGAATAGGGTGAAGCCAGAGGAAACTCTG-
GTGGAGGCTCGTAGCGATTCTGACGTGCAAATCGATCGTCAAATTTGGG-------------------
----

>Phylloporia_moricola_Wu1807-5

TCCCCTAGTAACTGCGAGTGAAGCGGGAAGAGCTCAAATTTGTAATCT-G-GCG-GTC--
TTGGGCCGTCCGAGTTGTAATCTGAAGAGGCATTGT-CCGCGTCG-
GACCGTGTACAAGTCTCCTGGAATGGAGCGTCATAGAGGGTGAGAATCCCG-
TCCATGACACGG-AC-CGCCGATGC-
TTTGTGAGGTGCCCTCGAAGAGTCGCGTTGTTTGGGAATGCAGCGCAAAACGGGTGGTAAATTC

CATCTAAAGCTAAATACTGGCGAGAGACCGATAGCGAACAAGTACCGTGAGGGAAAGATGAAA
AGCACTTTGAAAAGAGAGTTAAACAGTACGTGAAATTGTTGAAAGGGAAACGCTTGAAGTCAGT
CGCGTCTTGTGGTACTCAGCCTTGC----TTCGGCTTGGTGTACTTACCAT-TT--
GGACGGGTCAACGTCAA-TTTT-GAC-
CGGTGGACAAGGGCGAGGGGAATGTAGCGTCGTTTCGGCGGCGTG-TTATAGA-CTCTTGTT-
GTAT-ACACCGGTTGGGATTGAGGA-CCGCAGCACGCCC------TT-G-TGGCY-------
GGGGG----------------------TT---CG-CCCTACG-T-TCGGTGCTTAGGAT-
GTTGGCATAATGGCTTTAAGCGACCCGTCTTGAAACACGGACCAAGGAGTCTAACATGCTTGCG
AGTGTTTGGGTGGAAAACCCTTGCGCGTAATGAAAGTGAAAGGTTGGGAA-----CCTC-------
-----------CGCGA--GGGGGTGCACCGACGCCCGGCCCTAACG-----
TTCTCTGACGGTGCTGCGGTAGAGCAAGTATGTTGGG----
ACCCGAAAGATGGTGAACTATGCCTG-ATAGGGTGAAGCCAGAGGAAACTCTG-
GTGGAGGCTCGTAGCGATTCTGACGTGCAAATCGATCGTCAAATTTGGG------------------
----

>Phylloporia_murrayae_Wu_1404-4

TCCCTAGTAACTGCGAGTGAAGCGGGAAGAGCTCAAATTTGTAATCT-G-GTG-GAC--
TTTGGCCGCCCGAGTTGTAATCTGTAGAGGCGTCGT-CTGCGTCG-GCCCGTGTACAAGTCTCC-
GGGATGGAGCGTCATAGAGGGTGAGAATCCCG-TCCATGACACGG-AC-CACCGATGC-
TCTGTGAGGCGCCCTCAAAGAGTCGCGTTGTTTGGGAATGCAGCGCAAAGCGGGTGGTAAATT
CCATCTAAGGCTAAATATTGGCGAGAGACCGATAGCGAACAAGTACCGTGAGGGAAAGATGAA
AAGCACTTTGAAAAGAGAGTTAAACAGTACGTGAAATTGTTGAAAGGGAAACGCTTGAAGTCA
GTCGCGTCTCGAGGCACTCAGCCTTGC----TTTGGCTTGGTGTACTTGCCTT-G---
GGACGGGTCAACATCAA-TTTT-GGC-
CGGTGGACAAGGGCGAGGGGAATGTAGCGTTGTTTCGGCGACGTG-TTATAGC-CCCTCGTC-
AGAT-GCATCGGCTAGGATTGAGGA-CTGCAGCACGCCC------TT-GTAGGCC-------
GGGGG----------------------AT---CG-CCCCACG-T-AACGTGCTTAAGAT-
GTTGGCATAATGGCTTTAAGCGACCCGTCTTGAAACACGGACCAAGGAGTCTAACATGCTTGCG
AGTGTTCGGGTGGAAAACCCTTGCGCGCAATGAAAGTGAAA-GTTGGGAG-----CCTT-------
-----------CGTAA-AGGAGGTGCACCGATGCCCGGCTCTGACG-----
TTCTCTGACGACGCCGTGGTAGAGCAAGTCTGTTGGG----
ACCCGAAAGATGGTGAACTATGCCTG-ATAGGGCGAAGCCAGAGGAAACTCTG-GTGGA-
GCTCGTAGCGATTCTGACGTGCAAATCGATCGTCAAATTTGGG--------------------------
----

>Phylloporia_murrayae_Wu_1404-5

TCCCTTAGTAACTGCGAGTGAAGCGCGAAGAGCTCCAATTTGTAATCT-G-GTG-GAC--
TTTGGCCGCCCGAGTCGTAATCTGTAGAGGCGTCGTCCTGCGTCG-
GCCCGTGTACAAGTCTCCTGGGATGGAGCGTCATAGAGGGTGAGAATCCCG-
TCCATGACACGG-AC-CACCGATGC-
TCTGTGAGGCGCCCTCAAAGAGTCGCGTTGTTTGGGAATGCAGCGCAAAGCGGGTGGTAAATT
CCATCTAAGGCTAAATATTGGCGAGAGACCGATAGCGAACAAGTACCGTGAGGGAAAGATGAA

AAGCACTTTGAAAAGAGAGTTAAACAGTACGTGAAATTGTTGAAAGGGAAACGCTTGAAGTCA
GTCGCGTCTCGAGGCACTCAGCCTTGC----TTTGGCTTGGTGTACTTGCCTT-G---
GGACGGGTCAACATCAA-TTTT-GGC-
CGGTGGACAAGGGCGAGGGGAATGTAGCGTTGTTTCGGCGACGTG-TTATAGC-CCCTCGTC-
AGAT-GCATCGGCTAGGATTGAGGA-CTGCAGCACGCCC------TT-GTAGGCC-------
GGGGG----------------------AT---CG-CCCCACG-T-AACGTGCTTAAGAT-
GTTGGCATAATGGCTTTAAGCGACCCGTCTTGAAACACGGACCAAGGAGTCTAACATGCTTGCG
AGTGTTCGGGTGGAAAACCCTTGCGCGCAATGAAAGTGAAA-GTTGGGAG-----CCTT-------
-----------CGTAA-AGGAGGTGCACCGATGCCCGGCTCTGACG-----
TTCTCTGACGACGCCGTGGTAGAGCAAGTCTGTTGGG----
ACCCGAAAGATGGTGAACTATGCCTG-ATAGGGCGAAGCCAGAGGAAACTCTG-GTGGA-
GCTCGTAGCGATTCTGACGTGC-AATCGATCGTC-AATTTGGG----------------------------
----

>Phylloporia_nandinae_Dai_10588

TCCCCTAGTAACTGCGAGTGAAGCGGGAAGAGCTCAAATTTGTAATCT-G-GCG-GTC--
CCTGGCCGTCCGAGTTGTAATCTGTAGAGGCGTCGT-CCGCGTCG-
GCCCGTGTACAAGTCTCCTGGAATGGAGCGTCATAGAGGGTGAGAATCCCG-
TCCATGACACGG-AC-CACCGATGC-
TCTGTGAGATGCCCTCGAAGAGTCGCGTTGTTTGGGAATGCAGCGCAAAATGGGTGGTAAATTC
CATCTAAGGCTAAATATTGGCGAGAGACCGATAGCGAACAAGTACCGTGAGGGAAAGATGAAA
AGCACTTTGAAAAGAGAGTTAAACAGTACGTGAAATTGTTGAAAGGGAAACGCTTGAAGTCAGT
CGCGTTCCAAGGCATTCAGCCTCGC----TTTGGCTTGGTGTATTTGCCTC-G---
GGACGGGTCAACATCGA-TTTT-GGT-
TGGTGGAAAAGGGTGAGGGGAATGTAGCGTCGCTTTGGCGACGTG-TTATAGC-CCCTCGTC-
GAAT-ACACCGGCTGGGATCGAGGA-CCGCAGCATGCCC------TT-G-TGGCC-------
AGGGG----------------------TT---CG-CCCCATG-T-AACATGCTTAGGAT-
GTTGGCGTAATGGCTTTAAGCGACCCGTCTTGAAACACGGACCAAGGAGTCTAACATGCTTGCG
AGTGTTCGGGTGGAAAACCCTTGCGCGTAATGAAAGTAAAA-GTTGGGAG-----CCTT-------
-----------CACGA--GAGGGTGCATCGACGCCCGGCCCAGAAG-----
TTTTCTGACGGTGCTGCGGTAGAGCAAGTACGTTGGG----
ACCCGAAAGATGGTGAACTATGCCTGAATAGGGCGAAGCCAGAGGAAACTCTG-
GTGGAGGCTCGTAGCGATTCTGACGTGCAAATCGATCGTCAAATTTGGG------------------
----

>Phylloporia_nandinae_Dai_10625

AGTAACTGCGAGTGAAGCGGGAAGAGCTCAAATTTGTAATCT-G-GCG-GTC--
CCTGGCCGTCCGAGTTGTAATCTGTAGAGGCGTCGT-CCGCGTCG-
GCCCGTGTACAAGTCTCCTGGAATGGAGCGTCATAGAGGGTGAGAATCCCG-
TCCATGACACGG-AC-CACCGATGC-
TCTGTGAGATGCCCTCGAAGAGTCGCGTTGTTTGGGAATGCAGCGCAAAATGGGTGGTAAATTC
CATCTAAGGCTAAATATTGGCGAGAGACCGATAGCGAACAAGTACCGTGAGGGAAAGATGAAA

AGCACTTTGAAAAGAGAGTTAAACAGTACGTGAAATTGTTGAAAGGGAAACGCTTGAAGTCAGT
CGCGTTCCAAGGCATTCAGCCTCGC----TTTGGCTTGGTGTATTTGCCTC-G---
GGACGGGTCAACATCGA-TTTT-GGT-
TGGTGGAAAAGGGTGAGGGGAATGTAGCGTCGCTTTGGCGACGTG-TTATAGC-CCCTCGTC-
GAAT-ACACCGGCTGGGATCGAGGA-CCGCAGCATGCCC------TT-G-TGGCC-------
AGGGG---------------------TT---CG-CCCCATG-T-AACATGCTTAGGAT-
GTTGGCGTAATGGCTTTAAGCGACCCGTCTTGAAACACGGACCAAGGAGTCTAACATGCTTGCG
AGTGTTCGGGTGGAAAACCCTTGCGCGTAATGAAAGTAAAA-GTTGGGAG-----CCTT-------
-----------CACGA--GAGGGTGCATCGACGCCCGGCCCAGAAG-----
TTTTCTGACGGTGCTGTGGTAGAGCAAGTACGTTGGG----
ACCCGAAAGATGGTGAACTATGCCTGAATAGGGCGAAGCCAGAGGAAACTCTG-
GTGGAGGCTCGTAGCGATTCTGACGTGCAAATCGATCGTCAAATTTGGG-----------------
----

GGTACTGGTGAATTCGAGGCTGGTATCTCGAAGGACGGTCAGACCCGCGAGCATGCTCTGCTT
GCGTTCACCCTTGGTGTACGTCAGCTGATCGTCGCTGTGAACAAGATGGACACGGCCAAGGTTG
GTGGGCGCTATTGA--CCT---CTGTAACGAAGCTGAACC--CCGTTCG-------
AATAATCTAGTGGTCTGAGGAACGATTCAACGAAATCGTTAAGGAGACGTCGAACTTCATCAAG
AAAGTCGGCTACAACGCGAAGGGGGTAGCTTTCACCCCTATCTCCGGATGGTTCGGTGACAAC
ATGCTCGAGGAGTCGACCAAGTATACTTGACTT-TTTCCTGATTTATAACATAAGCG------
AAGCTGACGTGACATTTCTCAAGTATGCCTTGGTACAAGGGCTGGTCCAAGGAGACGAAGG---
---------GTGGTGTCGTC-
AAGGGAAAGACTCTCCTCGACGCCATCGATGCCATCGAACCTCCTGTCCGTCCGGAGAACAAG
CCCTTGCGACTTCCTCTCCAGGATGTGATTTCTGTTGGTTCATTATCCGCTCCGGTACTGGAATTC
TTGGAGGAATGGGGATTGGAGAGCCTTGAGGAGAACGCCCA---AGCTGCAA------
ATCTGACCAAGGTTTTCGTGAATGGAGTATGGCTCGGAGTACACCGAGATCCACACACCCTTGT
CAACACACTTCGAACGCTTAGGCGAAAGG---
AGGACCTCAACGCTGAGGTTTCCATTGTTCGTGACATACGTGAAAAGGAGGTTCGCATCTATAC
GGACGCAGGACGCGTTCTTCGACCGCTTTTCATTGTCGAGGACAATAGATTAAAGCTGAGACGT
CAGCACCTGCGGTGGCTTCAGACGAAGTGGAGGATT-------
GAGGAGAGGCCCAAAGACAGGGTCCT------------
GGATGAAGGCGAAGAGTCAGCAGGCGAGACAATAACTGAGCTAGAAGACGATGGGAAACCAA
TAATACGGGAAGGGGCTCGAATGAAGAAGAAGAGGAAACCGGTCACCTGGAAGACGCTGATT
AGTGATGGTGTCATTGAATACCTTGATGCAGAGGAGGAGGAAACAGTCATGATCTGCATGACAC
CAGAAGATCTTGTCGAATCTCGGTTAATGAGGCAAGGACTGGATCCTCG---
GGCGGACACGGAGTTCGATGCTTCTACGCGTCTCAAGCCTGTTCCAACT------
GCTCATAACTGGACACATTGCGAGATTCATCCCAGTATGATTCTAGGTATATGTGCCAGTATCAT

TCCGTTCCC
>Phylloporia_oblongospora_Zhou_179
AAGAGTTTTGA-----A-GG-CATCGGG------------------------------TCTGTTGCTG--
ATGTGTG-------------AGAAGG--CACATTATG-CATGGC----------------------------
----TC-TGTG-----CACGATCT--A--CTT-----------------AA-CCTC--TGTGCA-
CATTTACA---AAG------------------
-----------------------------------TAAAGTAGG----------------C------TTCATGC-
---------------------------TTTAAAG------------
CAGGTTGACGCTGAGTAGAACGAGA---
GCCT---

--CCTTT---
-----------------------------------GTGTGCAA-GGTG-------------GGGCCAGTA---
------------GTTTAAA-------AGCAAATG---------------------TCATTTGTTT---------
---GAAGC-ATTGTTGTTGAAAGC-TTTGA-------CTTT----AT-------------CA---AC--
TCTAT-ATGTGTTGTGAAT--G----CT-GTT-GC-C-TC-TT----GGAGGTGAAAC-T-----
AGATACAACTTTCAACAATGGATCTCTTGGCTCTCGCATCGATGAAGAACGCAGCGAAATGCGA
TAAGTAATGTGAATTGCAGAATTCAGTGAATCATCGAATCTTTGAACGCACCTTGCGCCCTTTGG
-TGTTCCAAAGGGCATGCCTGTTTGAGTGTCAGGTTCATCTCAGACCCCATCAGAGCAGG-
GTTTTTC--------------CTA----GGCT-----TGA-AGGGTTTGGAT-TTGGAGG-C--------
TCTGCTGTT-GTGCACGTGCTTT----GTGCCGT--
GTTATTATCAGCTCCTCTTAAAACCATCAGC-AGAGCTTTAG----------------T-TG---GCA-
TAA-----------GTGGTGTAGTAGTAA---------------AGTG-TTCAC----CAGAAG-
TGCTTG-TCTAG-AGGGTCTGC-TTT-----TAATTGTCTT------------------TAGAC-
AAACAAGGAGGG-TTG---TTTGT-AAC----C-TTTGCTTGACACGTTGACCTCA----
CTAGTAACTGCGAGTGAAGCGGGAAGAGCTCAAATTTGTAATCT-A-ATG-GCC--
TTTGGCCGTTCGAGTTGTAATCTGTAGAGGCGTTGT-CTGTGTCG-
GCCCATGTACAAGTCTCCTGGAATGGAGCGTCATAGAGGGTGAGAATCCCG-
TCCATGACATGG-GT-CCCCGATGC-
TCTGTGAGGCACCCTCGAAGAGTCGCGTTGTTTGGGAATGCAGCGCAAAATGGGTGGTAAATTC
CATCTAAGGCTAAATATTGGCGAGAGACCGATAGCGAACAAGTACCGTGAGGGAAAGATGAAA
AGCACTTTGAAAAGAGAGTTAAACAGTACGTGAAATTGTTGAAAGGGAAACGCTTGAAGTCAGT
CATGTCTTGAGACACTCAGCCTTGC----TTCGGCTTGGTGTATTTGTTTT-G---
AGACAGGTCAGCATCAA-TTTT-AGC-
CGATGGACAAAGGTGAGAGGAATGTGGTGTTGCTTTAGCGACATG-TTATAGC-CTCTTGCT-
GAATGGCGTTGGCTAGGATTGAGGA-TGGCAGCATGCCC------TT-G-TGGTT-------
GGAGG----------------------TT---CA--CTCACA-T-AACATGCTTAGGAT-
GCTGGCATAATGGCTTTAAGCGACCCGTCTTGAAACACGGACCAAGGAGTCTAACACGCTTGC
GAGTGTTCGGGTGGAAAACCCTTGCGCGTAATGAAAGTGAAA-GTTGAGAG-----CCCT------
------------TGAGA--GAGGGTGCACCGACGCCCGGCCCTGATG-----
ATCTCTGATGGTGCTGCGGTGGAGCAAGTGCGTTAGG----
ACCCGAAAGATGGTGAACTATGCCTGAATAGGGCGAAGCCAGAGGAAACTCTG-
GTGGAGGCTCGTAGCGATTCTGACGTGCAAATCGATCGTCGAATTTGGG------------------

----

>Phylloporia_oreophila_Cui_2219
ATGAGTTTTGA-----A-A---GCAGAG------------------------------TCTGCTGCTG--
GTGCA---------------GAAACG--CGCA-TGTG-CACGGC----------------------------
---TCT--GTG-----CTCAATCC--A--CTC-----------------AA-CCCC--TGTGCA-
CCTTTGCA---GAG-------------------
-------------------------------------CGAGTAGG----------------C------TTCACCC-
----------------------------TTTGAAGA-----------
GTGACCAATAGTGAGTAAGGCGAGC---
GTGT---
--GTGA---------

--GCACTAGTA-------
--------GTCTGAA-------AGCGAGCG---------------------TCTTTCATTT-----------
AGAGAGAAGTGCGGTCGAAAGC-TTTGA------TCTTT----AT-------------CA---
ACCTTTCGT-ATGTCATGTGAAT--G-----T-GTT-GC-T-CC-TT----GTGGGCGAAAT-------
AAATACAACTTTCAACAACGGATCTCTTGGCTCTCGCATCGATGAAGAACGCAGCGAAATGCGA
TAAGTAATGTGAATTGCAGAATTCAGTGAATCATCGAATCTTTGAACGCACCTTGCGCCCTTTGG

-TATTCCGAAGGGCATGCCTGTTTGAGTGTCATGTTTATCTCAAACCCC--------
AAACCCTTTTG--------------TTA----GGTTT----TGG-ATGGTTTGGAC-TTGGAGG-C----
----TTTGCTGGT-GTTGC--------------GCTCT--
GCGTGTGTCGGCTCCTCTGAAAAGCATTAGC-TGGACTTTGG----------------T-TC---
GCG-TGA-----------ATGGTGTGATAGTAA---------------TGTG-TTCGC----CGGAAG-
CGCTTG-CCTAA-CGGGTCTGC-TCC-----TAATTGTCTT------------------CGGAC-AA---
-----GG-TCC---TTTGT-GAC----C-TTCGCTTGACCGTTTGACCTCA----
CTAGTAACTGCGAGTGAAGCGGGAAGAGCTCAAATTTGTAATCT-G-GCG-GTC--
TCTGGCCGTCCGAGTTGTAATCTGTAGAGGCGTCGT-CCGCGTCG-
GCCCGTGTACAAGTCTCCTGGGATGGAGCGTCATAGAGGGTGAGAATCCCG-
TCCATGACATGG-AC-CACCGATGC-
TCTGTGAGGTGCCCTCGAAGAGTCGCGTTGTTTGGGAATGCAGCGCAAAATGGGTGGTAAATTC
CATCTAAGGCTAAATATTGGCGAGAGACCGATAGCGAACAAGTACCGTGAGGGAAAGATGAAA
AGCACTTTGGAAAGAGAGTTAAACAGTACGTGAAATTGTTGAAAGGGAAACGCTTGAAGTCAG
TCGCGTCCCGTGGCATTCAGCCTTGC----TTCGGCTTGGTGTACTTGCCTT-G---
GGATGGGTCAACATCAA-TTTT-GGC-
CGGTGGACAAGGGTGAGGGGAATGTAGCGTCGTTTCGGCGACGTG-TTATAGC-TCCTTGTC-
GAAT-ACACTGGCTGGGATTGAGGA-CCGCAGCACGCCC------TT-G-TGGCC-------
AGGGG----------------------TT---CG-CCCCATG-T-AACGTGCTTAGGAT-
GTTGGCATAATGGCTTTAAGCGACCCGTCTTGAAACACGGACCAAGGAGTCTAACATGCTTGCG
AGTGTTCGGGTGGAAAACCCTTGCGCATAATAAAAGTGAAA-GTTGGGAG-----CCTC-------
-----------CGCAA--GGGGGTGCACCGACACCCGGCCCTGACG-----
TTCTCTGACGGTGCTGCGGTAGAGCAAGTATGTTGGG----
ACCCGAAAGATGGTGAACTATGCCTGAATAGGGCGAAGCCAGAGGAAACTCTG-
GTGGAGGCTCGTAGCGATTCTGACGTGCAAATCGATCGTCAAATTTGGGTTGATGTGAC--AT--
-CGAGGATAACGTAAGGGAAAAT-
AATGATATTACCTTACTATTAGTGTCGTCCAAATGCTCGTGCCAGAAGACTCGGTAAGGCGAGA
GACGCAAACGTTAGTCGTCTTGATCAGGCGTAAAGGGTTTGTAGGCAGCTTTAAGTATCCAA-
TTTAATGTTTACTATTAT--------------------------------AATATTTACA--------------
----

TGTTATAATTTTTATTAAAGGATCAATTAAAGCTAGAATCTAGAAGAGGTGA----
ATACAATGCTTGAG-CTTAGGAGTACTAACCGCAAACATCAAGTAGAGTGC-
TAAGGGCGAAGGC-
AGTTTTCCAAAACAGATTGACGCTGAGAAACGAAGGTGAGGATAGAAAAAAGGATTAGATACC
CTTGTATCCCTCACTGTCAACGATGAATGGTAGTTACTAGGGAGAA----------------------
----
ATAACCTAGAGGCGATGTTAACACGTAAACCATTCCGCCTTGTGAGTACGACTGCAAAGTTGAA
AACAAAAAAATTAGTCGGTTTCGGAG--CAAACGAAGTGAAGC-ATGTTATTTAATT----------
----

>Phylloporia_oreophila_LWZ_20190811-27a
ATGAGTTTTG------A-A---GCAGAG------------------------------TCTGCTGCTG--
GTGCA---------------GAAACG--CGCA-TGTG-CACGGC----------------------------
---TCT--GTG-----CTCAATCC--A--CTC-----------------AA-CCCC--TGTGCA-
CCTTTGCA--GAG-------------------
------------------------------------CGAGTAGG----------------C------TTCACCC-
---------------------------TTTGAAGA-----------
GTGACCAATAGTGAGTAAGGCGAGC--
GTGT--
---GTGA---------

---GCACTAGTA-------
--------GTCTGAA-------AGCGAGCG---------------------TCTTTCATTT-----------
AGAGAGAAGTGCGGTCGAAAGC-TTTGA------TCTTT----AT-------------CA---
ACCTTTCGT-ATGTCATGTGAAT--G-----T-GTT-GC-T-CC-TT----ATGGGCGAAAT-------
AAATACAACTTTCAACAACGGATCTCTTGGCTCTCGCATCGATGAAGAACGCAGCGAAATGCGA
TAAGTAATGTGAATTGCAGAATTCAGTGAATCATCGAATCTTTGAACGCACCTTGCGCCCTTTGG
-TATTCCGAAGGGCATGCCTGTTTGAGTGTCATGTTTATCTCAAACCCC--------
AAACCCTTTTG--------------TTA----GGTTT----TGG-ATGGTTTGGAC-TTGGAGG-C----
----TTTGCTGGT-GTTGC--------------GCTCT--
GCGTGTGTCGGCTCCTCTGAAAAGCATTAGC-TGGACTTTGG----------------T-TC---
GCG-TGA-----------ATGGTGTGATAGTAA---------------TGTG-TTCGC----CGGAAG-
CGCTTG-CCTAA-CGGGTCTGC-TCC-----TAATTGTCTT------------------TGGAC-AA---
-----GG-TCC---TTTGT-GAC----C-TTCGCTTGACCGTTTGACCTCA-
CCCCTAGTAACTGCGAGTGAAGCGGGAAGAGCTCAAATTTGTAATCT-G-GCG-GTC--
TCTGGCCGTCCGAGTTGTAATCTGTAGAGGCGTCGT-CCGCGTCG-
GCCCGTGTACAAGTCTCCTGGGATGGAGCGTCATAGAGGGTGAGAATCCCG-
TCCATGACATGG-AC-CACCGATGC-
TCTGTGAGGTGCCCTCGAAGAGTCGCGTTGTTTGGGAATGCAGCGCAAAATGGGTGGTAAATTC
CATCTAAGGCTAAATATTGGCGAGAGACCGATAGCGAACAAGTACCGTGAGGGAAAGATGAAA
AGCACTTTGGAAAGAGAGTTAAACAGTACGTGAAATTGTTGAAAGGGAAACGCTTGAAGTCAG

TCGCGTCCCGTGGCATTCAGCCTTGC----TTCGGCTTGGTGTACTTGCCTT-G---
GGATGGGTCAACATCAA-TTTT-GGC-
CGGTGGACAAGGGTGAGGGGAATGTAGCGTCGTTTCGGCGACGTG-TTATAGC-TCCTTGTC-
GAAT-ACACTGGCTGGGATTGAGGA-CCGCAGCACGCCC------TT-G-TGGCC-------
AGGGG----------------------TT---CG-CCCCATG-T-AACGTGCTTAGGAT-
GTTGGCATAATGGCTTTAAGCGACCCGTCTTGAAACACGGACCAAGGAGTCTAACATGCTTGCG
AGTGTTCGGGTGGAAAACCCTTGCGCATAATAAAAGTGAAA-GTTGGGAG-----CCTC-------
-----------CGCAA--GGGGGTGCACCGACGCCCGGCCCTGACG-----
TTCTCTGACGGTGCTGCGGTAGAGCAAGTATGTTGGG----
ACCCGAAAGATGGTGAACTATGCCTGAATAGGGCGAAGCCAGAGGAAACTCTG-
GTGGAGGCTCGTAGCGATTCTGACGTGCAAATCGATCGTCAAATTTGGGTTGATGTGAC--AT--
-CGAGGATAACGTAAGGGAAAAT-
AATGATATTACCTTACTATTAGTGTCGTCCAAATGCTCGTGCCAGAAGACTCGGTAAGGCGAGA
GACGCAAACGTTAGTCGTCTTGATCAGGCGTAAAGGGTTTGTAGGCAGCTTTAAGTATCCAA-
TTTAATGTTTACTATTAT--------------------------------AATATTTACA---------------
----

TGTTATAATTTTTATTAAAGGATCAATTAAAGCTAGAATCTAGAAGAGGTGA----
ATACAATGCTTGAG-CTTAGGAGTACTAACCGCAAACATCAAGTAGAGTGC-
TAAGGGCGAAGGC-
AGTTTTCCAAAACAGATTGACGCTGAGAAACGAAGGTGAGGATAGAAAAAAGGATTAGATACC
CTTGTATCCCTCACTGTCAACGATGAATGGTAGTTACTAGGGAGAA-----------------------
----
ATAACCTAGAGGCGATGTTAACACGTAAACCATTCCGCCTTGTGAGTACGACTGCAAAGTTGAA
AACAAAAAAATTAGTCGGTTTCGGAG--CAAACGAAGTGAAGC-ATGTTATTTAT--
GGTACTGGTGAGTTCGAGGCTGGTATCTCGAAGGACGGTCAGACCCGCGAGCACGCTCTGCTT
GCGTTCACCCTCGGTGTGCGTCAGCTGATCGTTGCTGTGAACAAAATGGACATGGCTATGGTTG
GT-GGCGCCATTGA--CCTCT-CCGTAACGAAGCTGAATC--GCGTTTG-------
AATACTCCAGTGGGTTGAGGAACGATTCAACGAAATTGTTAAGGAGACATCGAACTTCATTAAG
AAAGTCGGCTACAACCCCAAGACCGTACCTTTCGTCCCGATCTCCGGATGGCACGGTGACAAC
ATGCTTGAGGAGTCGAGCAAGTATGTTTGATTT-TGCCC-----TACAATGTGAGCG-----
TGTGCCGACGTGACATCT-TTCAGTATGACTTGGTACAAGGGCTGGACCAAGGAGACGAAGG--
----------GCGGTGTCGTT-
AAGGGAAAGACTCTTCTCGATGCCATTGATGCCATCGAGCCCCCTGTCCGCCCAGAGAACAAA
CCCTTGCGACTTCCTCTCCAGGATGTGATTTCCGTTGGATCGTTATCCGCTCCGGTACTCGAATT
CTTGGAGGAATGGGGCTTGGAGAGCCTCGAGGAAAATGCACA---AGCCGCAA------
ATTTGACCAAAGTTTTCGTGAATGGCGTATGGCTCGGAGTACACCGAGACCCGCACACCCTTGT
CAACACTCTTCGAACGCTCAGGAGGAAGG---
AGGATCTCAACGCTGAGGTTTCCATTGTTCGTGACATACGCGAAAAGGAAGTTCGCATCTATAC
GGACGCAGGACGTGTTCTTCGACCGCTTTTCATCGTCGAGGACAATAGATTAAAGCTGAGGCGT
CAGCACCTGCGCTGGCTCCAGGCGAAGTGGAGGATT-------
GAGGAGAGGCCCAAAGACAGGGTCTT------------
GGATGAGGGCGAAGAATCAGCAGGCGAGACAATAACTGAGGTAGAAGACGATGGGAAGCCAA
TAATACGGGAAGGGGTTCGAATGAAGAAGAAGAGGAAACCTGTCACCTGGAAGACATTGATTA

GCGACGGCGTTATTGAATACCTTGATGCAGAGGAGGAGGAAACGGTCATGATCTGCATGACAC
CGGAAGATCTGGTCGAATGTCGGTTGATGAGGCAAGGCCTGGATCCTCG---
AGCAGACACGGAGTTCGATGCATCTACCCGTCTCAAGCCTGTACCAACT------
GCTCATAACTGGACACATTGCGAGATTCATCCTAGCATGATTCTAGGTATATGTGCAAGTATCAT
TCCGTTCCC
>Phylloporia_osmanthi_Yuan_5655

TCCCCTAGTAACTGCGAGTGAAGCGGGAAGAGCTCAAATTTGTAATCT-G-ACG-GTC--
TCTGGCCGTCCGAGTTGTAATCTGTAGAGGCGTTGT-CCGCGTCG-
GCCCGTGTACAAGTCTCCTGGGATGGAGCGTCATAGAGGGTGAGAATCCCG-
TCCATGACATGG-AC-CACCGATGC-
TTTGTGAGGTGCCCTCGAAGAGTCGCGTTGTTTGGGAATGCAGCGCAAAATGGGTGGTAAATTC
CATCTAAGGCTAAATATTGGCGAGAGACCGATAGCGAACAAGTACCGTGAGGGAAAGATGAAA
AGCACTTTGAAAAGAGAGTTAAACAGTACGTGAAATTGTTGAAAGGGAAACACTTGAAGTCAGT
CGTGTCCCATGGCACTCAGCCTTGC----TTCGGCTTGGTGTACTTGCCTT-G---
GGATGGGTCAACATCAA-TTTT-GGC-
TGGTGGACAAGGGTGAGGGGAAGGTAGCGTCGTTTCGGCGACGTG-TTATAGC-CTCTTGTT-
GAAT-ACATCGGCTGGGATTGAGGA-CTGCAGCACGCCC------TT-G-TGGTC-------
AGAGG----------------------TT---CG-CCTCATG-T-AACGTGCTTAGGAT-
GTTGGCATAATGGCTTTAAGTGACCCGTCTTGAAACACGGACCAAGGAGTCTAACATGCTTGCG
AGTGTTCGGGTGGAAAACCCTTGCGCATAATGAAAGTGAAA-GTTGGGAG-----CCTC-------
-----------CGTAA-TGGGGGTGCACTGACGCCCGGCCCTGATG-----
TTCTCAGACGGTGCTGCGGTAGAGCAAGTATGTTGGG----
ACCCGAAAGATGGTGAACTATGCCTGAATAGGGTGAAGCCAGAGGAAACTCTG-

GTGGAGGCTCGTAGCGATTCTGACGTGCAAATCGATCGTCAAATTTGGGGCTCTGGATT--AA--
-ATTTAATATGTAAGGCAACAAT-AATGATGAAACCTTACTATTAGTGTCGTCCAAA-
ACTGGTGCCAGAAGACTCGGTAAGGCCAGAGACGCAAACGTTAGTCGTCATTATCAGGCGTAA
AGGGTTCGTAGGCAGCTTTGAAATTTCTTTCTTTATAAGTAGCACTCT---------------------
----

AATTTAGAGTTAAGGAAAGAAGCAATTAAAGCTAGAATCATAAAGAGGTCATATTATATAATTTC
AAGA--
TAAGGGGTTAAATCTGAAAACACTTGAAGGAATTTTAAAGGGCGAAGGCTTATTTTCCATAAAT
GATTGACGCTGAGGAACGAAAGGGAGGAGCGGAACCAGGATTAGATACCCATTTACCCCTCTC
TGCTAACGATGAATGGTAGTTAACCTGTTATA------------------------------------
TAAAATAGGAAGCAAAGCTAACGCGATAACCATTCCGCCATGCGAGTACGACTGCAAAGTTGA
AAGCAAAAAAATTAGTCGGTTTCGAAG--CAAACGAAGTGAAGC-ATGTTATTTAATT--------
----

>Phylloporia_pendula_Cui_13691
ACGAATTTTGA-------A---GCGGGG------------------------------CTTGTTGCTG--
GTGCG---------------AAAACACGTACA-TGTG-CACGGC----------------------------
---TCT--GTG-----TTCGATCC--A--CTC-----------------AA-CCCC--TGTGCA-
CTTTTGCA--AAG-------------------
-----------------------------------CCGAGTAGG----------------C------
TACACTTC---------------------------TTTGGA-------------
GTGTCTAGTAACCAGTAAGAGGGGAGG--
GTGC--
--GCAAGCATC--
------------------------AGTAGT-CTGAACGTGA---------------------------------
AAAGGCT---TCACTGTTTGAA--GTGTCAAGTAGTA-----------------------------------
--GTGAGTAA-
TGCGAACACGCGTGCAAGCGCGAGTA---------------GCGTGAA-------AGCGAGAAG---

----------------TGCCTTCATTC------------AGAGA-GGTGTAGTTGAAGGC-TTTGA-----
-TTTTT----AT-------------CA---ACCTTTTAG-ATGTCTTGTGAAT--G-----T-GCT-GC-C-
CC-TT----GTGGGTGAAAT-------
GAATACAACTTTCAACAACGGATCTCTTGGCTCTCGCATCGATGAAGAACGCAGCGAAATGCGA
TAAGTAATGTGAATTGCAGAATTCAGTGAATCATCGAATCTTTGAACGCACCTTGCGCCCTTTGG
-TATTCCGAAGGGCATGCCTGTTTGAGTGTCATTTTCATCTCAAGCTTCTCG-----AGC-
CCTTTTG--------------TTA---GGACT-----CGG-GCAGTTTGGAC-TTGGAGG-C--------
CTTGCTGAT-GAGC---------------GTTCT--GCGCGGGTCGGCTCCTCTGAAAAGCATTAGC-
TAGACTTTGG----------------T-TC---GCA-TGG-----------ATGGTGTGATAGTA-------
---------TGTG-CTCAC----TGAAAG-TGCTTG-CCTAA-GAGGTCTGC-TTC-----
TAGTCGTCTT------------------CGGAC-AA--------GG-TCC---TTTGT-GAC----T-
TTTACTTGACAA-
TTGACCTCATCCCCTAGTAACTGCGAGTGAAGCGGGAAGAGCTCAAATTTGTAATCT-G-GTG-
GTC--TCTGGCCATCCGAGTTGTAATCTGTAGAGGCGTTGT-CTGCGTCG-
GCCCGTGTACAAGTCTCCTGGAATGGAGCGTCATAGAGGGTGAGAATCCCG-
TCCATGGCACGG-AC-CATCGATGC-
TCTGTGAGGCGCCCTCGAAGAGTCGCGTTGTTTGGGAATGCAGCGCAAAATGGGTGGTAAATTC
CATCTAAGGCTAAATATTGGCGAGAGACCGATAGCGAACAAGTACCGTGAGGGAAAGATGAAA
AGTACTTTGGAAAGAGAGTTAAATAGTATGTGAAATTGTTGAAAGGGAAACGCTTGAAGTCAGT
CGCGTTCTGGGGCACTCAGCCTTGC----TTTGGCTTGGTGTACTTGCCTT-G---
GGATGGGTCAACATCAA-TTTT-GGC-
GGGTGGATAAGGGTGAGAGGAACGTAGCGTCATTTCGGTGGCGTGTTTATAGC-CTCTCGTC-
GAAT-GCACCAGTTGGGATTGAGGA-CTGCAGCACGCCC------TT-G-TGGTC-------
GGAGG----------------------TT---CG-CCTCACG-T-TACGTGCTTAAGAT-
GTTGGCATAATGGCTTTAAGCGACCCGTCTTGAAACACGGACCAAGGAGTCTAACATGCTTGCG
AGTGTTCGGGTGGGAAACCCTTGCGCGTAATGAAAGTGAAA-GTTGAGAG-----CCTT-------
-----------CGAAG-AGGGGGTGCATCGACGCCCGGCCCTGACG-----
TTCTCTGACGGTGCCGCGGTAGAGCAAGCATGTTGGG----
ACCCGAAAGATGGTGAACTATGCCTGAATAGGGTGAAGCCAGAGGAAACTCTG-
GTGGAGGCTCGTAGCGATTCTGACGTGCAAATCGATCGTCGAATTTGGGTTGATGTGAC--AT--
-CGAGGATAACGTAAGGGAAAAT-
AATGATATTACCTTACTATTAGTGTCGTCCAAATGCTCGTGCCAGAAGACTCGGTAAAGCGAGA
GACGCAAACGTTAGTCGTCTTGATCAGGCGTAAAGGGTTTGTAGGCAGCTTTAAGTATTCAA-
TTTAATGTTTACTATTAT--------------------------------AATATTTACA--------------
----

AGTTATAATATTTATTAGAGGATCAATTAAAGCTAGAATCTAGAAGAGGTGA----
ATACAATGCTTGAG-CTTAGGAGTACTAACCGCAAACATCAAGTAGAGTGC-
TAAGGGCGAAGGC-
AGTTTTCCAATAAAGATTGACGCTGAGAAACGAAGGTGAGGATAGAAAAAAGGATTAGATACC
CTTGTATCCCTCACTGTCAACGATGAATGGTAGTTACTAGGGAGAA----------------------
----
ATGACCTAGAGGCGATGTTAACACGTAAACCATTCCGCCTTGTGAGTACAACTGCAAAGTTGAA
AACAAAAAAATTAGTCGGTTTCGGAG--CAAACGAAGTGAAGC-

ATGTTATTTAATTGGTACTGGCGAGTTCGAGGCTGGTATCTCGAAGGACGGTCAGACTCGCGAG
CATGCTCTGCTTGCGTTCACCCTCGGTGTGCGTCAGCTGATCGTCGCCGTGAACAAGATGGACA
CGGCCAAGGTTGGTGGGCGCTATTGA--CCTCT-CCGTAACGAAGCTGAACC--CCGTTCG----
---
AATAATTCAGTGGTCTGAGGAACGATTTAACGAAATCGTCAAAGAGACGTCGAACTTTATCAAG
AAGGTCGGTTACAACCCGAAGGCCGTACCTTTCGTCCCGATCTCCGGGTGGCACGGTGACAAC
ATGCTTGAGGAGTCGACTAAGTACGTTTGATTT-TGTCCTGCTTTACAAGATAGGCG-----
TGATCTGACGTGATGTCT-TTCAGTATGCCTTGGTACAAGGGCTGGTCCAAGGAGACGAAGG---
---------GTGGTGTCTTT-
AAAGGAAAGACTCTTCTCGACGCCATTGATGCCATCGAAGCCCCCGTCCGTCCGGAGAACAAG
CCCTTGCGACTTCCCCTGCAGGACGTGATTTCCGTTGGATCATTATCCGCTCCGGTGCTCGAATT
CTTGGAGGAATGGGGCTTGGAGAGCCTAGAGGAAAATGCGCA---AGCTGCAA------
ATCTGACCAAAGTCTTCGTGAATGGGGTATGGCTCGGAGTGCACCGAGACCCGCACACTCTTGT
AAACACTCTTCGAACGCTCAGGAGAAAAG---
AGGATCTCAACGCCGAGGTTTCTATTGTTCGTGACATACGCGAAAAGGAAGTTCGCATCTACAC
GGACGCAGGACGCGTCCTTCGACCGCTTTTCATTGTCGAGGACAATAGATTGAAGCTGAGACGT
CAACACCTGCGCTGGCTTCAGGAGAAGTGGAGGATT-------
GAGGAGAGGCCCAAGGACAGAGTCCT------------
GGATGAAGGCGAAGAATCAGCAGGAGAGACAATTACTGAGCTAGAAGGCGATGGGAAACCAA
TAATACGGGAAGGGGTCCGAATGAAGAGAAAGAGGAAACCTGTCACCTGGAAGACATTAATTG
GCGACGGCGTTATTGAATACCTTGATGCAGAGGAGGAGGAAACAGTCATGATCTGCATGACAC
CAGAAGATCTGGTCGAGTCTCGGTTAATGAGGCAAGGCTTGGATCCCCG---
AGCAGACGCGGAGTTGGATGCGTCCACGCGTCTCAAGCCTGTACCAACT------
GCTCATAACTGGACACATTGCGAGATTCATCCCAGTATGATTCTAGGCATATGTGCCAGTATCAT
TCCGTTCCC
>Phylloporia_pendula_Cui_13876
ACGAATTTTGA-------A---GCGGGG------------------------------CTTGTTGCTG--
GTGCG---------------AAAACACGTACA-TGTG-CACGGC----------------------------
---TCT--GTG-----TTCGATCC--A--CTC-----------------AA-CCCC--TGTGCA-
CTTTTGCA--AAG-------------------
-----------------------------------CCGAGTAGG----------------C------
TACACTTC----------------------------TTTGGA-------------
GTGTCTAGTAATCAGTAAGAGGGGAGG---------------------------------------
GTGC---
---GCAAGCATC--
-------------------------AGTAGT-CTGAACGTGA---------------------------------
AAAGGCT---TCACTGTTTGAA--GTGTCAAGTAGTA-----------------------------------
---GTGAGTAA-
TGCGAACACGCGTGCAAGCGCGAGTA---------------GCGTGAA-------AGCGAGAAG---
-----------------TGCCTTCATTC------------AGAGA-GGTGTAGTTGAAGGC-TTTGA-----
-TTTTT----AT-------------CA---ACCTTTTAG-ATGTCTTGTGAAT--G-----T-GCT-GC-C-
CC-TT----GTGGGTGAAAT-------
GAATACAACTTTCAACAACGGATCTCTTGGCTCTCGCATCGATGAAGAACGCAGCGAAATGCGA
TAAGTAATGTGAATTGCAGAATTCAGTGAATCATCGAATCTTTGAACGCACCTTGCGCCCTTTGG

-TATTCCGAAGGGCATGCCTGTTTGAGTGTCATTTTCATCTCAAGCTTCTCG-----AGC-
CCTTTTG--------------TTA---GGACT-----CGG-GCAGTTTGGAC-TTGGAGG-C--------
CTTGCTGAT-GAGC---------------GTTCT--GCGCGGGTCGGCTCCTCTGAAAAGCATTAGC-
TAGACTTTGG-----------------T-TC---GCA-TGG-----------ATGGTGTGATAGTA-------
---------TGTG-CTCAC----TGAAAG-TGCTTG-CCTAA-GAGGTCTGC-TTC-----
TAGTCGTCTT------------------CGGAC-AA--------GG-TCC---TTTGT-GAC----T-
TTTACTTGACAATTTGACCTCATCCCCTAGTAACTGCGAGTGAAGCGGGAAGAGCTCAAATTTGT
AATCT-G-GTG-GTC--TCTGGCCATCCGAGTTGTAATCTGTAGAGGCGTTGT-CTGCGTCG-
GCCCGTGTACAAGTCTCCTGGAATGGAGCGTCATAGAGGGTGAGAATCCCG-
TCCATGGCACGG-AC-CGTCGATGC-
TCTGTGAGGCGCCCTCGAAGAGTCGCGTTGTTTGGGAATGCAGCGCAAAATGGGTGGTAAATTC
CATCTAAGGCTAAATATTGGCGAGAGACCGATAGCGAACAAGTACCGTGAGGGAAAGATGAAA
AGTACTTTGGAAAGAGAGTTAAATAGTATGTGAAATTGTTGAAAGGGAAACGCTTGAAGTCAGT
CGCGTTCTGGGGCACTCAGCCTTGC----TTTGGCTTGGTGTACTTGCCTT-G---
GGATGGGTCAACATCAA-TTTT-GGC-
GGGTGGATAAGGGTGAGAGGAACGTAGCGTCATTTCGGTGGCGTGTTTATAGC-CTCTCGTC-
GAAT-GCACCAGCTGGGATTGAGGA-CTGCAGCACGCCC------TT-G-TGGTC-------
GGAGG----------------------TT---CG-CCTCACG-T-TACGTGCTTAAGAT-
GTTGGCATAATGGCTTTAAGCGACCCGTCTTGAAACACGGACCAAGGAGTCTAACATGCTTGCG
AGTGTTCGGGTGGGAAACCCTTGCGCGTAATGAAAGTGAAA-GTTGAGAG-----CCTT-------
-----------CGAAG-AGGGGGTGCATCGACGCCCGGCCCTGACG-----
TTCTCTGAGGGTGCCGCGGTAGAGCAAGCATGTTGGG----
ACCCGAAAGATGGTGAACTATGCCTGAATAGGGTGAAGCCAGAGGAAACTCTG-
GTGGAGGCTCGTAGCGATTCTGACGTGCAAATCGATCGTCGAATTTGGGTTGATGTGAC--AT--
-CGAGGATAACGTAAGGGAAAAT-
AATGATATTACCTTACTATTAGTGTCGTCCAAATGCTCGTGCCAGAAGACTCGGTAAAGCGAGA
GACGCAAACGTTAGTCGTCTTGATCAGGCGTAAAGGGTTTGTAGGCAGCTTTAAGTATTCAA-
TTTAATGTTTACTATTAT--------------------------------AATATTTACA---------------
----

AGTTATAATATTTATTAGAGGATCAATTAAAGCTAGAATCTAGAAGAGGTGA----
ATACAATGCTTGAG-CTTAGGAGTACTAACCGCAAACATCAAGTAGAGTGC-
TAAGGGCGAAGGC-
AGTTTTCCAATAAAGATTGACGCTGAGAAACGAAGGTGAGGATAGAAAAAAGGATTAGATACC
CTTGTATCCCTCACTGTCAACGATGAATGGTAGTTACTAGGGAGAA-----------------------
----
ATGACCTAGAGGCGATGTTAACACGTAAACCATTCCGCCTTGTGAGTACAACTGCAAAGTTGAA
AACAAAAAAATTAGTCGGTTTCGGAG--CAAACGAAGTGAAGC-ATGTTATTTAATT----------
----
CAGACTCGCGAGCATGCTCTGCTTGCGTTCACCCTCGGTGTGCGTCAGCTGATCGTCGCCGTGA
ACAAGATGGACACGGCCAAGGTTGGTGGGCGCTATTGA--CCTCT-
CCGTAACGAAGCTGAACC--CCGTTCG-------
AATAATTCAGTGGTCTGAGGAACGATTTAACGAAATCGTCAAAGAGACGTCGAACTTTATCAAG
AAGGTCGGTTACAACCCGAAGGCCGTACCTTTCGTCCCGATCTCCGGGTGGCACGGTGACAAC

ATGCTTGAGGAGTCGACTAAGTACGTTTGATTT-TGTCCTGCTTTACAAGATAGGCG-----
TGATCTGACGTGATGTCT-TTCAGTATGCCTTGGTACAAGGGCTGGTCCAAGGAGACGAAGG---
---------GTGGTGTCTTT-AAAGGAAAGACTCTTCTCGACGCCATT------------------------
----
ATTTCCGTTGGATCATTATCCGCTCCGGTGCTCGAATTCTTGGAGGAATGGGGCTTGGAGAGCC
TAGAGGAAAATGCGCA---AGCTGCAA------
ATCTGACCAAAGTCTTCGTGAATGGGGTATGGCTCGGAGTGCACCGAGACCCGCACACTCTTGT
AAACACTCTTCGAACGCTCAGGAGAAAAG---
AGGATCTCAACGCCGAGGTTTCTATTGTTCGTGACATACGCGAAAAGGAAGTTCGCATCTACAC
GGACGCAGGACGCGTCCTTCGACCGCTTTTCATTGTCGAGGACAATAGATTGAAGCTGAGACGT
CAACACCTGCGCTGGCTTCAGGAGAAGTGGAGGATT-------
GAGGAGAGGCCCAAGGACAGAGTCCT------------
GGATGAAGGCGAAGAATCAGCAGGAGAGACAATTACTGAGCTAGAAGGCGATGGGAAACCAA
TAATACGGGAAGGGGTCCGAATGAAGAGAAAGAGGAAACCTGTCACCTGGAAGACATTAATTG
GCGACGGCGTTATTGAATACCTTGATGCAGAGGAGGAGGAAACAGTCATGATCTGCATGACAC
CAGAAGATCTGGTCGAGTCTCGGTTAATGAGGCAAGGCTTGGATCCCCG---AGCAGAC------
----

>Phylloporia_perangusta_Dai_18139
ACGAGTTTTGA-----A-A---GCGGAG------------------------------TCTGCTGCTG--
GTGCG---------------GAAACG--TACA-TGTG-CACGGC-----------------------------
---TCT--GTG-----CTCGATCC--A--CTC-----------------AA-CCCC--TGTGCA-
CCTTTGCG--AAG-------------------
------------------------------------CGAGTAGG----------------C------TTCACCC-
---------------------------TTTGAAGG----------
TGGGACCAGTAGTGAGTAAGGCGAGC---
GTGT---
--GCGA--------

---GCATCAGTA------
---------GCCTGAA-------AGCGAGCG---------------------CCCTTCATTT-----------
AGAGAG-GGTGCGGTCGAAAGC-TTTGA------TCTTT----AT-------------CA---
ACCTTTTGT-ATGTCATGTGAAT--G-----T-GTT-GC-T-CC-TC----GTGGGCGAAAT-------
AAATACAACTTTCAACAACGGATCTCTTGGCTCTCGCATCGATGAAGAACGCAGCGAAATGCGA
TAAGTAATGTGAATTGCAGAATTCAGTGAATCATCGAATCTTTGAACGCACCTTGCGCCCTTTGG
-TATTCCGAAGGGCATGCCTGTTTGAGTGTCACGTTTATCTCAAACCTCC-------AAG-
CCTTTTG--------------TTA----GGTTT----CGG-ACGGTTTGGAC-TTGGAGG-C-C------
TTTGCTGGT-GTGC---------------GCTCT--GCGTGTGTCGGCTCCTCTGAAAAGCATTAGC-
TGGACTTTGG----------------T-TT---GCG-TGC-----------ATGGTGTGATAGTAA------
---------TGTG-TTCAC----CGGAAG-CGCTTG-CCTGA-CGGGTCTGC-TCC-----
TAATCGTCTT------------------CGGAC-GA--------GG-TCC---TTCGT-GAC----C-
TTCGCTTGACCGTTTGACCTCATCCCCTAGTAACTGCGAGTGAAGCGGGAAGAGCTCAAATTTG
TAATCT-G-GCG-GTC--TCTGGCCGTCCGAGTTGTAATCTGTAGAGGCGTCGT-CCGCGTCG-

GCCCGTGTACAAGTCTCCTGGGATGGAGCTTCATAGAGGGTGAGAATCCCG-
TCCATGACACGG-AC-CACCGATGC-
TCTGTGAGGTGCCCTCGAAGAGTCGCGTTGTTTGGGAATGCAGCGCAAAATGGGTGGTAAATTC
CATCTAAGGCTAAATATTGGCGAGAGACCGATAGCGAACAAGTACCGTGAGGGAAAGATGAAA
AGCACTTTGGAAAGAGAGTTAAACAGTACGTGAAATTGTTGAAAGGGAAACGCTTGAAGTCAG
TCGTGTCCCGTGACATTCAGCCTTGC----TTCGGCTTGGTGTACTTGCCTT-G---
GGACGGGTCAACATCAA-TTTT-GGC-
CGGTGGATAAGGGTGAGGGGAATGTAGCGTCGTTTCGGCGACGTG-TTATAGC-CTCTTGTC-
GAAT-ACGCCGGTTGGGATTGAGGA-CTGCAGCACGCCC------TT-G-TGGCC-------
GGGGG----------------------TT---CG-CCTCATG-T-AACGTGCTTAGGAT-
GTTGGCATAATGGCTTTAAGCGACCCGTCTTGAAACACGGACCAAGGAGTCTAACATGCTTGCG
AGTGTTCGGGTGGAAAACCCTTGCGCATAATAAAAGTGAAA-GTTGGGAG-----CCTC-------
-----------CGTAA-GGGGGGTGCACCGACGCCCGGCCCTGACG-----
TTCTCTGACGGTGCTGCGGTAGAGCAAGTATGTTGGG----
ACCCGAAAGATGGTGAACTATGCCTGAATAGGGTGAAGCCAGAGGAAACTCTG-
GTGGAGGCTCGTAGCGATTCTGACGTGCAAATCGATCGTCAAATTTGGGCCCAGCAGTC--AA-
--GTGTGATAACGTAAGGGAAGAT-AATGACATTACCTTACTATTAGTGTTGTCCAAAT-
CTGGTGCCAGAAGACTCGGTAAGGCCAGAGACGCAAACGTTAGTCGTCTTAATCAGGCGTAAA
GGGTGTGTAGGCAGCTTTTAACTTTATT-CTTTTTCTAAATCCTCATTAAT-CGAGGAA---
GAAATATAGACTAA--TATTATTATTA---

AATAAATAATAAAAATATTAAAGAAGGGAATGAACTAATAAAAGCTAGAATCAAATAGAGGATA
TATTAAATAATATTTAGA--GTAGGGGCGATATCCTTAAATACTAAATGGAATAT-
TAAAGGCGAAAGCTTTTTATCTACTAATGATTGACGCTGAAACACGAAGGTGAGTATAGGAAAT
AGGATTAGATACCCAGATACCACTCACTGTCAACGATGAATGGTGGTTACTAGTAAAAAA-----
----
ATAAATTAGTAGCTATGTTAACGCGATAACCATTCCGCCATGTGAGTACAACTGCAAAGTTGAA
AACAAAAAAATTAGTCGGTCTCGAAG--CAAACGAAGTGAAGC-
ATGTTATTTAATTGGTACTGGTGAGTTCGAGGCTGGTATCTCGAAGGACGGTCAGACTCGCGAG
CACGCTCTGCTTGCGTTCACCCTCGGTGTGCGTCAGCTGATCGTTGCTGTGAACAAGATGGATA
CGGCTAAGGTTGGT-GGTGCCATTGA--CCTCT-CCGTAACGAAGCTGAATC--GCGTTTG-----
--
AATACTCCAGTGGTCTGATGAACGATTCAACGAAATTGTTAAGGAGACATCGAACTTCATTAAG
AAAGTCGGCTACAACCCCAAGACCGTACCTTTTGTCCCGATCTCCGGATGGCACGGTGACAACA
TGCTTGAGGAGTCGAGCAAGTATGTTTGATTT-TGCCC-----TGCAATGTAAGCG-----
TGCGCCGACGTGACATCT-TTCAGTATGACTTGGTACAAGGGCTGGACCAAGGAGACGAAGG--
----------GCGGTGTCGTT-
AAGGGAAAGACTCTTCTCGATGCCATTGATGCCATCGAGCCCCCTGTCCGCCCAGAGAACAAA
CCCTTGCGACTCCCTCTCCAGGATGTGATTTCCGTTGGATCGTTGTCCGCTCCGGTACTCGAATT
CTTGGAGGAATGGGGCTTGGAGAGCCTCGAGGAAAATGCACA---AGCTGCAA------
ATTTGACCAAAGTTTTCGTGAATGGCGTATGGCTCGGAGTACACCGAGACCCGCACACCCTTGT
CAACACTCTTCGAACGCTCAGGAGGAAGG---
AGGATCTCAACGCTGAGGTTTCCATTGTTCGTGACATACGCGAAAAGGAAGTTCGCATCTATAC
GGACGCAGGACGTGTTCTTCGACCACTTTTCATCGTCGAGGACAATAGATTAAAGCTGAGGCGT

CAGCACCTGCGCTGGCTTCAGGCGAAGTGGAGGATT-------
GAGGAGAGGCCCAAAGACAGGGTCTT------------
GGATGAGGGCGAAGAATCAGCAGGCGAGACAATAACTGAGGTAGAAGACGATGGGAAGCCAA
TAATACGGGAAGGGGTTCGAATGAAGAAGAAGAGGAAACCTGTCACCTGGAAGACATTGATTA
GAGACGGCGTTATTGAATACCTTGATGCAGAGGAAGAGGAAACGGTCATGATCTGCATGACAC
CAGAAGATCTGGTCGAATCTCGGTTGATGAGGCAAGGCCTGGATCCTCG---
AGCAGACACGGAGTTCGATGCATCTACCCGTCTCAAGCCTGTACCAACT------
GCTCATAACTGGACACATTGCGAGATTCATCCTAGCATGATTCTAGGTATATGTGCAAGTATCAT
TCCGTTCCC
>Phylloporia_pseudopectinata_Cui_13749
AAGAGTCTTGA-----A-A---GCAGAG------------------------------TCTGCTGCTG--
GTGCG---------------GAAACG--CGCA-TGTG-CACGGC----------------------------
---TTT--GCG-----CTCAGTCC--A--CTC----------------AAA-CCCC--
TGTGCACCCTTTGCA---AGG-----------
---CGAGTAGG----------------C------
TTCGCTC----------------------------TTTGAA-------------
GCGTTCAGTAGCGAGTAAGGTGAAG--
GTGC---
--GCGAGCATT--
------------------------AGTAGC-CCGAAAGCGA---------------------------------
AAGGGCT---TCAATCTTTTGAA-GCGTTCAGTAGTAAGTAAGG---TGAAGGCGCAAGCG-----
-CTGGTAACCTGAAAGCGAAAG--GGCTTCATTGACTGTTTGAAGGGATC-AGTAGTGAGTAA-
GGTG-------------AATGCAGTA---------------GCCTGAA-------AGCGAGCG--------
-------------TCCTTCATTC-----------CGGGAG-GGCGTCGTCGAAAGC-TTTGA------
TCTTT----AT-------------CA---ACCTTTCGT-ATGTTTTGTGAAT--G-----C-GTT-GC-T-
CC-TC----GTGGGCGAAAT-------
CAATACAACTTTCAACAACGGATCTCTTGGCTCTCGCATCGATGAAGAACGCAGCGAAATGCGA
TAAGTAATGTGAATTGCAGAATTCAGTGAATCATCGAATCTTTGAACGCACCTTGCGCCCTTTGG
-TACTCCGAAGGGCATGCCTGTTTGAGTGTCATGTTAATCTCAGACCTC--------AAG-
TCTTTCG--------------TTA----GGCT-----TTG-ACGGTTTGGAG-TTGGAGG-C---------
-TGCTGGC-CT------------------------GTGCGGGTCGGCTCCTCTGAAAAGCATTAGC-
TGGACTTTGG----------------T-TC---GCA-TGG-----------ACGGTGTGATAGTAA-----
----------AGTG-TTCAC----CGGAAG-TGCCTG-TCTAA-TGGGTCCGC-TTC-----
TAATCGTCTT------------------CGGAC-AA--------GC--------------------------
CTTGATCGTTTGACCTCA-
CCCCTAGTAACTGCGAGTGAAGCGGGAAGAGCTCAAATTTGTAATCT-G-GCG-GTC--
TCCGGCCGTCCGAGTTGTAATCTGTAGAGGCGTCGT-CCGCGTCG-
GCCCGTGTACAAGTCTCCTGGAATGGAGCGTCATAGAGGGTGAGAATCCCG-
TCCATGACACGG-AC-CACCGATGC-
TCTGTGAGGCGCCCTCGAAGAGTCGCGTTGTTTGGGAATGCAGCGCAAAATGGGTGGTAAATTC
CATCTAAGGCTAAATATTGGCGAGAGACCGATAGCGAACAAGTACCGTGAGGGAAAGATGAAA
AGCACTTTGAAAAGAGAGTTAAACAGTACGTGAAATTGTTGAAAGGGAAACGCTTGAAGTCAGT
CGCGTCCCGAGGCACTCAGCCTTGC----TTCGGCTTGGTGTACTTGCCTT-G---
AGACGGGTCAACGTCGA-TTTT-GGC-

TGGTGGAAAAAGGTAAGGGGAACGTGGCGTCGTTTCGACGACGTG-TTATAGC-CTCTTATT-
GAAT-ACATCAGCCGGGATCGAGGA-CCGCAGCACGCCC------TT-G-TGGCC-------
GGGGG----------------------TT---CG-CCCCACG-T-AACGTGCTTAGGAT-
GTTGGCATAATGGCTTTAAGCGACCCGTCTTGAAACACGGACCAAGGAGTCTAACATGCTTGCG
AGTGTTCGGGTGGAAAACCCTTGCGCGCAATGAAAGTGAAA-GTTGGGAG-----CCTC-------
-----------CGTGA--GGGGGCGCACCGACGCCTGGCCCTGAAG-----
TTCTCTGACGGTGCTGCGGTAGAGCAAGTATGTTGGG----
ACCCGAAAGATGGTGAACTATGCCTGAATAGGGCGAAGCCAGAGGAAACTCTG-
GTGGAGGCTCGTAGCGATTCTGACGTGCAAATCGATCGTCAAATTTGGG------------------
----

GGTACTGGTGAGTTCGAGGCTGGTATCTCGAAGGACGGTCAGACCCGCGAGCACGCTCTGCTT
GCGTTCACCCTCGGTGTACGTCAGCTGATTGTCGCTGTGAATAAAATGGACACGGCCAAGGTTG
GTGGGCGCTATTGA--CCTGT-CCGTAACGGAGCTGAACC--CCGTTCG-------
AATAATTCAGTGGTCTGAGGAACGATTTCATGAAATCGTTAAGGAGACGTCGAACTTTATCAAG
AAAGTCGGCTACAACGCGAAGGCCGTACCTTTCGTCCCGATCTCCGGATGGCACGGTGACAAC
ATGCTTGAGGAGTCGACTAAGTACGTTTGATTT-TGTCCTGATTTATAAGATAAGCG-----
CGAGCTGACGTGATACTT-CTCAGTATGACTTGGTACAAGGGCTGGACCAAAGAGACGAAGG--
----------GCGGTGTCGTT-
AAAGGAAAGACTCTTCTCGATGCCATTGATGCCATCGAACCCCCTGTCCGTCCTGAGAACAAGC
CCTTGCGACTTCCTCTGCAGGACGTGATTTCTGTTGGATCATTATCCGCCCCGGTGCTCGAATTC
TTGGAGGAATGGGGCTTGGAGAGCCTCGAGGAAAATGCACA---GGCCGCAA------
ATTTGACCAAAGTTTTCGTGAATGGGGTATGGCTCGGAGTGCACCGAGATCCGCACACCCTTGT
CAACACTCTTCGGACGCTCAGGAGAAAGG---
AGGATCTCAACGCTGAGGTTTCCATTGTTCGTGACATACGCGAAAAGGAAGTTCGCATCTACAC
GGACGCAGGACGCGTTCTTCGACCGCTTTTCATTGTCGACGACAATAGATTGAAGCTGAGACGT
CAGCACCTGCGCTGGCTTCAGGCGAAGTGGAGGATC-------
GAGGAGAGGCCCAAAGACAGAGTCCT------------
GGATGAAGGTGAAGAATCAGCAGGCGAGACAATAACTGAGCTAGAAGACGATGGGAAACCAA
TAATACGGGAAGGGGTTCGAATGAAGAAGAAGAGGAAACCGGTCACCTGGAAGACGTTGATTA
GCGACGGCGTTATTGAATACCTTGATGCAGAGGAGGAGGAAACAGTCATGATCTGCATGACTC
CAGAAGATCTGGTCGAATCTCGATTAATGAGGCAAGGCCTTGATCCTCG---
AGCGGACACGGAGTTCGATGCTTCTACACGTCTCAAGCCTGTACCGACT------
GCTCATAACTGGACACATTGCGAGATTCATCCCAGCATGATCCTAGGTATATGTGCAAGTATCAT
TCCGTTCCC
>Phylloporia_pseudopectinata_Cui_13746
AAGAGTCTTGA-----A-A---GCAGAG-------------------------------TCTGCTGCTG--

GTGCG---------------GAAACG--CGCA-TGTG-CACGGC----------------------------
---TTT--GCG-----CTCAGTCC--A--CTC----------------AAA-CCCC--
TGTGCACCCTTTGCA---AGG-----------
---CGAGTAGG----------------C------
TTCGCTC----------------------------TTTGAA-------------
GCGTTCAGTAGCGAGTAAGGTGAAG---
GTGC--
--GCGAGCATT--
-------------------------AGTAGC-CCGAAAGCGA----------------------------------
AAGGGCT---TCAATCTTTTGAA-GCGTTCAGTAGTAAGTAAGG---TGAAGGCGCAAGCG-----
-CTGGTAACCTGAAAGCGAAAG--GGCTTCATTGACTGTTTGAAGGGATC-AGTAGTGAGTAA-
GGTG-------------AATGCAGTA---------------GCCTGAA-------AGCGAGCG--------
-------------TCCTTCATTC-----------CGGGAG-GGCGTCGTCGAAAGC-TTTGA------
TCTTT----AT-------------CA---ACCTTTCGT-ATGTTTTGTGAAT--G-----C-GTT-GC-T-
CC-TC----GTGGGCGAAAT-------
CAATACAACTTTCAACAACGGATCTCTTGGCTCTCGCATCGATGAAGAACGCAGCGAAATGCGA
TAAGTAATGTGAATTGCAGAATTCAGTGAATCATCGAATCTTTGAACGCACCTTGCGCCCTTTGG
-TACTCCGAAGGGCATGCCTGTTTGAGTGTCATGTTAATCTCAGACCTC--------AAG-
TCTTTCG--------------TTA----GGCT-----TTG-ACGGTTTGGAG-TTGGAGG-C---------
-TGCTGGC-CT------------------------GTGCGGGTCGGCTCCTCTGAAAAGCATTAGC-
TGGACTTTGG----------------T-TC---GCA-TGG-----------ACGGTGTGATAGTAA-----
----------AGTG-TTCAC----CGGAAG-TGCCTG-TCTAA-TGGGTCCGC-TTC-----
TAATCGTCTT------------------CGGAC-AA--------GC--------------------------
CTTGATCGTTTGACCTCA-
CCCCTAGTAACTGCGAGTGAAGCGGGAAGAGCTCAAATTTGTAATCT-G-GCG-GTC--
TCCGGCCGTCCGAGTTGTAATCTGTAGAGGCGTCGT-CCGCGTCG-
GCCCGTGTACAAGTCTCCTGGAATGGAGCGTCATAGAGGGTGAGAATCCCG-
TCCATGACACGG-AC-CACCGATGC-
TCTGTGAGGCGCCCTCGAAGAGTCGCGTTGTTTGGGAATGCAGCGCAAAATGGGTGGTAAATTC
CATCTAAGGCTAAATATTGGCGAGAGACCGATAGCGAACAAGTACCGTGAGGGAAAGATGAAA
AGCACTTTGAAAAGAGAGTTAAACAGTACGTGAAATTGTTGAAAGGGAAACGCTTGAAGTCAGT
CGCGTCCCGAGGCACTCAGCCTTGC----TTCGGCTTGGTGTACTTGCCTT-G---
AGACGGGTCAACGTCGA-TTTT-GGC-
TGGTGGAAAAAGGTAAGGGGAACGTGGCGTCGTTTCGACGACGTG-TTATAGC-CTCTTATT-
GAAT-ACATCAGCCGGGATCGAGGA-CCGCAGCACGCCC------TT-G-TGGCC-------
GGGGG----------------------TT---CG-CCCCACG-T-AACGTGCTTAGGAT-
GTTGGCATAATGGCTTTAAGCGACCCGTCTTGAAACACGGACCAAGGAGTCTAACATGCTTGCG
AGTGTTCGGGTGGAAAACCCTTGCGCGCAATGAAAGTGAAA-GTTGGGAG-----CCTC-------
-----------CGTGA--GGGGGCGCACCGACGCCTGGCCCTGAAG-----
TTCTCTGACGGTGCTGCGGTAGAGCAAGTATGTTGGG----
ACCCGAAAGATGGTGAACTATGCCTGAATAGGGCGAAGCCAGAGGAAACTCTG-
GTGGAGGCTCGTAGCGATTCTGACGTGCAAATCGATCGTCAAATTTGGG------------------
----

GGTACTGGTGAGTTCGAGGCTGGTATCTCGAAGGACGGTCAGACCCGCGAGCACGCTCTGCTT
GCGTTCACCCTCGGTGTACGTCAGCTGATTGTCGCTGTGAATAAAATGGACACGGCCAAGGTTG
GTGGGCGCTATTGA--CCTGT-CCGTAACGGAGCTGAACC--CCGTTCG-------
AATAATTCAGTGGTCTGAGGAACGATTTCATGAAATCGTTAAGGAGACGTCGAACTTTATCAAG
AAAGTCGGCTACAACGCGAAGGCCGTACCTTTCGTCCCGATCTCCGGATGGCACGGTGACAAC
ATGCTTGAGGAGTCGACTAAGTACGTTTGATTT-TGTCCTGATTTATAAGATAAGCG-----
CGAGCTGACGTGATACTT-CTCAGTATGACTTGGTACAAGGGCTGGACCAAAGAGACGAAGG--
----------GCGGTGTCGTT-
AAAGGAAAGACTCTTCTCGATGCCATTGATGCCATCGAACCCCCTGTCCGTCCTGAGAACAAGC
CCTTGCGACTTCCTCTGCAGGACGTGATTTCTGTTGGATCATTATCCGCCCCGGTGCTCGAATTC
TTGGAGGAATGGGGCTTGGAGAGCCTCGAGGAAAATGCACA---GGCCGCAA------
ATTTGACCAAAGTTTTCGTGAATGGGGTATGGCTCGGAGTGCACCGAGATCCGCACACCCTTGT
CAACACTCTTCGGACGCTCAGGAGAAAGG---
AGGATCTCAACGCTGAGGTTTCCATTGTTCGTGACATACGCGAAAAGGAAGTTCGCATCTACAC
GGACGCAGGACGCGTTCTTCGACCGCTTTTCATTGTCGACGACAATAGATTGAAGCTGAGACGT
CAGCACCTGCGCTGGCTTCAGGCGAAGTGGAGGATC-------
GAGGAGAGGCCCAAAGACAGAGTCCT------------
GGATGAAGGTGAAGAATCAGCAGGCGAGACAATAACTGAGCTAGAAGACGATGGGAAACCAA
TAATACGGGAAGGGGTTCGAATGAAGAAGAAGAGGAAACCGGTCACCTGGAAGACGTTGATTA
GCGACGGCGTTATTGAATACCTTGATGCAGAGGAGGAGGAAACAGTCATGATCTGCATGACTC
CAGAAGATCTGGTCGAATCTCGATTAATGAGGCAAGGCCTTGATCCTCG---AGCGGAC------
----

>Phylloporia_pulla_LWZ_20190428-2

TAACTGCGAGTGAAGCGGGAAGAGCTCAAATTTGTAATCT-G-GCG-GTC--
TCTGGCCGTCCGAGTTGTAATCTGTAGAGGCGTCGT-CCGCGTCG-
GCCCGTGTACAAGTCTCCTGGGATGGAGCGTCATAGAGGGTGAGAATCCCG-
TCCATGACATGG-AC-CACCGATGC-
TTTGTGAGGTGCCCTCGAAGAGTCGCGTTGTTTGGGAATGCAGCGCAAAATGGGTGGTAAATTC
CATCTAAGGCTAAATATTGGCGAGAGACCGATAGCGAACAAGTACCGTGAGGGAAAGATGAAA
AGCACTTTGGAAAGAGAGTTAAACAGTACGTGAAATTGTTGAAAGGGAAACGCTTGAAGTCAG
TCGCGTCCCGTGGCACTCAGCCTTGC----TTCGGCTTGGTGTACTTGCCTT-G---
GGACGGGTCAACATCAA-TTTT-GGC-
CGGTGGACAAGGGTGAGGGGAATGTAGCGTTGTTTCGGCGACGTG-TTATAGC-CCCTTGTC-
GAAT-ACACCGGCTGGGATTGAGGA-CCGCAGCACGCCC------TT-G-TGGCC-------
GGGGG----------------------TT---CG-CCCCATG-T-AACGTGCTTAGGAT-
GTTGGCATAATGGCTTTAAGCGACCCGTCTTGAAACACGGACCAAGGAGTCTAACATGCTTGCG
AGTGTTCGGGTGGAAAACCCTTGCGCGTAATGAAAGTGAAA-GTTGGGAG-----CCTC-------
-----------CGTGA--GGGGGTGCACCGACGCCTGGCCCTGACG-----
TTCTCTGACGGTGCTGCGGTAGAGCAAGTATGTTGGG----
ACCCGAAAGATGGTGAACTATGCCTGAATAGGGCGAAGCCAGAGGAAACTCTG-
GTGGAGGCTCGTAGCGATTCTGACGTGCAAATCGATCGTCAAATTTGGG-----------------
----

>Phylloporia_pulla_LWZ_20190428-3

TAACTGCGAGTGAAGCGGGAAGAGCTCAAATTTGTAATCT-G-GCG-GTC--
TCTGGCCGTCCGAGTTGTAATCTGTAGAGGCGTCGT-CCGCGTCG-
GCCCGTGTACAAGTCTCCTGGGATGGAGCGTCATAGAGGGTGAGAATCCCG-
TCCATGACATGG-AC-CACCGATGC-
TTTGTGAGGTGCCCTCGAAGAGTCGCGTTGTTTGGGAATGCAGCGCAAAATGGGTGGTAAATTC
CATCTAAGGCTAAATATTGGCGAGAGACCGATAGCGAACAAGTACCGTGAGGGAAAGATGAAA
AGCACTTTGGAAAGAGAGTTAAACAGTACGTGAAATTGTTGAAAGGGAAACGCTTGAAGTCAG
TCGCGTCCCGTGGCACTCAGCCTTGC----TTCGGCTTGGTGTACTTGCCTT-G---
GGACGGGTCAACATCAA-TTTT-GGC-
CGGTGGACAAGGGTGAGGGGAATGTAGCGTTGTTTCGGCGACGTG-TTATAGC-CCCTTGTC-
GAAT-ACACCGGCTGGGATTGAGGA-CCGCAGCACGCCC------TT-G-TGGCC-------
GGGGG----------------------TT---CG-CCCCATG-T-AACGTGCTTAGGAT-
GTTGGCATAATGGCTTTAAGCGACCCGTCTTGAAACACGGACCAAGGAGTCTAACATGCTTGCG
AGTGTTCGGGTGGAAAACCCTTGCGCGTAATGAAAGTGAAA-GTTGGGAG-----CCTC-------

-----------CGTGA--GGGGGTGCACCGACGCCTGGCCCTGACG-----
TTCTCTGACGGTGCTGCGGTAGAGCAAGTATGTTGGG----
ACCCGAAAGATGGTGAACTATGCCTGAATAGGGCGAAGCCAGAGGAAACTCTG-
GTGGAGGCTCGTAGCGATTCTGACGTGCAAATCGATCGTCAAATTTGGG------------------
----

>Phylloporia_radiata_LWZ_20141122-5
ATGAGTTTTGA-----A-A---GCGGGG-------------------------------TCTGCTGCTG--
GTGTG----------------GAAACG--CACA-TGTG-CACGGC-----------------------------
---CCT--GTA-----CTCAATCC--A--CTC------------------AA-CCCC--TGTGCA-
CCTTTGCA--AGG--------------------
-------------------------------------CGAGTAGG-----------------C-----TCTTACGC-
----------------------------TTTGAA-------------GTGTTTAGTAACTAGTAAGGCGAGC-
--GTGC----------------------------------

----------------------------ATGAGCATG---------------------------AGTAGT-
CTGAAAGTGA----------------------------------AAGGGCT---TCATTGTTTGAA--
GTGTCAAGTAGCAAGTAAGG--TTGAAGGTGCGTAAGTG----
CCAGTGGCCTGAAAGTGAAGA------TGCATCACTGTTTGAATTGATT-
AGTAGCGAGGAAGGGTGA--ATGTGCGCAAGTGCAAGTA----------------GCCTGAA------

-AGCGAGCG---------------------TCTTTCAAAT------------------
GGAGGGCTGAAAGC-TTTGA------TCTTT----AT-------------CA---ACTCTTTGT-
ATGTCTTGTGAAT--G-----T-GTT-GC-T-CC-TT----GTGAGTGAAAC-------
AAATACAACTTTCAACAATGGATCTCTTGGCTCTCGCATCGATGAAGAACGCAGCGAAATGCGA
TAAGTAATGTGAATTGCAGAATTCAGTGAATCATCGAATCTTTGAACGCACCTTGCGCCCTTTGG
-TATTCTGAAGGGCATGCCTGTTTGAGTGTCAGGTTAATCTCAAACCTC--------AAG-
CCTTTTG--------------TTA----GGC------TGA-ATGGTTTGGAC-ATGGAGG-C--------
TTTGCTGGT-GCAT---------------GCTTC--GTGTGTGTCGGCTTCTCTGAAAAGCATTAGC-
TGGGCTTTGG----------------T-TT---GCA-TGA-----------ATGGTGTAATAGTAA------
---------TATG-TTCAC----CAGATG-TGCTTG-CCTAA-AGGGTCTGC-TTC-----
TAATAGTCTT------------------CGGAC-AA--------GG-TTC---TTTGC-GAC----C-
TTTGCTTGACCATTTGACCTCATCCCCTAGTAACTGCGAGTGAACCGGGAAGAGCTCAAATTTGT
AATCT-G-ACG-GTCACTCTGGCCGTTCGAGTTGTAATCTGTAGAGGCGTTGT-CTGCATCG-
GCCTGTGTACAAGTCTCCTGGAATGGAGCGTCATAGAGGGTGAGAATCCCG-
TCCATGGCACAG-AC-CACCGATGC-
TTTGTGAGGCGCCCTCGAAGAGTCGCGTTGTTTGGGAATGCAGCGCAAAATGGGTGGTAAATTC
CATCTAAGGCTAAATATTGGCGAGAGACCGATAGCGAACAAGTACCGTGAGGGAAAGATGAAA
AGCACTTTGAAAAGAGAGTTAAACAGTACGTGAAATTGTTGAAAGGGAAACGCTTGAAGTCAGT
CACGTCCTGAGGCACTCAGCCTTGC----TTCGGCTTGGTGTACTTGCCTT-G---
GGACGGGTCAACATCAA-TTTT-GGC-
TGGTGGATAAGGGTGAGAGGAATGTGGCGTTGTTTCAGCAACGTG-TTATAGC-CTTTTGTC-
AAAT-GCACCAGCTGGGATTGAGGA-TCGCAGCACGCCC------TT-G-TGGCC-------
GGAGG----------------------CT---TG-CCTCACG-T-
AACGTGCTTAGGATGGTTGGCATAATGGCTTTAAGCGACCCGTCTTGAAACACGGACCAAGGA
GTCTAACATGCATGCAAGTGTTTGGGTGGAAAACCCTTGCGCGTAATAAAAGTGAAA-
GTTGGGAG-----CCTT------------------CGCAA--
GGGGGTGCACTGACGCCCGGCCCTGAAG-----
TTCTCTGATGGTGCTGCGGTAGAGCATGTATGTTGGG----
ACCCGAAAGATGGTGAACTATGCCTGAATAGGGTGAAGCCAGAGGAAACTCTG-
GTGGAGGCTCGTAGCGATTCTGACGTGCAAATCGATCGTCAAATTTGGGTTCACGAGAC--AT--
-CGAGGATAACGTAAGGGAAAAT-
AATGATGTAACCTTACTATTAGTGTCGTCCAAATGCTCGTGCCAGAAGACTCGGTAAGGCGAGA
GACGCAAACGTTAGTCGTCTTGATCAGGCGTAAAGGGTTTGTAGGCAGCTTTAAGTATAAAA-
TTTAATGTTTACTATTAT--------------------------------AATATTTTTA---------------
----

AGTTATAATATTTATTATAGGATCAATTAAAGCTAGAATCTAGAAGAGGTGA----
ATACAATGCTTGAG-CTTAGGAGTACTAACCGCAAACATCAAGTAGAGTGC-
TAAGGGCGAAGGC-
AGTTTTCCAATAAAGATTGACGCTGATAAACGAAGGTGAGGATAGAAAAAAGGATTAGATACCC
TTGTATCCCTCACTGTCAACAATGAATGGTAGTTACTAGGGAGAA-----------------------
----
ATGACCTAGAGGCGATGTTAACACGTAAACCATTCCGCCTTGTGAGTACGACTGCAAAGTTGAA
AACAAAAAAATTAGTCGGTTTCGGAG--CAAACGAAGTGAAGC-

ATGTTATTTAATTGGTACTGGTGAGTTCAAGGCTGGGATCTCGAAGGACGGTCAGACCCGCGAG
CACGCTCTGCTTGCGTTCACCCTCGGTGTGCGTCAGCTGATCGTCGCCGTGAACAAAATGGACA
CAGCCAAGGTTGGTGGGTGCTATTGA--CCT---TTGAAACGAAACTGAATC--ACGTTCG-----
--
AATAATCCAGTGGTCTGAGGAACGATTCAACGAAATCGTTAAGGAGACGTCGAACTTCATCAAG
AAAGTCGGCTACAACCCGAAGGCGGTATGCTTCGCCCCTATCTCCGGATGGCACGGTGACAAC
ATGCTTGAGGAGTCGACCAAGTATGCTTGACTT-TATCCTGATTTATAATATAAGCG------
AAGCTGACGTGACATTTCTCCAGTATGCCTTGGTATAAGGGCTGGTCCAAGGAGACGAAGG---
---------GTGGTGTCGTC-
AAGGGAAAGACTCTCCTTGACGCCATCGATGCCATCGAACCCCCAGTCCGTCCGGAGAACAAG
CCCTTGCGACTTCCTCTCCAGGATGTG---

>Phylloporia_radiata_LWZ_20141122-6
ATGAGTTTTGA-----A-A---GCGGGG------------------------------TCTGCTGCTG--
GTGTG---------------GAAACG--CACA-TGTG-CACGGC----------------------------
---CCT--GTA-----CTCAATCC--A--CTC-----------------AA-CCCC--TGTGCA-
CCTTTGCA---AGG-------------------
-------------------------------------TGAGTAGG----------------C-----TCTTACGC-
---------------------------TTTGAA-------------GTGTTTAGTAACTAGTAAGGCGAGC-
--GTGC---------------------------------

---------------------------ATGAGCATG--------------------------AGTAGT-
CTGAAAGTGA----------------------------------AAGGGCT---TCATTGTTTGAA--
GTGTCAAGTAGCAAGTAAGG--TTGAAGGTGCGTAAGTG----
CCAGTGGCCTGAAAGTGAAGA------TGCATCACTGTTTGAATTGATT-
AGTAGCGAGGAAGGGTGA--ATGTGCGCAAGTGCAAGTA---------------GCCTGAA------
-AGCGAGCG---------------------TCTTTCAAAT-------------------
GGAGGGCTGAAAGC-TTTGA------TCTTT----AT-------------CA---ACTCTTTGT-
ATGTCTTGTGAAT--G-----T-GTT-GC-T-CC-TT----GTGAGTGAAAC-------
AAATACAACTTTCAACAACGGATCTCTTGGCTCTTGCATCGATGAAGAACGCAGCGAAATGCGA
TAAGTAATGTGAATTGCAGAATTCAGTGAATCATCGAATTTTTGAACGCACCTTGCGCCCTTTGG
-TATTCCGAAGGGCATGCCTGTTTGAGTGTCAGGTTAATCTCAAACCTC--------AAG-
CCTTTTG--------------TTA----GGC------TGA-ATGGTTTGGAC-ATGGAGG-C--------
TTTGCTGGT-GCAT---------------GCTTC--GTGTGTGTCGGCTCCTCTGAAAAGCATTAGC-
TGGGCTTTGG----------------T-TT---GCA-TGA-----------ATGGTGTAATAGTAA------

---------TATG-TTCAC----CGGATG-TGCTTG-CCTAA-AGGGTCTGC-TTC-----
TAATAGTCTT------------------CGGAC-AA--------GG-TTC---TTTGC-GAC----C-
TTTGCTTGACCATTTGACCTCATCCCCTAGTAACTGCGAGTGAAGCGGGAAGAGCTCAAATTTGT
AATCT-G-ACG-GTCACTCTGGCCGTTCGAGTTGTAATCTGTAGAGGCGTTGT-CTGCATCG-
GCCTGTGTACAAGTCTCCTGGAATGGAGCGTCATAGAGGGTGAGAATCCCG-
TCCATGGCACAG-AC-CACCGATGC-
TTTGTGAGGCGCCCTCGAAGAGTCGCGTTGTTTGGGAATGCAGCGCAAAATGGGTGGTAAATTC
CATCTAAGGCTAAATATTGGCGAGAGACCGATAGCGAACAAGTACCGTGAGGGAAAGATGAAA
AGCACTTTGAAAAGAGAGTTAAACAGTACGTGAAATTGTTGAAAGGGAAACGCTTGAAGTCAGT
CGTGTCCTGAGGCACTCAGCCTTGC----TTCGGCTTGGTGTACTTGCCTT-G---
GGACGGGTCAACATCAA-TTTT-GGC-
TGGTGGATAAGGGTGAGAGGAATGTGGCGTTGTTTCAGCAACGTG-TTATAGC-CTTTTGTC-
AAAT-GCACCAGCTGGGATTGAGGA-TCGCAGCATGCCC------TT-G-TGGCC-------
GGAGG----------------------CT---TG-CCTCACG-T-
AACGTGCTTAGGATGGTTGGCATAATGGCTTTAAGCGACCCGTCTTGAAACACGGACCAAGGA
GTCTAACATGCATGCAAGTGTTTGGGTGGAAAACCCTTGTGCGTAATAAAAGTGAAA-
GTTGGGAG-----CCTT------------------CGCAA--
GGGGGTGCACTGACGCCCGGCCCTGAAG-----
TTCTCTGATGGTGCTGCGGTAGAGCATGTATGTTGGG----
ACCCGAAAGATGGTGAACTATGCCTGAATAGGGTGAAGCCAGAGGAAACTCTG-
GTGGAGGCTCGTAGCGATTCTGACGTGCAAATCGATCGTCAAATTTGGGTTGATATGAC--AT--
-CGAGGATAACGTAAGGGAAAAT-
AATGATGTAACCTTACTATTAGTGTCGTCCAAATGCTCGTGCCAGAAGACTCGGTAAGGCGAGA
GACGCAAACGTTAGTCGTCTTGATCAGGCGTAAAGGGTTTGTAGGCAGCTTTAAGTATAAAA-
TTTAATGTTTACTATTAT--------------------------------AATATTTTTA---------------
----

AGTTATAATATTTATTATAGGATCAATTAAAGCTAGAATCTAGAAGAGGTGA----
ATACAATGCTTGAG-CTTAGGAGTACTAACCGCAAACATCAAGTAGAGTGC-
TAAGGGCGAAGGC-
AGTTTTCCAATAAAGATTGACGCTGATAAACGAAGGTGAGGATAGAAAAAAGGATTAGATACCC
TTGTATCCCTCACTGTCAACAATGAATGGTAGTTACTAGGGAGAA------------------------
----
ATGACCTAGAGGCGATGTTAACACGTAAACCATTCCGCCTTGTGAGTACGACTGCAAAGTTGAA
AACAAAAAAATTAGTCGGTTTCGGAG--CAAACGAAGTGAAGC-
ATGTTATTTAATTGGTACTGGTGAGTTCGAGGCTGGGATCTCGAAGGACGGTCAGACCCGCGAG
CACGCTCTGCTTGCGTTCACCCTCGGTGTGCGTCAGCTGATCGTCGCCGTGAACAAAATGGACA
CAGCCAAGGTTGGTGGGTGCTATTGA--CCT---TTGAAACGAAACTGAATC--ACGTTCG-----
--
AATAATCCAGTGGTCTGAGGAACGATTCAACGAAATCGTTAAGGAGACGTCGAACTTCATCAAG
AAAGTCGGCTACAACCCGAAGGCGGTATGCTTCGCCCCTATCTCCGGATGGCACGGTGACAAC
ATGCTTGAGGAGTCGACCAAGTATGCTTGACTT-TATCCTGATTTGTAATATAAGCG------
AAGCTGACGTGACATTTCTCCAGTATGCCTTGGTATAAGGGCTGGTCCAAGGAGACGAAGG---
---------GTGGTGTCGTC-

AAGGGAAAGACTCTCCTTGACGCCATCGATGCCATCGAACCCCCAGTCCGTCCGGAGAACAAG
CCCTTGCGACTTCCTCTCCAGGATGTG--

>Phylloporia_rattanicola_Dai_18235
ACAAGTTTTGA-----A-A---GTGGAG------------------------------TCTGATGCTG--
GTGTG---------------GAAATG--CACA-CGTG-CACGGC----------------------------
---TCT--GTG-----CTCGATCC--A--CTC-----------------AA-TCCC--TGTGCA-CC-
TTGCA--AAG-----------------------
--------------------------------TGAGTAGG----------------CT----CTTCGCCC-----
-----------------------TTTGAAGT-----------GTGACCAGTAGTGAGTAAGGCGAGC----
--------------------------------------GTGT------------------------------------
----
------------------------GTGAGC---

---------------------GCACCAGTA---------------GCCTGAA-------AGCGAGCG-----
----------------TCTTTCATTT-----------AGAGAG-AGTGCGGTTGAAAGC-TTTGA------
TCTTC----AT-------------CA---ACCTTTCGT-ATGTCATGTGAAT--G-----T-GTT-GC-T-
TC-TC----GTGGGCGAAAT-------
AAATACAACTTTCAACAACGGATCTCTTGGCTCTCGCATCGATGAAGAACGCAGCGAAATGCGA
TAAGTAATGTGAATTGCAGAATTCAGTGAATCATCGAATCTTTGAACGCACCTTGCGCCCTTTGG
-TATTCCGAAGGGCATGCCTGTTTGAGTGTCATGTTTATCTCAAACCCC--------AAG-
CCTTTTG--------------TTA----GGT--------------TTTGGAC-TTGGAGG-C-T------
TTTGCTGGT-GTGC---------------GCTCT--GCGTGTGTCGGCTCCTCTGAAAAGCATTAGC-
TGGACTTTGG----------------T-TC---GCG-TGC-----------ATGGTGTGATAGTAA-----
----------TGTG-TTCAC----CGGAAG-CGCTTG-CCTAA-TGGGTCTGC-TAC-----
TAATTGTCTT------------------CGGAC-AA--------GG-TCC---TTTGT-GAC----C-
TTCGATTGACCGTTTGACCTCA-
CCCCTAGTAACTGCGAGTGAAGCGGGAAGAGCTCAAATTTGTAATCT-G-GCG-GTC--
TCTGGCCGTCCGAGTTGTAATCTGTAGAGGCGTCGT-CCGCGTCG-
GCCCGTGTACAAGTCTCCTGGGATGGAGCATCATAGAGGGTGAGAATCCCG-
TCCATGACATGG-AC-CACCGATGC-
TTTGTGAGATGCCCTCGAAGAGTCGCGTTGTTTGGGAATGCAGCGCAAAATGGGTGGTAAATTC
CATCTAAGGCTAAATATTGGCGAGAGACCGATAGCGAACAAGTACCGTGAGGGAAAGATGAAA
AGCACTTTGGAAAGAGAGTTAAACAGTACGTGAAATTGTTGAAAGGGAAACGCTTGAAGTCAG

TCGTGTCCCGTGGCATTCAGCCTTGC----TTCGGCTTAGTGTACTTGCCTT-G---
GGATGGGTCAACATCAA-TTTT-GGC-
TGGTGGACAAGGGTGAGGGGAATGTAGCGTCGTTTTGGCGATGTG-TTATAGC-CTCTTGTC-
GAAT-ACACTGGTTGGGATTGGGGA-CTGCAGCACGCCC------TT-G-TGGCC-------
AGGGG----------------------TT---CA-CCTCATG-T-AACGTGCTTAGGAT-
GTTGGCATAATGGCTTTAAGCGACCCGTCTTGAAACACGGACCAAGGAGTCTAACATGCTTGCG
AGTGTTCGGGTGGAAAACCCTTGCGCGTAATAAAAGTGAAA-GTTGGGAG-----CCTC-------
-----------TGCAA-GGGGGGTGCACCGACGCCTGGCCCTGACG-----
TTCTCTGATGGTGCTGTGGTAGAGCAAGTATGTTGGG----
ACCCGAAAGATGGTGAACTATGCCTGAATAGGGCGAAGCCAGAGGAAACTCTG-
GTGGAGGCTCGTAGCGATTCTGACGTGCAAATCGATCGTCAAATTTGGGTTGATGTGAC--AT--
-CGGGGATAACGTAAGGGATAAT-
AATGATATTACCTTACTATTAGTGTCGTCCAAATGCTCGTGCCAGAAGACTCGGTAAGGCGAGA
GACGCAAACGTTAGTCGTCTTGATCAGGCGTAAAGGGTTTGTAGGCAGCTTTAAGTATCCAA-
TTTAATGTTTACTATTAT--------------------------------AATATTTACA--------------
----

AGTGATAATATTTATTAAAGGATCAATTAAAGCTAGAATCTAGAAGAGGTGA----
ATACAATGCTTGAG-CTTAGGAGTACTAACCGCAAACATCAAGTAGAGTGC-
TAAGGGCGAAGGC-
AGTTTTCCAATAAAGATTGACGCTGAGAAACGAAGGTGAGGATAGAAAAAAGGATTAGATACC
CTTGTATCCCTCACTGTCAACGATGAATGGTAGTTACTAGGGAGAA----------------------
----
ATGACCTAGAGGCGATGTTAACACGTAAACCATTCCGCCTTGTGAGTACGACTGCAAAGTTGAA
AACAAAAAAATTAGTCGGTTTCGGAG--CAAACGAAGTGAAGC-
ATGTTATTTAATTGGTACTGGTGAGTTCGAGGCTGGTATCTCGAAGGACGGTCAGACCCGCGAG
CACGCTCTGCTTGCGTTCACCCTCGGTGTGCGTCAGCTGATCGTTGCTGTGAACAAGATGGACA
CGGCTAAGGTTGGT-GGTGCCATTGA--CCACT-CCGTAACGAAGCTGAATC--GCGTTTG-----
--
AATACTCCAGTGGTCTGAGGAACGATGCAACGAAATTATTAAGGAGACATCGAACTTCATTAAG
AAAGTCGGCTACAACCCCAAGACCGTACCTTTCGTCCCGATCTCCGGATGGCACGGTGACAAC
ATGCTTGAGGAGTCGAGCAAGTATGTTTGATTT-TGCCC-----TACAATGTAAGCG-----
TGTGCCGACGTGACATCT-TTCAGTATGACTTGGTACAAGGGCTGGACCAAAGAGACGAAGG--
----------GCGGTGTCGTT-
AAGGGAAAGACTCTTCTCGATGCCATTGATGCCATCGAGCCCCCTGTCCGCCCAGAGAACAAA
CCCTTGCGACTTCCTCTCCAGGATGTGATTTCCGTTGGATCGTTGTCCGCTCCGGTACTCGAATT
CTTGGAGGAATGGGGCTTGGAGAGCCTCGAGGAAAATGCACA---AGCTGCAA------
ATTTGACCAAAGTTTTCGTGAATGGCGTATGGCTCGGAGTACACCGAGACCCGCACACCCTTGT
CAACACACTTCGAACGCTCAGGAGGAAGG---
AGGATCTCAACGCTGAGGTTTCCATTGTTCGTGACATACGCGAAAAGGAAGTTCGCATCTATAC
GGACGCAGGACGTGTTCTTCGACCACTTTTCATCGTCGAGGACAATAGATTAAAGCTGAGGCGT
CAGCACCTGCGCTGGCTTCAGGCGAAGTGGAGGATT-------
GAGGAGAGGCCCAAAGACAGGGTCTT------------
GGATGAGGGCGAAGAATCAGCAGGCGAGACAATAACTGAGGTAGAAGACGATGGGAAGCCAA

TAATACGGGAAGGGGTTCGAATGAAGAAGAAGAGGAAACCTGTCACCTGGAAGACATTGATTA
GCGACGGCGTTATTGAATACCTTGATGCAGAGGAAGAGGAAACGGTCATGATCTGCATGACAC
CAGAAGATCTGGTCGAATCTCGGTTGATGAGGCAAGGCCTGGATCCTCG---
AGCAGACATGGAGTTCGATGCATCTACCCGTCTCAAGCCTGTACCAACT------
GCTCATAACTGGACACATTGCGAGATTCATCCTAGCATGATTCTAGGTATATGTGCAAGTATCAT
TCCATTCCC
>Phylloporia_rattanicola_Dai_18232
ACAAGTTTTGA-----A-A---GTGGAG------------------------------TCTGATGCTG--
GTGTG---------------GAAATG--CACA-CGTG-CACGGC----------------------------
---TCT--GTG-----CTCGATCC--A--CTC-----------------AA-TCCC--TGTGCA-CC-
TTGCA--AAG-----------------------
--------------------------------TGAGTAGG----------------CT----CTTCGCCC-----
-----------------------TTTGAAGT-----------GTGACCAGTAGTGAGTAAGGCGAGC----
--------------------------------------GTGT------------------------------------
----
------------------------GTGAGC---

---------------------GCACCAGTA---------------GCCTGAA-------AGCGAGCG-----
----------------TCTTTCATTT-----------AGAGAG-AGTGCGGTTGAAAGC-TTTGA------
TCTTC----AT-------------CA---ACCTTTCGT-ATGTCATGTGAAT--G-----T-GTT-GC-T-
TC-TC----GTGGGCGAAAT-------
AAATACAACTTTCAACAACGGATCTCTTGGCTCTCGCATCGATGAAGAACGCAGCGAAATGCGA
TAAGTAATGTGAATTGCAGAATTCAGTGAATCATCGAATCTTTGAACGCACCTTGCGCCCTTTGG
-TATTCCGAAGGGCATGCCTGTTTGAGTGTCATGTTTATCTCAAACCCC--------AAG-
CCTTTTG--------------TTA----GGT--------------TTTGGAC-TTGGAGG-C-T------
TTTGCTGGT-GTGC---------------GCTCT--GCGTGTGTCGGCTCCTCTGAAAAGCATTAGC-
TGGACTTTGG----------------T-TC---GCG-TGC-----------ATGGTGTGATAGTAA-----
----------TGTG-TTCAC----CGGAAG-CGCTTG-CCTAA-TGGGTCTGC-TAC-----
TAATTGTCTT------------------CGGAC-AA--------GG-TCC---TTTGT-GAC----C-
TTCGATTGACCGTTTGACCTCATCCCCTAGTAACTGCGAGTGAAGCGGGAAGAGCTCAAATTTG
TAATCT-G-GCG-GTC--TCTGGCCGTCCGAGTTGTAATCTGTAGAGGCGTCGT-CCGCGTCG-
GCCCGTGTACAAGTCTCCTGGGATGGAGCATCATAGAGGGTGAGAATCCCG-
TCCATGACATGG-AC-CACCGATGC-
TTTGTGAGATGCCCTCGAAGAGTCGCGTTGTTTGGGAATGCAGCGCAAAATGGGTGGTAAATTC
CATCTAAGGCTAAATATTGGCGAGAGACCGATAGCGAACAAGTACCGTGAGGGAAAGATGAAA
AGCACTTTGGAAAGAGAGTTAAACAGTACGTGAAATTGTTGAAAGGGAAACGCTTGAAGTCAG
TCGTGTCCCGTGGCATTCAGCCTTGC----TTCGGCTTAGTGTACTTGCCTT-G---
GGATGGGTCAACATCAA-TTTT-GGC-
TGGTGGACAAGGGTGAGGGGAATGTAGCGTCGTTTTGGCGATGTG-TTATAGC-CTCTTGTC-
GAAT-ACACTGGTTGGGATTGGGGA-CTGCAGCACGCCC------TT-G-TGGCC-------
AGGGG----------------------TT---CA-CCTCATG-T-AACGTGCTTAGGAT-
GTTGGCATAATGGCTTTAAGCGACCCGTCTTGAAACACGGACCAAGGAGTCTAACATGCTTGCG
AGTGTTCGGGTGGAAAACCCTTGCGCGTAATAAAAGTGAAA-GTTGGGAG-----CCTC-------

-----------TGCAA-GGGGGGTGCACCGACGCCTGGCCCTGACG-----
TTCTCTGATGGTGCTGTGGTAGAGCAAGTATGTTGGG----
ACCCGAAAGATGGTGAACTATGCCTGAATAGGGCGAAGCCAGAGGAAACTCTG-
GTGGAGGCTCGTAGCGATTCTGACGTGCAAATCGATCGTCAAATTTGGGTTGATGTGAC--AT--
-CGGGGATAACGTAAGGGATAAT-
AATGATATTACCTTACTATTAGTGTCGTCCAAATGCTCGTGCCAGAAGACTCGGTAAGGCGAGA
GACGCAAACGTTAGTCGTCTTGATCAGGCGTAAAGGGTTTGTAGGCAGCTTTAAGTATCCAA-
TTTAATGTTTACTATTAT--------------------------------AATATTTACA---------------
----

AGTGATAATATTTATTAAAGGATCAATTAAAGCTAGAATCTAGAAGAGGTGA----
ATACAATGCTTGAG-CTTAGGAGTACTAACCGCAAACATCAAGTAGAGTGC-
TAAGGGCGAAGGC-
AGTTTTCCAATAAAGATTGACGCTGAGAAACGAAGGTGAGGATAGAAAAAAGGATTAGATACC
CTTGTATCCCTCACTGTCAACGATGAATGGTAGTTACTAGGGAGAA-----------------------
----
ATGACCTAGAGGCGATGTTAACACGTAAACCATTCCGCCTTGTGAGTACGACTGCAAAGTTGAA
AACAAAAAAATTAGTCGGTTTCGGAG--CAAACGAAGTGAAGC-
ATGTTATTTAATTGGTACTGGTGAGTTCGAGGCTGGTATCTCGAAGGACGGTCAGACCCGCGAG
CACGCTCTGCTTGCGTTCACCCTCGGTGTGCGTCAGCTGATCGTTGCTGTGAACAAGATGGACA
CGGCTAAGGTTGGT-GGTGCCATTGA--CCACT-CCGTAACGAAGCTGAATC--GCGTTTG-----
--
AATACTCCAGTGGTCTGAGGAACGATGCAACGAAATTATTAAGGAGACATCGAACTTCATTAAG
AAAGTCGGCTACAACCCCAAGACCGTACCTTTCGTCCCGATCTCCGGATGGCACGGTGACAAC
ATGCTTGAGGAGTCGAGCAAGTATGTTTGATTT-TGCCC-----TACAATGTAAGCG-----
TGTGCCGACGTGACATCT-TTCAGTATGACTTGGTACAAGGGCTGGACCAAAGAGACGAAGG--
----------GCGGTGTCGTT-
AAGGGAAAGACTCTTCTCGATGCCATTGATGCCATCGAGCCCCCTGTCCGCCCAGAGAACAAA
CCCTTGCGACTTCCTCTCCAGGATGTGATTTCCGTTGGATCGTTGTCCGCTCCGGTACTCGAATT
CTTGGAGGAATGGGGCTTGGAGAGCCTCGAGGAAAATGCACA---AGCTGCAA------
ATTTGACCAAAGTTTTCGTGAATGGCGTATGGCTCGGAGTACACCGAGACCCGCACACCCTTGT
CAACACACTTCGAACGCTCAGGAGGAAGG---
AGGATCTCAACGCTGAGGTTTCCATTGTTCGTGACATACGCGAAAAGGAAGTTCGCATCTATAC
GGACGCAGGACGTGTTCTTCGACCACTTTTCATCGTCGAGGACAATAGATTAAAGCTGAGGCGT
CAGCACCTGCGCTGGCTTCAGGCGAAGTGGAGGATT-------
GAGGAGAGGCCCAAAGACAGGGTCTT------------
GGATGAGGGCGAAGAATCAGCAGGCGAGACAATAACTGAGGTAGAAGACGATGGGAAGCCAA
TAATACGGGAAGGGGTTCGAATGAAGAAGAAGAGGAAACCTGTCACCTGGAAGACATTGATTA
GCGACGGCGTTATTGAATACCTTGATGCAGAGGAAGAGGAAACGGTCATGATCTGCATGACAC
CAGAAGATCTGGTCGAATCTCGGTTGATGAGGCAAGGCCTGGATCCTCG---
AGCAGACATGGAGTTCGATGCATCTACCCGTCTCAAGCCTGTACCAACT------
GCTCATAACTGGACACATTGCGAGATTCATCCTAGCATGATTCTAGGTATATGTGCAAGTATCAT
TCCATTCCC
>Phylloporia_rubiacearum_Chen3580

TTAGTAACTGCGAGTGAAGAGGGAAGGGCTCAAATTTGGAATCT-G-GCG-
GTCCTGGGGGCCGTCCGAGTTGTAATCTAAAGAGGCGTCGT-CTGCGTCG-
GACCGTGTGCAAGTCTCCTGGAACGGAGCGTCGTAGAGGGTGAGAATCCCG-
TCTAGGACACGGAGC-CTCCGATGG-
GTTGTGAGGCGCTCTCGAAGAGTCGCGTTGTTTGGGAATGCAGCGCAAAATGGGTGGTAAATTC
CATCTAAGGCTAAACATGGGCGAGAGACCGATAGCGAACAAGTACCGTGAGGGAAAGATGAA
AAGCACTTTGGAAAGAGAGTTAAACAGCACGTGAAATTGTTGAAAGGGAAACGCTTGATGTCA
GTCGCGTCTGGAGGCATTCAGCCGTGCCC--TTGGGCTTGGTGTACTTGCCTC-C---
GGACGGGTCAACGTCAA-TTTT-GGT-
CGGCGGACAAGGGCGAGGGGAATGTGACGTGGCTTCGGCGACGTG-TTATAGC-CTCTCGTC-
GGAC-GCGTTGGCTGGGATTGAGGA-CCGCAGCGCGCCC------CT-G-GTGTT-------
GGTGGCCGGAGGTGGGGGGTTGCTCCCTC---TG-CCTTACGTT-TACGCGCTTAGGAT-
GTTGGCATAATGGCTTTAAGCGACCCGTCTTGAAACACGGACCAAGGAGTCTAACATGCTTGCG
AGTGTTCGGGTGGAAAACCCTTGCGCGGAATGAAAGTGAAG-GTCGGGAG-----CCTT------
------------TGAAA-
AAGGGGTGCACCGACGCCCGGCCCTGAGGAGAAACCCTCCGACGGTGCTGCGGCAGAGCAGG
TGTGTTGGG----
ACCCGAAAGATGGTGAACTATGCCTGAAGAGGGTGAAGCCAGAGGAAACTCTG-
GTGGAGGCTCGCAGCGATTCTGACGTGCAAATCGATCGTCGAATTTGGG------------------
----

>Phylloporia_rubiacearum_Chen_3583

AGTAACTGCGAGTGAAGAGGGAAGGGCTCAAATTTGGAATCT-G-GCG-
GTCCTGGGGGCCGTCCGAGTTGTAATCTAAAGAGGCGTCGT-CTGCGTCG-
GACCGTGTGCAAGTCTCCTGGAACGGAGCGTCGTAGAGGGTGAGAATCCCG-
TCTAGGACACGGAGC-CTCCGATGG-
GTTGTGAGGCGCTCTCGAAGAGTCGCGTTGTTTGGGAATGCAGCGCAAAATGGGTGGTAAATTC
CATCTAAGGCTAAACATGGGCGAGAGACCGATAGCGAACAAGTACCGTGAGGGAAAGATGAA
AAGCACTTTGGAAAGAGAGTTAAACAGCACGTGAAATTGTTGAAAGGGAAACGCTTGAAGTCA
GTCGCGTCTGGAGGCATTCAGCCGTGCCC--TTGGGCTTGGTGTACTTGCCTC-C---
GGACGGGTCAACGTCAA-TTTT-GGT-
CGGCGGACAAGGGCGAGGGGAATGTGACGTGGCTTCGGCGACGTG-TTATAGC-CTCTCGTC-
GGAC-GCGTTGGCTGGGATTGAGGA-CCGCAGCGCGCCC------CT-G-GTGTT-------
GGTGGCCGGAGGTGGGGGGTTGCTCCCTC---TG-CCTTACGTT-TACGCGCTTAGGAT-
GTTGGCATAATGGCTTTAAGCGACCCGTCTTGAAACACGGACCAAGGAGTCTAACATGCTTGCG
AGTGTTCGGGTGGAAAACCCTTGCGCGGAATGAAAGTGAAG-GTCGGGAG-----CCTT------
------------TGAAA-
AAGGGGTGCACCGACGCCCGGCCCTGAGGAGAAACCCTCCGACGGTGCTGCGGCAGAGCAGG
TGTGTTGGG----
ACCCGAAAGATGGTGAACTATGCCTGAAGAGGGTGAAGCCAGAGGAAACTCTG-
GTGGAGGCTCGCAGCGATTCTGACGTGCAAATCGATCGTCGAATTTGGG------------------
----

>Phylloporia_splendida_Cui_8429

TCCCCTAGTAACTGCGAGTGAAGCGGGAGGAGCTCAAATTTGTAATCT-G-GTG-GTC--
TCTGGCCATCCGAGTTGTAATCTGTAGAGGCGTCGT-CCGCGTCG-
GCCCGTGTACAAGTCTCCTGGAATGGAGCGTCATAGAGGGTGAGAATCCCG-
TCCATGACATGG-AC-CACCGATGC-
TCTGTGAGACACCCTCGAAGAGTCGCGTTGTTTGGGAATGCAGCGCAAAATGGGTGGTAAATTC
CATCTAAGGCTAAATATTGGCGAGAGACCGATAGCGAACAAGTACCGTGAGGGAAAGATGAAA
AGCACTTTGAAAAGAGAGTTAAACAGTACGTGAAATTGTTGAAAGGGAAACGCTTGAAGTCAGT
CGCGTCCCGTGGCACTCAGCCTTGC----TTTGGCTTGGTGTACTTGCCTT-GG--
TGATGGGTCAACATCGA-TTTTGGGC-
TGGCGGATGAGGGCGAGGAGAATGTAGCATTGCTTCGGTGATGTG-TTATAGC-CTCTCGTC-
AAAC-ACGTCGGTTGGGATCGAGGA-CTGCAGCACGCCC---TTTTT-G-TGGCC-------
AGGGGGG----------------GAGTTC---CT-CTCCATG-T-AACGTGCTTAGGAT-
GTTGGCATAATGGCTTTAAGCGACCCGTCTTGAAACACGGACCAAGGAGTCTAACATGCTTGCA
AGTGTTCGGGTGGAAAACCCTTGCGCGTAATGAAAGTGAAA-GTTGAGAG-----CCTC-------
-----------CGTGA-AGGGGGTGCACCGACGCCTGGCCCTGACG-----
TTCTCTGATGGTGCCGCGGTGGAGCAAGCACGTTGGG----
ACCCGAAAGATGGTGAACTATGCCTGAATAGGGCGAAGCCAGAGGAAACTCTG-
GTGGAGGCTCGTAGCGATTCTGACGTGCAAATCGATCGTCAAATTTGGG------------------
----

>Phylloporia_splendida_Dai_6282

TCCCCTAGTAACTGCGAGTGAAGCGGGAGGAGCTCAAATTTGTAATCT-G-GTG-GTC--
TCTGGCCATCCGAGTTGTAATCTGTAGAGGCGTCGT-CCGCGTCG-
GCCCGTGTACAAGTCTCCTGGAATGGAGCGTCATAGAGGGTGAGAATCCCG-
TCCATGACATGG-AC-CACCGATGC-
TCTGTGAGACACCCTCGAAGAGTCGCGTTGTTTGGGAATGCAGCGCAAAATGGGTGGTAAATTC
CATCTAAGGCTAAATATTGGCGAGAGACCGATAGCGAACAAGTACCGTGAGGGAAAGATGAAA
AGCACTTTGAAAAGAGAGTTAAACAGTACGTGAAATTGTTGAAAGGGAAACGCTTGAAGTCAGT
CGCGTCCCGTGGCACTCAGCCTTGC----TTTGGCTTGGTGTACTTGCCTT-GG--
TGATGGGTCAACATCGA-TTTTGGGC-
TGGCGGATGAGGGCGAGGAGAATGTAGCATTGCTTCGGTGATGTG-TTATAGC-CTCTCGTC-
AAAC-ACGTCGGTTGGGATCGAGGA-CTGCAGCACGCCC---TTTTT-G-TGGCC-------
AGGGGGG----------------GAGTTC---CT-CTCCATG-T-AACGTGCTTAGGAT-
GTTGGCATAATGGCTTTAAGCGACCCGTCTTGAAACACGGACCAAGGAGTCTAACATGCTTGCA
AGTGTTCGGGTGGAAAACCCTTGCGCGTAATGAAAGTGAAA-GTTGAGAG-----CCTC-------
-----------CGTGA-AGGGGGTGCACCGACGCCTGGCCCTGACG-----
TTCTCTGATGGTGCCGCGGTGGAGCAAGCACGTTGGG----
ACCCGAAAGATGGTGAACTATGCCTGAATAGGGCGAAGCCAGAGGAAACTCTG-
GTGGAGGCTCGTAGCGATTCTGACGTGCAAATCGATCGTCAAATTTGGG------------------
----

>Phylloporia_subpulla_Cui_5251

TCCCTTAGTAACTGCGAGTGAAGCGGGAAGAGCTCAAATTTGTAATCT-G-ATG-GTC--
TTTGACCATCTGAGTTGTAATCTGTAGAGGTGTTGT-CTGCATCG-
GCCCGTGTACAAGTCTCCTGGAATGGAGCGTCATAGAGGGTGAGAATCCCG-
TCCATGACACGG-AC-CACCGATGC-
TCTGTGAGGCGCCCTCAAAGAGTCGCGTTGTTTGGGAATGCAGCGCAAAATGGGTGGTAAATTC
CATCTAAGGCTAAATATTGGCGAGAGACCGATAGTGAACAAGTACCGTGAGGGAAAGATGAAA
AGCACTTTGGAAAGAGAGTTAAACAGTACGTGAAATTGTTGAAAGGGAAACGCTTAAAGTCAGT
CGCGTCTTGAGGCACTCAGCCTTGCC---TTTGGCTTGGTGTACTTGCCTT-G---
AGATGGGTCAACATCAA-TTTT-GGC-
TGGTGGACAAGAGTGAAAGGAATGTAGCGTCGTTTCGGCGACGTG-TTATAGC-CCTTCATT-
GAAT-ACACTGGCTGAGATTGAGGA-CTACAGCACGCCC------TT-G-TGGCC-----A-
GGGGG----------------------CT---CA-CCCCATG-T-AACGTGCTTAGGAT-
GTTGGCATAATGGTTTTAAGCGACCCGTCTTGAAACACGGACCAAGGAGTCTAACATGCTTGCG
AGTGTTCGGGTGGAAAACCCTTGCGCGTAATGAAAGTGAAA-GTTGGGAG-----CCTT-------
-----------CATAA--GGAGGTGCACCGACGCCCGGTCCTGATG-----
TTCTCTGATGGTGCTGCGGTAGAGCAAGTATGTTGGG----
ACCCGAAAGATGGTGAACTATGCCTGAATAGGGTGAAGCCAGAGGAAACTCTG-
GTGGAGGCTCGTAGCGATTCTGACGTGCAAATCGATCGTCAAATTTGGG------------------
----

>Phylloporia_subpulla_Dai_9627

TCCCTTAGTAACTGCGAGTGAAGCGGGAAGAGCTCAAATTTGTAATCT-G-ATG-GTC--
TTTGACCATCTGAGTTGTAATCTGTAGAGGTGTTGT-CTGCATCG-
GCCCGTGTACAAGTCTCCTGGAATGGAGCGTCATAGAGGGTGAGAATCCCG-
TCCATGACACGG-AC-CACCGATGC-
TCTGTGAGGCGCCCTCAAAGAGTCGCGTTGTTTGGGAATGCAGCGCAAAATGGGTGGTAAATTC
CATCTAAGGCTAAATATTGGCGAGAGACCGATAGTGAACAAGTACCGTGAGGGAAAGATGAAA
AGCACTTTGGAAAGAGAGTTAAACAGTACGTGAAATTGTTGAAAGGGAAACGCTTAAAGTCAGT
CGCGTCTTGAGGCACTCAGCCTTGCT---TTTGGCTTGGTGTACTTGCCTT-G---
AGATGGGTCAACATCAA-TTTT-GGC-
TGGTGGACAAGAGTGAAAGGAATGTAGCGTCGTTTCGGCGACGTG-TTATAGC-CCTTCATT-
GAAT-ACACTGGCTGAGATTGAGGA-CTACAGCACGCCC------TT-G-TGGCC-----
AGGGGGG----------------------CT---CA-CCCCATG-T-AACGTGCTTAGGAT-
GTTGGCATAATGGTTTTAAGCGACCCGTCTTGAAACACGGACCAAGGAGTCTAACATGCTTGCG
AGTGTTCGGGTGGAAAACCCTTGCGCGTAATGAAAGTGAAA-GTTGGGAG-----CCTT-------
-----------CATAA--GGAGGTGCACCGACGCCCGGTCCTGATG-----
TTCTCTGATGGTGCTGCGGTAGAGCAAGTATGTTGGG----
ACCCGAAAGATGGTGAACTATGCCTGAATAGGGTGAAGCCAGAGGAAACTCTG-
GTGGAGGCTCGTAGCGATTCTGACGTGCAAATCGATCGTCAAATTTGGG------------------
----

>Phylloporia_terrestris_Yuan_5738

TCCCCTAGTAACTGCGAGTGAAGCGGGAAGAGCTCAAATTTGTAATCT-G-GCG-GTCA-
TCTGGTCGTCTGAGTTGTAATCTGTAGAGGCATCAT-CTGTGTCG-
GCCCGTGTACAAGTCTCCTGGAATGGAGCGTCATAGAGGGTGAGAATCCCG-
TCCATGACACGG-AC-CGCCGATGC-
TCTGTGAGGTGCCCTCGAAGAGTCGCGTTGTTTGGGAATGCAGCGCAAAATGGGTGGTAAATTC
CATCTAAGGCTAAATATTGGCGAGAGACCGATAGTGAACAAGTACCGTGAGGGAAAGATGAAA
AGCACTTTGGAAAGAGAGTTAAACAGTACGTGAAATTGTTGAAAGGGAAACGCTTGAAGTCAG
TCGCGTCCCGAGGCACTCAGCCTTGC----TTCGGCTTGGTGTACTTGCCTT-G---
AGATGGGTCAGCATCAA-TTTT-GGC-
TGGTGGACAAGGTTGAGGGGAATGTAGCGTTGTTTCGATGACGTG-TTATAGC-TCCTTATC-
GAAT-ACACTGGTCGGGATTGAGGA-TTGCAGCACGCCC------TT-G-TGGTC-------
AGGGG----------------------TT---CG-CCCCATG-T-AACGTGCTTAGGAT-
GTTGGCATAATGGCTTTAAGCGACCCGTCTTGAAACACGGACCAAGGAGTCTAACATGCTTGCG
AGTGTTTGGGTGGAAAACCCTTGCGCGTAATGAAAGTGAAA-GTTGAGAG-----CCTC-------
-----------TATAAAAGGAGGTGCACTGACGCCCGGCCCTGACG-----
TTCTCTAATGGTGCTGCGGTAGAGCAAGTATGTTGGG----
ACCCGAAAGATGGTGAACTATGCCTGAATAGGGCGAAGCCAGAGGAAACTCTG-
GTGGAGGCTCGTAGCGATTCTGACGTGCAAATCGATCGTCAAATTTGGG------------------
----

>Phylloporia_terrestris_He_2359
ACGAGTTTTGA-----ACG---GCAGGG----------------------------------CCTGCTGCTG--
GTGCA---------------GAAATG--CGCA-TGTG-CACGGC----------------------------
---TCT--GTG-----CTCAATCC--A--CCT----------------------AA-CCCC--TGTGCA-
CCTTTGCA--AAG-------------------
-------------------------------------TGAGTAGG----------------C------TCCACCC-
----------------------------TTTGGAAG----------
TGAGACTAGTAGCGAGTAAGGCGAGC--
GTGC---
--GTGA---------

--GCGCCGGTA------
----------GTCTGAA-------AGTGAGCG----------------------TCTTTCATTT------------
GGGAG-GGTGTTGTCGAAGGC-TTTGA------TCTTT----AT-------------CA---
ACCCTTTGT-ATGTCTTGTGGAT--G-----T-GTT-GC-C-CC-TT----GTGGGTGAAAT-------
GAATACAACTTTCAACAACGGATCTCTTGGCTCTCGCATCGATGAAGAACGCAGCGAAATGCGA
TAAGTAATGTGAATTGCAGAATTCAGTGAATCATCGAATCTTTGAACGCACCTTGCGCCCTTTGG
-TATTCCGAAGGGCATGCCTGTTTGAGTGTCATGTTAATCTCAAACCTC--------AAG-
CCTTTCA--------------TTA----GGCT-----TGG-ACGGTTTGGAC-TTGGAGG-C--------
TTTGCTGGT-GTGCCA-------------CTTTT--GTGCGCGTCGGCTCCTCTGAAAAGCATTAGC-

TGGACTTTGG----------------T-TT---GCG-TGG-----------ATGGTGTAATAGTAA------
---------TGTG-TTCAC----CGGAAG-TGCTTG-CCTAA-CGGGTCCGC-TTC-----
TAATCGTCTT-------------------CGGAC-AA--------GG-TCC---TTTGC-GGC----C-
TTCGCCTTGATGTTTGACCTCATCCCCTAGTAACTGCGAGTGAAGCGGGAAGAGCTCAAATTTGT
AATCT-G-GCG-GTCA-TCTGGTCGTCTGAGTTGTAATCTGTAGAGGCATCAT-CTGTGTCG-
GCCCGTGTACAAGTCTCCTGGAATGGAGCGTCATAGAGGGTGAGAATCCCG-
TCCATGACACGG-AC-CGCCGATGC-
TCTGTGAGGTGCCCTCGAAGAGTCGCGTTGTTTGGGAATGCAGCGCAAAATGGGTGGTAAATTC
CATCTAAGGCTAAATATTGGCGAGAGACCGATAGTGAACAAGTACCGTGAGGGAAAGATGAAA
AGCACTTTGGAAAGAGAGTTAAACAGTACGTGAAATTGTTGAAAGGGAAACGCTTGAAGTCAG
TCGCGTCCCGAGGCACTCAGCCTTGC----TTCGGCTTGGTGTACTTGCCTT-G---
AGATGGGTCAGCATCAA-TTTT-GGC-
TGGTGGACAAGGTTGAGGGGAATGTAGCGTTGTTTCGATGACGTG-TTATAGC-TCCTTATC-
GAAT-ACACTGGTCGGGATTGAGGA-TTGCAGCACGCCC------TT-G-TGGTC-------
AGGGG----------------------TT---CG-CCCCATG-T-AACGTGCTTAGGAT-
GTTGGCATAATGGCTTTAAGCGACCCGTCTTGAAACACGGACCAAGGAGTCTAACATGCTTGCG
AGTGTTTGGGTGGAAAACCCTTGCGCGTAATGAAAGTGAAA-GTTGAGAG-----CCTC-------
-----------TATAAAAGGAGGTGCACTGACGCCCGGCCCTGACG-----
TTCTCTAATGGTGCTGCGGTAGAGCAAGTATGTTGGG----
ACCCGAAAGATGGTGAACTATGCCTGAATAGGGCGAAGCCAGAGGAAACTCTG-
GTGGAGGCTCGTAGCGATTCTGACGTGCAAATCGATCGTCAAATTTGGG------------------
----

ATTTCCGTTGGTTCTTTATCCGCTCCGGTACTCGAATTCTTGGAGGAATGGGGTTTGGAGAGCCT
CGAGGAAAATGCACA---AGCTGCAA------
ATTTGACCAAAGTTTTCGTGAATGGGGTATGGCTCGGAGTACACCGAGACCCGCACACACTTGT
CAACACACTTCGAACACTTAGGAGAAAGG---
AGGATCTCAACGCTGAGGTTTCCATTGTTCGTGACATACGCGAAAAGGAAGTTCGCATCTATAC
GGACGCAGGACGCGTTCTTCGACCGCTTTTCATTGTCGAGGACAATAGATTAAAGCTGAGACGT
CAGCACCTGCGTTGGCTTCAGGCGAAGTGGAGAATT-------

GAGGAGAGGCCCAAAGACAGGGTCCT------------
GGATGAAGGCGAAGAATCAGCAGGCGAGACAATAACTGAGCTAGAGGTCGATGGGAAGCCAA
TAATACGGGAAGGGGTTCGAATGAAGAAGAAGAGGAAACCGGTCACCTGGAGGACGTTGATTA
GCGACGGCGTTATCGAATACCTTGATGCAGAGGAGGAGGAAACAGTCATGATCTGCATGACAC
CAGAAGATCTAGTCGAATCTCGGTTAATGAGGCAAGGCCTGGATCCCCG---
AGCAGACACGGAATTCGATGCTTCTACGCGTCTCAAGCCTGTACCGACT------
GCTCATAACTGGACACATTGCGAAATTCACCCCAGCATGATTCTAGGTATATGTGCCAGTATCAT
TCCATTCCC
>Phylloporia_tiliae_Yuan_5491

TCCCCTAGTAACTGCGAGTGAAGCGGGAAGAGCTCAAATTTGTAATCT-G-GCG-GTC--
TCTGGCCGTCCGAGTTGTAATCTGTAGAGGCGTCGT-CCGCGTCG-
GCCCGTGTACAAGTCTCCTGGGATGGAGCGTCATAGAGGGTGAGAATCCCG-
TCCATGACACGG-AC-CACCGATGC-
TCTGTGAGGTGCCCTCGAAGAGTCGCGTTGTTTGGGAATGCAGCGCAAAATGGGTGGTAAATTC
CATCTAAGGCTAAATATTGGCGAGAGACCGATAGCGAACAAGTACCGTGAGGGAAAGATGAAA
AGCACTTTGGAAAGAGAGTTAAACAGTACGTGAAATTGTTGAAAGGGAAACGCTTGAAGTCAG
TCGTGTCCCGTGACATTCAGCCTTGC----TTCGGCTTGGTGTACTTGCCTT-G---
GGATGGGTCAACATCAA-TTTT-GGC-
CGGTGGACAAGGGCGAGGGGAATGTAGCGTCGTTTCGGCGACGTG-TTATAGC-CTCTTGTC-
GAAT-ACACTGGTTGGGATTGAGGA-CTGCAGCACGCCC------TT-G-TGGCC-----G-
GGGGG----------------------TT---CA-CCTCATG-T-AACGTGCTTAGGAT-
GTTGGCATAATGGCTTTAAGCGACCCGTCTTGAAACACGGACCAAGGAGTCTAACATGCTTGCG
AGTGTTCGGGTGGAAAACCCTTGCGCATAATGAAAGTGAAA-GTTGGGAG-----CCTC-------

-----------CGCAA--GGGGGTGCACCGACGCCCGGCCCTGATG-----
TTCTCTGACGGTGCTGCGGTAGAGCAAGTATGTTGGG----
ACCCGAAAGATGGTGAACTATGCCTGAATAGGGTGAAGCCAGAGGAAACTCTG-
GTGGAGGCTCGTAGCGATTCTGACGTGCAAATCGATCGTCAAATTTGGG------------------
----

>Phylloporia_tiliae_Cui_11306
ACGAGTTTTGA-----A-A---GCGGAG-------------------------------TCTGCTGCTG--
GTGCG---------------GAAACG--TACA-TGTG-CACGGC----------------------------
---TCT--GTG-----CTCGATCC--A--CTC-----------------AA-CCCC--TGTGCA-
CCTTTGCG--AAG-------------------
-------------------------------------CGAGTAGG----------------C------TTCACCC-
----------------------------TTTGAAGG----------
TGGGACCAGTAGTGAGTAAGGCGAGC--
GTGT--
--GCGA--------

---GCATCAGTA------
---------GCCTGAA-------AGCGAGCG----------------------CCCTTCATTT-----------

AGAGAG-GGTGCGGTCGAAAGC-TTTGA------TCTTT----AT-------------CA---
ACCTTTTGT-ATGTCATGTGAAT--G-----T-GTT-GC-T-CC-TC----GTGGGCGAAAT-------
AAATACAACTTTCAACAACGGATCTCTTGGCTCTCGCATCGATGAAGAACGCAGCGAAATGCGA
TAAGTAATGTGAATTGCAGAATTCAGTGAATCATCGAATCTTTGAACGCACCTTGCGCCCTTTGG
-TATTCCGAAGGGCATGCCTGTTTGAGTGTCACGTTTATCTCAAACCTCC-------AAG-
CCTTTTG--------------TTA----GGTTT----CGG-ACGGTTTGGAC-TTGGAGG-C-C------
TTTGCTGGT-GTGC---------------GCTCT--GCGTGTGTCGGCTCCTCTGAAAAGCATTAGC-
TGGACTTTGG----------------T-TT---GCG-TGC-----------ATGGTGTGATAGTAA------
---------TGTG-TTCAC----CGGAAG-CGCTTG-CCTGA-CGGGTCTGC-TCC-----
TAATCGTCTT------------------CGGAC-GA--------GG-TCC---TTCGT-GAC----C-
TTCGCTTGACCGTTTGACCTCATCCCCTAGTAACTGCGAGTGAAGCGGGAAGAGCTCAAATTTG
TAATCT-G-GCG-GTC--TCTGGCCGTCCGAGTTGTAATCTGTAGAGGCGTCGT-CCGCGTCG-
GCCCGTGTACAAGTCTCCTGGGATGGAGCTTCATAGAGGGTGAGAATCCCG-
TCCATGACACGG-AC-CACCGATGC-
TCTGTGAGGTGCCCTCGAAGAGTCGCGTTGTTTGGGAATGCAGCGCAAAATGGGTGGTAAATTC
CATCTAAGGCTAAATATTGGCGAGAGACCGATAGCGAACAAGTACCGTGAGGGAAAGATGAAA
AGCACTTTGGAAAGAGAGTTAAACAGTACGTGAAATTGTTGAAAGGGAAACGCTTGAAGTCAG
TCGTGTCCCGTGACATTCAGCCTTGC----TTCGGCTTGGTGTACTTGCCTT-G---
GGACGGGTCAACATCAA-TTTT-GGC-
CGGTGGATAAGGGTGAGGGGAATGTAGCGTCGTTTCGGCGACGTG-TTATAGC-CTCTTGTC-
GAAT-ACGCCGGTTGGGATTGAGGA-CTGCAGCACGCCC------TT-G-TGGCC-------
GGGGG----------------------TT---CG-CCTCATG-T-AACGTGCTTAGGAT-
GTTGGCATAATGGCTTTAAGCGACCCGTCTTGAAACACGGACCAAGGAGTCTAACATGCTTGCG
AGTGTTCGGGTGGAAAACCCTTGCGCATAATAAAAGTGAAA-GTTGGGAG-----CCTC-------
-----------CGTAA-GGGGGGTGCACCGACGCCCGGCCCTGACG-----
TTCTCTGACGGTGCTGCGGTAGAGCAAGTATGTTGGG----
ACCCGAAAGATGGTGAACTATGCCTGAATAGGGTGAAGCCAGAGGAAACTCTG-
GTGGAGGCTCGTAGCGATTCTGACGTGCAAATCGATCGTCAAATTTGGG------------------
----

>Phylloporia_sp_Dai_9242
ACGAGTTTTGA-----A-A---GTAAAG------------------------------TCTGATGCTG--
GCGTG---------------GAGACG--CGCA-TGTG-CACGGC----------------------------
---TTT--GCG-----CTCAATCT--A--CTT-----------------AA-CCCT--TGTGCA-
CTTTTGTA--AGG--------------------
-----------------------------------CGAGTAGG----------------C------TTTGCTC--
--------------------------TCTGAAG------------
CAACCGAGTCGTAAGTAAGGCTGAGG---
GTGC--
--AGTGAGCACG--
------------------------AGTAGC-CTGAAATCCA---------------------------------
ATGGTGCT--TTGGTGTTGAAA--GTGTTCATTAGCGAGTAAGG---TGAGCGTGCGCAAGCA---
-TTAGTAGCCTGAAAGCGAATGGTGCTTTGGTGTTGTGTTGGAAGCGTTC-CTTAGCGAGTAA-
GGTG-------------AATAGAGTT---------------GCCTGAA-------AGCGAGGG--------
-------------ACCTTCATTT-----------AGAG---AGCATGGTCGAAAGC-TTTGA-----
CTTTTT----AT-------------CA---ACCTTTTGT-ATGTCTTGTGAAT--G-----T-GTA-GC-T-
CC-TC----GCGGGCGAAAG-------
AAATACAACTTTCAACAATGGATCTCTTGGCTCTCGCATCGATGAAGAACGCAGCGAAATGCGA
TAAGTGATGTGAATTGCAGAATTCAGTGAATCATCGAATCTTTGAACGCACCTTGCGCCCTTTGG
-TATTCCGAAGGGCATGCCTGTTTGAGTGTCACGTTAATCTCAAACCTC--------AAG-
CCTTTTG--------------TGA----GGCG-----CTG-ATGGTTTGGAG-TTGGAGG-C--------
TTTGCTGAC-AAGAGCG-----------CCTTTT--
AGGTGTGTCGGCTCCTTTAAAAAGCATTAGC-TGGGCTCTGG----------------T-TC---
GCA-CGG-----------ATGGTGTAATAGTAA---------------AGTG-TTCGC----CAGAAT-
GGCATT-CCTAACGGGGTCTGC-TTC-----TAATCGTCTT------------------CAGAC-AA---
-----GG-TCC---CTTAC-GAC----C-
TTTGCTTGACCGATTGACCTCATCCCCTAGTAACTGCGAGTGAAGCGGGAAGGGCTCAAATTTG
TAATCT-G-GCG-GTC--TCTGGCCGTCTGAGTTGTAATCTGTAGAGGCGTCGT-CCGCATCA-
GCCCGTGTACAAGTCTCCTGGAATGGAGTGTCATAGAGGGTGAGAATCCCG-
TTCATGACATGG-AC-CACTGGTGC-
TTTGTGAGGCGCCCTCAAAGAGTCGCGTTGTTTGGGAATGCAGCGCAAAATGGGTGGTAAATTC
CATCTAAGGCTAAATATTGGCGAGAGACCGATAGCGAACAAGTACCGTGAGGGAAAGATGAAA
AGCACTTTGAAAAGAGAGTTAAACAGTACGTGAAATTGTTGAAAGGGAAACGCTTGAAGTCAGT
CGCGTCTTGAGGCATTCAGCCTTAC----TTCGGTTTGGTGTACTTGCCTT-G---

GGACGGGTCAACATGAATTTTT-GGC-
TGGTGGACAAGGATGAGGGGAATGTAGCGTCGTTTCGACGATGTG-TTATAGC-CTCTTGTC-
GAAT-ACGCTGGCTGGGATTGAGGAACTGCAGCACGCCC-----TTC-G-TGGCC-------
AGAGG----------------------TT---CG-CCTCATG-T-AACGTGCTTAGGAT-
GTTGGCATAATGGCTTTAAGCGACCCGTCTTGAAACACGGACCAAGGAGTCTAACATGCTTGCG
AGTGTTCGGGTGGAAAACCCTTGCGCATAATGAAAGTGAAA-GTTGGGAG-----CCTC-------
-----------TGTAA--GGAGGTGCACCGACGCCCGGCTCTGACG-----
TTCTCTAATGATGCCGCGGTAGAGCAAGTGTGTTGGG----
ACCCGAAAGATGGTGAACTATGCCTGAATAGGGCGAAGCCAGAGGAAACTCTG-
GTGGAGGCTCGTAGCGATTCTGACGTGCAAATCGATCGTCAAATTTGGG------------------
----

>Phylloporia_sp_Dai_18366
ACGAGTTTTGA-------A---GTAAAG------------------------------TCTGATGCTG--
GCGTG---------------GAGACG--CGCA-TGTG-CACGGC----------------------------
---TTT--GCG-----CTCAATCT--A--CTT-----------------AA-CCCT--TGTGCA-
CTTTTGTA---AGG--------------------
-----------------------------------CGAGTAGG----------------C------TTTGCTC--
--------------------------TCTGAAG------------
CAACCGAGTCGTAAGTAAGGCTGAGG--

GTGC---
---AGTGAGCACG--
------------------------AGTAGC-CTGAAATCCA---------------------------------
ATGGTGCT--TTGGTGTTGAAA--GTGTTCATTAGCGAGTAAGG---TGAGCGTGCGCAAGCA---
-TTAGTAGCCTGAAAGCGAATGGTGCTTTGGTGTTGTGTTGGAAGCGTTC-CTTAGCGAGTAA-
GGTG-------------AATAGAGTT---------------GCCTGAA-------AGCGAGGG--------
-------------ACCTTCATTT-----------AGAG---AGCATGGTCGAAAGC-TTTGA-----
CTTTTT----AT-------------CA---ACCTTTTGT-ATGTCTTGTGAAT--G-----T-GTA-GC-T-
CC-TC----GCGGGCGAAAG-------
AAATACAACTTTCAACAATGGATCTCTTGGCTCTCGCATCGATGAAGAACGCAGCGAAATGCGA
TAAGTAATGTGAATTGCAGAATTCAGTGAATCATCGAATCTTTGAACGCACCTTGCGCCCTTTGG
-TATTCCGAAGGGCATGCCTGTTTGAGTGTCACGTTAATCTCAAACCTC--------AAG-
CCTTTTG--------------TGA----GGCG-----CTG-ATGGTTTGGAG-TTGGAGG-C--------
TTTGCTGAC-AAGAGCG-----------CCTTTT--
AGGTGTGTCGGCTCCTTTAAAAAGCATTAGC-TGGGCTCTGG----------------T-TC---
GCA-CGG-----------ATGGTGTAATAGTAA---------------AGTG-TTCGC----CAGAAG-
TGCATT-CCTAACGGGGTCTGC-TTC-----TAATCGTCTT------------------CAGAC-AA---
-----GG-TCC---CTTAC-GAC----C-TTTGCTTGACCGATTGACCTCA--------------------
----

>Coltricia_abieticola_Cui_10321
GAGTTCTTTGA-----A-G--GCGGGAGTTTACAACAATTGTCCCTT-------
TGCCTCTGATGCTG--GCAGCAATAAATC-------TTTA-G-TTGCA-TGTG-
CTCGAGGTGCATTC---------GGGACAATGGAGTGCTCCCAAG-----TTCAAGTCT-G--
CTCATCTTGCTTGTTTTTTTAA-CACC--TGTGCA-CCATAAGCAAAAG---------------------
--------TACTATTCACTTTGG--
GTCAGCTTTTTGTGAGTGATA---------------------ACATGTGC-----------------------
-----ACTGTTGTACACTTTTCTCTAGTCTTATAGGCAGAGGAGTGTAT---------------------
----

------ATAGTAATTGTGTGTC--------------------------CATGCAATGAT--------------
----------------------------CTGCTGAGGGATC-----------------------------------
----------GACACATCCCAAA--GCGGGTAA-
---------------------TAGCA------------TTGATTTACAT------GGTGTATAATGT------
---------TTATATCCACACAAAGATG----TGCTCTTGAGTGTTATGGGAAC-TTTGT------
TTTTGATATAT----ACA------AA---CTCTTGTAT-CTGTCTTGTAGAAT-G-----A----------
-----A---ATGGGAAAAGT------
TTATTACAACTTTCAACAATGGATCTCTTGGCTCTTGTATCGATGAAGAACGCAGCGAAATGCGA
TAAGTAATGTGAATTGCAGAATTCAGTGAATCATCGAATCTTTGAACGCACCTTGCACTCCTTGG
GTCACCTATGGAGTATGCCTGTTTGAGTGTTATATTGATATCAATTCCC--------TCA-
GGTTTTTGT-----------AATGA--AGGCCTG--TTAG-GTGGATTGGAT-TTGGAGG-T-T----
TTAGTGCAGGT-CACCCCCC-------------------------TCTGCCATTCTATGATA---------
----

TCCCCTAGTAACTGCGAGTGAAGCGGGAAGAGCTCAAATTTGAAATCT-G-GCA-GCTC----
TGCTGTCCGAGTTGTAATCTGTAGAAGTGTTTT-CTGCATTG-
GACCGTGTATAAGTCTCTTGGAACAGAGCGTCATAGAGGGTGAGAATCCCG-
TCCATGACACGG-AC-TACCAATGC-
TTTGTGATACACTCTCAAAGAGTCGAGTTGTTTGGGAATGCAGCTCAAAATGGGTGGTAGACTC
CATCTAAGGCTAAATACAAGCGAGAGACCGATAGTGAACAAGTACCGTGAGGGAAAGATGAAA
AGCACTTTGGAAAGAGAGTTAAACAGTATGTGAAATTGTTGAAAGGGAAACACTTGAAGTCAGT

CACATCCTGTAGGACTCAGCCTTGCCCTTAGGGACTTGGTGTATTTCTTGC-A---
GGATGGGTCAACATCAA-TTTT-GGT-CAGTGGATAAAGGAGAAGGGAATGTAGCAC--
CTTTGCAGGTGTG-TTATAGC-CCTCCTTC-ATAT-ACATTGGCTGGGATTGAGGA-
CTGCAGCATGCCC------CT-C-TGGGG-------CCAGG----------------------CAA--CA-
TATCATGTT-CACATGCTTAGGAT-
GTTGACAAAATGGCTTTAAGTGACCCGTCTTGAAACACGGACCAAGGAGTCTAACATGCCTGCG
AGTATTTGGGTGGAAAACCCTTATGCATAATGAAAGTGAAA-ATTGGGAA-----CCCTTTA----
-----------TTATG--GGGGGTGCACTGATGCCCGGCCCAGATG-----
TTTACTGACGGTGCTGCGGTAGAGCATGTATGTTGGG----
ACCCGAAAGATGGTGAACTATGCCTGAATAGGGTGAAGCCAGAGGAAACTCTG-
GTGGAAGCTCGTAGCGATTCTGACGTGCAAATCGATCGTCAAATTTGGG-----------------
---------GTAAGGGAAAAT-AATGATATTACCTTACTATGAGTGTCGTCCAAAT-
CTCGTGCCAGAAGACTCGGTAAGGCGAGAGACGCAAACGTTAATCGTCTTGATCAGGCGTAAA
GGGTAAGTAGGCGGTGTAAATAATTATA-TTTAAAGTTTACTATTTT----------------------
---------ATAAATTAG---
---ATATAAAATA-
TTTAAATATAATTTAATTAAACTAGAATCTAAAAGAAGTTA----AAATTTAGCTTAAA--
GTAAGAGTGAAATCTTTAGATAATAAGCGATGTTTCTTACGGCGAAGGC-
ATTTAACTAATAAAGATTGACGCTGAGGAACGAAAGTTTGGGCAGAAAAAAGGATTAGAGACC
CTTGTATTCCAAACCGTAAACGATGAATGGCTGTCTCTAAGATTGA----------------------
---------------ATCTTAGTTTCATAGTTAACACG-
TGACCATTCCGCCTTGTAAGTACGATTGCAAAGTTGAAAACAAAAAAATTAGTCGATTTCGGAG-
-CAAACGGAGTGAAGC-ATGTTATTTA---
GGTACTGGTGAATTCGAAGCTGGTATTTCCAAGGACGGTCAGACCCGCGAGCACGCTCTTCTTG
CTTTCACTCTTGGTGTCCGTCAACTCATTGTGGCTGTTAACAAGATGGACACGACTAAGGTTTGT
-AACCATCTGAA--TGAGCACA-CAACGATTGTACCAA--CATATCG----------
ATTCTAGTGGTCTGAAGATCGGTTCAATGAAATTATCAAGGAGACGTCGAATTTCATCAAGAAG
GTCGGTTACAACCCAAAGGCTGTTGCATTCGTCCCGATCTCCGGTTGGCACGGTGACAACATGC
TGGAGCAATCCCCCAAGTATGTGTATTTC-AGTCTTTTTCTGCTAAACCTGCC-----ATT----
TACATTTGCAA-TTAAGCATGCCGTGGTACAAGGGTTGGACCAAAGAGACGAAAA-----------
-GTGGTTCTTCG-
AAGGGCTTCACTCTCCTGGAAGCTATCGATTCCATTGAACCTCCCACCCGCCCGTCCGATAAGC
CACTCCGTCTTCCGCTCCAGGATGTGATCTCCGTTGGCTCGTCCTCTGCTCTTGTCATCGAGTTCT
TGGAAGAATGGGGCCTCGAAAGCTTGGAAGAGCATGTCCA---ATCAACGA------
ACCTCACAAAGGTCTTCGTCAACGGGGTTTGGCTTGGTGTTCATCGCGAACCTACGAATCTTGTG
CGTACTCTTCGGATGATGAGGCGGAGGG---
AAGATATTAGTACCGAGGTTTCGATCGTGCATGATATTCGCGAAAGAGAGCTACGAATCCAGAC
CGATGCTGGTCGGGTCTTGAGACCGATATTCATTGTGGAAAATCATAAACTCAATCTCAAGCCA
CAACACCTCAGATGGCTGCAGACAGGAAGTCGCATC-------GAGACTAGACCATTCGTA----
--CCTGATGATGAAAGAGAGGCGGAATATCAGTCTGACGGCGAGACCGTGGTCGAT---
GAGGAGACTGGACAGAAGGCTATAGATTTCGAAAACAGGGTAAAGAAGAAGGTCAAACCTTAT
GGTTTCAAAGGTTTGGTTACAGATGGCGTAATTGAGTATCTAGACGCTGAAGAGGAGGAAACCG
TCATGATATGCATGACGCCAGAAGAGCTTGCAGAATCCCGGCTTCTACGCCAAGGCCTGGATCC
TCG---GGCGGATACCGATTTTGACCCAGCAGCGCGTCTCAGATCTCTTCCAGTT------

GCTCATTCATTTACCCATTGCGAGATTCATCCAAGTATGATCCTCGGAATTTGTGCTAGTATCATT
CCTTTCCC
>Coltricia_austrosinensis_Dai_13093
---GTCTTTGA-----A-G--
TGTTGGGTTTGCATCCTTTGTCTTTTGATATCCCCCTCTGATGCTG--GCAACTTAAAACT------
---------TGCA-TGTG-CTCGAGGTTCTTT---------AGGCAAAAGAGGCACCTCTCACT-----
TTCAAATTT-A--CTCCATTC-----TTATTTTAA-CACC--TGTGCA-CTAATAAACAGTG--------
---------------------TTCTGTTCATTGGGG--
----TCAGCCTTTGTCTGGTGAA----------------------TATGTGTATT-----------------
----

GTGGGTCCTGTATGAT---
--------------------CAGCAGTGGGATT---
--AACATATCCCAAA--GTGGATAA----------
------------TAACA------------GGGATCTGCATA-----TATGCATAT-----------------
-TTTATTCTCGC----AGA----GATGGC-TCAAGTGAATATGGC-TTTGT------TTTCAACATAC-
---AATTAACTCTT---GTCATATAT-ATGTCTTGTGGAAT-G-----A-AAT-GC-TCCAATT----
GTGAGCAAAGATGGTTATATATATAACTTTCAACAATGGATCTCTTGGCTCTTGCATCGATGAAG
AACGCAGCGAAATGCGATAAGTAATGTGAATTGCAGAATTCAGTGAATCATCGAATCTTTGAAC
GCACCTTGTGCTCCTTGGGTTACCTATGGAGCATGCCTGTTTGAGTGTCATATTAATCTCAGTTCC
C--------TCA-GGTCTTTTTTTAA-------CATGA--AGACCTGC-TCAG-GTGAATTGGAC-
TTGGAGG-T------TCTATGCAGGT-TCCCTTGT-----------AATTA--
TGGGAAATCAGCTCCTCTGAAAAGTATTAGC-TGGAGGATGTAGCAAAAGGGCTCTGGT-TT-
TAGCA-TTGTTTCATTTGCTATGGTGTAATAA--

TCCCCTAGTAACTGCGAGTGAAGCGGGAAGAGCTCAAATTTAGAATCT-G-GCA-GCT--
CTCTGCTGTCCGAGTTGTAATCTGTAGAAGCGTTTT-CTGCATTG-
GACCGTGTACAAGTCTCTTGGAACAGAGTGTCATAGAGGGTGAGAATCCCG-
TCCATGACACGG-AC-TACCAATGC-
TTTGTGATACGCTCTCAAAGAGTCGAGTTGTTTGGGAATGCAGCTCAAAATGGGTGGTAAATTCC
ATCTAAGGCTAAATACAGGCGAGAGACCGATAGTGAACAAGTACCGTGAGGGAAAGATGAAA
AGCACTTTGAAAAGAGAGTTAAACAGTACGTGAAATTGTTGAAAGGGAAACGCTTGAAGTCAGT
CACATCCTGCAGGACTCAGCCTCATC--AACAGATGTGGTGTACTTCCTGC-A---
GGATGGGTCAACATCAA-TCCT-GGCTCAATATATATGAGGGGGGGTGG------------------
--------------TCCCCTTC-ATGT-ACATTGGCTGGGATTGAGGA-CTGCAGCATGCCC-----
CTT-G-TGGCC-------AGGAC----------------------TT------TATCATG-T-
CACATGCTTAGGAT-
GTTGACAAAATGGCTTTAAGCGACCCGTCTTGAAACACGGACCAAGGAGTCTAACATGCCTGCA
AGTGTTAGGGTGGAAAACCCTTGTGCATAATGAAAGTGAAA-GTTGGGAA-----CCTC-------
-----------TGCAA--GGGGGTGCACCGATGCCCGGCCCAGATG-----
TTTACTGACGGTGCTGCGGTAGAGCATGTATGTTGGG----
ACCCGAAAGATGGTGAACTATGCCTGAATAGGGTGAAGCCAGAGGAAACTCTG-

GTGGAGGCTCGTAGCGATTCTGACGTGCAAATCGATCGTCAAATTTGGG------------------
---------GTAAGGGAAAAT-AATGATATTACCTTACTATGAGTGTCGTCCAAAT-
CTCGTGCCAGAAGACTCGGTAAGGCGAGAGACGCAAACGTTAATCGTCTTGATCAGGCGTAAA
GGGTAAGTAGGCGGTTTAAATAATTCTC-TTTAAAGTTTACTATTTT----------------------
---------ATAGTTTAGAA---
---ATATATAAAATA-
TTTAAATATAATTTAATTAAACTAGAATCTAATAGAAGTTA----AAATTTAGCTTAAA--
GTAAGAGTGAAATCTTTAGATAATAAGCGATGTTTCTTACGGCGAAGGC-
ATTTAACTAATAAAGATTGACGCTGAGGAACGAAAGTTTGGGCAGAAAAAAGGATTAGAGACC
CTTGTATTCCAAACCGTAAACGATGAATGGCTGTCTCTAAGATTGA----------------------
---------------ATCTTAGTTTCATAGTTAACATG-
TGACCATTCCGCCTTGTAAGTACGACTGCAAAGTTGAAAACAAAAAAATTAGTCGATTTCGGAG
--CAAACGGAGTGAAGC-ATGTTATTTA---
GGTACTGGTGAATTCGAAGCTGGCATTTCCAAGGACGGCCAGACCCGCGAGCACGCTCTTCTTG
CATTCACCCTCGGTGTCCGTCAGCTTATTGTCGCAGTCAACAAGATGGACACAACTAAGGTTTGT
AAAGCCTTTGAA--TGAAC-CTCTGACCCTCGAACTTG--
ACGATCAACTACTCTTTTTAAAAGTGGTCTGAGGATCGATTCAATGAAATTGTTAAAGAGACATC
GAATTTCATCAAGAAGGTCGGTTACAATCCGAAGGCTGTTGCTTTCGTTCCTATCTCTGGTTGGC
ACGGTGATAACATGCTGGAATCTTCCGCCAAGTAGGTGTATTTTCTGTCGTTCTTTGCCAAGTTC
AAGTCATTATAACAACTCGCTCGCCC-
TTTAGCATGCCGTGGTACAAGGGCTGGACCAAAGAGACGAAGG-----------
GTGGCGTTGTT-
AAGGGTGTCACTCTCCTGGACGCTATCGACTCCATTGAACCTCCTAGTCGCCCCGTCGACAAGC
CACTTCGCCTGCCCCTCCAGGATGTGATCTCTGTTGGCTCGTCCTCTGCTCTTGTCATCGAGTTCT
TGGAAGAATGGGGTCTCGAAAGCTTGGAAGAGAATGTCCA---ATCTATTA------
ACCTTACAAAAGTATTCGTTAACGGGGTGTGGCTCGGCGTTCATCGCGAGCCTACCAACCTTGT
GCGTACTCTTCGAATGATGAGGCGAAGAG---
AAGATATCAGCACCGAGGTTTCAATTGTGCATGATATTCGCGAACGAGAATTGCGAATCCAGAC
AGATGCTGGTCGAGTCTTGAGACCTATATTCATTGTGGAGAATCATAAACTCAATCTCAAGCCG
CGGCATCTTAGATGGCTGCAGGCAGGAAGTCGCATC-------GAGACCAGACCGTATGTG----
--CCAGACGATGAAAGGGAGGCCGAGTATCAATCCGATGGCGAGACCGTTGTAGAC---
GAGGAGACCGGACAGAAGGTTATCGATATCGACAACAGGGTAAAGAAGAAAGTCAAGCCCTA
TGGTTTCAAAGGTTTGGTCACGGATGGCGTAATTGAATACCTAGATGCTGAAGAGGAAGAAACC
GTCATGATATGCATGACGCCTGAAGAGCTCGCAGAATCTCGACTTCTTCGTCAAGGCCTTGATC
CCCG---GGCGGACACCGATTTTGATCCGGCAGCACGTCTCAGGTCTCTTCCAGTT------
GCCCATTCATTTACGCATTGTGAAATTCATCCAAGCATGATCCTCGGAATTTGCGCCAGCATCAT
TCCTTTTCC
>Coltricia_baoshanensis_Dai_13075
---TTCTTTGA-----A-GTGTGGTTGTCTTATATGTCTCACCTTTT----TGTTGTCTTTGATGCTG--
GCAATAGCAATTTGTTGTT--------GCAAA-TGTG-
CTCAAGGCTTTTTCTTGAAGGAGGAGCAAAAATGGA-CACCTGCT----TTTTGAGATT-G--
CTCATGTTCCT--TTAACACAA-CCCCCCTGTGCA-CTATAAGCAAAGC--------------------
---------TTTTGTTCCTGCAAG---
TCAAAATCTATGTTGTGAT-----------------------TTAGCATCTT---------------------

--ATTTGTTGCATAATGGGGCCCTCTATCACATATTGACCAGCTCAA----------------------
----

-----GGAATCATTATGTT--
------------------------------TAGTAAC---
----AAAATATTCCAAA---------------------------------------
GTGAAATGGGGAGT------------ATGTGTATG------------GGGTTTTGCACTAT--
ATATGCATGCA-----------------TATAGTCACAAAAGATGACTTT-------
AATGGGGATATTGGC-TTTGT------CTTCAT-ATAA----AAC------CC---CCCTTGTAA-
TTGTCTTGTACAAT-G-----AGAATGGC-T-CAATT----GTGAGCAAAAA-----
ATTCATACAACTTTCAACAATGGATCTCTTGGCTCTTGCATCGATGAAGAACGCAGCGAAATGC
GATAAGTAATGTGAATTGCAGAATTCAGTGAATCATCGAATCTTTGAACACACCTTGTGCTCCTT
GGGTTACCTATGGAGCATGCCTGTTTGAGTGTCATGTTAATCTCAATTCCC--------TCA-
GGTATCTCTTGTACAAACAAGAGAA--GGGCCTGCTCAGG-GTAAATTGGAT-TTGGAGG-T-T-
---TTTATGCAGGG-TCCCCTCT-----------
CTTTGTAGGGGGAATCAGCTCCTCTTAAAAATATTAGC-TGGAGGGATAATGCAAAATGAC----
T-TCTTAGCA-TTG-TTCATTGGCTATGGTGTAATAATTGT------------------TTCATCAT---
----

TCCCCTAGTAACTGCGAGTGAAGCGGGAAGAGCTCAAATTTAAAATCT-G-GCA-GCTT----
TGCTGTCCGAGTTGTAATCTGTAGAAGCATTTT-CTGCATTG-
GACTGTGTACAAGTCTCTTGGAATAGAGCGTCATAGAGGGTGAGAATCCCG-
TCCATGACACAG-AC-TACCAATGC-
TTTGTGATATGCTCTCAAAGAGTCGAGTTGTTTGGGAATGCAGCTCAAAATGGGTGGTAAATTCC
ATCTAAAGCTAAATATAGGCGAGAGACCGATAGCGAACAAGTACCGTGAGGGAAAGATGAAA
AGCACTTTGGAAAGAGAGTTAAACAGTACGTGAAATTGTTGAAAGGGAAACGCTTGATGTCAGT
TTCATTCTGCAGAACTCAGCCTTGCCT-ATTAGGCTTGGTGTATTTTCTGC-A---
GGATGGGTCAACATCAA-TTTT-GGT-CAGTGGATAAAGGAGGAGGGAATGTGGCAC--
CTGCGAAGGTGTGTTTATAGC-CCTCTTTC-ATAT-ACATTGACTGGGATTGAGGA-
CTGCAGCATGCCC------TC-T-TTATG-------GGGGG---------------------CCAGGCT-
TTTCATG-T-CACATGCTTAGGAT-
GTTGACAAAATGGCTTTAAGCGACCCGTCTTGAAACACGGACCAAGGAGTCTAACATGCCTGCA
AGTGTTTGGGTGGAAAACCCTTGCGCATAATGAAAGTGAAA-GTTGGGAA-----CTTC-------
-----------CGCAA--GGGGGTGCACCGACGCCCGGCCCTGATG-----
TTTACTGACGGTGCTGCGGTAGAGCATGTATGTTGGG----
ACCCGAAAGATGGTGAACTATGCCTGAATAGGGTGAAGCCAGAGGAAACTCTG-
GTGGAGGCTCGTAGCGATTCTGACGTGCAAATCGATCGTCAAATTTGGG-----------------
---------GTAAGGGAAAAT-AATGATATTACCTTACTATTAGTGTCGTCCAAAT-
CTCGTGCCAGAAGACTCGGTAAGGCGAGAGACGCAAACGTTAATCGTCTTGATCAGGCGTAAA
GGGTAAGTAGGCGGTTTAAACCATTATA-TTTAATATTTACTATTTT----------------------
---------ATAGTTAATAA---

TATATAAAATTATTTAAATATAATTTAATTAAACTAGAATCTAAAATAAGTTA----
AAACTTAGCTTAAA--

GTAAGAGTGAAATCTTTAGATAATAAGCGATGTTTATAATGGCGAAGGC-
ATTTAACTAATAAAGATTGACGCTGAGGAACGAAAGTTTGGGCAGAAAAAAGGATTAGAGACC
CTTGTATTCCAAACTGTAAACGATGAATGGCTGTTACTAAAATTAT-------------------------
--------------TCTTTAGTTACAAAGTTAACACG-
TGACCATTCCGCCTTGTAAGTACGACTGCAAAGTTGAAAACAAAAAAATTAGTCGATTTCGGAG
--CAAACGGAGTGAAGC-TGGTTATTTA---
GGTACCGGTGAATTTGAAGCTGGCATTTCTAAGGATGGTCAAACCCGCGAGCATGCACTTCTTG
CGTTTACTCTCGGTGTTCGTCAGCTCATCGTCGCTGTCAACAAGATGGACACGACTAAGGTTTGT
GTTTATTTAAA---CGAACAAT-GGGAGCCTTGCATTA--AGCATCA----------
ATTCTAGTGGTCTGAGGATCGATTCAACGAAATTGTTAAGGAAACGTCGAATTTCATCAAGAAG
GTCGGCTATAATCCGAAGGCTGTTGCCTTTGTTCCGATTTCTGGTTGGCACGGTGACAACATGTT
AGAAGCATCCCAGAAGTGTGTATGGTTT-TGTCGTTCCTTACTAAGTTCGCT-----GT-----
TGATGTGTGAT-TCCAGCATGCCGTGGTACAAGGGCTGGAACAAGGAGGTTAAAT-----------
-CAGGTGCACAG-
AAGGGCGTGACTCTGATCGATGCTATCGATGCTATTGAGCCTCCAACCCGCCCGTCCGACAAAC
CACTTCGACTTCCGCTTCAGGATGTGATTTCTGTTGGCTCATCTTCTGCTCTTGTCATTGAATTCTT
GGAAGAATGGGGTCTCGAGAGCTTGGAAGAGCATGTCCA---GTCGACCA------
ATCTTACGAAGGTATTCGTTAATGGGGTTTGGCTTGGAGTGCATCGGGAACCTACGAACCTCGT
GCGAACCCTCCGAATGATGAGGAGAAGGG---
AAGATATTAGCACCGAAGTTTCGATCGTCCACGATATTCGAGAAAGAGAGCTACGAATACAGAC
AGATGCCGGTCGAGTCTTGAGACCTCTGTTCATTGTGGAGAATCATAAACTGAATCTTAAGCCG
CAGCATCTCAGATGGTTGCAGGCAAGGAGTCGTATTA----TCGAGGATAGACCGTACATA----
--CCTGATGAGGAAAAGGAAGCCGAGTATCAATCTGACGGTGAAACCGTAGTTGAT---
GAGGATACTGGAGAAAAGGTAATTAATTTCGAGAAGAGAACCAAGAAGAAAGTCAAGCCTTAC
GGGTTCAAAGGCTTGGTAATGGATGGCGTAATTGAGTACCTAGATGCTGAGGAAGAAGAAACA
GTCATGATATGCATGACACCGGAAGAGCTGGCGGAGTATCGACTTCTGCGTCAAGGACTGGAT
CCTCG---GGCAGATACAGACTTTGATCCAGCAGCACGCGTTAGGACGCTTCCAATC------
GCTCATTCGTTTACCCACTGTGAAATTCATCCAAGTATGATACTGGGAATTTGCGCTAGCATTATT
CCCTTCCC
>Coltricia_globosa_Cui_7545
GAGTTCTTTGA-----A----GTGGGGGT--------GCTGTTTACCAGCTTTTTGTCTCTGATGCTG-
-GCAATATTACCTTA-------------TTGCA-TGTG-CTCGAGTCTTTCTA------
GACGAAGTGAAATGGC-CTCCTGCT-----TTCAATTCT-G--CTCAC--------ATTCTTCAA-
CACC--TGTGCA-CCAATAAGCAAAG------------------------------
TTCAGATATGTGAGC---
GGCTATCACTTGTGAATT------------TGT-----ATTTGCAATGT-------------------------
AGTAGTAGTACTGT---

GCAATCCTCA------AT-------------------------ATTACGATTGA----------------------
----------------------CCACCAAGGGGGA---
---TTGACATCCCAAA---GTGGAAAA---------
-G-----------TAATG------------AAGATTTGCAA------
TATGGGTGCAATACTACATGCATATATGCGCACTCACATGAGGGGG----GGTAGC-

TCATTGATGCTGGCC-TTTGT------CTTTTT-ACAC-------------AA---ACTCTATAT-
ATTTGTAGAAAAGGGG----GA-AAT-GC-T-CA-TT----GTGAGCAATAA----
AATATATACAACTTTCAACAATGGATCTCTTGGCTCTCGCATCGATGAAGAACGCAGCGAAATG
CGATAAGTAATGTGAATTGCAGAATTCAGTGAATCATCGAATCTTTGAACACATCTTGTGCTCCT
TGGGTTACCTATGGAGCATGCCTGTTTGAGTGTCATGTTGGTCTCAATTCTC--------TCT-
CGGGTTCTTTGTG--------TAAA--AGACCTGT-CAGA-GCAAATTGGAT-TTGGAGG-T-TT---
TTTATGCAGGT-CCTCTTTA------------------
GGAGAGATCAGCTCCTCTTAAAAGCATTAGC-TGGAGGATAAAGCAAAAATGAC----T-
CTTTAGCA-TTG-TTCATTGGCTATGGTGTAATAATTGT-------------------TCCATCAT----
GGCATTTTGG-ATGCA-AGAGACTTT-TCG-----CAATATTCTCTG-------
CTTCTAATCCTGAC-AG-----------TCC---TTTAT----------
TGACTCTCTCTCTTTGACCTCATCCCCTAGTAACTGCGAGTGAAGCGGGATGAGCTCAAATTTAA
AATCT-G-GCA-GCTC----TGCTGTCCGAGTTGTAATCTGTAGAAGCATTTT-CTGCATTG-
GACTGTGTACAAGTCTCTTGGAATAGAGCGTCATAGAGGGTGAGAATCCCG-
TCCATGACACAG-AC-TACCAATGC-
TATGTGATATGCTCTCAAAGAGTCGAGTTGTTTGGGAATGCAGCTCAAAATGGGTGGTAAATTC
CATCTAAAGCTAAATATAGGCGAGAGACCGATAGCGAACAAGTACCGTGAGGGAAAGATGAAA
AGCACTTTGGAAAGAGAGTTAAACAGTATGTGAAATTGTTGAAAGGGAAACGCTTGAAGTCAGT
CACATTCTGTGGGACTCAACCTTGCCT-ATTAGGCCTGGTGTATTTCCCTGTA---
GGATGGGTCAACATCAA-TCTT-GGT-CAATGGATAAAGGATGAGGGAATGTGGCAC--
CTTTGCAGGTGTG-TTATAGC-CCTCTTTCAATAT-ACATTGGCTGGGATTGAGGA-
CTGCAGCATGCCC------TT-G-TGGCC-------AGGA----------------------------CA-
TTTCATG-T-TACATGCTTAGGAT-
GTTGACAAAATGGCTTTAAGCGACCCGTCTTGAAACACGGACCAAGGAGTCTAACATGCCTGCA
AGTGTTTGGGTGGAAAACCCTTGCGCATAATGAAAGTGAAA-GTTGGGAA-----CCTC-------
-----------TGTAA--GGGGGTGCACCGACGCCCGGCCCTGATG-----
TTTACTGACGGTGCTGAGGTAGAGCATGTATGTTGGG----
ACCCGAAAGATGGTGAACTATGCCTGAATAGGGTGAAGCCAGAGGAAACTCTG-
GTGGAGGCTCGTAGCGATTCTGACGTGCAAATCGATCGTCAAATTTGGG------------------
---------GTAAGGGAAAAT-AATGATATTACCTTACTATTAGTGTCGTCCAAAT-
CTCGTGCCAGAAGACTCGGTAAGGCGAGAGACGCAAACGTTAATCGTCTTGATCAGGCGTAAA
GGGTAAGTAGGCGGTTTAAACCATAATA-TTTAAAGTTTACTATTTT----------------------
--------TATAGTTAATAA--

TTATATTAAATATAATATTTATTATGTAATTAAACTAGAATCTAATATAAGTTA----
AAACTTAGCTTAAA--
GTAAGAGTGAAATCTTTAGATAATAAGCGATGTTTATAATGGCGAAGGC-
ATTTAACTAATAAAGATTGACGCTGAGGAACGAAAGTTTGGGCAGAAAAAAGGATTAGAGACC
CTTGTATTCCAAACCGTAAACGATGAATGGCTGTAACTAAAAATTA----------------------
--------------ATCTTTAATTACAAAGTTAACACG-
TGACCATTCCGCCTTGTAAGTACGACTGCAAAGTTGAAAACAAAAAAATTAGTCGATTTCGGAG
--CAAACGGAGTGAAGC-ATGTTATTTA---
GGTACTGGTGAATTTGAGGCCGGTATCTCCAAGGACGGTCAGACCCGCGAGCATGCTCTTCTTG
CATTCACTCTCGGTGTCCGTCAGCTCATTGTTGCCGTCAACAAAATGGATACGACTAAGGTTTGT

TGTAGTCTTAA---CTAACAAATCGATGTTTTGTATTAACCGTTTCA----------
ATTCTAGTGGTCTGAGGATCGATTCAATGAAATTGTCAAAGAAACATCGAATTTCATCAAGAAG
GTCGGCTACAATCCGAAGGCTGTTCCTTTCGTCCCGATATCCGGTTGGCACGGCGACAACATGT
TGGAGCAATCCGCTAAGTGCGTATATTAC-CATCATGCTCTTCTCGCCGTTGA------------
CGGTTATTTTT-TTAAGCATGCCCTGGTATAAGGGCTGGACCAAGGAGACCAAGT------------
CCGGTGTTGTG-
AAAGGCTTCACTCTTATCGACGCTATCGACGCCATTGAGCCACCAGTCCGTCCGTCAGACAAGC
CTCTCCGGCTTCCGCTTCAAGATGTGATTTCCGTTGGTTCGTCATCTGCGCTTGTCATTGAATTCC
TAGAAGAATGGGGCCTCGAGAGTTTGGAAGAGAATGTCCA---ATCGACGA------
ATCTTACCAAAGTATTCGTTAATGGAGTTTGGCTTGGAGTACATCGCGAACCTACGAACCTTGTG
CGAACTCTTCGAATGATGAGGAGAAGAG---
AGGATATTAGCACCGAGGTCTCTATCGTTCACGATATTCGAGAAAGAGAGTTGCGAATCCAGAC
CGATGCCGGTCGGGTCTTGAGACCTTTGTTTATCGTTGAGAATCATAAACTCAATCTTAAGCCAC
AGCATCTCAGATGGCTGCAAGCAGGAAGTCGAATT-------GAGACTAGACCTTACATA------
CCCGATGATGAAAAGGAAGCTGAGTATCAATCGGACGGCGAAACTGTCGTCGAT---
GAGGATACTGGAGAAAAGGCAATTGATGTGGAAAACAGGGTCAAGAAGAAGGTCAAGCCTTA
CGGATTCAAAGGCTTGGTTACGGACGGCGTAATCGAGTACCTGGACGCTGAGGAGGAAGAAAC
TGTCATGATATGCATGACACCAGAAGAGCTGGCAGAGTCTCGACTTCTACGCCAAGGTCTAGAT
CCTCG---AGCAGACACAGA---

>Fomitiporella_chinensis_Cui_11230
ACGAGTCTTGT-----A-C--GCAAGAG-------------------------------CTTGTAGCTG--
GCGCG----------------GAGACG--CGCA-TGTGTCACGGC-----------------------------
---TCT-TGTG-----CTCAATCC--A--CTC------------------AA-CCCC--TGTGCA-
CCTATGCG--AAG--------------------
--------------------------------------CTAGTAGC-----------------T------
TTCTCTTTTCT------------------------TGTTCTTCTGCCCCGA--------------------------
---CGGCTGTAC---------------------------

------------------------------------GTGGGC---------------------------------------
--GGGCCT---GCCTTGT------------------

-------------------------------------TGCGAGGT--------------
TGGTTCGCTTAGTTCACGGAGGGGTATATA----------------------------------GA-
AGGAGGAG-GAGGGGGGCGAACGC-TTTGA-------TCTTA-TTAC--------------AA---
ACCCATTGTAATGTCCTGTCGAAT-G-----T-AAT-GC-C-CC-TC----GTGGGTGAAAT------
GAAATACAACTTTCAACAACGGATCTCTTGGCTCTCGCATCGATGAAGAACGCAGCGAAATGCG
ATAAGTAATGTGAATTGCAGAATTCAGTGAATCATCGAATCTTTGAACGCACCTTGCGCCCCTTG
G-TATTCCGAGGGGCATGCCTGTTTGAGTGTCATGTTAATCTCAAACCCC--------AAG-
CCTTTCTTAAC----------CGAA--GGGCT-----TGA-TCGGTTTGGAC-TTGGAGG-T-T------
GATGCTGGC-TTCA--------------CTCTCTT--TGTGTGGTCGGCTCCTCTAAAATGCATTAGC-
CGGACTTTGG-----------------T-TC---GCATCTT-----------
ACGGTGTAATAGTAACAAAAAG--------CTTG-TTCGCC------GGG-TGCTTG-CCTAA-
GGAGTCCGC-TTC-----TAATCGCCCCC-----------GTTTCCGGGGC-AA--------GG------

AGCAAT-------CCTTTTCATGAACACTTTGACCTCA--
CCCTAGTAACTGCGAGTGAAGCGGGAAGAGCTCAAATTTAAAATCT-G-ACG-GCC--
TCTGGCCGTCCGAGTTGTAACCTGAAGGGGCGTTTT-CCGCGTCG-
GACCGTGTACAAGTCTCCTGGAACGGAGCGTCATAGAGGGTGAGAATCCCG-
TCCATGACACGG-AC-GCCCGATGC-
TTTGTGATACGCCCCCGAAGAGTCGCGTTGTTTGGGAATGCAGCGCAAAATGGGTGGTAAATTC
CATCTAAAGCTAAATATTGGCGAGAGACCGATAGCGAACAAGTACCGTGAGGGAAAGATGAAA
AGCACTTTGGAAAGAGAGTTAAACAGTACGTGAAATTGTTGAAAGGGAAACGCTTGAAGTCAG
TCGCGTCCTGTGGAACTCAGCCTTGC----TTCGGCCTGGTGTACTTTCCGC-T---
GGACGGGTCAACATCAA-TTTT-GGC-
CGGTGGACAAGGGCGGGGGGAATGTAGCGTCGCTTCGGCGACGTG-TTATAGC-CCCTCGTC-
GGAT-GCACCGGCCGGGATTGAGGA-CCGCAGCGCGCCC------TT-G-TGGCC-------
GGGGG----------------------TT---CG-CCCCACG-T-ACCGCGCTTAGGAT-
GTTGGCATAATGGCTTTAAGCGACCCGTCTTGAAACACGGACCAAGGAGTCTAACATGCTTGCG
AGTGTTCGGGTGGAAAACCCTTGCGCGCAATGAAAGTGAAA-GTTGGGAG-----CCTC-------
-----------CGCGA--GGGGGTGCACCGACGCCCGGCCCTGACG-----
TTCTCTGACGGTGCTGCGGTAGAGCAGGTATGTTGGG----
ACCCGAAAGATGGTGAACTATGCCTGAATAGGGCGAAGCCAGAGGAAACTCTG-
GTGGAGGCTCGTAGCGATTCTGACGTGCAAATCGATCGTCAAATTTGGG------------------
---------GTAAGGGATAAT-
AATGATAATACCTTACTATTAGTGTTGTCCAAGTGCTCGTGCCAGAAGACTCGGTAAGGCGAGA
GACGCAAACGTTAGTCGTCTTAAACAGGCGTAAAGGGTTTGTAGGCGGCTTTAAGTACCCAA-
TATTAAGTTTACTATTAT--------------------------------AGTTCACTAT--------------
----

GGGAAAAGTGCTATAATATTTTTTAAAGGGTCGTTTAAAGCTAGAATCTAAAAGAGGTTA----
AAATTATGCTTGAC-AACAGGTGTACTAACCGCATACAGCAAGTAGAGTGT-
TAAGGGCGAAGGC-
TTTTTTCCAATGAAGATTGACGCTGAGAAACGAAGGTGAGGACAGAAAAAAGGATTAGAGACC
CTTGTATCCCTCACTGTCAACGATGAATGGTAGTTACTAGGGAGAA----------------------
----
ATAACCTAGAGACGATGTTAACACGAAAACCATTCCGCCTTGTGAGTACGACTGCAAAGTTGAA
AACAAAAAAATTAGTCGGTTTCGGAG--CAAACGAAGTGAAGC-ATGTTATTTA---
GGTACTGGTGAGTTCGAAGCTGGTATTTCGAAGGACGGTCAGACCCGCGAACACGCCCTCTTG
GCCTACACTCTTGGTGTGCGTCAGCTCATCGTTGCCGTTAACAAGATGGACACCACCAAGGTCA
GTGATGTTTCAAAG--TTGGCATCAACCTGGAACTAAATT--TTGTTAT------
CATAATTGCAGTGGTCTGAGGATCGATTCAATGAGATCGTTAAGGAAACTTCTAATTTCATCAAG
AAGGTCGGCTACAACCCGAAGACTGTTGCATTCGTCCCAATTTCCGGCTGGCATGGTGACAATA
TGCTTGAGGAGTCGTCGAAGTATGTTTTTTAG-TCATCAG------GTGATTCAAT----
AATAGACTAATGCGGTATA-
CAAAGCATGCCTTGGTACAAGGGTTGGGCCAAAGAGACCAAGG------------
CCGGTGTTGTC-
AAAGGCAAGACTCTCCTAGACGCTATCGATGCTATTGAACCGCCTGTCCGTCCCGAGAACAAGC
CGCTTCGGCTCCCTCTTCAGGACGTGGTCAGTGTCGGCAAACCTTACGAGCCACTGACTCAATT

CATGTTCGATCGTGGCATGGACCTTCTTGAGGAGTATGACCC---
AGTCGCTACACCAAACGCCACAAAGTGTTTCGTCAACGGAGTCTGGGTCGGAGTCAGTCGCGA
CCCTGGCAACCTGGTCAGCACCATGAGGCAGCTCAGGAACAGCGGTTACGGTATCGACCCCGA
GGTCAGCTTGATTCGGGATATTCGTGATCGAGAATTCCGCATATTCACTGACGCTGGTCGCATCA
TGCGCCCTCTTTTCGTTGTCGAACAGCG-----------------------------
AGTGGGCCACGAGAACAGGGGCAATTTGGCCATCAAGCGTGTGCACATCGATAAGCTCCTC---
---GATGACAAGGAC--
GTCAATGTGCAGGACATGGCACTGCATGAAATGGACGAGC-
AACAGATTGGCTGGCGGGGTCTGATCAAGCAAGGCGTGGTTGAGTACCTCGACGCGGAAGAA
GAGGAGTCTGCAATGATCGTCATGACGCCCCAAGACCTCGAGGAGCATCATCTTGTGAGGACG
GGCCAGAAACTATACTCCATGGACGACGATAGCGATCCGCATAAGCGATTCAAGCCCGCGCCA
AATCCCAACATTCGTAACTTTACACATTGCGAGATTCATCCTAGCATGATTTTGGGAGTGTGTGC
GAGCATCATTCCGTTCCC
>Fomitiporella_sinica_LWZ_20140624-5
ACGAGTTTTGA-----A-A--GCGGAGG------------------------------CTTGTTGCTG--
GTGCG---------------GAAACG--TACA-TGTG-CACGGC----------------------------
---CTT-CGTG-----CTCAATCC--A--CTC-----------------AA-CCCC--TGTGCA-
CCTTGCG---AAG-------------------
------------------------------------CTAGTAGC----------------T------TCTCTCT--
--------------------------CTCTCTATCTGC-------TCGTTCTGTATCCTCTTTGAC-------
-----------------------------------TGGTATGT----------------------------------
----

------------------------------AAGAGCCCT---GCCTTGT--------------------------
----
---------------------GCGGGTTCAAACT-------------
TAACTCGGTTGAAGTGGGGGGGAGGAACAG-------------------------------TG-
CAAGGAGG-TGGTGAAGCGAACGC-TTTGA------TTTTTA-TTAC-------------AA---
ACCCACTGTAATGTCTTGTTGAAT-G-----T-AAT-GC-T-CC-TT----GTGAGCGAAAC-G-----
AAATACAACTTTCAACAACGGATCTCTTGGCTCTCGCATCGATGAAGAACGCAGCGAAATGCGA
TAAGTAATGTGAATTGCAGAATTCAGTGAATCATCGAATCTTTGAACGCACCTTGCGCCCCTTGG
-TATTCCGAGGGGCATGCCTGTTTGAGTGTCATGTTAATCTCAAACCTC--------GGG-
TCTTTTTAA------------CTAG--AGACC-----TGT-TCGGTTTGGAC-TTGGAGG-T-T-----
AACTGCTGGC-TCCC---------------------TTCTGTGGTCGGCTCCTCTGAAATGCATTAGC-
TGGACTTTGG----------------T-TC---GCA-TCC-----------ACGGTGTAATAGTAACT---
----------TGTA-TTCGCC------GGG-TGCTTG-CCTAG-GGAGTCCGC-TTC-----
TAATCGCCCCC--------T--------CGGGC-AA--------GGTCTCC-TTTTGT-GAC----C-
TTC--
TTGAACACTTGACCTCATCCCCTAGTAACTGCGAGTGAAGCGGGAAGAGCTCAAATTTAAAATC
T-G-GCG-GTC--T-TGGCCGTCCGAGTTGTAACCTGGAGGAGCGTTTT-CCGTGTCG-
GACCGTGTACAAGTCTCCTGGAACGGAGCGTCATAGAGGGTGAGAATCCCG-
TCCATGACACGG-AC-GCCCGATGC-
TTTGTGATACGCTCCCGAAGAGTCGCGTTGTTTGGGAATGCAGCGCAAAATGGGTGGTAAATTC
CATCTAAAGCTAAATATTGGCGAGAGACCGATAGCGAACAAGTACCGTGAGGGAAAGATGAAA

AGCACTTTGGAAAGAGAGTTAAACAGTACGTGAAATTGTTGAAAGGGAAACGCTTGAAGTCAG
TCGCGTCCGGTGGAACTCAGCCTTGC----TTCGGCCTGGTGTACTTTCCTC-T---
GGACGGGTCAACATCAA-TTTC-AAC-
CGGTGGACAAGGGCGAGGGGAATGTAGCGTTGTTTCGGCGACGTG-TTATAGC-CCCTCGCC-
GTAT-GCACCGGCCGGGATTGAGGA-CCGCAGCACGCCC------TT-G-TGGCC-------
GGGGT----------------------------TC-GCCCACG-T-ACCGTGCTTAGGAT-
GTTGGCATAATGGCTTTAAGCGACCCGTCTTGAAACACGGACCAAGGAGTCTAACATGCCTGCG
AGTGTTCGGGTGGAAAACCCTTGCGCGTAACGAAAGTGAAA-GTTGGGAG-----CCTC-------
-----------CGCAA--GGGGGTGCACCGACGCCCGGCCCTGACG-----
TTCTCTGACGGTGCTGCGGTAGAGCAAGTATGTTGGG----
ACCCGAAAGATGGTGAACTATGCCTGAATAGGGCGAAGCCAGAGGAAACTCTG-
GTGGAGGCTCGTAGCGATTCTGACGTGCAAATCGATCGTCAAATTTGGG------------------
----

>Fomitiporella_subinermis_Dai_15131
ACGAGTTTTGA-----A-G--GCGAGAG-------------------------------TTTGTCGCTG--
GTGCG----------------GAAACG--CGCA-TGTG-CACGAC-----------------------------
---TTT-CGTG-----CTCAATCC--A--CTC------------------AA-CCCC--TGTGCA-
CCTTTGCG--AAG--------------------
-------------------------------------CTAGTAGC----------------T------TCTCTTTT-

---------------------------CTTGTTCTGTCTTGA------------------------------------
--------------------------------CGACTGTAT---------------------------------------
----
------------------------GTGAGTGCTT--
---GCCTTGT------------------------------

------------------------TGCGAGGT-------------TCACTTGGT-------CGGAGGGGT---
--------------------------------------GGAAGG-AAGGGGAGCGAACGC-TTTGA-----
--CTTTA-TTAC-------------AA---ACTCATTGTAATGTCTTGTCGAAT-G-----T-AAT-GC-
T-CC-TT----GTGGGCGAAAT------
GAAATACAACTTTCAACAACGGATCTCTTGGCTCTCGCATCGATGAAGAACGCAGCGAAATGCG
ATAAGTAATGTGAATTGCAGAATTCAGTGAATCATCGAATCTTTGAACGCACCTTGCGCCCCTTG
G-TATTCCGAGGGGCATGCCTGTTTGAGTGTCATGTTAATCTCAAACCTC--------AAG-
TCTTTCTTAAC----------TGAA--GGGCT-----CGG-TCGGTTTGGAC-TTGGAGG-T-T------
ACTGCTGGC-TTCA---------------------TTTTGTAGTCGGCTCCTCTGAAATGCATTAGC-
TGGACTTTGG----------------T-TC---GCATTTT-----------
ACGGTGTAATAGTAACAACAA---------CTTG-TTCGCC------GGG-TGCTTG-CCTAA-
GGAGTCCGC-TTC-----TAATCGCCCCG--------TTAGTAAAAGGGGC-AA--------GG-----
-AGTAAT--------
CCTTTCATGAACACTTTGACCTCATCCCCTAGTAACTGCGAGTGAAGCGGGAAGAGCTCAAATT
TAAAATCT-G-GCG-GTC--TCTGGCCGTCCGAGTTGTAACCTGAAGGAGCGTTTT-
CCGCGTCG-GACCGTGTACAAGTCTCCTGGAACGGAGCGTCATAGAGGGTGAGAATCCCG-
TCCATGACACGG-AC-GCCCGATGC-
TTTGTGATACGCTCCCGAAGAGTCGCGTTGTTTGGGAATGCAGCGCAAAATGGGTGGTAAATTC
CATCTAAAGCTAAATATTGGCGAGAGACCGATAGCGAACAAGTACCGTGAGGGAAAGATGAAA
AGCACTTTGGAAAGAGAGTTAAACAGTACGTGAAATTGTTGAAAGGGAAACGCTTGAAGTCAG
TCGCGTCTTGTGGAATTCAGCCTTGC----TTCGGCCTGGTGTACTTTCCGC-T---
AGATGGGTCAACATCAA-TTTT-GGC-
CGGTGGACAAGGGCGAGGGGAATGTAGCGTCGTTTCGACGACGTG-TTATAGC-CCCTTGTC-
GCAT-ACACCGGCCGGGATTGAGGA-CCGCAGCGCGCCC------TT-G-TGGCC-------
GGGGG----------------------TT---CG-CCCCACG-T-ACCGCGCTTAGGAT-
GTTGGCATAATGGCTTTAAGCGACCCGTCTTGAAACACGGACCAAGGAGTCTAACATGCTTGCG
AGTGTTCGGGTGGAAAACCCTTGCGCGTAATGAAAGTGAAA-GTTGGGAG-----CCTC-------
-----------CGCAA--GGGGGTGCACCGACGCCCGGCCCTGACG-----
TTCTCTGACGGTGCTGCGGTAGAGCAGGTATGTTGGG----
ACCCGAAAGATGGTGAACTATGCCTGAATAGGGCGAAGCCAGAGGAAACTCTG-
GTGGAGGCTCGTAGCGATTCTGACGTGCAAATCGATCGTCAAATTTGGG-----------------
----

>Fomitiporella_tenuissima_Dai_12365
ACGAGTCTGAA-----A----CCGGGAG------------------------------CCTGTTGCTG--
GTGCG---------------GAGACG--CACA-TGTG-CACGGC----------------------------
---TTT-CGTG-----CTCAATCC--A--CTC-----------------AA-CCCC--TGTGCA-
CCTTTGCG--AAG-------------------
------------------------------------CCAGTAGC----------------C------TTTTTTCT-
--------------------------CTCTTTATT---
-----------------------------CAGCTGTAT---------------------------------------
----

--------------------------GGGAGCTT---GCCTTGT-------------------------------
----
---------------------TGCGGGTT-------------CCACTTGGCTT----------------------
-------------------------------GGAGGGAG-GAGGGAGGCGAACGC-TTTGA------
TTTTTA-TTAC-------------AA---ACCCATTAT-ATGTCTTGTGGAAT-G-----C-AAT-GC-T-
CC-TT----GTGGGCGAAAT------
GAAATACAACTTTCAACAACGGATCTCTTGGCTCTCGCATCGATGAAGAACGCAGCGAAATGCG
ATAAGTAATGTGAATTGCAGAATTCAGTGAATCATCGAATCTTTGAACGCACCTTGCGCCCCTTG
G-TATTCCGAGGGGCATGCCTGTTTGAGTGTCATGTTAATCTCAAACCAC--------GAG-
TCTTTCTTAAT----------GGAG--GGACT-----CGT-CCGGTTTGGAC-TTGGAGGAT-T------
ACTGCTGGC-TCCC----------------------TTTGTAGTCGGCTCCTCTGAAATGCATTAGC-
TGGACTTTGG----------------T-TC---GCA-TTT-----------ACGGTGTAATAGTAATAAA-
----------CTTG-TTCGCCG-----GGG-TGCTTG-CCTAA-GGAGTCTGC-TTC-----
TAATCGCCCGT----------TATTTAAAGGGC-AA--------GG-TCC---TCGGTTGAC------
CTTCTTGAACACTTTGACCTCATCCCCTAGTAACTGCGAGTGAAGCGGGAAGAGCTCAAATTTA

AAATCT-G-GCG-GTC--TCTGGCCGTCCGAGTTGTAACCTGGAGGAGCGTTTT-CCGCGTCG-
GACCGTGTACAAGTCTCCTGGAACGGAGCGTCATAGAGGGTGAGAATCCCG-
TCCATGACACGG-AC-GCCCGATGC-
TTTGTGATACGCCCCCGAAGAGTCGCGTTGTTTGGGAATGCAGCGCAAAATGGGTGGTAAATTC
CATCTAAAGCTAAATATTGGCGAGAGACCGATAGCGAACAAGTACCGTGAGGGAAAGATGAAA
AGCACTTTGGAAAGAGAGTTAAACAGTACGTGAAATTGTTGAAAGGGAAACGCTTGAAGTCAG
TCGTGTCCCGTGGAACTCAGCCTTGC----TTCGGCTYGGTGTACTTTCTGC-G---
GGACGGGTCAACATCAA-TTTC-ACC-
CGGTGGACAAGGGTGAGGGGAATGTAGCGTTGTTTCGGCGACGTG-TTATAGC-CTCTTGTC-
GTAT-ACACCGGGCGGGATTGAGGA-CCGCAGCGCGCCC------TT-G-TGGCC-------
GGGGT---------------------------TC-GCCCACG-T-ACCGCGCTTAGGAT-
GTYGGCATAATGGCTTTAAGCGACCCGTCTTGAAACACGGACCAAGGAGTCTAACATGCTTGCG
AGTGTTCGGGTGGAAAACCCTTGCGCGTAATGAAAGTGAAA-GTTGGGAA-----CCTC-------
-----------CGCGA--GGGGGTGCACCGACGCCCGGCCCTGAAG-----
TTTACTGACGGTGCTGCGGTAGAGCAAGTATGTTGGG----
ACCCGAAAGATGGTGAACTATGCCTGAATAGGGCGAAGCCAGAGGAAACTCTG-
GTGGAGGCTCGTAGCGATTCTGACGTGCAAATCGATCGTCAAATTTGGG------------------
----

>Fomitiporia_alpina_Dai_15735

CCGAGTTGGAA----------CGTGGAG------------------------------GTTGATGCTG--
GTGCA-CATAT---TTATTGTATGTG--TACA-TGTG-CTCGCC----------------------------
---TTCACACT-----CTTCATCC--A--CTC-----------------AACCCCC--TGTGCA-
CTTTATCA--GAG-------------------
------------------------------------TTAGTAAT----------------------AGTATT----
---------------------------GTGG--TGGCCG---TTTGTTATCCATTGTTAGAA--GTGGG--
----

--------------------------GTAACTCTT--

----------------TCTAGCAGTAGTAATA---
---------ATAACAATCTTGGTTC-----------TACTGC-TACTACTATGAACAT-TTTGA------
CTTTTACTTAT-----------ACAA---ACACTTTGCTTGTTCTTGTGAATGTG-----T-AAT-GC-
T-CC-TT----GTGAGCGAAATAC---
AAATATACAACTTTCAACAACGGATCTCTTGGCTCTCGCATCGATGAAGAACGCAGCGAAATGC
GATAAGTAATGTGAATTGCAGAATTCAGTGAATCATCGAATCTTTGAACGCACCTTGCGCCCCTT
GG-TATTCCGAGGGGCATGCCTGTTTGAGTGTCATGTAATTCTCAATCCTC--------TTT-
TTCTTAA--------------TTGA-----------AGAA-GGGGCTTGGAC-TTGGAGG-T-T-
ATATACATGCTGGT-ACT-----------------------
GTTTGTATCGGCTCCTCTTAAATGCATTAGC-TGGACTGTAG----------------T-TC---
GCATTGT-----------TTGGTGTAGTAATAGTTTTC------TATCTATA-TTCACTA-----CAG-
TGCTTG-CTTAG-ACTGTCTGC-TTC-----CAATTGTCCGC---------CTGTGTGTTGGAC-----
-----AGG-TAC---TCTGT----------TACCTTAAATCATTTGACCCCT-
CCCCTAGTAACTGCGAGTGAAGCGGGAAGAGCTCAAATTTAAAATCT-G-GCA-GTCT-
TCTGGCTGTCCGAGTTGTAATCTGGAGAAGCGTTTT-CTGTGTTA-
GACCGTGTACAAGTCTCCTGGAACGGAGCCTCATAGAGGGTGAGAATCCCG-
TCCATGACACGG-AC-TACTAATAC-
TTTGTGATACGCTCTCGAAGAGTCGAGTTGTTTGGGAATGCAGCTCAAAATGGGTGGTAAATTC
CATCTAAAGCTAAATACAGGCGAGAGACCGATAGCGAACAAGTACCGTGAGGGAAAGATGAA
AAGCACTTTGGAAAGAGAGTTAAACAGTACGTGAAATTGTTGAAAGGGAAACGCTTGAAGTCA
GTCGCGTTGTGCAGAACTCAGCCTTGC----CTCGGCTTGGTGTACTTTCTGC-A---
CAACGGGTCAACATCAA-TTTT-GGC-
CCATGGAGAAAGGTGGGGGGAATGTAGCGCTGTTTCGGCAGCGTG-TTATAGC-CCTCTATT-
GTAT-ACATGGGCTGGGATTGAGGA-TCGCAGCACGCCC------TT-G-TGGCC-------
GGGGG----------------------TT---CG-CCCTACG-T-ACCGTGCTTAGGAT-
GTTGGCAGAATGGCTTTAAGCGACCCGTCTTGAAACACGGACCAAGGAGTCTAACATGCTTGCG
AGTGTTCGGGTGGAAAACCCTTGTGCGTAATGAAAGTGAAA-GTTGGGAA-----CCTC-------
-----------CGCAA--GGGGGTGCACCGACGCCCGGCCCTGACG-----
TTCTCTGACGGTGCTGCGGTAGAGCAAGTATGCTAGG----
ACCCGAAAGATGGTGAACTATGCCTGAATAGGGCGAAGCCAGAGGAAACTCTG-
GTGGAGGCTCGTAGCGATTCTGACGTGCAAATCGATCGTCAAATTTGGG------------------
----

GGCACTGGTGAGTTCGAGGCTGGTATCTCGAAGGATGGACAAACTCGCGAGCACGCCTTGCTC
GCCTTCACCCTTGGCGTGCGACAGCTAATTGTCGCTGTTAACAAAATGGACACAACCAAGGTTC
GCTTGATTATTCAA--ATAATAGTCCTCGCACGCTAGATT--TAACCTTTAAC--
GTTGACATTAGTGGTCCGAGGACCGATTCAATGAAATCGTCAAGGAAACTTCGAACTTCATCAA
GAAGGTCGGTTACAACCCGAAGGCAGTCGCGTTCGTCCCGATTTCTGGCTGGCACGGTGACAA
CATGTTGGAGGAGTCTAGCAAGTAAGATAGGTTG-TACTTTG--AGCGCTCTAATTCG-----
TGCCTAATTACATCTCTT-GATAGCATGCCATGGTACAAGGGCTGGACAAAGGAAACGAAGG--
----------CCGGTGTTGTC-
AAGGGCAAGACGCTTCTTGATGCCATTGACGCCATTGAACCGCCTGTTCGTCCTGAAAACAAGC
CTCTCCGTCTTCCCCTGCAGGATGTGATTTCTGTTGGATCAGTGTCGGGACCTGTCATTGACTTCT
TAGAGGAATGGGGACTGGAGCCTCTTGAAGAGAATACTCA---GTCTACGA------
ATCTCACCAAAGTTTTCGTTAACGGCGTGTGGCTCGGGGTGTGTCGCGACCCGCTTAACATGGT
GAATACACTCCGTACTCTTAGGCGAATGC---
AGGATTTCGACAGCGAAGTGTCTATTGTTCGTGACATCCGTGAAAGGGAGATACGCATATACTC
TGACGCTGGTCGGGTTCTTCGACCTCTCTTCATTGTGGAAGATCACAAGCTGAAACTCAAGCGT
CAGCATCTCCGCTGGTTGCAGGAGAAATGGAGGATC-------GAGGAGAGGCCGTACGTC----
--CCAAGGGAAGAAAGAAACAAGGAGGAAGTTTCGGAGGGCGAGACCGTTGCAGAC---
GAAGAAGATGGTAGGCCGATTATGAGAGACGAACGAAGAGTGAAGAGAAAGAAGAAGCCGTT
CACTTGGTCTTCGTTGATTCGTGACGGTGTGATTGAGCTGCTTGACGCGGAGGAAGAGGAGACG
GTCATGATTTGCATGACACCAGATGATCTACTTGAATCTCGACTACTGCGCAGAGGTCTGGACC
CACG---TGCAGAGGCTGATTTCAACCCGGCTGCTCGGTTGAAACCATTACCGATT------
GCGCATACATGGACACATTGTGAAATTCATCCTAGCATGATTCTAGGTATTTGTGCTAGTATTATT
CCTTTCCC
>Fomitiporia_gaoligongensis_Cui_16433
ACGAGTTGGAA----T------GCGGGG-------------------------------GTTGATGCTG--
GTGCAATATAT---TTAT--TGTATG--TACA-TGTG-CTCGCC----------------------------
---TTCACACT-----CTTCATCC--ACTCTC-----------------AA-CCCC--TGTGCA-
CTTTATCA---GAG-------------------
--------------------------------------TTAGTAAT-----------------------GGTATT----
---------------------------GTGGTATGGCCA---TTTGTTATTCATTGTTAGAAGTGTGGT--
----

--------------------------GTAATCCTT---

----------------TCTGGCAGTAGTAATA---
---------AT---AATCTTGGTT--------------CTGC-TGCTACTATGAACAC-TTTGA------

CTTTTACTTAT-----------ACAA---ACACTCTGCTTGTTCTTGTGAATGTG-----TAAAT-GC-
T-CT-CT----GTGAGCGAAATACC-
AATATATACAACTTTCAACAACGGATCTCTTGGCTCTCGCATCGATGAAGAACGCAGCGAAATG
CGATAAGTAATGTGAATTGCAGAATTCAGTGAATCATCGAATCTTTGAACGCACCTTGCGCGCC
TTGG-TATTCCGAGGGGCATGTCTGTTTGAGTGTCATGTAATTCTCAATCCTT--------TTT-
TTCTTAA--------------TTGA-----------AGAA-GGGGCTTGGAC-TTGGAGG-T---
GTACATATGCTGGT-ACT----------------------
GTCTGTATTAGCTCCTCTCAAATGCATTAGC-TGGACTGTAG----------------T-TT---
GCATTGT-----------TTGGTGTAGTAATAGTTTTC------TATCTATA-TTCACTA-----CAG-
TGCTTG-CCTAG-ACTGTCTGC-TTC-----CAATAGTCTGG---------CTATGTGTTGGAC--A--
------GG-TAC---TCTGA----------TACCTTAAACCATTTGACCTCT-
CCCCTAGTAACTGCGAGTGAAGCGGGAAGAGCTCAAATTTAAAATCT-G-GTA-GTCT-
TCTGGCTGTCCGAGTTGTAATCTGGAGAAGTGTTTT-CTGTGTTA-
GACCGTGTACAAGTCTCCTGGAACGGAGCGTCATAGAGGGTGAGAATCCCG-
TCCATGACACGG-AC-TACTAATAC-
TTTGTGATACGCTCTCAAAGAGTCGAGTTGTTTGGGAATGCAGCTCAAAATGGGTGGTAAATTCC
ATCTAAAGCTAAATACAGGCGAGAGACCGATAGCGAACAAGTACCGTGAGGGAAAGATGAAA
AGCACTTTGGAAAGAGAGTTAAACAGTACGTGAAATTGTTGAAAGGGAAACGCTTGAAGTCAG
TCGCGTTGTGCAGAACTCAGCCTTGC----CTTGGCTTGGTGTACTTTCTGC-A---
CAACGGGTCAACATCAA-TTTT-GGC-
CCATGGAGAAAGGTAGGAGGAATGTAGCGCTGTTTCGGCAGCGTG-TTATAGC-CCTCTATT-
GTAT-ACATGGGCTGGGATTGAGGA-TTGCAGCACGCCC------TT-G-TGGCC-------
GGGGG----------------------TTCG-CG-CCCTACG-T-ACCGTGCTTAGGAT-
GTTGGCAGAATGGCTTTAAGCGACCCGTCTTGAAACACGGACCAAGGAGTCTAACATGCTTGCG
AGTGTTCGGGTGGAAAACCCTTGTGCGTAATGAAAGTGAAA-GTTGGGAA-----CCTC-------
-----------CGCAA--GGGGGTGCACCGACGCCCGGCCCTGACG-----
TTCTCTGACGGTGCTGCGGTAAAGCAAGTATGCTAGG----
ACCCGAAAGATGGTGAACTATGCCTGAATAGGGCGAAGCCAGAGGAAACTCTG-
GTGGAGGCTCGTAGCGATTCTGACGTGCAAATCGATCGTCAAATTTGGG------------------
----

GGCACTGGTGAGTTCGAGGCTGGTATCTCGAAGGATGGACAAACTCGCGAGCACGCTCTGCTC
GCCTTCACCCTTGGCGTGCGTCAGCTAATTGTCGCTGTTAACAAAATGGACACAACCAAGGTTC
GCTTGGTTATTCAA--ATATTATTCCTCACATGCTAGATC--TGACCTTTAAC--
GTTGACATTAGTGGTCCGAGGACCGATTCAATGAAATCGTCAAAGAAACTTCCAACTTCATCAA
GAAGGTCGGTTACAACCCGAAGGCGGTCGCGTTCGTCCCGATTTCTGGCTGGCACGGTGACAA
CATGTTGGAGGAATCGACCAAGTAAGATA-GTTG-GACTTTG--AGCGCTCTAATTCG-----

TGCCTAATTACATCTCTT-AATAGCATGCCATGGTACAAGGGCTGGACAAAGGAAACGAAGG--
----------CCGGTGTTGTC-
AAGGGCAAGACGCTTCTTGATGCCATTGATGCCATTGAACCGCCTGTTCGTCCTGAAAACAAGC
CTCTCCGTCTTCCCCTGCAGGATGTGATTTCTGTTGGTTCTGTGTCGGGACCTGTCATTGACTTCT
TGGAGGAATGGGGACTGGAGACCCTTGAGGAGAATACCCA---GTCCGCGA------
ATCTTACGAAAGTCTTCGTTAACGGCGTGTGGCTCGGGGTGTGTCGTGACCCGCTTAACATGGT
GAATACACTCCGCACTCTCAGGCGAATGC---
AGGATTTCGACAGCGAAGTGTCTATTGTTCGCGATATCCGTGAAAGAGAGATACGTATATACTC
CGATGCCGGCCGTGTTCTTCGACCACTCTTCATTGTGGAAGATCTCAAGCTGAAACTCAAGCGT
CAGCATCTCCGCTGGCTGCAAGAGAAATGGAGGATT-------GAGGAACGGCCATATGTC----
--CCAAGGGAAGAAAGAAACAAGGAGGAGGTTTCGGAGGGTGAAACCGTCGCAGAC---
GAAGAAGACGGTAGGCCGATTATGAGGGACGAACGAAGGGTGAAGAGAAAGAAGAAGCCATT
CACTTGGTCTTCGTTGATTCGTGACGGTGTTATTGAGCTGCTGGACGCAGAAGAAGAGGAGACG
GTCATGATCTGCATGACACCAGATGATTTACTCGAATCTCGACTATTACGCAGAGGTCTAGACC
CACG---TGCAGAGGCTGATTTCAACCCAGCTGCCCGGTTGAAGCCATTACCGATT------
GCGCATACATGGACACATTGTGAAATTCACCCTAGCATGATTCTGGGTATCTGTGCTAGTATTAT
A--------
>Fomitiporia_punicata_Dai_10640
ACGAGTTGGAA----------CGTGGAG------------------------------GTTGATGCTG--
GTGCA-TATATTTATTATTGTATGTG--TACA-TGTG-CTCGCC----------------------------
---TTCACACT-----CTTCATCC--A--CTC-----------------AA-CCCC--TGTGCA-
CTTTATCA--GAG-------------------
------------------------------------TTAGTAAT----------------------AGTATTGT--
--------------------------AGTGG--CGGCCG---TTTGTTATTCATTGTTAGAA--GCGGG--
----

--------------------------GTAACTCTT--

----------------TCTAGCAGTAGTAATA---
---------ATAACAATCTTGGTTC----------TACTAC-TATTACTGTGAACAC-TTTGA------
CTTTTACTTAT----------ACAA---ACACTTTGCTTGTTCTTGTGAATGTG-----T-AAT-GC-
T-CC-TT----GTGAGTGAAATAC---
AAATATACAACTTTCAACAACGGATCTCTTGGCTCTCGCATCGATGAAGAACGCAGCGAAATGC
GATAAGTAATGTGAATTGCAGAATTCAGTGAATCATCGAATCTTTGAACGCACCTTGCGCCCCTT
GG-TATTCCGAGGGGCATGCCTGTTTGAGTGTCATGTAATTCTCAATCCTC--------TTT-
TTCTTAA--------------TTGA-----------AGAA-GGAGTTTGGAC-TTGGAGG-T-
TAATATACATGCTGGT-ACT-----------------------
GTCTGTATCGGCTCCTCTAAAATGCATTAGC-TGGACTGTAG----------------T-TC---
GCATTGT-----------TTGGTGTAGTAATAGTTTTC------TATCTATA-TTCACTA-----CAG-
TGCTTG-CTTAG-ACTGTCTGC-TTC-----TAATAGTCCGC---------TTATGTGTCGGAC------
----AGG-TAC---TCTGT----------TACCTTAAACCATTTGACTCCT-
CCCCTAGTAACTGCGAGTGAAGCGGGAAGAGCTCAAATTTAAAATCT-G-GCA-GTCT-
TCTGGCTGTCCGAGTTGTAATCTGGAGAAGCGTTTT-CTGTGTTA-

GACCGTGTACAAGTCTCCTGGAACGGAGTGTCATAGAGGGTGAGAATCCCG-
TCCATGACACGG-AC-TACTAATAC-
TTTGTGATACGCTCTCGAAGAGTCGAGTTGTTTGGGAATGCAGCTCAAAATGGGTGGTAAATTC
CATCTAAAGCTAAATACAGGCGAGAGACCGATAGCGAACAAGTACCGTGAGGGAAAGATGAA
AAGCACTTTGGAAAGAGAGTTAAACAGTACGTGAAATTGTTGAAAGGGAAACGCTTGAAGTCA
GTCGCGTTGTGCAGAACTCAGCCTTGC----CTCGGCTTGGTGTACTTTCTGC-A---
CAACGGGTCAACATCAA-TTTT-GGC-
CCATGGAGAAAGGTGGGGGGAATGTAGCGCTGTTTCGGCAGCGTG-TTATAGC-CCTCTATT-
GTAT-ACATGGGCTGGGATTGAGGA-TCGCAGCACGCCC------TT-G-TGGCC-------
GGGGG----------------------TT---CG-CCCTACG-T-ACCGTGCTTAGGAT-
GTTGGCAGAATGGCTTTAAGCGACCCGTCTTGAAACACGGACCAAGGAGTCTAACATGCTTGCG
AGTGTTCGGGTGGAAAACCCTTGTGCGTAATGAAAGTGAAA-GTTGGGAA-----CCTC-------
-----------CGCAA--GGGGGTGCACCGACGCCCGGCCCTGACG-----
TTCTCTGACGGTGCTGCGGTAGAGCAAGTATGCTAGG----
ACCCGAAAGATGGTGAACTATGCCTGAATAGGGCGAAGCCAGAGGAAACTCTG-
GTGGAGGCTCGTAGCGATTCTGACGTGCAAATCGATCGTCAAATTTGGG-----------------
----

GGCACTGGTGAGTTCGAGGCTGGTATCTCGAAGGATGGACAAACTCGCGAGCACGCTCTGCTC
GCCTTCACCCTTGGCGTGCGACAGCTAATTGTCGCTGTTAACAAAATGGACACAACCAAGGTTC
GCTTGATTATTCAA--ATAATATTCCTCGCACGTTAGATT--TAACCTTTAAC--
GTTGACATTAGTGGTCCGAGGACCGATTCAATGAAATCGTCAAGGAAACTTCGAACTTCATCAA
GAAGGTCGGTTACAACCCGAAGGCAGTCGCGTTTGTCCCGATTTCTGGCTGGCACGGTGACAA
CATGTTGGAGGAGTCTAGCAAGTAAGATAGGTTG-TACTTTG--AGCGCTCTAATTCG-----
TGCCTAATTACATCTCTT-GATAGCATGCCATGGTACAAGGGCTGGACAAAGGAAACGAAGG--
----------CCGGTGTTGTC-
AAGGGCAAGACGCTTCTTGATGCCATTGATGCCATTGAACCGCCTGTTCGTCCTGAAAACAAGC
CTCTCCGTCTTCCTCTGCAGGATGTGATTTCTGTTGGATCAGTGTCGGGACCTGTCATTGACTTCT
TAGAGGAATGGGGTTTGGAGCCCCTTGAGGAGAATACCCA---GTCCACGA------
ATCTTACAAAAGTTTTCGTTAACGGCGTGTGGCTCGGGGTGTGTCGCGACCCGCTTAACATGGT
GAATACACTCCGCACTCTCAGGCGAATGC---
AGGATTTCGATAGCGAAGTGTCCATTGTTCGTGACATCCGTGAAAGGGAGATACGCATATACTC
TGACGCTGGTCGTGTTCTTCGACCACTCTTCATTGTGGAAGATCACAAGCTGAAACTCAAGCGT
CAGCATCTCCGCTGGCTGCAAGAGAAATGGAGGATC-------GAGGAAAGGCCTTACGTC----
--CCAAGGGAAGAAAGAAACAAGGAGGAAGTTTCGGAGGGCGAGACCGTTGCAGAC---
GAAGAAGAGGGTAGGCCGATTATGAGGGACGAACGAAGAGTGAAGAGAAAGAAGAAGCCATT
CACTTGGTCTTCGTTGATTCGCGATGGTGTCATTGAGCTGCTTGACGCGGAGGAAGAGGAGACA

GTTATGATCTGCATGACACCAGATGACTTACTCGAGTCTCGACTTTTGCGTAGAGGTCTGGATCC
ACG---TGCAGAGGCCGACTTCAACCCGGCTGCTCGGTTAAAACCATTACCGATT------
GCGCATACATGGACGCATTGTGAAATTCATCCTAGCATGATTCTGGGTATTTGTGCTAGCATTAT
ACCTTTCCC
>Fomitiporia_subrobusta_Dai_13577
ACGAGTTGGAA----C-----GGTGGAG------------------------------GTTGACGCTG--
GTGCA-TATAT---TTATTGTATGTG--TACA-TGTG-CTCACC-----------------------------
---TTCACACT-----CTTCATCC--A--CTC-----------------AC-CCCC--TGTGCA-
CTTTATCA---GAG-------------------
-------------------------------------TTAGTAAT----------------------AGTATT----
---------------------------GTGG--TACCCA---CTTGTTATTCATTGTTAGAA--GCAGG--
----

--------------------------GTAACCCTT---

----------------TCTGACAGTACCAATA---
---------------ATCTTGGTTC-----------TACTAC-TACTACTATGAACGC-TTAGA-------
CTTTACTTAT-----------ACAA---ACACTTTGCTTGTTCTTGTGAATGTG-----T-AAT-GC-T-
CC-TT----GTGAGCGAAATAC---
AAATGTACAACTTTCAACAACGGATCTCTTGGCTCTCGCATCGATGAAGAACGCAGCGAAATGC
GATAAGTAATGTGAATTGCAGAATTCAGTGAATCATCGAATCTTTGAACGCATCTTGCGCCCCTT
GG-TATTCCGAGGGGCATGCCTGTTTGAGTGTTATGTAATTCTCAATCCTT--------TTT-
TTCTTAG--------------TTGA-----------AGAA-GGGGCTTGGAC-TTGGAGT---------
GTATGCTGGT-ACT-----------------------GTCTGTATTGGCTCCTCTTAAATGCATTAGC-
TGGACTGTAG----------------T-TT---GCATTTT-----------TTGGTGTAATAGT---TTTC-
-----TATCTATA-TTCACTA-----CAG-TGCTTA-CTTAG-ACTGTCTGC-TTC-----
CAATAGTCCGC---------CTATGTGTTGGAC-AA--------GG-TAT---TCTGT----------
TACCTTAAACCATTTGACACCT-
CCCCTAGTAACTGCGAGTGAAGCGGGAAGAGCTCAAATTTAAAATCT-G-GCA-GTCT-
TCTGGCTGTTCGAGTTGTAATCTGGAGAAGTGTTTT-CTGTGTTA-
GACCGTGTACAAGTCTCCTGGAACGGAGCGTCATAGAGGGTGAGAATCCCG-
TCCATGGCACGG-AC-TACTAATAC-
TTTGTGATACACTCTCGAAGAGTCGAGTTGTTTGGGAATGCAGCTCAAAATGGGTGGTAAATTCC
ATCTAAAGCTAAATACAGGCGAGAGACCGATAGCGAACAAGTACCGTGAGGGAAAGATGAAA
AGCACTTTGGAAAGAGAGTTAAACAGTACGTGAAATTGTTGAAAGGGAAACGCTTGAAGTCAG
TCGCGTTGTGCAGAACTCAGCCTTGT----CTCGGCTTGGTGTACTTTCTGT-A---
CAACGGGTCAACATCAA-TTTT-GGC-
CCATGGAGAAAGGTGGGGGGAATGTAGCGCTGTTTCGGCAGCGTG-TTATAGC-CCTCTACT-
GTAT-ACATGGGCTAGGATTGAGGA-ATGCAGCACGCCC------TT-G-TGGCC-------
GGAGG----------------------TT---CG-CCTCACG-T-ACCGTGCTTAGGAT-
GTTGGCAGAATGGCTTTAAGCGACCCGTCTTGAAACACGGACCAAGGAGTCTAACATGCTTGCG
AGTGTTTGGGTGGAAAACCCTTGTGCATAATGAAAGTGAAA-GTTGGGAA-----CCTC-------
-----------CGCAA--GGGGGTGCACCGACGCCCGGCCCTGACG-----

TTCTCTGACGGTGCTGCGGTAGAGCAAGTATGCTAGG----
ACCCGAAAGATGGTGAACTATGCCTGAATAGGGCGAAGCCAGAGGAAACTCTG-
GTGGAGGCTCGTAGCGATTCTGACGTGCAAATCGATCGTCAAATTTGGG------------------
----

GGCACTGGTGAGTTCGAGGCTGGTATCTCAAAGGATGGACAAACCCGCGAGCACGCTCTGCTC
GCCTTCACCCTTGGCGTGCGTCAGCTAATTGTCGCTGTTAACAAAATGGACACAACCAAGGTTC
GCTCGATTATTCAA--ATATTATTCCTCGCACGCTAGATT--TAACCTTTAAC--
ATTGACATTAGTGGTCCGAGGACCGATTCAATGAAATCGTCAAGGAAACTTCCAACTTTATCAA
GAAGGTCGGTTACAACCCGAAGGCAGTCGCGTTCGTCCCGATTTCTGGCTGGCACGGTGACAA
CATGTTGGAGGAGTCTATCAAGTAAGATAGGTTG-TACTTTG--AGCGCTATAATTCG-----
TGCCTAATTACATCTCTT-GATAGCATGCCATGGTACAAGGGCTGGACAAAGGAAACGAAGG--
----------CCGGCGTTGTC-
AAGGGCAAGACACTTCTTGATGCCATTGATGCCATTGAACCGCCTGTTCGTCCTGAAAACAAGC
CTCTCCGTCTTCCCCTGCAGGATGTAATCTCCGTCGGTTCAGTGTCGGGACCTGTTATTGACTTCT
TAGAAGAATGGGGTTTGGAGCCTCTTGAGGAGAATGCCCA---GTCCACGA------
ATCTTACCAAAGTCTTCGTCAACGGCGTGTGGCTCGGGGTGTGCCGCGACCCGCTCAACATGGT
GAATACACTCCGTACTCTCAGGCGAATGC---
AGGATTTCGATAGTGAAGTGTCTATCGTTCGTGACATCCGCGAGAGGGAGATACGTATATACTC
TGATGCCGGCCGAGTTCTTCGACCACTCTTCATTGTGGAGGATCACAAGCTGAAACTTAAGCGT
CAGCATCTCCGCTGGCTGCAAGAGAAATGGAGGATC-------GAGGAAAGGCCATACGTT----
--CCAAGGGAAGAAAGAAACAAGGAGGAAGTTTCGGAGGGCGAGACCGTCGCAGAC---
GAAGAAGACGGTAGGCCGATTATGAGAGACGAACGAAGGATGAAGAGGAAGAAGAAGCCGTT
CACCTGGTCTTCGTTGATTCGCGACGGTGTCATTGAGCTGCTGGACGCAGAGGAAGAGGAGAC
CGTCATGATCTGCATGACACCAGATGATTTACTTGAATCTCGACTATTGCGCAGAGGTCTGGAC
CCACG---CGCAGAGGCCGACTTCAACCCGGCTACCCGGTTGAAGCCATTACCGATT------
GCGCATACATGGACACATTGTGAAATTCATCCTAGCATGATTCTGGGTATTTGTGCTAGTATTAT
ACCTTTCCC
>Fulvifomes_dracaenicola_Dai_22097
ATGAGTTTGAA----------CCGGGAG-------------------------------TCTGTTGCTG--
GTGCG---------------GAAACG--CACATTGTG-CACGGC-----------------------------
---TTC-TGTG-----CTCAATCC--A-TTTC-----------------AA-CCCC--TGTGCA-
CCTAATCG---AAG-------------------
-------------------------------------CGAGTAGT----------------C------
TCTTTTTATT--
TGTTGGTTGTATGTGGAAGGGGGAA---

---CGGGTTACT---------
-----------------ATAAGT-ATGTCAGTAA---------------------------------
GGCGAACG-CACCTTTTTCG--

GCGTTACTA---------------GCTTGAA-------GGCGAAAGTAGTAGTAGT--------
AGTCCTTTTGTACACTTGGCC-GGCAAGGA-AAGAGAGTCGAAAGC-TTTGA------CCTC-----
TT-------------AA---ACACTTTAT-ATGTCTTGTGGAAT-G-----T-ATTTGC-T-CC-TC----
GTGGGCGAAAT-------
AAATACAACTTTCAACAACGGATCTCTTGGCTCTCGCATCGATGAAGAACGCAGCGAAATGCGA
TAAGTAATGTGAATTGCAGAATTCAGTGAATCATCGAATCTTTGAACGCACCTTGCGCCCCTTGG
-TATTCCGAGGGGCATGCCTGTTTGAGTGTCATGTTAATCTCAAACCCC--------TTG-
TCTTTAT--------------------TGACTC----GGT-TTGGTTTGGAC-TTGGAGG-T--------
GTTGCTGGC----------------------------GCTGCGTCGGCTCCTCTTAAATGTATTAGC-
TGGACTTTGG----------------T-TC---GCA-TTA-----------ATGGTGTAATAGTAA------
---------AGTG-TTCGCC------AGG-TGCTTG-CCTAA-TGAGTCTGC-TTC-----
TAATCGTCTT------------------CGGAC-AA--------GG-TCG---ACTGC-GAC----
CTTTTCTCTTGACCTTTGACCTCA-
CCCCTAGTAACTGCGAGTGAAGCGGGAAGAGCTCAAATTTAAAATCT-G-GCG-GCTT-
TTTGGTCGTCCGAGTTGTAACCTGGAGAGGCATTTT-CCGCGTTG-
GACCGTGTACAAGTCTCCTGGAATGGAGCGTCATAGAGGGTGAGAATCCCG-
TCCATGACACGG-AC-CGCCAATGC-
TCTGTGATATGCCTTCGAAGAGTCGCGTTGTTTGGGAATGCAGCGCAAAATGGGTGGTAAATTC
CATCTAAAGCTAAATATTGGCGAGAGACCGATAGCGAACAAGTACCGTGAGGGAAAGATGAAA
AGCACTTTGGAAAGAGAGTTAAACAGTACGTGAAATTGTTGAAAGGGAAACGCTTGAAGTCAG
TCGCGTTCCGTGGAACTCAGCCTTAC----TTCGGTTTGGTGTACTTTCCAT-GT--
GAACGGGTCAACGTCAA-TTTT-GGC-
CGGTGGATAAAGGCAAAGGGAATGTAGCGTTGCTTAGGTGACGTG-TTATAGC-CCCTTGTT-
GTAT-ACACCGGCTGGGATTGAGGA-CCGCAGCACGCCC------TT-G-TGGCC-------
GGGGG----------------------TT---CG-CCCCACG-T-ACCGTGCTTAGGAT-
GTTGGCATAATGGCTTTAAGCGACCCGTCTTGAAACACGGACCAAGGAGTCTAACATGCTTGCA
AGTGTTCGGGTGGAAAACCCTTGCGCGTAATGAAAGTGAAA-GTTAGGAA-----CCTTCTC----
----------GCAAGA--GGGGGTGCACTGACGCCCGGCCCTGACG-----
TTCTCTGACGGTGCTGCGGTAGAGCAAGTATGTTGGG----
ACCCGAAAGATGGTGAACTATGCCTGAATAGGGCGAAGCCAGAGGAAACTCTG-
GTGGAGGCTCGTAGCGATTCTGACGTGCAAATCGATCGTCAAATTTGGG------------------
----

>Fulvifomes_fastuosus_Dai_18292
ATGAGTTTGAA----------TCGGGAG-------------------------------CCTGTTGCTG--
GTGCG---------------GAAACG--CATATCGTG-CACGGC-----------------------------
---TTT-TGTG-----CTCAATCC--A-TTTC-----------------AA-CCCC--TGTGCA-
CCTTATCG--AAG--------------------
-------------------------------------TGAGTAGT----------------C------
TCTTTTTATT--
TGTTGGCTGTATGCGGAAGGGGGA--

---CGAATTACT---------
-----------------ATGAGT-ATGTTAGTAA----------------------------------
GGCGAACG-CACCTTTTTAG--

ATGTTACTA---------------GCTTGAA-------GGCGAAAGTAGTAGTAGT--------
AGTTCTCTTGTACTCTTGGCC-GGCAAGG--AAGAGAGTCGAAAGC-TTTGA------CTTCT----
TT-------------AA---ACACTTTAT-ATGTCTTGTGGAAT-G-----T-ATTAGC-T-CC-TT----
GTGGGCGAAAT-------
AAATACAACTTTCAACAACGGATCTCTTGGCTCTCGCATCGATGAAGAACGCAGCGAAATGCGA
TAAGTAATGTGAATTGCAGAATTCAGTGAATCATCGAATCTTTGAACGCACCTTGCGCCCTTTGG
-TATTCCGAAGGGCATGCCTGTTTGAGTGTCATGTTAATCTCAAACCCC--------TTG-
TCTTTGT--------------------TGACTC----GGT-TTGGTTTGGAC-TTGGAGG-T--------
GTTGCTGGC-ACATGG-------------GTTCT--TGCCTTGTCGGCTCCTCTTAAATGTATTAGC-
TGGGCTTTGG----------------T-TC---GCA-TTA-----------ACGGTGTAATAGTAA------
---------AGTG-TTCGCC------GGG-TGCTTG-CCTAA-CGAGTCTGC-TTC-----
TAATTGTCTT------------------CGGAC-AA--------GG-TCG---ACTCT-GAC----
CTTTTCTCTTGACCTTTGACCTCATCCCCTAGTAACTGCGAGTGAAGCGGGAAGAGCTCAAATTT
AAAATCT-G-GCG-GCT--TCTAGTCGTCCGAGTTGTAACCTGGAGAGGCATTTT-CCGCGTTG-

GACCGTGTACAAGTCTCCTGGAATGGAGCGTCATAGAGGGTGAGAATCCCG-
TCCATGACACGG-AC-CGCCAATGC-
TCTGTGATATGCCTTCAAAGAGTCGCGTTGTTTGGGAATGCAGCGCAAAATGGGTGGTAAATTC
CATCTAAAGCTAAATATTGGCGAGAGACCGATAGCGAACAAGTACCGTGAGGGAAAGATGAAA
AGCACTTTGGAAAGAGAGTTAAACAGTACGTGAAATTGTTGAAAGGGAAACGCTTGAAGTCAG
TCGCGTTCCGTGGAACTCAGCCTTAC----TTCGGTTTGGTGTACTTTCCAT-GT--
GAACGGGTCAACGTCAA-TTTT-GAC-
TGGTGGACAAAGACAAAGGGAATGTAGCGTTGCTTCGGTGACGTG-TTATAGC-CCCTTGTT-
GTAT-ACACCGGCTGGGATTGAGGA-CTGCAGCACGCCC------TT-G-TGGCC-------
GAGGGG---------------------TT---CGCCCCTACG-T-ACCGTGCTTAGGAT-
GTTGGCATAATGGCTTTAAGCGACCCGTCTTGAAACACGGACCAAGGAGTCTAACATGCTTGCG
AGTGTTCGGGTGGAAAACCCTTGCGCGTAATGAAAGTGAAA-GTTAGGAA-----CCTTCTC----
----------GCAAGA--GAGGGTGCACTGACGCCCGGCCCTGACG-----
TTCTCTGACGGTGCTGCGGTAGAGCAAGTATGTTGGG----
ACCCGAAAGATGGTGAACTATGCCTGAATAGGGCGAAGCCAGAGGAAACTCTG-
GTGGAGGCTCGTAGCGATTCTGACGTGCAAATCGATCGTCAAATTTGGG------------------
----

>Fulvifomes_hainanensis_Dai_11573
ACGAGTTTGAA----------TCGAGAG------------------------------CCTGTTGCTG--

GTGCG---------------GAAACG--CACATCGTG-CACGGC-----------------------------
---TCC-CGTG-----CTCAATCC--A-TTTC-----------------AA-CCCCCTTGTGCA-
CCTTATCG---AAG-------------------
-------------------------------------TCAGTAGT----------------CT----
TTCTCTTTATT--
TGCTGGTTGTGGCCGGCGAGGAGA---

GAGAGGGTCGAAAGC-TTTGA------CCTTTC-TATT-------------AA---ACGCTTTGT-
ATGTCTTGTGGAAC-G-----T-ATCAGC-T-CC-TTTGTGGGGGGCGAAAT-------
AAATACAACTTTCAACAACGGATCTCTTGGCTCTCGCATCGATGAAGAACGCAGCGAAATGCGA
TAAGTAATGTGAATTGCAGAATTCAGTGAATCATCGAATCTTTGAACGCACCTTGCGCCCCTTGG
-TATTCCGAGGGGCATGCCTGTTTGAGTGTCATGTTAATCTCAAACCCC--------TTG-
TCTTTTTTGT------------------TGACT----TGG-YCGGTTTGGAC-TTGGAGG-T--------
GTTGCTGGC-GTGTAGTA-----------
CRCACACTGCTGCGTCGGCTCCTCTTAATTGCATTAGC-TGGACTTTGG----------------T-
TC---GCATTTG-----------ACGGTGTAATAGTAA---------------AGTG-TTCGC----
CGGGCG-TGCTTG-CCTAA-CGGGTCCGC-TTC-----TAATCGTCTT------------------
CGGAC-AA--------CT-GTG------------------
TTCGTTTTGACCTTTGACCTCATCCCCTAGTAACTGCGAGTGAAGCGGGAAGAGCTCAAATTTAA
AATCT-G-GCG-GCT--TTCAGTCGTCCGAGTTGTAATCTGGAGAGGCATTTT-CCGCGTTG-
GACCGTGTACAAGTCTCCTGGAACGGAGCGTCATAGAGGGTGAGAATCCCG-
TCCATGACACGG-AC-CGCCAATGC-
TCTGTGATATGCCTTCGAAGAGTCGCGTTGTTTGGGAATGCAGCGCAAAATGGGTGGTAAATTC
CATCTAAAGCTAAATATGGGCGAGAGACCGATAGCGAACAAGTACCGTGAGGGAAAGATGAA
AAGCACTTTGAAAAGAGAGTTAAACAGTACGTGAAATTGTTGAAAGGGAAACGCTTGAAGTCA
GTCGCGTTCCGTGGAACTCAGCCTTAC----TTCGGTTTGGTGTACTTTCCAT-GT--
GAACGGGTCAACGTCAA-TTTT-GGC-
CGGTGGACAAAGGCAAGGGGAACGTAGCGTCGCTTCGGTGACGTG-TTATAGC-CCCTCGTT-
GTAT-ACACCGGCTGGGATTGAGGA-CCGCAGCACGCCCTTAG--TT-G-TGGCC-------
GGGAGG---------------------TT---CGCCTCTACGTT-ACCGTGCTTAGGAT-
GTTGGCATAATGGCTTTAAGCGACCCGTCTTGAAACACGGACCAAGGAGTCTAACATGCTTGCG
AGTGTTCGGGTGGAAAACCCTTGCGCGTAATGAAAGTGAAA-GGTTAGGAGCCCTTCTC------
------------TSTAG--AGGGGTGCACTGACGCCCGGCCCTGAAG-----
TTCTCTGACGGTGCTGCGGTAGAGCAAGCACGTTGGG----
ACCCGAAAGATGGTGAACTATGCCTGAATAGGGCGAAGCCAGAGGAAACTCTG-
GTGGAGGCTCGTAGCGATTCTGACGTGCAAATCGATCGTCAAATTTGGG------------------
----

>Fulvifomes_lloydii_Dai_9642
ATGAGTTTGAA----------TCGGGAG-------------------------------TCTGTTGCTG--
GTGCG----------------GAAACG--TACATTGTG-CACGGC-----------------------------
---TTC-TGTG-----CTCAATCC--A-TTTC------------------AA-CCCC--TGTGCA-
CCTCATCA--AAG--------------------
-------------------------------------TCAGTAGT----------------C------
TCTTTTTATT---
TGTTGGCTGTATGCGGAAGGGGGACGAATTACTATAAGTATGTTAGTAAAGCGAACACACCTTT
TTCGGTGTTACTAGCTTGAAGGCGAAAGTAGTAGTAGTAGTCCTTTTGTACTCTTGGCCGGCAAG
GAAAGGGTCGAAAGCTTTGA------------
CTCGGAGTGAGTAGTCTCTTTTTGTTTGTTGGCTGTATGCGGAAGGGGGACGAATTACT-------
-------------------ATAAGT-ATGTTAGTAA----------------------------------
AGCGAACG-CACCTTTTTCG--

GTGTTACTA----------------GCTTGAA-------GGCGAAAGTAGTAGTAGT--------
AGTCCCTTTGTACTCTTGGCC-GGCAAGGG-AAGAGGGTTGAAAGC-TTTGA------CTTC-----
TT-------------AA---ACACTTTGT-ATGTCTTGTGGAAT-G-----T-ATTAGC-T-CC-TT----
GTGGGCGAAAT-------
AAATACAACTTTCAACAACGGATCTCTTGGCTCTCGCATCGATGAAGAACGCAGCGAAATGCGA
TAAGTAATGTGAATTGCAGAATTCAGTGAATCATCGAATCTTTGAACGCACCTTGCGCCCTTTGG
-TATTCCGAAGGGCATGCCTGTTTGAGTGTCATGTTAATCTCAAACCCC--------TTG-

TCTTTAT--------------------TGACTT----GGT-TTGGTTTGGAC-TTGGAGG-T--------
GTTGCTGGC-ACTTAGGT-----------TTCTT--GCCTATGTCGGCTCCTCTTAAATGTATTAGC-
TGGACTTTGG----------------T-TC---GCA-TTA-----------ATGGTGTAATAGTAA------
---------AGTG-TTCGCC------AGG-TGCTTG-CCTAA-CGAGTCTGCTTTC-----
TAATTGTCTT------------------CGGAC-AA---------G-TCG---ACTCT-GAC----
CTTCTCTCTTGA-
CTTTGACTCAATCCCCTAGTAACTGCGAGTGAAGCGGGAAGAGCTCAAATTTAAAATCT-G-
GCG-GCT--TTTAGTCGTCCGAGTTGTAACCTGGAGAGGCATTTT-CCGCGTTG-
GACCGTGTACAAGTCTCCTGGAATGGAGCGTCATAGAGGGTGAGAATCCCG-
TCCATGACACGG-AC-TACCAATGC-
TTTGTGATATGCTTTCGAAGAGTCGCGTTGTTTGGGAATGCAGCGCAAAATGGGTGGTAAATTCC
ATCTAAAGCTAAATATTGGCGAGAGACCGATAGCGAACAAGTACCGTGAGGGAAAGATGAAAA
GCACTTTGAAAAGAGAGTTAAACAGTACGTGAAATTGTTGAAAGGGAAACGCTTGAAGTCAGTC
GCGTTCCGTGGAACTCAGCCTTAC----TTCGGTTTGGTGTACTTTCCAT-GT--
GAACGGGTCAACATCAA-TTTT-GGC-
CGGTGGACAAAGGCAAGGGGAATGTAGCGTTGCTTCGGTGACGTG-TTATAGC-CCCTTGTT-
GTAT-ACACCGGCTGGGATTGAGGA-TCGCAGCACGCCC------TT-G-TGCCC-------
GGGGG----------------------TT---CGCCCCCACG-T-ACCGTGCTTAGGAT-
GTTGGCATAATGGCTTTAAGCGACCCGTCTTGAAACACGGACCAAGGAGTCTAACATGCTTGCG
AGTGTTCGGGTGGAAAACCCTTGTGCGTAATGAAAGTGAAA-GTTAGGAA-----CCTTTTC----
----------GCAAGA--GAGGGTGCACTGACGCCCGGCCCTGACG-----
TTCTCTGACGGTGCTGCGGTAGAGCAAGTATGTTGGG----
ACCCGAAAGATGGTGAACTATGCCTGAATAGGGCGAAGCCAGAGGAAACTCTG-
GTGGAGGCTCGTAGCGATTCTGACGTGCAAATCGATCGTCAAATTTGGG------------------
----

>Fuscoporia_chinensis_Dai_15713
----------------------AGCGGA----------------------------CGCTTGAGCTG--
GCAC------------------------TGCA-GGTG-CTCAGCG-----------------------------
TCTC--------CATTCATTCC--A-ATTC----------------AAA-CACC--TGTGCA-
CCTCGAGAACACGT--GAG-----------------
-------------------------TTGGGTTATTGGATCTGTGGT-----------------------------
----

-------------------TCAGTT---
---ATTCCCTTATTTGGGTTGAA---CTTAGT-GAAAGTGGCCCGCTC--------TCGTTTCTTA-
TTAC-------------AA---ACTCGTTATATTGTTTTGTAGAAT-G-----T-ATC-GC-C-TT-GAA-
--TTAGGCAAAGTCT-----
AAATACAACTTTCAACAACGGATCTCTTGGCTCTCGCATCGATGAAGAACGCAGCGAAATGCGA
TAAGTAATGTGAATTGCAGAATTCAGTGAATCATCGAATCTTTGAACGCACCTTGCGCTCCTTGG
-TATTCCGAGGAGCATGCCTGTTTGAGTGTCATTATGTACTCAACTCCC--------TTG-
ATTTTTTATAAA----------------------TCAT-TCGAGTTGGAT-TTGGAGG-T-TC---
TTTATGCTGGT-CC------------------------TCACGGATCGGCTCCTCTTAAATGCATTAGC-
TGAGCTTTCG----------------C-TT---GAT-GCA-----------ATGGTGTAATAATTATC----
--------------TTCAC----CGATGT-AACTAG-TGATA-TAGGCTTGC-TTC-----
TAATCGTCTCCTCTCCGAGACATTATAAAAGAT-AAA----------CCC--TTTCGG-
GGGGGAAT-CTTTTTGACTCTTCTGACCT---
CCCCTAGTAACTGCGAGTGAAGCGGGAAAAGCTCAAATTTAAAATCT-G-GCG-GTTC----
TGCCGTCCGAGTTGTAATCTGGAGAAGCGTTTT-CTGTGCTG-
GACCGTGTACAAGTCTCTTGGAACAGAGCGTCATAGAGGGTGAGAATCCCG-
TCCATGACACGG-AC-GCCCAGTGC-
TATGTGATACGTTTTCAAAGAGTCGAGTTGTTTGGGAATGCAGCTCAAAATGGGTGGTAAGCTC
CATCTAAAGCTAAATATTGGCGAGAGACCGATAGCGAACAAGTACCGTGAGGGAAAGATGAAA
AGCACTTTGGAAAGAGAGTTAAACAGTACGTGAAATTGTTGAAAGGGAAACGCTTGAAGTTAGT
CGCGTCTTACTTGACTCAGCCTTGC----CTCGGCTTGGTGTATTTCATGT-G---
AGACGGGTCAACATCGA-TTTT-GAC-CGGCAGAGAAGGGGGGCGAGAATGTGGCAT--
TCTTCGGAGTGTG-TTATAGC-TTACTCTC-ATAT-GTGTCGGTTGGGATCGAGGC-
CCGCAGCGCGCCC------TT-G-TGGCC-------GGAGG---------------------TT---CG-
CCTCACG-T-TACGCGCTTAGGAT-
GTTGGCATAATGGCTTTAAGCGACCCGTCTTGAAACACGGACCAAGGAGTCTAACATGCTCGCG

AGTGTTAGGGTGGAAAACCCTTGCGCAGAATGAAAGTAAAA-GTTGGGAA-----CCTC-------
-----------CGCAA--GGGGGCGCACCGACGCCCGGCCCAGAAG-----
TTTACGGACGGTGCTGCGGTAGAGCGCGTATGTTGGG----
ACCCGAAAGATGGTGAACTATGCCTGAATAGGGCGAAGCCAGGGGAAACCCTGTGTGGAGGC
TCGTAGCGATTCTGACGTGCAAATCGATCGTCAAATTTGGG------------------------------
----

GGTACTGGTGAGTTCGAGGCTGGTATCTCCAAGGATGGCCAGACTCGCGAGCACGCTCTGCTT
GCATTCACGCTCGGTGTGCGTCAGCTCATCGTTGCGGTCAACAAAATGGACACTACCAAGGTTC
GTGGCTTACTTGTAC-TTGAAAAATCATGTCAATGAACTA--------------------
TTATCAGTGGTCTGAGGATCGTTTCAACGAAATCGTCAAGGAGACGTCCAACTTTATCAAGAAG
GTTGGTTACAACCCGAAGGCCGTCTCTTTCGTCCCGATTTCTGGCTGGCACGGTGACAACATGC
TTGAGGAGTCCGCCAAGTACGAATTTTCA-AATATCC---------AGACAGA-----
CCCTCTTTCTCACCTATT-TATAGCATGCCGTGGTACAAGGGCTGGACTAAGGAGACCAAGG---
---------GCGGTGTCGTC-
AAAGGAAAGACTCTCCTTGACGCCATTGACGCCATTGAGCCCCCTGTCCGTCCCCAGGACAAG
CCTCTCCGTCTCCCCCTCCAGGATGTG-----------------------------------
GAGTTCTTGCAGGAATGGGGTCTGGATGGCCTAGAAGAGAATGCTCA---ATCGACTA------
ATTTGACGAAAGTATTCGTCAATGGTGTATGGTTGGGTGTCCATCGGGATCCATCCGGACTTGTT
CGAACTCTCAGAATGTTGAGACGAAGAG---
ACGATATCAGTACGGAGGTTTCCATTGTACGAGACATTCGAGAAAGGGAACTGCGCATAAACA
CCGATGCTGGCCGTGTTTGCAGGCCTCTTTTTATTGTTGAAGATCACAAGCTCAAGCTCAAGCGC
CAGCATTTACGGTGGATCCAAAAAAAATACAGAGTC-------GAAGAAAGACCGTATTCA-----
-------------GAAGAAAGAGATGTGATGTCGGAAGGAGAAACAGAAGATGAA---
GATGCGGAGGGT---------------
GTTGAGCCCAGAATGAAGAGAAAGAAGAAGCCGTATAGGTGGGGTTCTCTGGTGAAGGATGG
GATTATTGAGTATTTGGACGCCGAGGAAGAGGAGACGGTCATGATCTGTATGACCCCTGACGA
CTTGGTCGAGTCGCGCTTGATGAGGCAAGGCATCGATCCACG---
TCAGGAGGGAGATTTCGATCCCGCAGCCCGTTTGAAATCGGTTCCTATG------
GCCCACACATGGACGCATTGCGAGATCCATCCAAGTATGATACTTGGCATATGCGCTAGTATCA
TTCCTTTCCC
>Fuscoporia_contigua_Dai_16025
-----------------G----TAGTGGGT------------------------------CGCTTGAGCTG--
GCAT-------------------------TGCA-GGTG-CTCGGCG------------------------------
ACTT-CACT----CTCTCATCC--A--TTA------------------AA-CCCC-TTGTGCA-
CTATGAAGCGAGGTT---GAA------------------
------------------------------------TCGAGTTGTAAT---------------GACT------------

----

---------------------------------CTGAGGAGACTAAGC-----------TTTCTTT-ATAT----
---------AA---ACTCATTTA-TTGTCTTGGAGAAT-G-----T-GATAGCCT-CT-TT----
GTGGGCGAAAAAG----
TTAATACAACTTTCAACAACGGATCTCTTGGCTCTCGCATCGATGAAGAACGCAGCGAAATGCG
ATAAGTAATGTGAATTGCAGAATTCAGTGAATCATCGAATCTTTGAACGCACCTTGCGCTCCTTG
G-TATTCCGAGGAGCATGCCTGTTTGAGTGTCATGATATTCTCAATCTTA--------TTG-
ATTTCTTGTTAA----------------------------TAAGATTGGAG-TTGGAGG----------
TCTGCTGGT-TCG-----------------------TCTTGAATCGGCTCCTCTTGAATGCATTAGC-
TGGGCTTATG----------------C-TC---GAT-GCA-----------ACAGTGTAATAATTGT----
---------------TTTACCAC-TGGTGT-GACTTG-CATGA-TGGGTCCGC-TTC-----
TAATCGTCTCTGTC--------------GAGAC-ATT----ATGGG------------
AACACTTCGTTCTCTGATCTCTTTGACCT---
CCCCTAGTAACTGCGAGTGAAGCGGGAAAAGCTCAAATTTAAAATCT-G-GCG-GCTT----
TGCTGCCCGAGTTGTAATCTGGAGAAGCGTTTT-CCGTGCTG-
GACCGTGTACAAGTCTCTTGGAACAGAGCGTCATAGAGGGTGAGAATCCCG-
TCCATGACACGG-AC-TCCCAGTGC-
TTTGTGATACGCTCTCGAAGAGTCGAGTTGTTTGGGAATGCAGCTCAAAATGGGTGGTAAATTC
CATCTAAAGCTAAATATTGGCGAGAGACCGATAGCGAACAAGTACCGTGAGGGAAAGATGAAA
AGCACTTTGGAAAGAGAGTTAAACAGTACGTGAAATTGTTGAAAGGGAAACGCTTGAAGTCAG
TCGCGTCGTCCACAACTCAGCCTTGC----CTCGGCTTGGTGCATTTTGTGC-A---
TGACGGGTCAACATCGA-TTTG-GAC-CGACGGACAAAGCGTGAGGGAATGTGGCAC--
TCCTCGGAGTGTG-TTATAGC-CCTTACGC-ATAT-ACGCCGGTCGGGATCGAGGA-
CCGCAGCGCGCCC------TT-G-TGGCC-------GGAGG----------------------TT---CG-
CCTCACG-T-TTCGCGCTTAGGAT-
GTTGGCAAAATGGCTTTAAGCGACCCGTCTTGAAACACGGACCAAGGAGTCTAACATGCTCGC
GAGTGTTAGGGTGGAAAACCCTTGCGCATAATGAAAGTAAAA-GTTGAGAG-----CCTC------
------------CGCAA--GGGGGTGCATCGACGCCCGGCCCTGAAG-----
TTTACGGACGGTGCTGCGGTTGAGCGCGTATGTTGGG----
ACCCGAAAGATGGTGAACTATGCCTGAATAGGGCGAAGCCAGAGGAAACTCTG-
GTGGAGGCTCGTAGCGATTCTGACGTGCAAATCGATCGTCAAATTTGGG-----------------
----

GGTACTGGTGAGTTCGAGGCTGGTATCTCCAAGGATGGTCAGACTCGCGAGCACGCTCTGCTTG
CATTCACGCTTGGTGTGCGTCAGCTCATCGTCGCGGTCAACAAAATGGATACCACTAAGGTCAG
CATCTAACAATTATG--------GATAATGCTTGGAACT--GACAGCA----------
TTTTTAGTGGTCTGAGGATCGTTTCAACGAAATTGTGAAGGAAACCTCCAACTTCATCAAGAAGG
TCGGTTACAACCCGAAGAATGTCTCCTTCGTCCCCATCTCCGGGTGGCACGGTGACAATATGCT
TGAGGAGTCCCCGAAGTGAATATTACAA-CATCATATTCTT----TTTTTGG-----
GACTATCTAATAATTATA-TTTAGCATGACCTGGTACAAGGGCTGGACCAAGGAGACTAAAG---
---------CCGGTGTCGTC-
AAGGGCAAGACTCTCCTCGATGCTATTGATGCCATTGAACCGCCCGTGCGTCCCCAGGACAAG
CCGCTCCGCCTTCCTCTC---
GAATTTTTGGAAGAATGGGGCTTGGAGAACCTGGAGGAAAATGCTCA---ATCGACAA------
ACCTAACGAAGGTCTTCGTAAACGGTGTCTGGCTTGGCGTTCATCGCGATCCATCCGGTTTGGT
CAAAACTCTCAGGATGCTCAGGCGACGAG---
ATGACATCAGCACCGAAGTCTCAATTGTTCGTGACATTCGCGAGAGGGAATTACGTATCAACAC
CGATGCGGGTCGCGTGTGCAGACCCTTATTCATTGTTGAGGATCACAAGTTGAAGCTGAAGCGC
TCACATCTCCAATGGTTGCAACAAAAGTACAAGGTT-------
GATGATAGACCTTATGTACCGCTT------------
GACGAGAGGGAAGTTGATTCAGAGGGCGAGACTGAGGATGAA---GACGACGAGAGT-------
----
GTCCAGCCGAGAATGAAGAGAAAGCGCAAGCCGTATCGGTGGAACTCACTAGTGAAGGACGG
CGTCATTGAATACCTAGATGCGGAGGAGGAGGAGACAGTCATGATATGCATGACTCCAGATGA
TTTGGTCGAGTCTCGCTTAGTGAAGCAGGGTATAGATCCCCG---
AGCAGATGGTGATTTTGATCCTGCGGCTCGCCTGAAATCTATTCCTCTA------
GCACATACCTGGACACACTGTGAAATTCATCCGAGTATGATTTTGGGTATCTGCGCTAGTATCAT
ACCTTTCCC
>Fuscoporia_ramulicola_Dai_15723
TCGAGTTTTGA------------GTGGGC----------------------------TGCTTGAGCTG--
GCA-------------------------TGCA-GGTG-CTCAGCG------------------------------
GCTC------C-GCTCTCATCC--A--CTC-----------------AA-CCCC--TGTGCA-
CCTTGAATGA---GAG-------------------
-------------------------------------TCGGTCCC--------------------
GATGCTGCATA--

---GCAGAAGGGCCATTC----------
TTGTTTC-TTAC-------------AA---ACTCTATATATTGTCTTGTAATGC---------ATT-
GCCT-CT-TT----GTGGGCGAAATGT-----
TAATACAACTTTCAACAACGGATCTCTTGGCTCTCGCATCGATGAAGAACGCAGCGAAATGCGA

TAAGTAATGTGAATTGCAGAATTCAGTGAATCATCGAATCTTTGAACGCACCTTGCGCTCCTTGG
-TATTCCGAGGAGCATGCCTGTTTGAGTGTCATGTTCTTCTCAATCCCT--------TCG-
GTTTTGCGA---------------------------TCGT-TGGGCTTGGAT-GTGGAGG----------
TCTGCTGGC---------------------------TATGCGGTCGGCTCCTCTGGAATGCATTAGC-
TGGGCTTTCG----------------C-TC---GAT-GCA-----------ACGGTGTAATACTTTC----
---------------TTCAC----CGGTGT-ACCTTG-CGATA-GAGGCTCGC-TTC-----
TAATCGTCGA------------------AAGAC-ACA-----------------------------------
TTGACTCCTTTGACCTCATCCCCTAGTAACTGCGAGTGAAGCGGGAAAAGCTCAAATTTAAAAT
CT-G-ACG-GCTT----TGCTGTCCGAGTTGTAATCTGGAGAAGCGTTTT-CCGCGTTG-
GACTGTGTACAAGTCTCTTGGAACAGAGCGTCATAGAGGGTGAGAATCCCG-
TCCATGCCACAG-AT-CCCCAATGC-
TTTGTGATACGCTCTCGAAGAGTCGAGTTGTTTGGGAATGCAGCTCAAAATGGGTGGTAAGCTC
CATCTAAAGCTAAATATTGGCGAGAGACCGATAGCGAACAAGTACCGTGAGGGAAAGATGAAA
AGCACTTTGGAAAGAGAGTTAAACAGTACGTGAAATTGTTGAAAGGGAAACGCTGGACGTCAG
TCGCGTCTTACTGGACTCAGCCTTGT----TTCGGCTTGGTGTATTTCCTGT-G---
AGACGGGTCAACATCGA-TTTT-GAC-CGGTGGACAAGGGGGTAGGGAATGTGGCAC--
GTCTCGGCGTGTG-TTATAAC-CTTGCCTC-ATAT-GCATCGGTTGGGATCGAGGC-
CCGCAGCGCGCCT------TT---TGGCC-------GGGG------------------------T---TC-
GCCCACG-T-AACGCGCTTAGGAT-
GTTGGCATAATGGCGTTCAGCGACCCGTCTTGAAACACGGACCAAGGAGTCTAACATGCTCGC
GAGTGTTCGGGTGGAAAACCCTTGCGCGAAATGAAAGTGAAT-GTTGAGAG-----CCTC------
------------CGCAA--GGGGGTGCATCGACGCCCGGCCCTGAAG-----
TTCTCTGACGGTGCTGCGGTTGAGCGCGTATGTTGGG----
ACCCGAAAGATGGTGAACTATGCCTGAATAGGGCGAAGCCAGAGGAAACTCTG-
GTGGAGGCTCGTAGCGATTCTGACGTGCAAATCGATCGTCAAATTTGGG------------------
----

GGTACCGGTGAGTTCGAGGCTGGTATCTCGAAGGATGGCCAGACCCGCGAGCACGCTTTGCTT
GCCTTCACGCTCGGTGTGCGTCAGCTCATCGTTGCGGTCAACAAAATGGACACCACCAAGGTTC
GTTGCACCAGTAGG---CGAAAGTGTGGATTTTCTAATTG--TTGCCTT----------
GTATTAGTGGTCTGAGGATCGTTTCAATGAAATTATCAAGGAAACGTCCAACTTCATCAAGAAG
GTCGGTTACAACCCGAAGGCTGTCTCCTTCGTCCCGATTTCTGGATGGCACGGTGACAACATGC
TTGAGGAGTCATCCAAGTACGGTCCTACA-AATCTTA-------TAATCGTGG-----
ACATAAATCTTATCTTCT-TATAGCATGCCCTGGTACAAAGGCTGGACCAAGGAGACCAAGG---
---------CTGGTGTCGTC-
AAGGGTAAGACCCTCCTTGACGCCATCGATGCCATCGAGCCGCCTGTCCGTCCCCAGGATAAG
CCCCTCCGTCTCCCCCTCCAGGATGTC---------------------------------

GAATTCTTGGAGGAATGGGGTCTGGAAAACCTTGAAGAAAATGCCCA---GTCAACTA------
ACCTGACGAAGGTGTTCGTGAACGGTGTCTGGCTGGGAGTGCATCGTGATCCTTCTGGTCTCGT
CAGGACGCTCAGAATGCTCAGACGAAGGG---
ACGATATCAGCACTGAGGTCTCTATTGTCCGTGATATCCGCGAGAAGGAGCTGCGTATTAATAC
AGACGCTGGACGTGTCTGCAGGCCGTTGTTTATAGTGGAGGATCACGAGCTTAAATTGAAGCGG
CAGCATCTGCGATGGTTACAGCAAAAATTCAGGGTC-------GAAGAAAGGCCTTACACA-----
-------------GAAGATGGAGATGTCCTTTCCGAGGGGGAAACAGAAGACGAA---
GACGCTGAAAGC---------------
GTTCAGCCTAGAATGAAGAGGAAACGGAAGCCGTACAGGTGGGGTTCTTTAGTAAAGGATGGT
GTCATCGAATACCTGGATGCTGAGGAAGAGGAGACAGTCATGATTTGCATGACACCGGATGATT
TAGTGGAATCCCGGTTAATGAAGCAAGGCCTTGACGCTCG---
ACACGATGGAGACTTTGATCCGGCGGCTCGTCTAAAATCTGTGCCGATG------
GCTCATACTTGGACGCATTGTGAAATCCATCCTAGCATGATTCTTGGTATTTGCGCTAGCATCATT
CCATTCCC
>Fuscoporia_rhabarbarina_Dai_16550
----------------------TGTGGGT----------------------------CTCTTGAGCTG--
GCTT-----------------------TGCA-GGTG-CTCAGAG-----------------------------
GCTC------CCATTCTCATCC--A--CTC-----------------AA-CCCC--TGTGCA-
CTTTTGAACGC---AAG-------------------
------------------------------------TGAGTCGT----------------C----
AGTTCCCCTAAGTT--

---GGGAGTGACTTGA---TTTGC----
TTCGTTTA-TTAT-------------AA---ACTCTTTTT-ATGTCTTGTAGAAT-G-----C-TTT-
GCCT-CT-CT----GTAGGCGAAAT------
GTAATACAACTTTCAACAACGGATCTCTTGGCTCTCGCATCGATGAAGAACGCAGCGAAATGCG
ATAAGTAATGTGAATTGCAGAATTCAGTGAATCATCGAATCTTTGAACGCACCTTGCGCTCCTTG
G-TATTCCGAGGAGCATGCCTGTTTGAGTGTTATGTTATCCTCAATCCCC--------TTG-
GTTTTGTTAAA----------ATAA--GACCTC--------TGGGTTTGGTT-TTGGA--------------
----

-------------------ACATCGA-TTT---GC-CGGTGGACAAGGG-GTAAGGAATGTGGCAC--

TCTTTGGAGTGTG-TTATAGC-CTTTC-TC-ATAT-ACATCGGTTGGGATTGAGGC-
CCGCAGCGCGCCC------TT-G-TGGCC-------GGAGG-----------------------TT---CG-
CCTCACG-T-AACGCGCTTAGGAT-
GTTGGCATAATGGCTTTAAGCGACCCGTCTTGAAACACGGACCAAGGAGTCTAACATGCTCGCG
AGTGTTAGGGTGGAAAACCCTTGCGCGTAATGAAAGTGAAA-GTTGAGAA-----CCTC-------
-----------CGCAA--GGGGGTGCATCGACGCCCGGCCCTGAAG-----
TTTACTGACGGTGCTGCGGTAGAGCGCGTATGTTGGG----
ACCCGAAAGATGGTGAACTATGCCTGAATAGGGCGAAGCCAGAGGAAACTCTG-
GTGGAGGCTCGTAGCGATTCTGACGTGCAAATCGATCGTCAAATTTGGG-------------------
----

GGYACTGGTGAGTTCGAGGCTGGTATCTCYAAGGATGGCCAGACCCGCGAGCACGCTCTGCTT
GCTTTCACGCTCGGTGTGCGTCAGCTCATTGTTGCGGTCAACAAAATGGACACCACCAAGGTCC
GTAAYCATTACGAT--ATAAAATCGCACCCCTTCTAAGTT--TCTTGTT----------
ACTTCAGTGGTCTGAGGAACGCTTCAAYGAAATCGTTAAGGAAACGTCCAACTTTATCAAGAAA
GTCGGTTACAACCCGAAGGCCGTCTCTTTCGTCCCGATCTCTGGATGGCATGGTGACAACATGC
TTGAGGAGTCTAGCAAGTATGAACTATTA-TATTTTG---------ACATTGT-----
AATTTTTACTAACCTTTC-TGTAGCATGCCATGGTACAAGGGCTGGACCAAGGAGACCAAGG---
---------GCGGTTCTGTC-
AAGGGCAAGACGCTCCTCGATGCTATTGATGCCATTGAGCCGCCTGTCCGTCCCCAGGATAAGC
CCCTCCGTCTTCCTCTCCAGGATGTG----------------------------------
GAATTCTTAGAAGAGTGGGGCCTAGAAAACCTGGAGGAAAACGCTCA---ATCGACCA------
ATTTGACGAAGGTCTTTGTCAATGGTGTCTGGTTGGGTGTTCATCGTGATCCGTCTGGTCTAGTC
AGAACTCTCAGGATGCTCAGACGAAGGG---
ACGACATAAGTACCGAGGTTTCCATTGTCCGTGATATCCGCGAGCGAGAGTTGCGCATTAATAC
GGACGCGGGYCGTGTGTGCAGGCCGCTGTTTATCGTAGAAGACCACAAGCTGAAGCTGAAGCG
TCAACACCTAAGATGGCTTCAGCAGAAGTTCAGGGTT-------GAGGAAAGGCCTTACTCA----
--------------GACGAAAGAGATGTTCTCTCAGAAGGCGAAACGGAAGATGAG---
GATGCTGAAGGC---------------
ACACAACCTCGTATGAAGAGGAAACGGAAGCCGTATAGATGGGGCTCTCTGGTAAAAGACGGT
GTCATCGAATACCTGGATGCTGAAGAGGAAGAGACAGTCATGATTTGCATGACTCCGGATGATT
TGGTCGAATCTCGTTTGATGAGGCAAGGTATTGACCCRCG---
ACAGGACGGGGATTTCGATCCGGCAGCTCGTCTTAAATCTGTGCCTATG------
GCTCATACATGGACGCACTGCGAAATCCACCCAAGCATGATACTCGGCATTTGTGCGAGTATTA
TTCCTTTCCC
>Hirschioporus_acontextus_Dai_23793
ACGTGTCGAAG-----A-CTAAGCAAGG-------------------------------TCATGCGCGA--

ATATA----------------------AGGCA-TGTG-CTCAGC------------------------------
-CCC--------GCTCCAATCT--A--CTT-----------------AA-CCCC--TGTGCA-
CTATACGG--AGT--------------------
-----------------------------------GTCGCAAGTTGAGA----------C--------------
---------------------------TTGAATTA---------------------------------------
----

-------------GAGGCCTTAGTC------------GCATTTGTAA-------TGTGGCTGGGTG-----
-----------------TCTATC-----------TAAGTC-GGTCAAGGCTTGTCC-TCCGG------
TCTATA--TAC-------------AA---ACTCTTATA-ATGTCTTGTAGAAT-G-----T-
GTCAGCCT-CT-TG----TTAGGCGAAACTC-----
AAATACAACTTTCAACAACGGATCTCTTGGCTCTCGCATCGATGAAGAACGCAGCGAAATGCGA
TAAGTAATGTGAATTGCAGAATTCAGTGAATCATCGAATCTTTGAACGCACCTTGCGCTCCTTGG
-TATTCCGAGGAGCATGCCTGTTTGAGTGTCATGTTAATATCAACCCCG--------TCG-
GTTTGTTAA------------TCAGA----------CCGTTGGGTGTTGGAC-TTGGAGG-T-T------
CTTGCTGGC---------------------------TGCAAAGTCGGCTCCTCTTGAATGCATTAGC-
TTGGGCCTGT--------------TGT-GC---GTG-CTC-----------GCGGTGTAATAAGTTTTA--
---------------TTCATCGTTTTGGGT-CGTACA-CCTAT-TGGGCTTGC-TTC-----
TAACTGTCTT------------------C--
---ATTTAAAATCT-G-GCG-GTTT---
-CACCGTCCGAGTTGTAATCTGGAGAAGCGTTTT-CCGTGTCG-
GACCGTGCACAAGTCTCTTGGAACAGAGCGTCATAGAGGGTGAGAATCCCG-
TTTATGGCACGG-AC-AACCGATAC-
TTTGTGATACGTTTTCTAAGAGTCGAGTTGTTTGGGAATGCAGCTCAAAATGGGTGGTAAATTCC
ATCTAAAGCTAAATATAGGCGAGAGACCGATAGCGAACAAGTACCGTGAGGGAAAGATGAAA
AGCACTTTGGAAAGAGAGTTAAACAGTACGTGAAATTGTTGAAAGGGAAACGCTTGAAGTCAG
TCGCGTCTTCTAGGACTCAGCCTTGC----TTCGGTTAGGTGTACTTCCTGGTT---
TGACGGGTCAACATCGA-TTTT-GAT-CGACGGATAAAGGAGAAGGGAACGTGGCACCTCC---
-GGGTGTG-TTATAGC-CCTCT-TC-GTAT-ACGTCGATTGGGATCGAGGA-
CTGCAGCACGCCC------TT-G-TGGCC-------GGGGG----------------------TT---CG-
CCCTACG-T-ATCGTGCTTAGGAT-
GTTGACATAATGGCTTTAAGCGACCCGTCTTGAAACACGGACCAAGGAGTCTAACATGCTCGCG
AGTGTTAGGGTGGAAAACCCTTGCGCGTAATGAAAGTGAAA-GTTGGGA------CCTC-------
-----------TGCGA--GGAGG-GCACCGACGCCCGGCCCTGAAG-----
TTTTCTGACGGTGCTGCGGTAGAGCGCGTATGTTGGG----
ACCCGAAAGATGGTGAACTATGCCTGAATAGGGCGAAGCCAGAGGAAACTCTG-
GTGGAGGCTCGTAGCGATTCTGACGTGCAAATCGATCGTCAAATTTGGGTTGGTGTGTC--AT--
-CGAGGATAACGTAAGGGAAAAT-
AATGATATTACCTTACTATTAGTGTCGTCCAAGTACTCGTGCCAGAAGACTCGGTAAGGCGAGA
GACGCAAACGTTAGTCGTCTTGATCAGGCGTAAAGGGTTTGTAGGCGGCTTTAAGTATCTTA-
TTTTTAGTTTACTATTTT---------------------------------AATGTTTATA-------------

ATTATAATTGAATTTTTAATGATAAAAAGAAAAATTTTTAAATATTGGTTCGGGCTTTAGTCTGCC
ATTCATTTATAGAATTTACTAAATTAATAATTTAACATTAAAAAAATAAAGAAAGGATCAATTAAA
GCTAGAATCTAAAAGAGGTTA----AAACAATACTCTTC--
AAAGGAGTGCTAACCGTAGATAAGGAGCAGAGTGT-TAAGGGCGAAGGC-
ATTTATCCAATAAAGATTGACGCTGAGAAACGAAGGTGAGGGCAGAAAAAAGGATTAGAGACC
CTTGTATTCCTCACTGTCAACGATGAATGGTAGTTACTAGGTAGAT-----------------------
----
ATGACCTAGAGACGATGTTAACACGTAAACCATTCCGCCTTGTGAGTACGACTGCAAAGTTGAA
AACAAAAAAATTAGTCGGTTTCGGAG--CAAACGAAGTGAAGC-A------------
GGTACTGGTGAGTTCGAAGCTGGCATCTCCAAGGATGGCCAGACGCGTGAGCACGCTTTGCTC
GCGTTTACCCTCGGTGTTCGGCAACTCATTGTCGCCGTCAACAAAATGGACACGACGAAGGTTA
GTTTATACAATCAG--ATTCTAATCCTATCTTCTATTCTAA-------------
CATATGTTATAGTGGTCTGAGGACCGTTTCAACGAAATCGTCAAGGAAACGTCTAGCTTCATCA
AGAAGGTCGGTTTCAATCCCAAGGCTGTTGCTTTTGTTCCGATTTCAGGCTGGCACGGTGACAA
CATGATTGAGGAGTCTACCAAGTATGTGTGAAGT-ACGTTG----------ACCAAAG-----
ATTGTTATTGATTGTTAT-TGCAGCATGCCCTGGTACAAGGGCTGGACCAAGGAAACGAAGG---
---------GGGGTRTTKTG-AAGGGCAAGACGTTGCTCGATGCCATTGATGCCATTGAACCT----
----

>Hirschioporus_chinensis_Dai_20264
TTATATTTTAC-----A-CT---TTAGG------------------------------TTGCGCGCGG--
GAGAA---------------TT-ACAATAGCA-TGTG-CTCAGC-----------------------------
--CTC--------CCTCTTATCC--A--CTC-----------------AA-CCCC--TGTGCA-
CTATTCGG--AGT-------------------
------------------------------------GTTGCAAGCTGAGA----------C--------------
----------------------------TTGAAT--

-------------AGGTCTTGGTC------------GCGTTTCTAA-------TGCGGCTGGGT--------
------------------TC-----------TAAGAC-AGTCAGGGCTTGTCC-TCCGG------
TCTATA--TAC-------------AA---ACTCTTATA-ATGTCTTGTAGAAT-G-----T-GTTAGCCT-
CT-CG----TTAGGCGAAACTT-----

AAATACAACTTTCAACAACGGATCTCTTGGCTCTCGCATCGATGAAGAACGCAGCGAAATGCGA
TAAGTAATGTGAATTGCAGAATTCAGTGAATCATCGAATCTTTGAACGCACCTTGCGCTCCTTGG
-TATTCCGAGGAGCATGCCTGTTTGAGTGTCATGTTAATATCAACCCCG--------TCG-
GTTTGTAAA------------TCAGA----------CCGTTGGGTGTTGGAC-TTGGAGG-T-T------
CTTGCTGGC---------------------------TGCAAAGTCGGCTCCTCTTGAATGCATTAGC-
TTGGGCCTGT--------------TGT-GC---GTG-CTC-----------GCGGTGTAATAAGTTTTA--
----------------TTCATCGT-TTGGGT-CGTACA-CTTAT-TGGGCTTGC-TTC-----
TAACTGTCTT------------------C---
-----------------CCTAGTAACTGCGAGTGAAGCGGGAAAAGCTCAAATTTAAAATCT-G-
GCG-GTTT----CACCGTCCGAGTTGTAATCTGGAGAAGCGTTTT-CCGTGTCG-
GACCGTGCACAAGTCTCTTGGAACAGAGCGTCATAGAGGGTGAGAATCCCGTTTTATGGCACG
G-AC-ACCCGATAC-
TTTGTGATACGTTTTCTAAGAGTCGAGTTGTTTGGGAATGCAGCTCAAAATGGGTGGTAAATTCC
ATCTAAAGCTAAATATAGGCGAGAGACCGATAGCGAACAAGTACCGTGAGGGAAAGATGAAA
AGCACTTTGGAAAGAGAGTTAAACAGTACGTGAAATTGTTGAAAGGGAAACGCTTGAAGTCAG
TCGCGTCTTCTAGGACTCAGCCTTAC----TTCGGTTAGGTGTACTTCCTGGTT---
TGACGGGTCAACATCGA-TTTT-GAT-CGACGGATAAAGGAGAAGGGAATGTGGCACCTCC----
GGGTGTG-TTATAGC-CCTCT-TC-GTAT-ACGTCGATTGGGATCGAGGA-CTGCAGCACGCCC-
-----TT-G-TGGCC-------GGGGG----------------------TT---CG-CCCTACG-T-
ATCGTGCTTAGGAT-
GTTGACATAATGGCTTTAAGCGACCCGTCTTGAAACACGGACCAAGGAGTCTAACATGCTCGCG
AGTGTTAGGGTGGAAAACCCTTGCGCGTAATGAAAGTGAAA-GTTGGGA------CCTC-------
-----------TGCAA--GGAGG-GCACCGACGCCCGGCCCTGAAG-----
TTCTCTGACGGTGCTGCGGTAGAGCGCGTATGTTGGG----
ACCCGAAAGATGGTGAACTATGCCTGAATAGGGCGAAGCCAGAGGAAACTCTG-
GTGGAGGCTCGTAGCGATTCTGACGTGCAAATCGATCGTCAAATTTGGGTCGGTGTGTC--AT--
-CGAGGATAACGTAAGGGAAAAT-
AATGATATTACCTTACTATTAGTGTCGTCCAAGTACTCGTGCCAGAAGACTCGGTAAGGCGAGA
GACGCAAACGTTAGTCGTCTTGATCAGGCGTAAAGGGTTTGTAGGCGGCTTTAAGTATCTTA-
TTTTTAGTTTACTATTTT--------------------------------AATGTTTATA-------------
ATTATAATTGAATTTTTAATGATAAAAGAAAA---TTTTAAATATTGG---------
GAGTTTTTCCTTCATTTATAAATTTTACTAAATTAATAATTTTACATTAAAAAAATAAAGAAAGGAT
CAATTAAAGCTAGAATCTAAAAGAGGTTA----AAACAATACTTTAG--
AAAGGAGTGCTAACCGTAGATAATGAGCAGAGTGT-TAAGGGCGAAGGC-
ATTTATCCAATAAAGATTGACGCTGAGAAACGAAGGTGAGGACAGAAAAAAGGATTAGAGACC
CTTGTATCCCTCACTGTCAACGATGAATGGTAGTTACTAGGTAGTT----------------------
----
ATGACCTAGAGACGATGTTAACACGTAAACCATTCCGCCTTGTGAGTACGACTGCAAAGTTGAA
AACAAAAAAATTAGTCGGTTTCGGAG--CAAACGAAGTGAAGC-A------------
GGTACTGGTGAGTTCGAGGCTGGTATCTCCAAGGACGGCCAAACGCGTGAGCACGCTTTGCTC
GCGTTTACCCTCGGTGTGCGACAACTCATTGTCGCCGTCAACAAAATGGACACGACGAAGGTTT
GTTTGTCCGACAAG--TTTTCAACCCTATTAACGATACTA--AATTTTG----------
CGTATAGTGGTCTGAGGATCGTTTCAACGAAATTGTCAAGGAAACGTCCAACTTCATTAAGAAG
GTCGGCTACAACCCCAAGTCCGTCGCTTTCGTTCCGATCTCCGGCTGGCACGGTGACAACATGC

TTGAGGAGTCTGCAAAGTAAGCGCTCAAA-GGTTGGT-------GGTATGTTA-----
TTCGTTGCTGATTATCGT-CATAGCATGCCTTGGTACAAGGGCTGGACCAAGGAGACGAAGG--
----------CCGGTGCGAGC-
AAGGGCAAAACTCTCCTCGATGCGATTGATGCCATCGATCCTCCGGTTCGTCCGAGTGACAAGC
CCCTCCGTCTTCCTCTCCAGGATGTG--

>Hirschioporus_pubescens_Dai_23710
ACATTTCTACA-----G-TT---TCGGG-------------------------------TTGCGCGCGG--
GTCAC---------------TCAATAATAGCA-TGTG-CTCAGC------------------------------
--CTC--------CGTCTTATCC--A--CTC-----------------AA-CCCC--TGTGCA-
CTATTCGG---AGT-------------------
-------------------------------------GTTGCAAGCTGAGA----------C--------------
-----------------------------TTGAAT---

-------------AGGTCTTGGTC------------GCGTTTCTAA-------TGCGACTGGGT--------
------------------TC-----------TAAGAC-AGTCAAGGCTTGTCC-TCTGG------TCTATA-
-TAC-------------AA---ACTCTTATA-TTGTCTTGTAGAAT-G-----T-GTTAGCCT-CT-CG--
--TTAGGCGAAACTT-----
AAATACAACTTTCAACAACGGATCTCTTGGCTCTCGCATCGATGAAGAACGCAGCGAAATGCGA
TAAGTAATGTGAATTGCAGAATTCAGTGAATCATCGAATCTTTGAACGCACCTTGCGCTCCTTGG
-TATTCCGAGGAGCATGCCTGTTTGAGTGTCATGTTAATATCAACCCCG--------TCG-
GTTTGTAAA------------TCAGA----------CCGTTGGGTGTTGGAC-TTGGAGG-T-T------
CTTGCTGGC----------------------------TGCAAAGTCGGCTCCTCTTGAATGCATTAGC-
TTGGGCCTGT--------------TGT-GC---GTA-CTC-----------GCGGTGTAATAAGTTTTA--
---------------TTCATCGT-TTGGGT-CGTACA-CTTAT-TGGGCTTGC-TTC-----
TAACTGTCTT------------------CGGAC-AA--------GC-----------------------------
-------------------CCTAGTAACTGCGAGTGAAGCGGGAAAAGCTCAAATTTAAAATCT-G-
GCG-GTTT----CACCGTCCGAGTTGTAATCTGGAGAAGCGTTTT-CCGTGTCG-
GACCGTGCACAAGTCTCTTGGAACAGAGCGTCATAGAGGGTGAGAATCCCGTTTTATGGCACG
G-AC-ACCCGATAC-
TTTGTGATACGTTTTCTAAGAGTCGAGTTGTTTGGGAATGCAGCTCAAAATGGGTGGTAAATTCC

ATCTAAAGCTAAATATAGGCGAGAGACCGATAGCGAACAAGTACCGTGAGGGAAAGATGAAA
AGCACTTTGGAAAGAGAGTTAAACAGTACGTGAAATTGTTGAAAGGGAAACGCTTGAAGTCAG
TCGCGTCTTCTAGGACTCAGCCTTAC----TTCGGTTAGGTGTACTTCCTGGTT---
TGACGGGTCAACATCGA-TTTT-GAT-CGACGGATAAAGGAGAAGGGAATGTGGCACCTCC----
GGGTGTG-TTATAGC-CCTCT-TC-GTAT-ACGTCGATTGGGATCGAGGA-CTGCAGCACGCCC-
-----TT-G-TGGCC-------GGGGG----------------------TT---CG-CCCTACG-T-
ATCGTGCTTAGGAT-
GTTGACATAATGGCTTTAAGCGACCCGTCTTGAAACACGGACCAAGGAGTCTAACATGCTCGCG
AGTGTTAGGGTGGAAAACCCTTGCGCGTAATGAAAGTGAAA-GTTGGGA------CCTC-------
-----------TGCAA--GGAGG-GCACCGACGCCCGGCCCTGAAG-----
TTCTCTGACGGTGCTGCGGTAGAGCGCGTATGTTGGG----
ACCCGAAAGATGGTGAACTATGCCTGAATAGGGCGAAGCCAGAGGAAACTCTG-
GTGGAGGCTCGTAGCGATTCTGACGTGCAAATCGATCGTCAAATTTGGGTCGGTGTGTC--AT--
-CGAGGATAACGTAAGGGAAAAT-
AATGATATTACCTTACTATTAGTGTCGTCCAAGTACTCGTGCCAGAAGACTCGGTAAGGCGAGA
GACGCAAACGTTAGTCGTCTTGATCAGGCGTAAAGGGTTTGTAGGCGGCTTTAAGTATCTTA-
TTTTTAGTTTACTATTTT--------------------------------AATGTTTATA------------
ATTATAATTGAATTTTTAATGATAAAAGAAAA---TTTTAAATATTGG---------
GAGTTTTTCCTTCATTTATAAATTTTACTAAATTAATAATTTTACATTAAAAAAATAAAGAAAGGAT
CAATTAAAGCTAGAATCTAAAAGAGGTTA----AAACAATACTTTAG--
AAAGGAGTGCTAACCGTAGATAATGAGCAGAGTGT-TAAGGGCGAAGGC-
ATTTATCCAATAAAGATTGACGCTGAGAAACGAAGGTGAGGACAGAAAAAAGGATTAGAGACC
CTTGTATCCCTCACTGTCAACGATGAATGGTAGTTACTAGGTAGTT-----------------------
----
ATGACCTAGAGACGATGTTAACACGTAAACCATTCCGCCTTGTGAGTACGACTGCAAAGTTGAA
AACAAAAAAATTAGTCGGTTTCGGAG--CAAACGAAGTGAAGC-A------------
GGTACTGGTGAGTTCGAGGCTGGTATCTCCAAGGATGGCCAAACGCGTGAGCACGCTTTGCTC
GCGTTCACCCTCGGTGTGCGGCAACTCATTGTCGCCGTCAACAAAATGGACACGACGAAGGTTT
GTTTGTCCGACAAC--TTTCTAACCCTATCAACGATTCTA--AAATTTG----------
CGTATAGTGGTCTGAGGATCGTTTCAACGAAATCGTCAAGGAAACGTCCAACTTCATTAAGAAG
GTCGGCTACAATCCCAAGTCCGTCGCGTTCGTTCCGATTTCCGGCTGGCACGGTGACAACATGC
TCGAGGAGTCTCCTAAGTAAGTG--CAAA-GGCCGGC-------GGTATAAGA-----
TCCGTTGCTAATTATTAT-CGTAGCATGCCTTGGTACAAGGGCTGGACCAAGGAGACGAAGG--
----------CTGGTGCGAGC-
AAGGGCAAAACTCTCCTCGATGCTATTGATGCCATCGATCCTCCGGTTCGTCCGAGTGACAAAC
CCCTCCGTCTTCCTCTCCAGGATGTG--

>Hirschioporus_tianschanicus_Dai_19067
TTATTTATTAC-----A-CT---TTAGG------------------------------TT--GCGCGG--
GAAAA---------------TT-ACAATAGCA-TGTG-CTCAGC-----------------------------
--CTC--------CCTCTTATCC--A--CTC-----------------AA-CCCC--TGTGCA-
CTATTCGG--AGT-------------------
------------------------------------GTTGCAAGCTGAGA----------C--------------
----------------------------TTGAAT--

-------------AGGTCTTGGTC------------GCGTTTCTAA-------TGCGGCTGGGT--------
------------------TC-----------TAAGAC-AGTCAAGGCTTGTCC-TCCGG------
TCTATA--TAC-------------AA---ACTCTTATA-TTGTCTTGTAGAAT-G-----T-GTTAGCCT-
CT-CG----TTAGGCGAAACTT-----
AAATACAACTTTCAACAACGGATCTCTTGGCTCTCGCATCGATGAAGAACGCAGCGAAATGCGA
TAAGTAATGTGAATTGCAGAATTCAGTGAATCATCGAATCTTTGAACGCACCTTGCGCTCCTTGG
-TATTCCGAGGAGCATGCCTGTTTGAGTGTCATGTTAATATCAACCCCG--------TCG-
GTTTGTAAA------------TCAAA----------CCGTTGGGTGTTGGAC-TTGGAGG-T-T------
CTTGCTGGC---------------------------TGCAAAGTCGGCTCCTCTTGAATGCATTAGC-
TTGGGCCTGT--------------TGT-GC---GTG-CTC-----------GCGGTGTAATAAGTTTTA--
---------------TTCATCGT-TTGGGT-CGTACA-CTTAT-TGGGCTTGC-TTC-----
TAACTGTCTT------------------C--
----------------CCTAGTAACTGCGAGTGAAGCGGGAAAAGCTCAAATTTAAAATCT-G-
GCG-GTTT----CACCGTCCGAGTTGTAATCTGGAGAAGCGTTTT-CCGTGTCG-
GACCGTGCACAAGTCTCTTGGAACAGAGCGTCATAGAGGGTGAGAATCCCGTTTTATGGCACG
G-AC-ACCCGATAC-
TTTGTGATACGTTTTCTAAGAGTCGAGTTGTTTGGGAATGCAGCTCAAAATGGGTGGTAAATTCC
ATCTAAAGCTAAATATAGGCGAGAGACCGATAGCGAACAAGTACCGTGAGGGAAAGATGAAA
AGCACTTTGGAAAGAGAGTTAAACAGTACGTGAAATTGTTGAAAGGGAAACGCTTGAAGTCAG
TCGCGTCTTCTAGGACTCAGCCTTAC----TTCGGTTAGGTGTACTTCCTGGTT---
TGACGGGTCAACATCGA-TTTT-GAT-CGACGGATAAAGGAGAAGGGAACGTGGCACCTCC---
-GGGTGTG-TTATAGC-CCTCT-TC-GTAT-ACGTCGATTGGGATCGAGGA-
CTGCAGCACGCCC------TT-G-TGGCC-------GGGGG----------------------TT---CG-
CCCTACG-T-ATCGTGCTTAGGAT-
GTTGACATAATGGCTTTAAGCGACCCGTCTTGAAACACGGACCAAGGAGTCTAACATGCTCGCG
AGTGTTAGGGTGGAAAACCCTTGCGCGTAATGAAAGTGAAA-GTTGGGA------CCTC-------
-----------TGCGA--GGAGG-GCACCGACGCCCGGCCCTGAAG-----
TTCTCTGACGGTGCTGCGGTAGAGCGCGTATGTTGGG----
ACCCGAAAGATGGTGAACTATGCCTGAATAGGGCGAAGCCAGAGGAAACTCTG-
GTGGAGGCTCGTAGCGATTCTGACGTGCAAATCGATCGTCAAATTTGGGTCGGTGTGTC--AT--

-CGAGGATAACGTAAGGGAAAAT-
AATGATATTACCTTACTATTAGTGTCGTCCAAGTACTCGTGCCAGAAGACTCGGTAAGGCGAGA
GACGCAAACGTTAGTCGTCTTGATCAGGCGTAAAGGGTTTGTAGGCGGCTTTAAGTATCTTA-
TTTTTAGTTTACTATTTT--------------------------------AATGTTTATA-------------
ATTATAATTGAATTTTTAATGATAAAAGAAAA---TTTTAAATATTGG---------
GAGTTTTTCCTTCATTTATAAATTTTACTAAATTAATAATTTTACATTAAAAAAATAAAGAAAGGAT
CAATTAAAGCTAGAATCTAAAAGAGGTTA----AAACAATACTTTAG--
AAAGGAGTGCTAACCGTAGATAATGAGCAGAGTGT-TAAGGGCGAAGGC-
ATTTATCCAATAAAGATTGACGCTGAGAAACGAAGGTGAGGACAGAAAAAAGGATTAGAGACC
CTTGTATCCCTCACTGTCAACGATGAATGGTAGTTACTAGGTAGTT-----------------------
----
ATGACCTAGAGACGATGTTAACACGTAAACCATTCCGCCTTGTGAGTACGACTGCAAAGTTGAA
AACAAAAAAATTAGTCGGTTTCGGAG--CAAACGAAGTGAAGC-A------------
GGTACTGGTGAGTTCGAGGCTGGTATCTCCAAGGACGGCCAAACGCGTGAGCACGCTTTGCTC
GCGTTTACCCTCGGTGTGCGACAACTCATTGTCGCCGTCAACAAAATGGACACGACGAAGGTTT
GTTTGTCCGACAAG--TTTTCAACCCTATTAACGATTCTA--AAATTTG----------
CGTATAGTGGTCTGAGGATCGTTTCAACGAAATCGTCAAGGAAACGTCCAACTTCATTAAGAAG
GTCGGCTACAACCCCAAGTCCGTCGCTTTCGTTCCGATCTCCGGCTGGCACGGTGACAACATGC
TTGAGGAGTCTGCAAAGTAAGCGCTCAAA-GGTTGGT-------GGTATGTGA-----
TTCGTTGCTGATTACCAT-CGTAGCATGCCTTGGTACAAGGGCTGGACCAAGGAGACGAAGG--
----------CCGGTGCGAGC-
AAGGGCAAAACTCTCCTCGATGCGATTGATGCCATCGATCCTCCGGTTCGTCCGAGTGACAAGC
CCCTCCGTCTTCCTCTCCAGGATGTG--

>Hydnoporia_gigasetosa_He_1442
TCGAGTTTTGA-----A-----GTAAGG------------------------------CTTGATGCTG--
GTGCG---------------GAAACG--TACA-TGTG-CTCAGT----------------------------
--CTT--ACA-----CTCATTCC--A--CTC-----------------AA-CACC--TGTGCA--
CTTATCG--AAG-------------------
----------------------------------TTAGTAGT---------------C------GTTCCTC---
------------------------CTTTCA-----------------TTAGGAGCCGTTGAGAGGAGAG--
----------------------------------TCTCGCAA---------------------------------
----
-------------------------GTAATCATT--
----------------------------------AGGAAG---GTTGATT------------------------

----
---------------------------TATTGACT--------------TTCTTAAA-------GGTGAAAGG-
------------------CGGGGTCACCT------------TGACT-GGGAGCGGTGAAACC-TTTGA--
------CTTA-TTAT-------------AA---ACCTTTTAC-TTGTCTTGTAGAAT-G-----T-AAT-
GC-T-CC-TC----GTGGGCGAAATGA-----
AAATACAACTTTCAACAACGGATCTCTTGGCTCTCGCATCGATGAAGAACGCAGCGAAATGCGA
TAAGTAATGTGAATTGCAGAATTCAGTGAATCATCGAATCTTTGAACGCACCTTGCGCCCTTTGG
-TATTCCGAAGGGCATGCCTGTTTGAGTGTCATGTTAATCTCAATATCC--------TTG-
TTTTCTTAA------------TTGA--AGACTTG-------GAGCATTGGAC-TTGGAGG-T-T------
TATGCTGGC-CCCT----------------------TTTGTGGTCGGCTCCTCTTAAATGTATTAGT-
TGGACTTTGG----------------T-TC---GCG-T-G-----------AAGATGTAATAG---------
---------TTTA-TTCAT----CATTAG-CGCTTG-CCTAA-TGGGTCTGC-TTC-----
TAATCGTCTT------------------GGGAC-AA--------AG------ATTAAC-TTC----T-TTC--
TTGACTCTTTGACCTCATCCCCTAGTAACTGCGAGTGAAGCGGGAAAAGCTCAAATTTAAAATCT
-G-GCG-TTC--TTTGGACGTCCGAGTTGTAATCTGGAGAAGCGTTTT-CCGTGTTG-
GACCGTGTACAAGTCTCCTGGAACGGAGCGTCATAGAGGGTGAGAATCCCG-
TCCATGACACGG-AC-TACCAATGC-
TTTGTGATACGCTCTCGAAGAGTCGAGTTGTTTGGGAATGCAGCTCAAAATGGGTGGTAAATTC
CATCTAAAGCTAAATATTGGCGAGAGACCGATAGCGAACAAGTACCGTGAGGGAAAGATGAAA
AGCACTTTGGAAAGAGAGTTAAACAGTACGTGAAATTGTTGAAAGGGAAACGCTTGAAGTCAG
TCGCGTCTTGCAGGACTCAGCCTTAC----TTCGGTTTGGTGTACTTTCTGT-T---
TGACGGGTCAACATCAA-TTTT-GAT-
CGGTAGAAAAAGCTTGAAGAAATGTAGCGTTGTTTCGGCGACGTG-TTATAGT-CTTCTTGT-
GTAT-ATATCGGTTGGGATTGAGGA-CCGCAGCACGCCT------TTAA-TGGCC-------
GGGGG----------------------TT---CG-CCCTACG-T-ATCGTGCTTAGGAT-
GTTGGCATAATGGCTTTAAGCGACCCGTCTTGAAACACGGACCAAGGAGTCTAACATGCTTGCG
AGTGTTCGGGTGGAAAACCCTTGCGCGTAATGAAAGTGAAA-GTTGGGAA-----CCTC-------
-----------CGCAA--GGGGGTGCACCGACGCCCGGCCCTGAAG-----
TTCTCTGACGGTGCTGCGGTAGAGCACGTATGTTGGG----
ACCCGAAAGATGGTGAACTATGCCTGAATAGGGCGAAGCCAGAGGAAACTCTG-
GTGGAGGCTCGTAGCGATTCTGACGTGCAAATCGATCGTCAAATTTGGG------------------
----

>Hydnoporia_lamellata_Cui_7629
TCGAGTTTTGA-----A----G--CAGG------------------------------CTTGATGCTG--
GTGCG---------------GAAACG--TACA-TGTG-CTCGGT-----------------------------
--CTT--GCG-----CTCATTCC--A--CTC-----------------AA-CCCC--TGTGCA--
CTTATCG--AAG--------------------
-----------------------------------TTAGTAGT----------------C------GTTCCTC---
-------------------------CTTC-------------------GTTGGAGCCGTCGAGAG---------
-----------------------------------TCGCGA------------------------------------
----
------------------------GTAATCATT--
---------------------------------AGGAA---------------------------------------
----
-------------------GTCTTCATTGACT-------------TT--TGAAA-------GGTGGAAGC---
--------------------GTCGCTT------------TGACC-GGGAGCGGTGAAACC-TTTGA-----
--CTTTA-CTAT--------------A---AACCTTTAT-TTGTCTTGTAGAAT-G-----T-AAT-GC-T-
CC-TC----GTGGGCGAAAT------
TAAATACAACTTTCAACAACGGATCTCTTGGCTCTCGCATCGATGAAGAACGCAGCGAAATGCG
ATAAGTAATGTGAATTGCAGAATTCAGTGAATCATCGAATCTTTGAACGCACCTTGCGCCCTTTG
G-TATTCCGAAGGGCATGCCTGTTTGAGTGTCATGTTAATCTCAATCCCC--------TTG-
TTTCTTAATTG----------------AGGCTT----AAG-GTGGATTGGAC-TTGGAGG----------
TTTGCTGGC-CCTA---------------------TGTGGTCGTCGGCTCCTCTTAAATATATTAGC-
TGGACTTTGG----------------T-TC---GCG--TG-----------AAGGTGTAATAG---------
---------TTTA-TTCGC----CATTAG-CGCTTG-CCTAA-GAGGTCTGC-TTC-----
CAATCGTCTT------------------CGGAC-AA--------GG------ATTAAT--------
TTCCTTCTTGACTCT---------
TCCCCTAGTAACTGCGAGTGAAGCGGGAAAAGCTCAAATTTAAAATCT-G-GCG-TTC--
TCTGGGCGTCCGAGTTGTAATCTGGAGAAGCGTTTT-CCGCGTTG-
GACCGTGTACAAGTCTCCTGGAACGGAGCGTCATAGAGGGTGAGAATCCCG-
TCCATGACACGG-AC-TACCAGTGC-
TTTGTGATACGCTCTCGAAGAGTCGAGTTGTTTGGGAATGCAGCTCAAAATGGGTGGTAAATTC
CATCTAAAGCTAAATATTGGCGAGAGACCGATAGCGAACAAGTACCGTGAGGGAAAGATGAAA

AGCACTTTGGAAAGAGAGTTAAACAGTACGTGAAATTGTTGAAAGGGAAACGCTTGAAGTCAG
TCGCGTCTTGCGGAACTCAGCCTTAC----TTCGGTCTGGTGTACTTTCCGT-T---
TGACGGGTCAACATCAA-TTTC-GAC-
CGGCAGAAAAAGCTTGAGGAAATGTAGCGTCGTTTCGGTGACGTG-TTATAGT-CCTGTTGC-
GCAT-ATGTCGATTGGGATTGAGGA-CCGCAGCACGCCT------TTAA-TGGCC-------
GGGGG----------------------TT---CG-CCCTACG-T-ATCGTGCTTAGGAT-
GTTGGCATAATGGCTTTAAGCGACCCGTCTTGAAACACGGACCAAGGAGTCTAACATGCTTGCG
AGTGTTCGGGTGGAAAACCCTTGCGCGTAATGAAAGTGAAA-GTTGGGAA-----CCTC-------
-----------CGCAA--GGGGGTGCACCGACGCCCGGCCCTGAAG-----
TTCTCTGACGGTGCTGCGGTAGAGCACGTATGTTGGG----
ACCCGAAAGATGGTGAACTATGCCTGAATAGGGCGAAGCCAGAGGAAACTCTG-
GTGGAGGCTCGTAGCGATTCTGACGTGCAAATCGATCGTCAAATTTGGG------------------
----

>Hydnoporia_tabacina_LWZ_20210924-26a
TCGAGTTTTGA-----A----AGTAAGG------------------------------CTTGATGCTG--
GTACG---------------GAAACG--TACA-TGTG-CTCAGT-----------------------------
--CTT--GCG-----CTCATTCC--A--CTC-----------------AA-CCCC--TGTGCA--
CTTATCG---AAG--------------------
-----------------------------------TTAGTAGT----------------C------GTTCCTC---

-------------------------CTTT-------------------ATTGGAGCCGTCGAGAG--------
---------------------------------TCTCGCAA-----------------------------------
----
------------------------GTAATCATT---
---------------------------------AGGAAAA-------------------------------------
----
------------------GGTCTTTATTGATT-------------TTTCTTAAA-------GGTGAAAGC---
--------------------GGTCTCTT------------TGACT-GGGAGCGGTGAAAGC-TTTGA-----
--CTTTA-TTAT--------------A---AACCTTTAT-TTGTCTTGTAGAAT-G-----T-AAT-GC-T-
CC-TA----GTGGGCGAAAT------
TAAATACAACTTTCAACAACGGATCTCTTGGCTCTCGCATCGATGAAGAACGCAGCGAAATGCG
ATAAGTAATGTGAATTGCAGAATTCAGTGAATCATCGAATCTTTGAACGCACCTTGCGCCCTTTG
G-TATTCCGAAGGGCATGCCTGTTTGAGTGTCATGTTAATCTCAATCCCC--------TCG-
TTTCTTAATTG----------------AAGCTT--------GGAGGTTGGAC-TTGGAGC-T---------
-TGCTGGC-CCCT----------------------CTTGTGGTCGGCTTCTCTTAAATGCATTAGC-
TGGACTTTGG----------------T-TCT--GCG--TG-----------AAGGTGTAATAG--------
---------TTTA-TTCGC----CATTAG-CGCTTG-CCTAA-CGAGTCTGC-TTC-----
TAATCGTCTT------------------CGGAC-AA--------GG------ATTCAG--------
TTCCTTCTTGACTCTTTGACCTCATCCCCTAGTAACTGCGAGTGAAGCGGGAAAAGCTCAAATTT
AAAATCT-G-GCG-TCC--TTTGGGCGTCCGAGTTGTAATCTGGAGAAGCGTTTT-CCGCGTTG-
GACCGTGTACAAGTCTCCTGGAACGGAGCGTCATAGAGGGTGAGAATCCCG-
TCCATGACACGG-AC-TACCAATGC-
TTTGTGATACGCTTTCGAAGAGTCGAGTTGTTTGGGAATGCAGCTCAAAATGGGTGGTAAATTCC
ATCTAAAGCTAAATATTGGCGAGAGACCGATAGCGAACAAGTACCGTGAGGGAAAGATGAAAA
GCACTTTGGAAAGAGAGTTAAACAGTACGTGAAATTGTTGAAAGGGAAACGCTTGAAGTCAGTC
GCGTCTTGCGGAACTCAGCCTTAC----TTCGGTCTGGTGTACTTTCCGT-T---
TGACGGGTCAACATCAA-TTTT-GAT-
CGGTAGAAAAAGCTTGAGGAAAGGTAGCGTTGTTTCGGCGACGTG-TTATAGT-CTTCTTGC-
GTAT-ATATCGGTTGGGATTGAGGA-TCGCAGCACGCCT------TTAA-TGGCC-------
GGGGG----------------------TT---CG-CCCTACG-T-ATCGTGCTTAGGAT-
GTTGGCATAATGGCTTTAAGCGACCCGTCTTGAAACACGGACCAAGGAGTCTAACATGCTTGCG
AGTGTTCGGGTGGAAAACCCTTGCGCGTAATGAAAGTGAAA-GTTGGGAA-----CCTC-------
-----------CGCAA--GGGGGTGCACCGACGCCCGGCCCTGAAG-----
TTCTCTGACGGTGCTGCGGTAGAGCACGTATGTTGGG----
ACCCGAAAGATGGTGAACTATGCCTGAATAGGGCGAAGCCAGAGGAAACTCTG-
GTGGAGGCTCGTAGCGATTCTGACGTGCAAATCGATCGTCAAATTTGGGTTGATGTGTC--AT--
-CGAGGATAAGGTAAGGGAAAAT-AATGATATTACCTTACTATTAGTGTCGTCCAAA-
GCTCGTGCCAGAAGACTCGGTAATGCGAGAGACACAAACGTTAATCGTCTTGATCAGGCGTAA
AGGGTATGTAGGCGGCTTTAAGTACTCAGTTTTTAAGTTTACCGTTAT--------------------
-----------ATAATACTTAG-----------CCCCAGAA--------------------------------
----
GGAAAAGTAGTATATAATGTTAATACTGGGTCAATTAAAGCTAGAATCTAACAGAGGTTA----
AAACAATGCTTAAG--GTAGGAGTACGAACCGCAAATATTAAGTAGAGTGT-
TAAGGGCGAAGGC-

ATTTTTCCAATGAAGATTGACGCTGAGAAACGAAGGTGAGGACAGAAAAAAGGATTAGAGACC
CTTGTATCCCTCACTGTCAACGATGAATGGTAGTTACTAGGGAGAA------------------------
----
ATAACCTAGATACGATGTTAACACGAAAACCATTCCGCCTTGTGAGTACGACTGCAAAGTTGAA
AACAAAAAAATTAGTCGGTTTCGGAG--CAAACGGAGTGAAGC-ATGTTATT-----
GGTACTGGTGAGTTCGAGGCTGGTATCTCCAAGGATGGCCAGACCCGCGAGCACGCCCTTCTC
GCCTTCACCCTCGGTGTGCGTCAGCTCATCGTCGCTGTCAACAAAATGGACACCACCAAGGTTC
ATTCTCATAAATAT--TTATATTTCTTATTTTATACTTAA--CGCTTAT----------
TATCCAGTGGTCCGAGGATCGTTTCACTGAAATTATCAAGGAGACGTCCAACTTCATCAAGAAG
GTCGGTTACAACCCGAAGACCGTTGCCTTCGTCCCGATCTCCGGCTGGCACGGTGACAACATGT
TGGAGGAGTCTGCCAAGTAAGTTGCATTG-CTCTATTTGTCTTG--ATATGGA-----
ATTAAACGCGTTATCGTC-TACAGCATGCCGTGGTACAAGGGCTGGACCAAGGAGACCAAGG--
----------CTGGTGTCGTA-
AAGGGTAAGACCCTCCTCGATGCCATCGACGCTATTGAGCCCCCCATCCGCCCCGAGAACAAG
CCCCTTCGTCTCCCCCTCCAGGATGTGATCTCCGTTGGGTCATTTTCCGCTCCTGTTATCGAGTTC
TTGGAAGAATGGGGATTGGAAAGCCTCGAAGAGAATGCACA---GTCAACAA------
ACCTTACCAAGGTCTTCGTCAACGGTGTTTGGCTTGGTGTGCATCGTGATCCTTTCAACCTGGTC
AACACCATTCGTACGCTCCGACGCAGGG---
AGGATATCAGTTGTGAAGTATCTGTCGTTCGTGACATCCGCGAGAAGGAATTGCGTATTTACACT
GATGCAGGCCGTGTCCTTCGTCCTCTATTCATCGTGGAGGATCATAAGCTGAAATTAAAGCGTC
AGCATCTCCAATGGCTTCACGATAAGCAGCGGATA-------
GAGGAGAAGCCGCTCGAGAAGCCTCCACCGCGAGAAGGCGAGGTCCAGGAAGACTCGGAAG
GCGAGACTATCGCGATA---
GAGGAGAATGGGATGCCGATCATGAAGAACGGTGTCAAGATGAAGAAGAAGAGAGTCCCTTA
CAATTGGGGCTCCCTAATAAAAGATGGTGTGATTGAATATCTTGATGCTGAGGAAGAGGAGACT
GTTATGATCTGCATGACGCCGGATGATCTGGTAGAGTCGCGGTTGATGCGCAAGGGCTTAAACC
CACG---AGCGGACAATGACTTTGACCCTGCCGCTCGGTTGAAGGCTGTCCCAATG------
GCGCACACTTGGACACATTGCGAAATTCACCCTAGCATGATTCTGGGTATCTGCGCTAGTATCAT
T--------
>Hydnoporia_tabacinoides_LWZ_20190814-29b
TCGAGTTTTGA-----A------GCAGG-----------------------------CTTGATGCTG--
GTGCG---------------GAAACG--TACA-TGTG-CTCGGT----------------------------
--CTT--GCG-----CTCATTCC--A--CTC-----------------AA-CCCC--TGTGCA--
CCTATCG--AAG--------------------
-----------------------------------TTAGTAGT----------------C------GTTCCTC---
-------------------------CTTC-------------------ATTGGAGCCGTCGAGAG---------
-----------------------------------TCGCGA------------------------------------
----
------------------------GTAATCATT--
---------------------------------AGGAA---------------------------------------
----
-------------------GTCTTCATTGACT-------------TT--TGAAG-------GGTGGAAGC--
---------------------GTCGCTT------------TGACT-GGGAGCGGTGAAACC-TTTGA----
---CTTTA-CTAT--------------A---AACCTTTAT-TTGTCTTGTAGAAT-G-----T-AAT-GC-

T-CC-TC----GTGGGCGAAAT------
TAAATACAACTTTCAACAACGGATCTCTTGGCTCTCGCATCGATGAAGAACGCAGCGAAATGCG
ATAAGTAATGTGAATTGCAGAATTCAGTGAATCATCGAATCTTTGAACGCACCTTGCGCCCTTTG
G-TATTCCGAAGGGCATGCCTGTTTGAGTGTCATGTTAATCTCAATCTCC--------TTG-
TTTCTTAATTG----------------AGGCTT----AAG-GTGGATTGGAC-TTGGAGG----------
TTTGCTGGC-CCCT------------------------TGTGGTCGGCTCCTCTTAAATACATTAGC-
TGGACTTTGG----------------T-TC---GCG--TG-----------AAGGTGTAATAG---------
---------TTTA-TTCGC----CATTAG-CGCTTG-CCTAA-GAGGTCTGC-TTC-----
CAATCGTCTT------------------CGGAC-AA--------GG------ATTAAT--------
TTCCTTCTTGACTCTTTGACCTCATCCCCTAGTAACTGCGAGTGAAGCGGGAAAAGCTCAAATTT
AAAATCT-G-GCG-TCC--TCTGGGCGTCCGAGTTGTAATCTGGAGAAGCGTTTT-CCGCGTTG-
GACCGTGTACAAGTCTCCTGGAACGGAGCGTCATAGAGGGTGAGAATCCCG-
TCCATGACACGG-AC-TACCAGTGC-
TTTGTGATACGCTCTCGAAGAGTCGAGTTGTTTGGGAATGCAGCTCAAAATGGGTGGTAAATTC
CATCTAAAGCTAAATATTGGCGAGAGACCGATAGCGAACAAGTACCGTGAGGGAAAGATGAAA
AGCACTTTGGAAAGAGAGTTAAACAGTACGTGAAATTGTTGAAAGGGAAACGCTTGAAGTCAG
TCGCGTCTTGCGGAACTCAGCCTTAC----TTCGGTCTGGTGTACTTTCCGT-T---
TGACGGGTCAACATCAA-TTTC-GAC-
CGGCAGAAAAAGCTTGAGGAAATGTAGCGTCGTTTCGGCGACGTG-TTATAGT-CCTGTTGC-
GCAT-ATGTCGATTGGGATTGAGGA-CCGCAGCACGCCT------TTAA-TGGCC-------
GGGGG----------------------TT---CG-CCCTACG-T-ATCGTGCTTAGGAT-
GTTGGCATAATGGCTTTAAGCGACCCGTCTTGAAACACGGACCAAGGAGTCTAACATGCTTGCG
AGTGTTCGGGTGGAAAACCCTTGCGCGTAATGAAAGTGAAA-GTTGGGAA-----CCTC-------
-----------CGCAA--GGGGGTGCACCGACGCCCGGCCCTGAAG-----
TTCTCTGACGGTGCTGCGGTAGAGCACGTATGTTGGG----
ACCCGAAAGATGGTGAACTATGCCTGAATAGGGCGAAGCCAGAGGAAACTCTG-
GTGGAGGCTCGTAGCGATTCTGACGTGCAAATCGATCGTCAAATTTGGGTTAATGTGTC--AT--
-TGAGGATAAGGTAAGGGAAAAT-AATGATACTACCTTACTATTAGTGTCGTCCAAA-
GCTCGTGCCAGAAGACTCGGTAAGGCGAGAGACACAAACGTTAATCATCTTGATCAGGCGTAA
AGGGTTTGTAGGCGGCTTTAAGTACCCTGTTTTTAAGTTTACCATTAT----------------------
----------TTAAAACTTAG-----------CCCGCCAATCC-------------GTAAGG----------
----
ATTGTGCAAAAGTGGTTTATAATGTTAATACAGGGTCAATTAAAGCTAGAATCTAACAGAGGTTA
----AAACAATGCTTAAG--GTAGGAGTACGAACTGCAAATATTAAGTGGAGTGT-
TAAGGGCGAAGGC-
ATTTATCCAATGAAGATTGACGCTGAGAAACGAAGGTGAGGAAAGAAAAAAGGATTAGAGACC
CTTGTATCCCTCACTGTCAACGATGAATGGTAGTTACTAGGGAGAA-----------------------
----
ATGACCTAGAGACAATGTTAACGCGAAAACCATTCCGCCTTGTTAGTACGACTGCAAAGTTGAA
AACAAAAAAATTAGTCGGTTTCGGAG--CAAACGAAGTGAAGC-ATGTTATTTAATT----------
----

>Hymenochaete_asetosa_Dai_10756
GTGAGATTTTA------------GGTGA-------------------------------CTTGTTGCTG--
GCGCCCCTT--------------GGG--TGTA-TGTG-CACAGCC-ATCT-----------------------
---GACTCGCC-----TTCGTTCCTTA-TATC-----------------AA-CCCC--TGTGCA-
CTATATTAGGAT------------------------------------TAGAGAGAAG------------------
-----------------CGCGTCGTTTAAGGGATTAACCTAGTATT----------------T----------
----

-----------------------GTGGGTCCCA--------CCCAC------------------------------
----

--GGAGAAAGGTT-----
------------AATCTGTGATCAG---------CGACGCGTGGTACCTCGACGTC-CTTCT--------
-TAA-CCAC----A--------AA---CTACTA----TAGTCTTGTCAGAATG-----A-TAG-TC-C-
CT-TAGCT-GGACGCTAAAT------
GTAATACAACTTTCAACAACGGATCTCTTGGCTCTCGCATCGATGAAGAACGCAGCGAAATGCG
ATAAGTAATGTGAATTGCAGAATTCAGTGAATCATCGAATCTTTGAACGCACCTTGCGCCCTTTG
G-TATTCCGAAGGGCACGCCTGTTTGAGTGTCATGTTAACCTCAATCTAT--------CAG-
CTTTTTTTG----------------------------TTGC-TTGGATTGGAT-TTGGGAG----------
TCTGCTGGC-CCT-----------------------CTTGGGGTTGGCTCTCCTTGAATGCATGAGT-
GAGCATCTGG----------------C-TC---GCG-TCG-----------CTAGTGTGATAGCA------
---------------TTCATCAT-TAGCTG-CGCTTG-CCTAA-TGAGCTTGC-TTTC----
GAACCGTCTTT-----------------TTGAC-AAC----------------------TTTTTGAC-------
----------------TCCCTTAGTAACTGCGAGTGAACCGGGATGAGCTCAAATTTAAAATCT-G-
GCG-GTTT----CACCGTCCGAGTTGTAATCTGGAGAAGCGTTTT-CCGCGTCG-
GACCGTGTACAAGTCTCTTGGAACAGAGCGTCATAGAGGGTGAGAATCCCG-
TCCATGACACGG-AC-TCCCGATGC-
TTTGTGATACGCTCTCAAAGAGTCGAGTTGTTTGGGAATGCAGCTCAAAATGGGTGGTAAATTCC
ATCTAAAGCTAAATATTGGCGAGAGACCGATAGCGAACAAGTACCGTGAGGGAAAGATGAAAA
GCACTTTGGAAAGAGAGTTAAACAGTACGTGAAATTGTTGAAAGGGAAACGCTTGAAGTCAGTC

GCGTCGTACGGAGCTCAGCCTTGC----TTCTGCTTGGTGTATTCTCCGT-AT--
CGATGGGTCAACGTCAA-TTTC-AGC-
CGGTGGAGAAGGACTTAGGGAATGTAGCGTTGTTTCGGCGACGTG-TTATAGC-CTTAGGTC-
GTAT-GCATCGGCAGGGATTGAGGA-CCGCAGCACGCCC-----TTT-G-TGGCC-------
GGGGG----------------------TT---CG-CCCCACG---TTCGTGCTTAGGAT-
GTTGGCAAAATGGCTTTAAGCGACCCGTCTTGAAACACGGACCAAGGAGTCTAACATGCCTGC
GAGTGTTCGGGTGGAAAACCCTTGCGCGTAATGAAAGTGAAA-GTTGGGAT-----CCTT------
------------CGTAA--GGGGGTGCACCGACGCCCGGCCCTGACG-----
TTCTCTGACGGTGCTGCGGTAGAGCATGTATGTTGGG----
ACCCGAAAGATGGTGAACTATGCCTGAATAGGGTGAAGCCAGAGGAAACTCTG-
GTGGAGGCTCGTAGCGATTCTGACGTGCAAATCGATCGTCAAATTTGGGTTTATGTGTC--AT---
TAAAGATAACGTAAAGGAAAAT-
AATGATATTACTTTACTATTAGTGTCGTCCAAATACTCGTGCCAGAAGACTCGGTAAGGCGAGA
GACGCAAACGTTATTCGTCTTGATCAGGCGTAAAGGGTGTGTAGGCTGCTTTAAGTATTAA--
TATAGTGTTTACTGTTAT-------------------------------AATTTCTACA------------
ATTACATTGTGCTAAGTT---------------------AAATTTAAA----------------------
ATAATTCAATTTAATATAAGCAATTGGGAGAATTATATTATTTAT--
ACTAATCAATTAAAGCTAGAATCTAAAGGAGGTTA----AAACAATACTTAAA--
GTAGGAGTACTAACTGTAAATATTAAGTAGAGTGC-TAACGGCGAAGGC-
ATTTATCCAGTAAAGATTGACGCTGAGAAACGAAAGTGAGGGCAGAAAAAAGGATTAGAGACC
CTTGTATTCCTCACTGTCAACGATGAATGGTAGTTACTAGTTAGAA----------------------
----
ATTAACTAGGGGCGATGTTAACACGAAAACCATTCCGCCTTGTGAGTACAACTGCAAAGTTGAA
AACAAAAAAATTAGTCGGTTTCGGGG--CAAACGAAGTGAAGC-ATGTTATTTAATT----------
----

>Hymenochaete_cyclolamellata_Cui_7393
GCGAGTTTGTG-----------AGGGTC------------------------------TTAAGTGCTG--
GCATCCT-------------TCGGGG--TGCA-TGTG-CACGGCT-CTCC--------------------

----CTCTCGTC-----TTCGTTCCTTC-TATC------------------AA-CCCC--TGTGCA-
CTATATTAGGAT-------------------------------------TAGAGAGAAG------------------
------------------CGCGGCAGTTTAAGGATTAGCCTAGTAGT-----------------T----------
----

-------------------------GTGGGTCCGT--------CCCGT-----------------------------
----

--GGCGAAAGGTT----
-------------AATTT------------------TGACGCGTGGTACCTCGAACTC-CTTAA---------
TTA-CTAC----A--------AA---CCACTATTT-ATGTCTTGTCAGAATG-----T-TCA-GT-C-
CC-TCGTT-GGACGCTAAAT------
GAAATACAACTTTCAACAACGGATCTCTTGGCTCTCGCATCGATGAAGAACGCAGCGAAATGCG
ATAAGTAATGTGAATTGCAGAATTCAGTGAATCATCGAATCTTTGAACGCACCTTGCGCCCTTT-
----TTCGGAAGGGCACGCCTGTTTGAGTGTCATGTTCATCTCAATCCAA--------TAG-
CTTTTGTGG----------------------------CTGC-TTGGATTGGAT-TTGGGAG----------
TCTGCTGGCCCCT------------------------CCCCGGGTCGGCTCTCCTTGAATGCATGAGT-
GAGCATCTGG----------------C-TC---GCGTTTA-----------CTGATGTGATAAC-------
---------------TTCATCAT-TGGTAG-CGCTTG-CCTAACCGGGCTTGC-TTCC----
GAACCGTCTTC------------------TCAAC-GGA-----------------------GACAA----
TACATTGACAATT---------
TCCCTTAGTAACTGCGAGTGAACCGGGAAGAGCTCAAATTTAAAATCT-G-GCG-GTTT----
TGCCGTCCGAGTTGTAATCTGGAGAAGCGTTTT-CCGCGTCG-
GACCGTGTACAAGTCTCTTGGAATAGAGCGTCATAGAGGGTGAGAATCCCG-
TCCATGACACGG-AC-TCCCGGTGC-
TTTGTGATACGCTCTCAAAGAGTCGAGTTGTTTGGGAATGCAGCTCAAAATGGGTGGTAAATTCC
ATCTAAAGCTAAATATTGGCGAGAGACCGATAGCGAACAAGTACCGTGAGGGAAAGATGAAAA
GCACTTTGGAAAGAGAGTTAAACAGTACGTGAAATTGTTGAAAGGGAAACGCTTGAAGTCAGTC
GCGTCGTACGGAGCTCAGCCTTGC----TTCTGCTTGGTGTATTCTCCGT-GT--
CGACGGGTCAACATCAA-TTTT-GAC-
CGGTGGAAAAGGGCTTAGGGAATGTAGCGTTGTTTCGACGACGTG-TTATAGC-CCTAGGTC-
GTAT-GCATCGGTTGGGATTGAGGA-CCGCAGCACGCCC------TT-G-TGGCC-------
GGGGG-----------------------TT---CG-CCCCACG---TTCGTGCTTAGGAT-
GTTGGCAAAATGGCTTTAAGCGACCCGTCTTGAAACACGGACCAAGGAGTCTAACATGCCTGC
GAGTGTTCGGGTGGAAAACCCTTGCGCGTAATGAAAGTGAAA-GTTGGGAT-----CCTT------
------------CGTAA--GGGGGTGCACCGACGCCCGGCCCTGAAG-----
TTCTCTGACGGTGCTGCGGTAGAGCATGTATGTTGGG----
ACCCGAAAGATGGTGAACTATGCCTGAATAGGGTGAAGCCAGAGGAAACTCTG-
GTGGAGGCTCGTAGCGATTCTGACGTGCAAATCGATCGTCAAATTTGGGTTGATGTGTC--AT--
-CAATGATAACGTAAAGAAAAAT-
AATGATATTACTTTACTATTAGTGTCGTCCAAATACTCGTGCCAGAAGACTCGGTAAGGCGAGA
GACGCAAACGTTATTCGTCTTGATCAGGCGTAAAGGGTGTGTAGGCTGCTTTAAGTATATA--
TGTTAGGTTTACCTTTATTTAA-AACATTATTTAAAATATGTTTAAA--

TATTATTTATATTTAATTATTAATTTTTTTATTTATACTA----------------------------------
----
TTCAAACACGTACAATATTGTTGCAAATAAAGCTAATAAATATCAATTAAAGCTAGAATCTAAAA
GAGGTTA----AAACAATGCTTAAG--GTAGGAGTACTAACCGTAAATATTAAGTAGAGTGC-
TAAGGGCGAAGGC-
TTTTATCCACTAAAGATTGACGCTGAGAAACGAAAGTGAGGGCAGAAAAAAGGATTAGAGACC
CTTGTATTCCTCACTGTCAACGATGAATGGTAGTTACTAGTTAGAA-----------------------
----
ATCAACTAGAGACAATGTTAACACGAAAACCATTCCGCCTTGTGAGTACAACTGCAAAGTTGAA
AACAAAAAAATTAGTCGGTTTCGGAG--CAAACGAAGTGAAGC-ATGTTATTTAATG---------
----

>Hymenochaete_huangshanensis_LWZ_20201014-27
ATGAGTTGCGG------------GTGGT------------------------------TGGTTTGCTG--
GCGTCTC-------------TCCAGA--CGCA-TGTG-CACGGCCGCTCC---------------------
----TTCTCATC-----TTCGTTCCTTC-TATC-----------------AA-CCCC--TGTGCA-
CTACAATAGGAT-----------------------------------TAGAGCGAAG----------------
------------------CGCGTCGTTT-AGGGATTAACCTAGTATT----------------C---------
----

------------------------GTGGGTCCAC-------ACCCGC----------------------------
----

--GGCGAAAGGTT----
-------------AATCTGTGATCGGC--------GGCGTCGTGGTACCTCGAAGTC-CTTGT-------
-TTTA-AACC----A--------AA---CCACTATAT-ACGTCTTGTCAGAATG-----T-CAG-CC-C-
CT-TCGTTTGGGCGCTAAAT------
AAAATACAACTTTCAACAACGGATCTCTTGGCTCTCGCATCGATGAAGAACGCAGCGAAATGCG

ATAAGTAATGTGAATTGCAGAATTCAGTGAATCATCGAATCTTTGAACGCACCTTGCGCCCTTTG
G-TATTCCGAAGGGCACGCCTGTTTGAGTGTCATGTTCATCTCAATTCAA--------CAG-
CTTTGCGGC---------------------------TGGC-ATGAGTTGGAT-TTGGGAG----------
TCTGCTGGC-CC------------------------CCCCGGGTCGGCTCTCCTTGAATGCATGAGT-
GAGCATCTGG----------------C-TC---GCG-TCG-----------CTGGTGTGATAGTAT-----
---------------TTCATCAT-TGGTGA-CGCCTG-TCTAA-TGGGCTTGC-TTTC----
GAACCGTCTTC-----------------TCAAC-GGA----------------------GACACATC-
TTAATTGACACATTTGACCTCA-
CCCCTAGTAACTGCGAGTGAAGCGGGATGAGCTCAAATTTAAAATCT-G-GCG-GTTT----
CACCGTCCGAGTTGTAATCTGGAGAAGCGTTTT-CCGCGTCG-
GACCGTGTACAAGTCTCTTGGAATAGAGCGTCATAGAGGGTGAGAATCCCG-
TCCATGACACGG-AC-TCCCGGTGC-
TATGTGATACGCTCTCAAAGAGTCGAGTTGTTTGGGAATGCAGCTCAAAATGGGTGGTAAATTC
CATCTAAAGCTAAATATTGGCGAGAGACCGATAGCGAACAAGTACCGTGAGGGAAAGATGAAA
AGCACTTTGGAAAGAGAGTTAAACAGTACGTGAAATTGTTGAAAGGGAAACGCTTGAAGTCAG
TCGCGTTGTACGGAGCTCAGCCTTGC----TTCTGCTTGGTGTATTCTCTGT-GT--
CAACGGGTCAACATCAA-TTTC-GAT-
CGGTGGAGAAGGGCTTGGGGAATGTGGCGTCGTTTCGGCGACGTG-TTATAGC-CCTAGGTC-
GTAT-GCATCGATTGGGATTGAGGA-CCGCAGCACGCCC------TT-G-TGGCC-------
GGGGGG---------------------TT---CGCCCTTACG-T-TTCGTGCTTAGGAT-
GTTGGCAAAATGGCTTTAAGCGACCCGTCTTGAAACACGGACCAAGGAGTCTAACATGCCTGC
GAGTGTTCGGGTGGAAAACCCTTGCGCGTAATGAAAGTGAAA-GTTGGGAT-----CCCT------
------------CGCAA--GGGGGTGCACCGACGCCCGGCCCTGAAG-----
TTCTCTGACGGTGCTGCGGTAGAGCATGTATGTTGGG----
ACCCGAAAGATGGTGAACTATGCCTGAATAGGGTGAAGCCAGAGGAAACTCTG-
GTGGAGGCTCGTAGCGATTCTGACGTGCAAATCGATCGTCAAATTTGGGTTGATGTGTC--AT--
-CGAAGATAACGTAAAGGAAAAT-
AATGATATTACTTTACTATTAGTGTCGTCCAAATACTCGTGCCAGAAGACTCGGTAAGGCGAGA
GACGCAAACGTTATTCGTCTTGATCAGGCGTAAAGGGTGTGTAGGCTGCTTTAAGTATTTA--
TATTAAGTTTACCGTTATATCTCTAACCAGGTTTGTTCTTGTTTTTATTTTTTATTTTTA----------
--AATTATAATTGCATCAA---
---------------CACCTG-----
TCTAAATTTATAATGTTAATATTTTTCAATTAAAGCTAGAATCTAAAAGAGGTTA----
AAACAATACTTAAG--GTAGGAGTACTAACCGTAAATATTAAGCAGAGTGT-
TAATGGCGAAGGC-
ATTTATCCACTAAAGATTGACGCTGAGAAACGAAAGTGAGGGCAGAAAAAAGGATTAGAGACC
CTTGTATTCCTCACTGTCAACGATGAATGGTAGTTACTAGTTAGAA-----------------------
----
ATTAACTAATGGCGATGTTAACACGAAAACCATTCCGCCTTGTGAGTACAACTGCAAAGTTGAA
AACAAAAAAATTAGTCGGTTTCGGAG--CAAACGAAGTGAAGC-ATGTTATTTAT------------
----

>Hymenochaete_xerantica_LWZ_20190814-13b
GCGAGTTTGCG-----------GGATGC---------------------------------TATTGCTG--
GCGCTCTCT-----------CGGGGG--TGCA-TGTG-CATGGCT-CTCC----------------------
----TTCTCGTC-----TTCGTTCCTTC-TATC-----------------AA-CCCC--TGTGCA-
CTATAATAGGAT------------------------------------TAGAGCGAAG----------------
-------------------CGCGGCAGTTAAGGATTTAGCCTAGTAGT----------------T--------
----

--------------------------GTGGGTCCG---------CCCGC----------------------------
----

---AACGAAAGGTT---
---------------AATCTT-----------------GACGCGTGGTACCTCGAACTC-CTTGA--------
--TA-TTAC----A--------AA---CCACTATTT-ATGTCTTGTCAGAATG-----T-TCA-GT-C-
CC-TCGTT-GGACGCTAAAT------
AAAATACAACTTTCAACAACGGATCTCTTGGCTCTCGCATCGATGAAGAACGCAGCGAAATGCG
ATAAGTAATGTGAATTGCAGAATTCAGTGAATCATCGAATCTTTGAACGCACCTTGCGCCCTTT-
----TTCGGAAGGGCACGCCTGTTTGAGTGTCATGTTCATCTCAATCCAA--------CAG-
CTTTGCGGC----------------------------TGAC-TTGGATTGGAT-TTGGGAG----------
TCTGCTGGC-CCT------------------------TCTTGGGTCGGCTCTCCTTGAATACATGAGT-
GAGCATCTGG----------------C-TT---GCG-TTG-----------CTAATGTGATAAC--------
---------------TTCATCAT-TGGCGG-CGCTTG-CCTAA-CGGGCTTGC-TTCC----
GAACCGTCTTC-----------------TCAAC-GGA----------------------GACAAATC-
ATCATTGACAATTTTGACCTCA-
CCCCTAGTAACTGCGAGTGAACCGGGATGAGCTCAAATTTAAAATCT-G-GCG-GTTC----
TGCCGTCCGAGTTGTAATCTGGAGAAGCGTTTT-CCGCGTCG-
GACCGTGTACAAGTCTCTTGGAATAGAGCGTCATAGAGGGTGAGAATCCCG-
TCCATGACACGG-AC-TCCCGGTGC-
TTTGTGATACGCTCTCAAAGAGTCGAGTTGTTTGGGAATGCAGCTCAAAATGGGTGGTAAATTCC
ATCTAAAGCTAAATATTGGCGAGAGACCGATAGCGAACAAGTACCGTGAGGGAAAGATGAAAA

GCACTTTGGAAAGAGAGTTAAACAGTACGTGAAATTGTTGAAAGGGAAACGCTTGAAGTCAGTC
GCGTCGTACGGAGCTCAGCCTTGC----TTCTGCTTGGTGTATTCTCCGT-GT--
CGACGGGTCAACGTCAA-TTTT-GAC-
CGGTGGAAAAGGGCCTAGGGAATGTGGCGTTGTTTCGGCGACGTG-TTATAGC-CTTAGGTC-
GTAT-GCATCGGTTGGGATTGAGGA-CCGCAGCACGCCC------TT-G-TGGCC-------
GAGGG----------------------TT---CG-CCCTACG---TTCGTGCTTAGGAT-
GTTGGCAAAATGGCTTTAAGCGACCCGTCTTGAAACACGGACCAAGGAGTCTAACATGCCTGC
GAGTGTTCGGGTGGAAAACCCTTGCGCATAATGAAAGTGAAA-GTTGGGAT-----CCTT------
------------CGTAA--GGGGGTGCACCGACGCCCGGCCCTGAAG-----
TTCTCTGACGGTGCTGCGGTAGAGCATGTATGTTGGG----
ACCCGAAAGATGGTGAACTATGCCTGAATAGGGTGAAGCCAGAGGAAACTCTG-
GTGGAGGCTCGTAGCGATTCTGACGTGCAAATCGATCGTCAAATTTGGGTTGGTGTGTC--AT--
-CAATGATAATGTAAAGAAAAAT-
AATGATATTACTTTACTATTAGTGTCGTCCAAATACTCGTGCCAGAAGACTCGGTAAGGCGAGA
GACGCAAACGTTATTCGTCTTGATCAGGCGTAAAGGGTGTGTAGGCTGCTTTAAGAATATT--
TGTTAGGTTTACCTTTATCTAT-AAATTAATTTAAATTATGTTTTAA--
TATTATTTATAATATATTATTTATAATATGTTATTTATAATT-------------------------------
----
TTTTTTTACACGTACAATTTTTAAGTAGATTCTGCTAGCAAATATCAATTAAAGCTAGAATCTAAA
GGAGGTTA----AAACAATACTTAAG--GTAGGAGTACTAACCGTAAATATTAAGTAGAGTGC-
TAACGGCGAAGGC-
ATTTATCCACTAAAGATTGACGCTGAGAAACGAAAGTGAGGGCAGAAAAAAGGATTAGAGACC
CTTGTATTCCTCACTGTCAACGATGAATGGTAGTTACTAGTTAGAA-----------------------
----
ATTAACTAGAGACGATGTTAACACGAAAACCATTCCGCCTTGTGAGTACAACTGCAAAGTTGAA
AACAAAAAAATTAGTCGGTTTCGGAG--CAAACGAAGTGAAGC-
ATGTTATTTAATTGGCATAGGTGAATTCGAGGCTGGTATCTCCAAGGATGGCCAGACCCGCGAG
CACGCGCTCCTTGCCTTCACCCTCGGTGTGCGTCAGCTCATCGTCGCCGTCAACAAGATGGACA
CCACGAAGGTTGGTTAATGTTCTTT---TGTTAGTCAGAACCTCGTGCTGAC--CTGCTCT------
----
ATCACAGTGGTCTGAGGATCGTTTCAACGAAATCGTGAAGGAGACTTCCAACTTCATCAAGAAG
GTCGGCTACAACCCGAAGGCTGTCCCCTTCATCCCGATTTCCGGCTGGAACGGTGACAACATGC
TGGAGGAGACGCCCAAGTAAGTCACATGC-TCTGTTC------TTAACGCAAC-----
ATAATCTGATCGCTCGCC-ATCAGTGCTCCGTGGTGGAAGGGTTGGACTAAGGAGACGAAGG--
----------CCGGTGTCGTC-
AAGGGCAAGACCCTCCTTGACGCCATCGACGCCATCGAGCCTCCTGTCCGCCCGGAGAACAAG
CCCCTCCGTCTCCCGCTCCAGGACGTCATCTCCGTCGGATCTTTGTCTGAGCCGGTCATCGAGTT
CTTGGAGGAGTGGGGTCTGGAGAGTTTGGAAGAAAATGCTCA---TGCGACTA------
ATCTGACCAAGGTGTTTGTAAACGGCGTCTGGGTAGGCGTTCACCGCGACTCAGCCACCCTCAT
AAACACGTTCCGAGGATTGCGACGGAGAA---
ACGAAATCGAGGACGAGGTATCATTGGCTCGAGACATCCGAGAGAAAGAAGTGCGGATGTACA
CGGATGCTGGACGTGTTCTTCGTCCGCTATTTGTCGTCGATAAGCACCGGTTGCTCTTGAAACGT
AGGCATCTGAACTGGCTCTCGATGGGA---AGGGTG-------GAGGTACGCCCGTGGGTA-----
-CCTGCCCATTTGAAG---------------

TCCCCTGGAGCAACCGAGGAGGAACTTGATGAAGAGGACAGACCAATGATGAAGGATGGTATT
CGGATAAAGAAGAAGCGGAAAGAGTACAAATGGGGGAACTTGCTCCATGACGGCGTCGTTGA
ATACATTGATGCGGAGGAAGAGGAGTCGCTCATGATTTGCATGACTCCAGATGATCTCACGGAA
TCTCGTCTCATGAAACAGGGTCTCAATCCACG---AGCAGGTGTGG---
CTGATCCGGCAGCGCGACTCAAAGCGATATCCATG------
GCTCACACTTGGACGCATTGCGAGATTCACCCGAGTATGATTTTGGGTATCTGTGCGAGCATCAT
TCCATTTCC
>Inonotus_griseus_Dai_13436
TCGAGTTTTGA-----A-A--GCGGGAG------------------------------GCCTGTGCTG--
GCGCG---------------GAAACG--CGCA-TGTG-CACGGC----------------------------
---TTTTCGTG-----CTCAAATCC-A--ATC-----------------AA-CCCC--TGTGCA-
CCTAAAGA---GTCGGGAG-----------------
--------------------------------------TCAGTAGT----------------C------TCTGT--
---------------------------AATCGAATTGCT-------------------------------------
----

--TGTAGTGAGTAA--------
------------------------------CTCGAG-GGAGGAGGCGAAAGCTTTTGG------CTAAT--
TTAT----AA-------AC---ACCTTTTGT-TTGTAATGTGAAT--G-----T-AAT-GC-T-CC-TC--
--GTGGGCGATAATG-----
AAATACAACTTTCAACAACGGATCTCTTGGCTCTCGCATCGATGAAGAACGCAGCGAAATGCGA
TAAGTAATGTGAATTGCAGAATTCAGTGAATCATCGAATCTTTGAACGCACCTTGCGCCCTTTGG
-TATTCCGAAGGGCATGCCTGTTTGAGTGTCATGTTAATCTCAAATCAC--------TTG-
TCTATTCTTAAC---------GGAATGGGGCCT--------GTGATTTGGAC-TTGGAGG-T-C-----
-TTTGCTGGC-GCGAGGGGGGGTTTACTTCTCCT--
TGCTTTGTCGGCTCCTCTCAAATCTATTAGC-TGGACTTTGG----------------T-TC---GCG-
TTA-----------GCGGTGTAATAATAT---------------TCGT-TTCACTA-----AGA-
CGCTTG-CCTAG-ATGGTCTGC-TTC-----CAATCGTCCATAGCA-------------TGGAC-AA--
------GGACCC--GTCAGT-GGC----C-
TTTCTTGACACCTTTGACCTCATCCCCTAGTAACTGCGAGTGAAGCGGGAAAAGCTCAAATTTAA
AATCT-G-GCG-GTCT-TCTGGCCGTCCGAGTTGTAGTCTGGAGAAGTGTTAT-CCGCGTCG-
GACCGTGTACAAGTCTCCTGGAATGGAGCGTCATAGAGGGTGAGAATCCCG-
TCCATGACACGG-AC-GACCGATGC-
TATGTGAGGCACTCTCAAAGAGTCGAGTTGTTTGGGAATGCAGCTCAAAATGGGTGGTAAATTC
CATCTAAAGCTAAATATTGGCGAGAGACCGATAGCGAACAAGTACCGTGAGGGAAAGATGAAA
AGCACTTTGGAAAGAGAGTTAAACAGTACGTGAAATTGTTGAAAGGGAAACGCTTGAAGTCAG
TCGCGTTCCGTGGAACTCAGCCTTGC----TTCGGCCTGGTGTACTTTCTGC-G---
GGACGGGTCAACATCAG-TTTT-GGC-
CGGTGGAAAAAGGCAGAGGGAATGTAGCGCCGTTTCGGCGGCGTG-TTATAGC-CCTTTGTC-
GAAT-ACACTGGCTGGGACTGAGGA-CTGCAGCACGCCC------TT-G-TGGCC-------
GGGGG----------------------TT---CG-CCCCACG-T-AACGTGCTTAGGAT-

GTTGGCATAATGGCTTTAAGCGACCCGTCTTGAAACACGGACCAAGGAGTCTAACATGCCTGCG
AGTGTTCGGGTGGAAAACCCTTGCGCGTAATGAAAGTGAAA-GTTGGGAA-----CCTC-------
-----------CGCAA--GGGGGTGCACCGACGCCCGGCCCTGAAG-----
TTCTCTGACGGTGCTGCGGTAGAGCAAGTATGTTGGG----
ACCCGAAAGATGGTGAACTATGCCTGAATAGGGCGAAGCCAGAGGAAACTCTG-
GTGGAGGCTCGTAGCGATTCTGACGTGCAAATCGATCGTCAAATTTGGG-----------------
---------GTAAAGGAAAAT-
AATGATATTACTTTACTATTAGTGTCGTCCAAGTGCTCGTGCCAGAAGACTCGGTAAGGCGAGA
GACGCAAACGTTATTCGTCTTGATCAGGCGTAAAGGGTTTGTAGGCTGCTTTAAGTACCTAA-
ATTTAAGTTTACTATTAT-------------------------------AAAATTACTG--------------
----

TTGTAGTATTATAATATTTAAAAAAGGGTCAATTAAAGCTAGAATCTAAAAGAGGTTA----
AAACAATGCTTGATATACAGGTGTACTAACCGAAAACAACAAGTAGAGTGT-
TAAGGGCGAAGGC-
TTTTATCCAATAAAGATTGACGCTGAGAAACGAAGGTGAGGACAGAAAAAAGGATTAGAGACC
CTTGTATCCCTCACTGTCAACGATGAATGGTAGTTACTAGGTAGAAG---------------------
----
ATGACCTAGAGACGATGTTAACACGAAAACCATTCCGCCTTGTGAGTACGACTGCAAAGTTGAA
AACAAAAAAATTAGTCGGTTTCGGAG--CAAACGAAGTGAAGC-ATGTTATTTA---
GGTACTGGTGAATTCGAGGCTGGTATTTCAAAGGATGGCCAAACGCGCGAGCATGCTCTCCTTG
CCTACACTCTTGGTGTGCGTCAGCTTATCGTGGCTATCAACAAGATGGACACCACGAAGGTCAG
CTTTGATTTGCCATGCAATCGTTACATCCATGTTGATTT--CCGCCTT------
TTCCCATTCAGTGGTCTGAGGATCGATTCAATGAAATCGTTAAGGAAACGTCCAACTTTGTCAAG
AAGGTCGGTTACAACCCGAAGGCCGTACCCTTCGTCCCAATTTCTGGCTGGCACGGTGACAACA
TGCTTGAGGAATCCTCAAAGTATTTTATCTCC-TAAAATATGAAAGGGCATCAGCT-----
GACTAGTGGTATCAATTT-CACAGTATGACGTGGTACAAGGGCTGGACCAAGGAAACTAAGG--
----------GTGGTGTCGTC-
AAGGGCAAGACACTCCTCGATGCCATCGACGCTATCGAACCTCCTGTCCGTCCTGAGAACAAG
CCCTTGCGTCTTCCGCTCCAGGACGTGATTTCTGTTGGTTCACTATCGGCGCCGGTACTTGAATT
CTTAGAAGAGTGGGGTTTGGAGAGTTTAGAGGAAAACACCCA---AACTCCAA------
ATTTAACCAAAGTGTTCGTCAACGGTGTTTGGATTGGCGTTCATCGCGACCCTTCCACATTAGTG
AACATGCTTCGTACCCTCCGAAGAAAGA---
CTGACCTTGGCGAGGAAGTCTCCATCGTGCGAGATATTCGTGAGAGGGAAGTCCGCATCTATAC
GGATGCCGGTCGTGTGCTTCGGCCACTTTTCATCGTCGAGAACCACCGCTTGAAGCTGAGGCGT
CAGCATTTGCTGTGGCTGCAGCAGAAATGGAGGATC-------
GAGGAAAGGCCCTTTGAAAGACCGCC---------------
GGAAGCTAATATCTCAGACGGCGAAACAGTAGCTCGT---
GAAGAAGACGGGAAACCTATAATTCGGGACGGTGTTCGGATGAAGAAAAAGCGGAGGGCGTT
CAAATGGCAGTCTTTCCTCGACGATGGTGTCGTTGAATATCTTGACGCCGAGGAGGAGGAGACA
GTCATGATCTGCATGACACCAGACGACTTGGTCGAGTCTCGATTAATGCTCAGGGGTCTCAATC
CTCG---AATAGAGGGAGAAATTGACCCTGCTGCGCGTCTTAAGGCGATCCCGACG------
GCACATACATGGACGCATTGCGAGATCCATCCGAGCATGATTTTGGGTATCTGTGCCAGTATTAT
TCCATTCCC

>Inonotus_henanensis_Dai_13157
TCGAGTTTTGA-----A----GCGAGAG------------------------------GTCTGTGCTG--
GCGCG---------------GAAACG--CGTA-TGTG-CACGGC----------------------------
---TTTTCGTG-----CTCAAATCC-A-TTTC-----------------AA-CCCC--TGTGCA-
CCTTTAAG---AGTCGAAG------------------
-------------------------------------TCGGTAGT----------------C------TCTGT---
--------------------------AATTGAACGGGGC------------------------------------
----

---TTTGGAGTAT--

--GCTTACC-------
GGCAGAAAAGAGGCCTTT------------------------------ACTAGAGA-
AAAGGAGGCGAAGGCTTTTGG------CTAATA-TTAC----AAA------AC---ACACTTTGA-
TTGTTATGTGAAT--G-----T-AAT-GC-T-CG-TT----GTGAGCGATAATG-----
AAATACAACTTTCAACAACGGATCTCTTGGCTCTCGCATCGATGAAGAACGCAGCGAAATGCGA
TAAGTAATGTGAATTGCAGAATTCAGTGAATCATCGAATCTTTGAACGCACCTTGCGCCCCTTGG
-TATTCCGAGGGGCATGCCTGTTTGAGTGTCATGTTAATCTCAAATCAC--------TTG-
TTCTTTTCTTTAC--------GGGAAGGAAGTG--------GTGGTTTGGAC-TTGGAGG-T-T-----
-TTTGCTGGC-CC------------------------CATTTGGTCGGCTCCTCTCAAATACATTAGC-
TGGACTTTGG----------------T-CT---GCGTTTT-----------ACGGTGTGATAATTA------
---------TCAC-TTCACCA-----GAG-CGCTTG-CCTAA-TGGGTCTGC-TTC-----
TAATCGTCCTTAAGT-------------TGGAC-AA--------GGATCCC-GTGAGT-GGG----C-
CTTCTTGACACCTTTGACCTCATCCCCTAGTAACTGCGAGTGAAGCGGGAAAAGCTCAAATTTAA
AATCT-G-GCG-GTCT-TCTGGCCGTCCGAGTTGTAGTCTGGAGAAGTGTTAT-CCGCGTCG-
GACCGTGTACAAGTCTCCTGGAATGGAGCGTCATAGAGGGTGAGAATCCCG-
TCCATGACACGG-AC-TACCGATGC-
TATGTGAGGCACTCTCAAAGAGTCGAGTTGTTTGGGAATGCAGCTCAAAATGGGTGGTAAATTC
CATCTAAAGCTAAATATTGGCGAGAGACCGATAGCGAACAAGTACCGTGAGGGAAAGATGAAA
AGCACTTTGGAAAGAGAGTTAAACAGTACGTGAAATTGTTGAAAGGGAAACGCTTGAAGTCAG
TCGCGTCCTGTGGAACTCAGCCTTGC----TTCGGCCTGGTGTACTTTCTGC-A---
GGACGGGTCAACATCAG-TTTT-GGC-
CGGTGGAAAAAGGCGAGGGGAATGTAGCGCCGTTTCGGCGGCGTG-TTATAGC-CCGTTGTC-
AAAT-ACACTGGCTGGGACTGAGGA-CTGCAGCACGCCC------TT-G-TGGCC-------
GGGGG----------------------TT---CG-CCCCACG-T-AACGTGCTTAGGAT-
GTTGGCATAATGGCTTTAAGCGACCCGTCTTGAAACACGGACCAAGGAGTCTAACATGCCTGCA
AGTGTTCGGGTGGAAAACCCTTGCGCGTAATGAAAGTGAAA-GTTGGGAA-----CCTC--------
-----------CGCAA--GGGGGTGCACCGACGCCCGGCCCTGAAG-----
TTCTCTGACGGTGCTGCGGTAGAGCAAGTATGTTGGG----
ACCCGAAAGATGGTGAACTATGCCTGAATAGGGCGAAGCCAGAGGAAACTCTG-
GTGGAGGCTCGTAGCGATTCTGACGTGCAAATCGATCGTCAAATTTGGG------------------
----

CAGACCCGCGAGCATGCTCTCCTTGCTTTCACTCTTGGTGTGCGTCAGCTTATCGTGGCTGTCAA
CAAGATGGACACTACGAAGGTCAGCTTCGATCTGCTCT-GCAACATTACACCTTTATTGATTT--
TCGCCTT------
TTCCCGTTCAGTGGTCTGAGGACCGATTCAATGAAATTGTTAAGGAAACGTCCAACTTCATCAA
GAAGGTTGGTTACAACCCGAAGGCCGTACCTTTTGTCCCGATTTCTGGCTGGCACGGCGACAAC
ATGCTTGAGGAATCGTCAAAGTACGTTATCTCT-TCTAAG--AAAAGAAAGGGTATC-----
AGCTGAACAATATCAATT-TGCAGCATGTCCTGGTACAAGGGCTGGACAAAGGATACTAAGG--
----------GCGGCGTTCTC-AAGGGCAAGACTCTCCTCGATGCTATC---------------------
----
ATCTCCGTTGGTTCGCTATCGGCCCCGGTACTTGAATTCCTAGAAGAGTGGGGTTTGGAAAGTCT
GGAGGAGAACACCCA---AACGCCGA------
ACCTGACCAAAGTGTTCGTTAACGGTGTTTGGATTGGTGTCCATCGCGACCCCTCTACATTAGTG
AACATGCTTCGCACTCTCCGAAGAAAGA---
CCGATCTTGGTGAGGAAGTCTCCATTGTACGAGATATCCGAGAGAGAGAAGTCCGGATCTATAC
GGATGCTGGTCGGGTGCTTCGACCACTTTTCATTGTTGAGAACCACCACTTGAAGCTGAGGCGC
CAGCATTTGTTGTGGCTACAACAAAAATGGAGAATT-------
GAGGAAAGGCCCGTCGAAAGACCGCC---------------
GGATGTTCAGTTCTCTGATGGCGAAACGGTAGCTCTT---
GAAGAGGACGGACAGCCCATAATTCGGGAAGGGGTTCGGATGAAGAAGAAGCGGAAGCCTTT
CAAATGGCAGTCTTTCCTCGACGATGGTGTTGTCGAATACCTCGATGCGGAAGAGGAGGAGAC
AGTAATGATCTGCATGACACCAGATGACTTAGTCGAGTCTCGATTAATGCTCAGAGGTCTGAAT
CCCCG---AGTGGAT---

>Inonotus_obliquus_Dai_10715
GTTTATTTTGA-----A-A--TCGAGGG-------------------------------GCCTGTGCTG--
GCACG---------------GAAACGTTTGCA-TGTG-CACGGC-----------------------------
---CTTTCGTG-----CTCAAATCCAA--CTCTC--------------AAA-CCCC--TGTGCA-
CCTATACA--AGTTGAAGGT----------------
-------------------------------------CTTAGTAGT----------------T------TCTGT---
---------------------------AATCGAACGGCAAG------------------------------------
----

-----------------------TCAAGTACG---------------------------TCGAGT-
AATCAAGTAC----------------------------------GAGGGTTT--CGGCCCTTGGAAA-----
--------------------------------------G---
---TGTGAAA-----

--GATGGAAAGGCAAGCTT-------------------------------CAGAGACA-
AGGAGACGAAAAGGCTTTTGG-------CTTCA-TTAC----AAA------CA---CCAATATAA-
TTGTTATGTGAAT--G-----A-AAT-GC-T-CC-TT----GTGGGCGATAA------
TAAATACAACTTTCAACAACGGATCTCTAGGCTCTCGCATCGATGAAGAACGCAGCGAAATGCG
ATAAGTAATGTGAATTGCAGAATTCAGTGAATCATCGAATCTTTGAACGCACCTTGCGCCCCTTG
G-TATTCCGAGGGGCATGCCTGTTTGAGTGTCATGTTAATCTCAAATCGC--------TCG-
TCTATTCTTAA----------TTGAAGTGGCTT--------TCGATTTGGAC-TTGGAGG-T--------
TTTGCTGGC-CCGGGCGA---CTTTGGTTGCCCT--
TGGTTTGTCGGCTCCTCTCAAATACATTAGC-TGGACTTTGG----------------T-TC---GCG-
TTT-----------ACGGTGTAATAATGT---------------AATG-TTCACTA-----AGA-
CGCTTG-CCTAA-CAAGTCTGC-TTC-----TAATCGTCCTTAAGT-------------TGGAC-AA--
------GGATCC--CTTCGTTGGG----C-CTTCTTGACACCTTTGACCT----
CCCTAGTAACTGCGAGTGAAGCGGGATGAGCTCAAATTTAAAATCT-G-GCG-GTCT-
TCTGGCCGTCCGAGTTGTAGTTTCGAGAAGTGTTAT-CCGCGTCG-
GACCGTGTACAAGTCTCCTGGAATGGAGCGTCATAGAGGGTGAGAATCCCG-
TCCATGACACGG-AC-GCCCGATGC-
TATGTGAGGCACTCTCAAAGAGTCGAGTTGTTTGGGAATGCAGCTCAAAATGGGTGGTAAATTC
CATCTAAAGCTAAATATTGGCGAGAGACCGATAGCGAACAAGTACCGTGAGGGAAAGATGAAA
AGCACTTTGGAAAGAGAGTTAAACAGTACGTGAAATTGTTGAAAGGGAAACGCTTGAAGTCAG
TCGCGTCCCTTAGAACTCAGCCTTGC----TTCTGCCTGGTGTACTTTCTAA-TG--
GGATGGGTCAACATCAG-TTTT-GGG-
CGGTGGAAAAAGGCAGAGGGAATGTAGCGCTGCTTCGGCGGCGTG-TTATAGC-CCTTTGTC-
TAAT-ACACTGGCTGGGACTGAGGA-CTGCAGCACGCCC------TT-G-TGGCC-------
AGGGG----------------------TT---CG-CCCCATG-T-AACGTGCTTAGGAT-
GTTGGCATAATGGCTTTAAGCGACCCGTCTTGAAACACGGACCAAGGAGTCTAACATGCTTGCA
AGTGTTCGGGTGGAAAACCCTTGCGCGTAATGAAAGTGAAA-GTTGGGAA-----CCTC-------
-----------CGCGA--GGGGGTGCACCGACGCCCGGCCCTGAAG-----
TTCTCTGACGGTGCTGCGGTAGAGCAGGTATGTTGGG----
ACCCGAAAGATGGTGAACTATGCCTGAATAGGGCGAAGCCAGAGGAAACTCTG-
GTGGAGGCTCGTAGCGATTCTGACGTGCAAATCGATCGTCAAATTTGGG------------------
----

>Inonotus_rickii_Dai_12996
TCGTGTTTTGA-----A-A--CAGAAGG------------------------------CTTGTTGGTG--
GTACA---------------GAAACG--TACA-TGTG-CACGGC----------------------------
---TTT-CGTG-----CTCAATCC--A--CTC----------------AAA-CCCC--TGTCCA-
CTTTGAAC---GGTTGAAG------------------
-------------------------------------TTAGTAGT----------------CT----
AGTAACAC----------------------------TCTCGACTCCGA--------------------------
----

---------------------------------CTTAGTACT--------------------------GTGAGT-
ATACAAGTAG---------------------------------GAGGGCTT-----------------------
----
-------------------------------------GCTTTTGCG------------GCCTTTCGAA-------
CGTTAAGAGAA---------------------------------------GAGAGG-
GTGAAGGGCGAACGC-TTTGAC--TAGTTTATG-TTAT-------------AA---
ACCCTTTGTATTGTTATGTGAAT--G-----T-AAT-GC-T-CC-TT----GTGGGCGATAATG-----
AAGTACAACTTTCAACAACGGATCTCTTGGCTCTCGCATCGATGAAGAACGCAGCGAAATGCGA
TAAGTAATGTGAATTGCAGAATTCAGTGAATCATCGAATCTTTGAACGCACCTTGCGCCCCTTGG
-TATTCCGAGGGGCATGCCTGTTTGAGTGTCGTGTTAATCTCAAATCAC--------TTG-
TCTTTTT--------------------TGACTT--------GCGATTTGGAC-TTGGAGG-T-T------
TATGCTGGC-ACGGGCGACTTTGTTGTTGCCCTT--
TGGTTTGTCGGCTCCTCTTAAATGCATTAGC-TGGACTTTGG----------------T-TC---GCG-
TTT-----------GCGGTGTAATAGTGTGTTAT----------AATACTTCACCA-----GAG-
CGCTTG-CCTAA-CGGGTCTGC--
-----------------------T--
GAAGCGGGAAAAGCTCAAATTTAAAATCT-G-GCG-GTCT-
TCTGGCCGTCCGAGTTGTAGTCTGGAGAAGTGTTAT-CCGCGTCG-
GACCGTGTACAAGTCTCCTGGAATGGAGCGTCATAGAGGGTGAGAATCCCG-
TCCATGATACGG-AC-GCCCGATGC-
TTTGTGAGGCACTCTCGAAGAGTCGAGTTGTTTGGGAATGCAGCTCAAAATGGGTGGTAAATTC
CATCTAAAGCTAAATATTGGCGAGAGACCGATAGCGAACAAGTACCGTGAGGGAAAGATGAAA
AGCACTTTGGAAAGAGAGTTAAACAGTACGTGAAATTGTTGAAAGGGAAACGCTTGAAGTCAG

TCGCGTCCCGTAGAACTCAGCCTGGC----TTTGGTCTGGTGTATTTTCTAT-GT--
GGACGGGTCAACATCAG-TTTT-GGC-
CGGTGGAAAAAGGCAGAGGGAATGTAGCGCCGCTTCGGCGGCGTG-TTATAGC-CCTTTGTC-
AAAT-ACACTGGCTGGGACTGAGGA-CCGCAGCACGCCC------TT-G-TGGCC-------
GGGGG----------------------TT---CG-CCCCACG-T-ACCGTGCTTAGGAT-
GTTGGCATAATGGCTTTAAGCGACCCGTCTTGAAACACGGACCAAGGAGTCCAACATGCTTGCG
AGTGTTCGGGTGGAAAACCCTTGCGCGTAATGAAAGTGAAA-GTTGGGAA-----CCTC-------
-----------CGCAA--GGGGGTGCACCGACGCCCGGCCCTGACG-----
TTCTCTGACGGTGCTGCGGTAGAGCAAGTATGCTGGG----
ACCCGAAAGATGGTGAACTATGCCTGAATAGGGCGAAGCCAGAGGAAACTCTG-
GTGGAGGCTCGTAGCGATTCTGACGTGCAAATCGATCGTCAAATTTGGG------------------
----

>Pallidohirschioporus_biformis_Dai_14531
-CGAGTTTTGA-----A------GTGGG------------------------------CTTGATGCTG--
GCTTG---------------TAACAG--AGCACTGTG-CTCAGC-----------------------------
--CCC--------GCTCCAATCC--A--TTC-----------------AA-CCCC--TGTGCA-
CTATTCGG--AGT-------------------
------------------------------------GTTGCAAGCTGAGA----------C--------------
-------------------------AATGTGGGGA---

--------------GTGGTCCCGGTT------------GTATTTCTAA-------TGCGACTTGGGG----
----------------CTTACTT-----------TCAAAC-GGTCAAGGCTTGTCC-TCCGG------
TTTATA-TTAC-------------AA---ACACTTTTA-TTGTCTTGTCGAAT-G-----T-ATTAGCCT-
CT-CG----TTAGGCGAAACTT-----
AAATACAACTTTCAACAACGGATCTCTTGGCTCTCGCATCGATGAAGAACGCAGCGAAATGCGA
TAAGTAATGTGAATTGCAGAATTCAGTGAATCATCGAATCTTTGAACGCACCTTGCGCTCCTTGG
CTATTCCGAGGAGCATGCCTGTTTGAGTGTCATGTTAATATCAACTCTG--------ATG-
GTTTTTGTTAA-------------------------CCATTGGATGTTGGAC-TTGGAGG-T-T------
CGTGCTGGC--------------------------TGCAAAGTCGGCTCCTCTTGAATGCATTAGC-
TTGGACCTGT-------------GCGC-GT---TTG-CTA-----------GCGGTGTAATACATTTTA--
---------------TTCACCAC---GGGC-CGTGTCACTATT-AGGGTCTGC-TTC-----
TAATCGTCCTT--
----------------CCTAGTAACTGCGAGTGAAGCGGGAAAAGCTCAAATTTAAAATCT-G-
GCG-GCTT----TGTCGTCCGAGTTGTAATCTGGAGAAGCGTTTT-CCGTGTCG-
GACCGTGCACAAGTCTCTTGGAACAGAGCGTCATAGAGGGTGAGAATCCCG-
TCTTTGGCACGG-AC-AGCCGATAC-
TTTGTGATACGTTTTCAAAGAGTCGAGTTGTTTGGGAATGCAGCTCAAAATGGGTGGTAAATTCC
ATCTAAAGCTAAATATAGGCGAGAGACCGATAGCGAACAAGTACCGTGAGGGAAAGATGAAA
AGCACTTTGGAAAGAGAGTTAAATAGTACGTGAAATTGTTGAAAGGGAAACGCTTGAAGTCAGT
CGCGTCTGCCGGGACTCAGCCTTAC----TTTTGTATGGTGTATTTCCTGGTT---
TGACGGGTCAACATCGA-TTTT-GAC-CGATGGATAAAGGAGAAGGGAATGTGGCACCTTC----
GGGTGTG-TTATAGC-CCTCT-TT-GTAT-ACATCGGTTGGGATCGAGGA-CTGCAGCACGCCC--
----TT-G-TGGCC-------GGGGG----------------------TT---CG-CCCTACG-T-
ATCGTGCTTAGGAT-
GTTGACATAATGGCTTTAAGCGACCCGTCTTGAAACACGGACCAAGGAGTCTAACATGCTCGCG
AGTGTTAGGGTGGAAAACCCTTGCGCGTAATGAAAGTGAAG-GTTGGGA------CCTC-------
-----------TGCGA--GGAGG-GCACCGACGCCCGGCCCTGAAG-----
TTCTCTGACGGTGCTGCGGTAGAGCGCGTATGTTGGG----
ACCCGAAAGATGGTGAACTATGCCTGAATAGGGCGAAGCCAGAGGAAACTCTG-
GTGGAGGCTCGTAGCGATTCTGACGTGCAAATCGATCGTCAAATTTGGGTCGATGTGTC--AT--
-TGGGGATAACGTAAGGGAAAAT-
AATGATATTACCTTACTATTAGTGTCGTCCAAGTACTCGTGCCAGAAGACTCGGTAAGGCGAGA
GACGCAAACGTTAGTCGTCTTGATCAGGCGTAAAGGGTTTGTAGGCGGCTTTAAGTATCTAA-
TTTTTAGTTTACTATTTT-------------------------------AATATAGGAA------------
AAATTT--
TTATTTTTTATTTTCATAAAAATTATAATTTAATATTAAAAAAATAAAGAAAGGATCAATTAAAGCT
AGAATCTAAAAGAGGTTA----AAACAATACTTCAG--
AAAGGAGTGCTAACCGTAGATAAGGAGCAGAGTGT-TAAGGGCGAAGGC-
ATTTATCCAATAAAGATTGACGCTGAGAAACGAAGGTGAGGACAGAAAAAAGGATTAGAGACC

CTTGTATCCCTCACTGTCAACGATGAATGGTAGTTACTAGTAAGAA-----------------------
----
ATAAGCTAGAGACGATGTTAACACGAAAACCATTCCGCCTTGTGAGTACGACTGCAAAGTTGAA
AACAAAAAAATTAGTCGGTTTCGGAG--CAAACGAAGTGAAGC-A------------
GGTACTGGTGAGTTCGAGGCTGGTATCTCCAAGGATGGCCAGACTCGTGAGCACGCGCTGCTT
GCGTTTACCCTTGGTGTGCGACAACTCATTGTCGCCGTCAACAAAATGGACACCACGAAGGTTT
GTTGCTTTAACAAA--AAAAAATTGCACATGTGTTTATTT--AAATCTT----------
GCCACAGTGGTCCCAGGATCGTTTCAACGAAATGATCAAGGAAACGTCCAACTTCATCAAGAA
AGTCGGCTACAACCCTAAGACAGTTGCTTTCGTCCCGATTTCCGGCTGGCACGGTGACAACATG

>Pallidohirschioporus_brastagii_Dai_12316
-CGAGTTTTGA-----A------GTGGG--------------------------------CTTGATGCTG--
GCTTG---------------TAACAG--AGCACTGTG-CTCAGC------------------------------
--CCC--------GCTCCAATCC--A--TTC------------------AA-CCCC--TGTGCA-
CTATTCGG--AGT--------------------
-------------------------------------GTTGCAAGCTGAGA----------C---------------
--------------------------AATGTGGGGAG---

--------------GTAGTCTCGGTC------------GTATTTCTAA-------TGCGACTTGGGG----
-----------------TTACTT-----------TCAAAC-GGTCAAGGCTTGTCC-TCCGG------
TTTATA-TTAC-------------AA---ACACTTTTA-TTGTCTTGTCGAAT-G-----T-ATTAGCCT-
CT-CG----TTAGGCGAAACTT-----
AAATACAACTTTCAACAACGGATCTCTTGGCTCTCGCATCGATGAAGAACGCAGCGAAATGCGA
TAAGTAATGTGAATTGCAGAATTCAGTGAATCATCGAATCTTTGAACGCACCTTGCGCTCCTTGG
CTATTCCGAGGAGCATGCCTGTTTGAGTGTCATGTTAATATCAACTCCG--------ATG-
GTTTTTGTTAA--------------------------CTATTGGGTGTTGGAC-TTGGAGG-T-T------
CGTGCTGGC----------------------------TGCAAAGTCGGCTCCTCTTGAATGCATTAGC-
TTGGACCTGT-------------GCGCGGT---TTG-CTA-----------GCGGTGTAATACATTTTA-

----------------TTCACCAC---GGGC-CGTGTCACTATT-AGGGTCTGC-TTC-----
TAATCGTCCTT--
----------------CCTAGTAACTGCGAGTGAAGCGGGAAAAGCTCAAATTTAAAATCT-G-
GCG-GCTT----TGTCGTCCGAGTTGTAATCTGGAGAAGCGTTTT-CCGTGTCG-
GACCGTGCACAAGTCTCTTGGAACAGAGCGTCATAGAGGGTGAGAATCCCG-
TCTTTGGCACGG-AC-AGCCGATAC-
TTTGTGATACGTTTTCAAAGAGTCGAGTTGTTTGGGAATGCAGCTCAAAATGGGTGGTAAATTCC
ATCTAAAGCTAAATATAGGCGAGAGACCGATAGCGAACAAGTACCGTGAGGGAAAGATGAAA
AGCACTTTGGAAAGAGAGTTAAATAGTACGTGAAATTGTTGAAAGGGAAACGCTTGAAGTCAGT
CGCGTCTGCCGGGACTCAGCCTTAC----TTTTGTATGGTGTATTTCCTGGTT---
TGACGGGTCAACATCGA-TTTT-GAC-CGATGGATAAAGGAGAAGGGAATGTGGCACCTCC----
GGGTGTG-TTATAGC-CCTCT-TT-GTAT-ACATCGGTTGGGATCGAGGA-CTGCAGCACGCCC--
----TT-G-TGGCC-------GGGGG----------------------TT---CG-CCCTACG-T-
ATCGTGCTTAGGAT-
GTTGACATAATGGCTTTAAGCGACCCGTCTTGAAACACGGACCAAGGAGTCTAACATGCTCGCG
AGTGTTAGGGTGGAAAACCCTTGCGCGTAATGAAAGTGAAG-GTTGGGA------CCTC-------
-----------TGCGA--GGAGG-GCACCGACGCCCGGCCCTGAAG-----
TTCTCTGACGGTGCTGCGGTAGAGCGCGTATGTTGGG----
ACCCGAAAGATGGTGAACTATGCCTGAATAGGGCGAAGCCAGAGGAAACTCTG-
GTGGAGGCTCGTAGCGATTCTGACGTGCAAATCGATCGTCAAATTTGGGTCGATGTGTC--AT--
-TGGGGATAACGTAAGGGAAAAT-
AATGATATTACCTTACTATTAGTGTCGTCCAAGTACTCGTGCCAGAAGACTCGGTAAGGCGAGA
GACGCAAACGTTAGTCGTCTTGATCAGGCGTAAAGGGTTTGTAGGCGGCTTTAAGTATCTAA-
TTTTTAGTTTACTATTTT--------------------------------AATATAGGAA-------------
AAATTT---
TTATTTTTTATTTTCATAAAAATTATAATTTAATATTAAAAAAATAAAGAAAGGATCAATTAAAGCT
AGAATCTAAAAGAGGTTA----AAACAATACTTCAG--
AAAGGAGTGCTAACCGTAGATAAGGAGCAGAGTGT-TAAGGGCGAAGGC-
ATTTATCCAATAAAGATTGACGCTGAGAAACGAAGGTGAGGACAGAAAAAAGGATTAGAGACC
CTTGTATCCCTCACTGTCAACGATGAATGGTAGTTACTAGTAAGAA-----------------------
----
ATAAGCTAGAGACGATGTTAACACGAAAACCATTCCGCCTTGTGAGTACGACTGCAAAGTTGAA
AACAAAAAAATTAGTCGGTTTCGGAG--CAAACGAAGTGAAGC-A------------
GGTACTGGTGAGTTCGAGGCTGGTATCTCCAAGGATGGCCAGACTCGTGAGCACGCGCTGCTG
GCGTTTACCCTTGGTGTGCGACAACTCATTGTCGCCGTCAACAAAATGGACACCACGAAGGTTT
GTTG-TTTAACAAA--AAAAAATTGCATAAGTGTTTATTT--AAATCTT----------
GTTACAGTGGTCCCAGGATCGTTTCAACGAAATCGTCAAGGAAACGTCCAACTTCATCAAGAAA
GTCGGCTACAACCCTAAGACAGTTGCTTTCGTCCCGATTTCCGGCTGGCACGGTGACAACATGA
TTGAGGAATCTGTCAAGTAGGTGGTTTTG-GAGCG---GTCTATACAATATAT-----
ATGCTCACCGCATGTTTT-
CATAGCATGCCGTGGTACAAAGGTTGGACGAAGGAAGTTAAGGGCA---------
AAGGCGAGGTG-ACCGGCAAGACCCTCCTAGAGGCTATCGATGCCATTGAACCTCCT--------
----

>Pallidohirschioporus_polycystidiatus_Dai_14686
-CGAGTTTTGA-----A------GTGGG------------------------------CTTGATGCTG--
GCTTT---------------TTA-----AGCACTGTG-CTCAGC-------------------------------
-CCC--------GCTCCAATCC--A--TTC-----------------AA-CCCC--TGTGCA-
CTATTCGG---AGT-------------------
-------------------------------------GTTGCAAGCTGAGA----------C--------------
-------------------------AATGTGGGCG--

---------------TAGTCTAAGTT------------GTATT----------------ACTTGGG--------
--------------TTACAA-----------CCAAAC-GGTCAAGGCTTGTCC-TCTGG-------
TTATA-TTAC-------------AA---ACACGTTTA-TTGTCTTGTAGAAT-G-----T-ATTAGCCT-
CT-TG----TTGGGCGAAACTT-----
AAATACAACTTTCAACAACGGATCTCTTGGCTCTCGCATCGATGAAGAACGCAGCGAAATGCGA
TAAGTAATGTGAATTGCAGAATTCAGTGAATCATCGAATCTTTGAACGCACCTTGCGCTCCTTGG
CTATTCCGAGGAGCATGCCTGTTTGAGTGTCATGTTAATATCAACTCCG--------ATG-
GTTTTTA-TAA-------------------------CTATTGGGTGTTGGAC-TTGGAGG-T-T------
CGTGCTGGC---------------------------TGCAAAGTCGGCTCCTCTTGAATGCATTAGC-
TTGGACCTGT-------------GCGC-GT---TTG-CTA-----------GCGGTGTAATACATTTTA--
---------------TTCACCAC---GGGC-CGTGTCACTCTT-AGGGTCTGC-TTC-----
TAATCGTCCTC---
----------------CCTAGTAACTGCGAGTGAAGCGGGAAAAGCTCAAATTTAAAATCT-G-
GCG-GCTC----TGTCGTCCGAGTTGTAATCTGGAGAAGCGTTTT-CCGTGTCG-
GACCGTGCACAAGTCTCTTGGAACAGAGCGTCATAGAGGGTGAGAATCCCG-
TCTTTGGCACGG-AC-AGCCGATAC-
TTTGTGATACGTTTTCAAAGAGTCGAGTTGTTTGGGAATGCAGCTCAAAATGGGTGGTAAATTCC
ATCTAAAGCTAAATATAGGCGAGAGACCGATAGCGAACAAGTACCGTGAGGGAAAGATGAAA
AGCACTTTGGAAAGAGAGTTAAATAGTACGTGAAATTGTTGAAAGGGAAACGCTTGAAGTCAGT
CGCGTCTGCCGGGACTCAGCCTTAC----TTCTGTATGGTGTATTTCCTGGTT---
TGACGGGTCAACATCGA-TTTT-GAC-CGATGGATAAAGGAGAAGGGAATGTGGCACCTCC----
GGGTGTG-TTATAGC-CCTCT-TT-GTAT-ACATCGGTTGGGATCGAGGA-CTGCAGCACGCCC--
----TT-G-TGGCC-------GGGGG----------------------TT---CG-CCCTACG-T-

ATCGTGCTTAGGAT-
GTTGACATAATGGCTTTAAGCGACCCGTCTTGAAACACGGACCAAGGAGTCTAACATGCTCGCG
AGTGTTAGGGTGGAAAACCCTTGCGCGTAATGAAAGTGAAG-GTTGGGA------CCTC-------
-----------TGCGA--GGAGG-GCACCGACGCCCGGCCCTGAAG-----
TTCTCTGACGGTGCTGCGGTAGAGCGCGTATGTTGGG----
ACCCGAAAGATGGTGAACTATGCCTGAATAGGGCGAAGCCAGAGGAAACTCTG-
GTGGAGGCTCGTAGCGATTCTGACGTGCAAATCGATCGTCAAATTTGGGTCAATGTGTC--AT--
-TGGGGATAACGTAAGGGAAAAT-
AATGATATTACCTTACTATTAGTGTCGTCCAAGTACTCGTGCCAGAAGACTCGGTAAGGCGAGA
GACGCAAACGTTATTCGTCTTGATCAGGCGTAAAGGGTTTGTAGGCTGCTTTAAGTATCTAA-
TTTTTAGTTTACTATTTT-------------------------------AATCTTTAAA-------------
AATAAA---
TATATAATAAAAT--
ATATATTAAATAATATAATATTAAAAAAATAAAGAAAGGATCAATTAAAGCTAGAATCTAAAAGA
GGTTA----AAACAATACTTCAG--AAAGGAGTGCTAACCGTAGATAAGGAGCAGAGTGT-
TAAGGGCGAAGGC-
ATTTATCCAATAAAGATTGACGCTGAGAAACGAAGGTGAGGACAGAAAAAAGGATTAGAGACC
CTTGTATCCCTCACTGTCAACGATGAATGGTAGTTACTAGTAAGAA-----------------------
----
ATAAGCTAGAGACGATGTTAACACGAAAACCATTCCGCCTTGTGAGTACGACTGCAAAGTTGAA
AACAAAAAAATTAGTCGGTTTCGGAG--CAAACGAAGTGAAGC-A------------
GGTACTGGTGAGTTCGAGGCTGGTATCTCCAAGGATGGCCAGACTCGTGAGCACGCGCTGCTT
GCGTTCACTCTCGGTGTGCGTCAACTCATTGTCGCCGTCAACAAAATGGACACCACGAAGGTCA
GTTG-TTTGACAAG--ATTTTA-TATCAATGCGTTTATTA--ATAT-TT----------
GTTTTAGTGGTCCGAGGATCGTTTTAACGAAATTGTCAAGGAAACGTCCAACTTCATCAAGAAG
GTCGGTTACAACCCCAAGACCGTCGCTTTCGTCCCCATCTCCGGCTGGCATGGTGATAACATGA
TTGAGCCCTCTACAAAGTGAGTGGGAGGT-GATTGT--GATTTAATAAGATAT-----
ATGCTTACAATATATTTT-CACAGCATGCCGTGGTACAAGGGCTGGACCAAGGAGACGAAGG--
----------CCGGCTCGTCG-
ACTGGTAAGACCCTCCTCGAGGCCATCGATGCCATTGAACCTCCTTCCCGTCCTTCCGACAAGC
CCCTCCGTCTCCCGCTCCAGGATGTC--

>Pallidohirschioporus_versicolor_Dai_19332
-CGAGTTTTGA-----A------GTGGG----------------------------CTTGATGCTG--
GCTTG---------------TAACAG--AGCACTGTG-CTCAGC----------------------------

--CCC--------GCTCCAATCC--A--TTC-----------------AA-CCCC--TGTGCA-
CTATTCGG--AGT-------------------
------------------------------------GTTGCAAGCTGAGA----------C--------------
-------------------------AATGTGGGTG---

----------------TGACCCGGTT------------GTATTTTTAA-------TGCGACTTGGG------
----------------TTACTA-----------TCAAAC-GGTCAAGGCTTGTCC-TCTGG------
TTTATA-TTAC-------------AA---ACACTTTTA-TTGTCTTGTAGAAT-G-----T-ATTAGCCT-
CT-CG----TTAGGCGAAACTT-----
AAATACAACTTTCAACAACGGATCTCTTGGCTCTCGCATCGATGAAGAACGCAGCGAAATGCGA
TAAGTAATGTGAATTGCAGAATTCAGTGAATCATCGAATCTTTGAACGCACCTTGCGCTCCTTGG
CTATTCCGAGGAGCATGCCTGTTTGAGTGTCATGTTAATATCAACTCCG--------ATG-
GTTTTTGTTAA-------------------------CTATTGGGTGTTGGAC-TTGGAGG-T-T------
AGTGCTGGC---------------------------TGCAAAGTCGGCTCCTCTTGAATGCATTAGC-
TTGGACCTGT-------------GCGC-GT---TTG-CTA-----------GCGGTGTAATACATTTTA--
---------------TTCACCAC---GGGC-CGTGTCACTATT-AGGGTCTGC-TTC-----
TAATCGTCCTC--
----------------CCTAGTAACTGCGAGTGAAGCGGGAAAAGCTCAAATTTAAAATCT-G-
GCG-GCTT----TGTCGTCCGAGTTGTAATCTGGAGAAGCGTTTT-CCGTGTCG-
GACCGTGCACAAGTCTCTTGGAACAGAGCGTCATAGAGGGTGAGAATCCCG-
TCTTTGGCACGG-AC-AGCCGATAC-
TTTGTGATACGTTTTCAAAGAGTCGAGTTGTTTGGGAATGCAGCTCAAAATGGGTGGTAAATTCC
ATCTAAAGCTAAATATAGGCGAGAGACCGATAGCGAACAAGTACCGTGAGGGAAAGATGAAA
AGCACTTTGGAAAGAGAGTTAAATAGTACGTGAAATTGTTGAAAGGGAAACGCTTGAAGTCAGT
CGCGTCTGCCGGGACTCAGCCTTACT---TTTTGTATGGTGTATTTCCTGGTT---
TGACGGGTCAACATCGA-TTTT-GAC-CGATGGATAAAGGAGAAGGGAATGTGGCACCTCC----
GGGTGTG-TTATAGC-CCTCT-TT-GTAT-ACATCGGTTGGGATCGAGGA-CTGCAGCACGCCC--
----TT-G-TGGCC-------GGGGG----------------------TT---CG-CCCTACG-T-
ATCGTGCTTAGGAT-
GTTGACATAATGGCTTTAAGCGACCCGTCTTGAAACACGGACCAAGGAGTCTAACATGCTCGCG
AGTGTTAGGGTGGAAAACCCTTGCGCGTAATGAAAGTGAAG-GTTGGGA------CCTC-------
-----------TGCGA--GGAGG-GCACCGACGCCCGGCCCTGAAG-----
TTCTCTGACGGTGCTGCGGTAGAGCGCGTATGTTGGG----
ACCCGAAAGATGGTGAACTATGCCTGAATAGGGCGAAGCCAGAGGAAACTCTG-
GTGGAGGCTCGTAGCGATTCTGACGTGCAAATCGATCGTCAAATTTGGGTAGATGTTTCATAT--
-AATGGATCACGTAAGGGAAAAT-
AATGATATTACCTTACTATTAGTGTCGTCCAAGTACTCGTGCCAGAAGACTCGGTAAGGCGAGA
GACGCAAACGTTAGTCGTCTTGATCAGGCGTAAAGGGTTTGTAGGCGGCTTTAAGTATCTAA-
TTTTTAGTTTACTATTTT-------------------------------AATATAGGAA-------------
AAATT---

TTATTTATTTTCATAAAAATTATAATTTAATATTAAAAAAATAAAGAAAGGATCAATTAAAGCTAG
AATCTAAAAGAGGTTA----AAACAATACTTCAG--
AAAGGAGTGCTAACCGTAGATAAGGAGCAGAGTGT-TAAGGGCGAAGGC-
ATTTATCCAATAAAGATTGACGCTGAGAAACGAAGGTGAGGACAGAAAAAAGGATTAGAGACC
CTTGTATCCCTCACTGTCAACGATGAATGGTAGTTACTAGTAAGAA-----------------------
----
ATAAGCTAGAGACGATGTTAACACGAAAACCATTCCGCCTTGTGAGTACGACTGCAAAGTTGAA
AACAAAAAAATTAGTCGGTTTCGGAG--CAAACGAAGTGAAGC-A------------
GGTACTGGTGAGTTCGAGGCTGGTATCTCCAAGGATGGCCAGACTCGTGAGCATGCGCTGCTTG
CGTTCACTCTTGGTGTGCGTCAACTCATTGTCGCCGTCAACAAAATGGATACCACAAAGGTCTGT
TA-TTTAACAAA--ATTTCTATAGTAATGTGTTTATTT--AAATTTT----------
ATTACAGTGGTCCCAGGATCGTTTCAACGAAATTGTCAAGGAAACGTCCAATTTCATCAAGAAA
GTTGGCTACAACCCTAAGGCGGTCGCTTTCGTTCCGATCTCCGGCTGGCACGGTGACAACATGA
TTGAGGAGTCTACCAAGTACGTGGAACTT-GAATTT--GTCTATATAAGATCT-----
ATGCTCACAGTATGTTTT-
CATAGCATGCCGTGGTACAAAGGCTGGTCAAAGGAAATAAAGACCAAGAGCGGGCCCGAAAC
GGTG-
ACCGGCAAGACTCTCCTGGACGCCATCGATGCCATTGAACCTCCTTCTCGTCCGTCGGACAAAC
CTCTCCGCCTTCCTCTCCAGGATGTC---

>Phellinopsis_asetosa_Dai_13553
-TGAGTCTTGA-----A-G--CAAGCTT-----------------------------TTTGTTGCTG--
GCGCA---------------GAGATG--CGCA-CGTG-CACAAG---------------------------
---CTT-TGCG-----CTCAATCC-AA--CTC-----------------AA-CCCC--TGTGCA-
CCTATTG---GAG------------------
-----------------------------------TTAGTAGT---------------C------TCTTCCG--
-------------------------GTCCGGTACAC--------CTCTCTAAGGTGTCTGATTTGG------
----

-------------------------GCGAACACT-------------------------
TCAAGTCCTTGAAGT--
CAGTAGTTGGAAA--CCTGGGA---
--AGGGG----------
TTCAGCTTGTGTCAGTAGTGTGTGGTGGAGAACGCTGCA-------TGCAAAAAGCAC---------
------TTGCCTTTGCCTC----------GAAAGG-GAGACTGGCGAGCGC-TTTGA------

GTCTTA--TAA-------------AA---ATACTATAT-AAGTTTTGTTAGAATG-----T-AAT-GC-T-
CC-TT----GTGGGCGAAAC-------
AAATACAACTTTCAACAACGGATCTCTTGGCTCTCGCATCGATGAAGAACGCAGCGAAATGCGA
TAAGTAATGTGAATTGCAGAATTCAGTGAATCATCGAATCTTTGAACGCACCTTGCGCCCCTTGG
-TATTCCGAGGGGCATGCCTGTTTGAGTGTCATTTAAATTTCAAACCCC--------ATG-
TGCTTTTCA------------------AAGCTC--------TAGGTTTGGAC-TTGGAGG-T-T------
TCTGCTGGC-TCCT-------------------------GTTGTCGGCTCCTCTCAAATGCATTAGC-
TGGACTTTGG----------------T-TC---GCG-TAA-----------TCGGTGTGATAGTTGAC---
----------TCGA-TTCACC------GTG-CGCTTG-TCTAA-TGGGTCTGC-TTC-----
TAATGGCCTT------------------CGGGC-AA--------GG------CTTTAT-TGC----C-
TTCTATTGACCCTTTGACCTCATCCCCTAGTAACTGCGAGTGAAGCGGGAAGAGCTCAAATTTAA
AATCT-G-GCG-GTCT-TCCGGCCGTCCGAGTTGTAGTCTGGAGAAGTGTTTT-CCGCGTCG-
GACCGTGTACAAGTCTCCTGGAACGGAGTGTCAGAGAGGGTGAGAATCCCG-
TCCATGACACGG-AC-TCCCGATGC-
TATGTGATACGCTTTCAAAGAGTCGAGTTGTTTGGGAATGCAGCTCAAAATGGGTGGTAAATTC
CATCTAAAGCTAAATATTGGCGAGAGACCGATAGCGAACAAGTACCGTGAGGGAAAGATGAAA
AGCACTTTGGAAAGAGAGTTAAACAGTACGTGAAATTGTTGAAAGGGAAACGCTTGAAGTCAG
TCGCGTCCCTCGGAACTCAGCCTTGC----TTTGGCTTGGTGTACTTTCCGT-GT--
GGATGGGTCAACGTCAG-TTTC-TGC-
CGGTGGAGAAAGGGAAGGGGAATGTGGCGCCGCCTCGGTGGCGTG-TTATAGC-CCTTTTCC-
TGAT-GCATTGGTGGGGACTGAGGA-CCGCAGCGCGCCT------TT-T-TGGCC-------
GGGGG----------------------TT---CG-CCCCACG-T-ATCGCGCTTAGGAT-
GTTGGCATAATGGCTTTAAGCGACCCGTCTTGAAACACGGACCAAGGAGTCTAACATGCTTGCG
AGTGTTCGGGTGGAAAACCCTTGCGCGTAATGAAAGTGAAA-GTTGGGAA-----CCTC-------
---------------------GGTGCACCGACGCCCGGCCCTGACG-----
TTTTCTGAAGGTGCTGCGGTAGAGCAAGTATGCTGGG----
ACCCGAAAGATGGTGAACTATGCCTGAATAGGGCGAAGCCAGAGGAAACTCTG-
GTGGAGGCTCGTAGCGATTCTGACGTGCAAATCGATCGTCAAATTTGGG------------------
----

>Phellinopsis_conchata_L-7601
ACGAGTTTTGA-----A-A---GCGAGC------------------------------GTTGTTGCTG--
GCGCG---------------GAGACG--CGCA-TGTG-CACGGG----------------------------
---CTC--GCA-----CTCAATCC-AA--CTCTC--------------AAC-CCCC--TGTGCA-
CCTGTTG--AAG-------------------
-------------------------------------TCAGTAGT----------------C------TTTT------
-------------------------TCCGGTACC----------TCTTTGAGGGACCGGTGGGAGGA----
----

-------------------------GCGAACACT--------------------------TCAAAT-
CTTGAAGT--CAGTAGTTGGAAA--------
--CCTGGAA---------------------------
--------------AGGGGGG----------
TCAGGCTTGCGTCAGTAGTAAGCGGTGGAGGACGCCGCA-------TGCGAAAGCAAT--------
-------TTGCCTTTGCCTC----------GGAAGG-GAGACCGGCGAGCGC-TTCGA------
GTCTT----AT-------------AA---ACCTTTTAC-AAGTCATGTTAGAATG-----T-AAT-GC-
TCCC-TT----GTGGGCGAAAC-------
AAATACAACTTTCAACAACGGATCTCTTGGCTCTCGCATCGATGAAGAACGCAGCGAAATGCGA
TAAGTAATGTGAATTGCAGAATTCAGTGAATCATCGAATCTTTGAACGCACCTTGCGCCCCTTGG
-TATTCCGAGGGGCATGCCTGTTTGAGTGTCATTTAAATCTCAAACCCT--------TTG-
CTTTTCG--------------------AAGCCC--------TGGGTTTGGAC-TTGGAGG-T-T------
TTTGCTGGC-CCCC---------------------AACGGTCGTCGGCTCCTCTGAAATGCATTAGC-
TGGACTTTGG----------------T-TC---GCG-TGA-----------TCGGTGTGATAGTTAAT---
----------TCGA-TTCGCC------GTG-CGCTTG-CCTAA-CGGGTCTGC-TTC-----
TAATGGCCTT------------------CGGGC-AA--------GG------CGGA---AGC----C-
TTCTATTGACCCTTTGACCTCA-----
TAGTAACTGCGAGTGAAGCGGGAAGAGCTCAAATTTAAAATCT-G-GCG-GTCT-
TCTGGCCGTCCGAGTTGTAGTCTGGAGAAGTGTTTT-CCGCGTCG-
GACCGTGTACAAGTCTCCTGGAACGGAGCGTCATAGAGGGTGAGAATCCCG-
TCCATGACACGG-ATCCTCCGATGC-
TATGTGATACGCTTTCAAAGAGTCGAGTTGTTTGGGAATGCAGCTCAAAACGGGTGGTAAATTC
CATCTAAAGCTAAATATTGGCGAGAGACCGATAGCGAACAAGTACCGTGAGGGAAAGATGAAA
AGCACTTTGGAAAGAGAGTTAAACAGTACGTGAAATTGTTGAAAGGGAAACGCTTGAAGTCAG
TCGCGTCCCTTGGAACTCAGCCTTGC----TTCGGCTTGGTGTACTCTCTGA-GT--

GGACGGGTCAACGTCAG-TTTC-TGC-
CGACGGAGAAAGGGAGGGGGAATGTGGCGCCGCTTCGGCGGCGTG-TTATAGC-CTCCTTCC-
TGAT-GCGTCGGTGGGGACTGAGGA-CCGCAGCGCGCCT------TC-T-TGGCC-------
GGGGG----------------------TT---CG-CCCCACG-T-ACCGCGCTTAGGAT-
GTTGGCATAATGGCTTTAAGCGACCCGTCTTGAAACACGGACCAAGGAGTCTAACATGTTTGCG
AGTGTTCGGGTGGAAAACCCTTGCGCGTAATGAAAGTGAAA-GTTGGGAA-----CCTC-------
-----------CGCGA--GGGGGTGCACCGACGCCCGGCCCTGACG-----
TTCTCTGACGGTGCTGCGGTAGAGCAAGTATGCTGGG----
ACCCGAAAGATGGTGAACTATGCCTGAATAGGGCGAAGCCAGAGGAAACTCTG-
GTGGAGGCTCGTAGCGATTCTGACGTGCAAATCGATCGTC----------------------------
----

GGTACTGGTGAGTTCGAGGCTGGTATCTCGAAGGACGGTCAGACTCGCGAGCACGCTCTCCTT
GCGTTCACCCTCGGTGTGCGTCAGCTGATCGTTGCTGTCAACAAAATGGACACCACCAAGGTTA
GCATCGGATACCTGC-----CAAATTATGACAGTCAAGACT-CACTTTG----------
TCTTCAGTGGTCTGAGGATCGCTTCAATGAAATTGTCAAGGAAACCTCCAACTTCATCAAGAAG
GTCGGATACAACCCGAAATCAGTTGCCTTCGTCCCTATTTCTGGCTGGCACGGAGACAACATGT
TGGAGGAGTCTCCCAAGTAAGCTACCATT-AATTTAT------TGTACGTGAT-----
CTGAAGCTAAATGATGCT-TGCAGCATGCCGTGGTACAAGGGCTGGACTAAGGAAACCAAGG-
-----------CGGGTGTCGTA-
AAGGGCAAGACTCTCCTCGATGCCATCGATGCCATCGAACCCCCAGTGCGTCCTGAGAACAAG
CCTCTCCGACTTCCTCTCC--------
ATCTCCGTCGGCTCAAACTCAGCGCCTGTTCTCGAATTCCTCGAAGAGTGGGGCTTGGAGAGTC
TTGAAGAAAACATCCA---GTCCACGA------
ATCTTACCAAAGTCTTCGTAAACGGTGTCTGGTTGGGGGTACACCGTGATCCATTGAACCTGGT
AAAGACTCTTCGCGTACTGAGGCGGAGGG---
AGGATATAAGCTACGAAGTCTCTGTTGTTCGTGACATCCGCGAGAAGGAATTTCGCATCTATAC
CGACGCCGGACGGGTACTTCGACCGCTCTTTATTGTAGAGGACCGAAAATTGAAGTTGAAGCGT
CAGCACCTACGCTGGCTTCAAGAGAAGTGGCGAATA-------
GAAGAAAGACCCATCGTCTTGCCG------
CATGAACGGGAAGAAGGTGAAGTTTCGGCGGGTGAAACGATAGCGGAG---
GAGGAAGACGGCATGCCAATCATGAGGGAAGGAATAAAAATGAAGAAAAAAAGGAAGCCTTT
CAGATTCCACTCTTTGGTGCATGATGGTGTCATTGAGTATTTGGACGCCGAGGAAGAGGAGACG
GTCATGATCTGTATGACACCGGATGATCTAGTCGAGAGTAGACTAATCCGTAGAGGCTTGGACC
CACG---AGCAGGCGCAGAGCTTGATCGAGCTGCTCGCTTAAAGCCGGTGCCAGTG------
GATCACGAATGGACGCATTGCGAAATCCACCCCAGCATGATTCTAGGCATTTGTGCTAGTATCA
TACCATTCCC

>Phellinopsis_lonicericola_Dai_22911
ATGAGTTTTGA-------A---GCAAGC------------------------------CTCGCCGCTG--
GCGTG---------------GAGACG--CGCA-TGTG-CACGGG----------------------------
---CTT--GCA----CCTCAATCC-AA--CTC-----------------AA-CCTC--TGTGCA-
CCCGTTG---AAG-------------------
------------------------------------TCAGTAGT----------------C------TCTTC----
--------------------------TTCGGTACC----------TCTTTGAGGGACCGGTGGGAG------
----

-------------------------GCGAACATT--------------------------TCAAAT-
CTTGAAGT---CAGTAGTTGGAAT--------
---CCCGGAACGG-----------------------
--------------ATGGGGG----------
TCAGGCTTGGGCCAGTAGCGAGTGGTGGAGGACGCCGCA-------TGCGATTTGCCC--------
-------TTCGCCTCGCCTCGGAA------GGAAGG-GAGACCGGCGAGCGC-TTTGA------
GTCTT----AC-------------AA---ACCTTTTAC-AAGTCACGTTAGAATG-----T-AAT-GC-T-
CC-TT----GTGGGCGAAAGAA-----
ATATATAACTTTCAACAACGGATCTCTTGGCTCTCGCATCGATGAAGAACGCAGCGAAATGCGA
TAAGTAATGTGAATTGCAGAATTCAGTGAATCATCGAATCTTTGAACGCACCTTGCGCCCCTTGG
-TATTCCGAGGGGCATGCCTGTTTGAGTGTCATTTAAATCTCAAACCCT--------TTG-
CTTTTCG--------------------AAGCCC--------TGGGTTTGGAC-TTGGAGG-T--------
TTTGCTGGC-CCTT---------------------AGTGGTTGTCGGCTCCTCTGAAATGCATTAGC-
TGGACTTTGG----------------T-TC---GCG-TGA-----------TCGGTGTGATAGTTAAC---
----------TCGA-TTCGCC------GCA-CGCTTG-CCTAA-CGGGTCCGC-TCC-----
TAATAGCCTT------------------CGGGCGGA--------GG------CGTA---AGC----C-
TTCTATTGACCCTTTGACCTCA-
CCCCTAGTAACTGCGAGTGAAGCGGGAAGAGCTCAAATTTAGAATCT-G-GCGTGTCT-
TCCGGCCGTCCGAGTTGTAGTCTGGAGAAGTGTTTT-CCGCGTCG-
GACCGTGTACAAGTCTCCTGGAACGGAGCGTCATAGAGGGTGAGAATCCCG-
TCCATGACACGG-CC-TCCCGATGC-
TATGTGATACGCTTTCGAAGAGTCGAGTTGTTTGGGAATGCAGCTCAAAATGGGTGGTAAATTC
CATCTAAAGCTAAATATTGGCGAGAGACCGATAGCGAACAAGTACCGTGAGGGAAAGATGAAA
AGCACTTTGGAAAGAGAGTTAAACAGTACGTGAAATTGTTGAAAGGGAAACGCTTGAAGTCAG
TCGCGTCCCTTGGAACTCAGCCTTGC----TTCGGCTTGGTGTATTCTCTGA-GT--
GGACGGGTCAACGTCAG-TTTC-TGC-
CGACAGAGAAAGGGAGGGGGAATGTGGCGCCGCTTCGGCGGCGTG-TTATAGC-CTCCTTCC-
TGAT-GTGTCGGTGGGGACTGAGGA-CCGCAGCGCGCCT------TT-T-TGGCC-------
GGGGG----------------------CT---CG-TCCCACG-T-ACCGCGCTTAAGAT-
GTTGGCATAATGGCTTTAAGCGACCCGTCTTGAAACACGGACCAAGGAGTCTAACATGCTTGCG
AGTGTTAGGGTGGAAAACCCTTGCGCGCAATGAAAGTGAAA-GTTGGGAA-----CCTC-------
-----------CGCGA--GGGGGTGCACCGACGCCCGGCCCTGACG-----
TTCTCTGACGGTGCTGCGGTAGAGCAAGTATGCTGGG----
ACCCGAAAGATGGTGAACTATGCCTGAATAGGGCGAAGCCAGAGGAAACTCTG-
GTGGAGGCTCGTAGCGATTCTGACGTGCAAATCGATCGTCAAATTTGGG------------------

----

>Phellinopsis_tibetica_LWZ_20190808-31a
ATGAGTTTTGA-----A-A---GCAAGC---------------------------------CTCGCCGCTG--
GCGCG----------------GAGACG--CGCA-TGTG-CACGGG-------------------------------
---CTT--GCA----CCTCAATCC-AA--CTC------------------AA-CCCC--TGTGCA-
CCCGTTG---AAG---------------------
--TCAGTAGT------------------C------TCTTC----
-----------------------------TTCGGTACC----------TCTTTGAGGGACCGGTGGGAG------
----

---------------------------GCGAACATT----------------------------TCAAAT-
CTTGAAGT---CAGTAGTTGGAAT--------
---CCCGGAACGG-------------------------
---------------ATGGGGGG----------
TCAGGCTTGGGCCAGTAGCGAGTGGTGGAGGACGCCGCA-------TGCGATTTGCCC--------
-------TTCGCCTCGCCTCGGAA------GGAAGG-GAGACCGGCGAGCGC-TTTGA------
GTCTT----AC-------------AA---ACCTTTTAC-AAGTCACGTTAGAATG-----T-AAT-GC-T-
CC-TT----GTGGGCGAAAGAA-----
ATATATAACTTTCAACAACGGATCTCTTGGCTCTCGCATCGATGAAGAACGCAGCGAAATGCGA

TAAGTAATGTGAATTGCAGAATTCAGTGAATCATCGAATCTTTGAACGCACCTTGCGCCCCTTGG
-TATTCCGAGGGGCATGCCTGTTTGAGTGTCATTTAAATCTCAAACCCT--------TTG-
CTTTTCG--------------------AAGCCC--------TGGGTTTGGAC-TTGGAGG-T--------
TTTGCTGGC-CCTT---------------------AGTGGTTGTCGGCTCCTCTGAAATGCATTAGC-
TGGACTTTGG----------------T-TC---GCG-TGA-----------TCGGTGTGATAGTTAAC---
----------TCGA-TTCGCC------GCA-CGCTTG-CCTAA-CGGGTCCGC-TCC-----
TAATAGCCTT------------------CGGGCGGA--------GG------CGTA---AGC----C-
TTCTATTGACCCTTTGACCTCA-
CCCCTAGTAACTGCGAGTGAAGCGGGAAGAGCTCAAATTTAGAATCT-G-GCG-GTCT-
TCCGGCCGTCCGAGTTGTAGTCTGGAGAAGTGTTTT-CCGCGTCG-
GACCGTGTACAAGTCTCCTGGAACGGAGCGTCATAGAGGGTGAGAATCCCG-
TCCATGACACGG-CC-TTCCGATGC-
TATGTGATACGCTTTCGAAGAGTCGAGTTGTTTGGGAATGCAGCTCAAAATGGGTGGTAAATTC
CATCTAAAGCTAAATATTGGCGAGAGACCGATAGCGAACAAGTACCGTGAGGGAAAGATGAAA
AGCACTTTGGAAAGAGAGTTAAACAGTACGTGAAATTGTTGAAAGGGAAACGCTTGAAGTCAG
TCGCGTCCCTTGGAACTCAGCCTTGC----TTCGGCTTGGTGTATTCTCTGA-GT--
GGACGGGTCAACGTCAG-TTTC-TGT-
CGACAGAGAAAGGGAGGGGGAATGTGGCGCCGCTTCGGCGGCGTG-TTATAGC-CTCCTTCC-
TGAT-GTGTCGGTGGGGACTGAGGA-CCGCAGCGCGCCT------TT-T-TGGCC-------
GGGGG----------------------CT---CG-TCCCACG-T-ACCGCGCTTAAGAT-
GTTGGCATAATGGCTTTAAGCGACCCGTCTTGAAACACGGACCAAGGAGTCTAACATGCTTGCG
AGTGTTAGGGTGGAAAACCCTTGCGCGCAATGAAAGTGAAA-GTTGGGAA-----CCTC-------
-----------CGCGA--GGGGGTGCACCGACGCCCGGCCCTGACG-----
TTCTCTGACGGTGCTGCGGTAGAGCAAGTATGCTGGG----
ACCCGAAAGATGGTGAACTATGCCTGAATAGGGCGAAGCCAGAGGAAACTCTG-
GTGGAGGCTCGTAGCGATTCTGACGTGCAAATCGATCGTCAAATTTGGGTTGGTGTGTC--AT--
-CGAGGATAACGTAAGGGAAAAT-
AATGATATTACCTTACTATTAGTGTCGTCCAAATGCTCGTGCCAGAAGACTCGGTAAGACGAGA
GACGCTAACGTTATTCGTCTTGATCAGGCGTAAAGGGTTTGTAGGCGGCTTTAAGTATTCAA-
TATTAAGTTTACAATTAT--------------------------------AATCTTTTTT---------------
----
-------------TTAATA--
ATTATAATATTTATTAAAGGATCAATTAAAGCTAGAATCTAACAGAGGTTA----
AAACAATACTTACA--GTAGGTGTACAAACCACAGATAGTAAGTAGAGTGT-
TAAGGGCGAAGGC-
GTTTATCCACTGAAGATTGACGCTGAGAAACGAAGGTGAGGACAGAAAAAAGGATTAGAGACC
CTTGTATCCCTCACTGTCAACGATGAATGGTAGTTACTAGGGAGAA-----------------------
----
ATAAACTAGAGACGATGTTAACACGAAAACCATTCCGCCTTGTGAGTACGACTGCAAAGTTGAA
AACAAAAAAATTAGTCGGTTTCGGAG--CAAACGAAGTGAAGC-ATGTTATTTA-------------
----

>Phellinus_castanopsidis_Cui_10153
ATGAGTTTTTTT--GAA-GC-AGGGGGG---------------------------------CCCTTTGCTG--
GCGTGGGG-----TTTTGCGCGCGCA--TATA-TGTG-CACGGC----------------------------
-----TTC-CGTGCT-TCCTCAATCC--A--CTC------------------AA-CCCC--TGTGCA-
CTTTATGG--GAG---------------------
--TTAGTAGT-----------------C------
GGCGGTGG-----------------------------TCTGTACTA----------------------------------
----

--CTTGTCGGCGAACAC-TTCAA------
TATATA-----C--A---------AA---ACCCATACTATTGTTATGTTGAAT-G-----T-AATAGC-
T-CC-TT----GTGGGCGAAATATC---
AAAATACAACTTTCAACAACGGATCTCTTGGCTCTCGCATCGATGAAGAACGCAGCGAAATGCG
ATAAGTAATGTGAATTGCAGAATTCAGTGAATCATCGAATCTTTGAACGCACCTTGCGCCCCTTG
G-TATTCCGAGGGGCATGCCTGTTTGAGTGTCATGTTCATCTCAACTTCC--------TTG-
TCTTTTTTA------------TTA---AGGCTT--------GGGTGTTGGAC-TTGGAGG-T-TG---
TATGTGCTGGC-CCCAGT-------------------
AGTGGTGGTCGGCTCCTCTTAAATGCATTAGC-TGGGCTTTGG----------------C-TC---
GCG-TTG-----------ACGGTGTAATAGGAATTAGTTCTATCTAAACACA-TTCACCC-----
TTG-CGCTTG-CCTAG-AGGGTCTGC-TTC-----TAATTGTCTTT-----------------TGGAC-
AAA--------------------------------T-
TTTTTCTTGACTCCTGACCTCATCCCCTAGTAACTGCGAGTGAAGCGGGAAAAGCTCAAATTTAA
AATCT-G-GCG-GTC--TATGGCCGTCCGAGTTGTAATCTGGAGAAGCGTTTT-CCGCGTTG-
GACCGTGCACAAGTCTCCTGGAACGGAGCGTCATAGAGGGTGAGAATCCCG-
TCCATGGCACGG-ACTACCCAATGC-
TTTGTGATACGCTCTCGAAGAGTCGAGTTGTTTGGGAATGCAGCTCAAAATGGGTGGTAAATTC
CATCTAAAGCTAAATACTGGCGAGAGACCGATAGCGAACAAGTACCGTGAGGGAAAGATGAAA

AGCACTTTGGAAAGAGAGTTAAACAGTACGTGAAATTGTTGAAAGGGAAACGCTTGAAGTCAG
TCGCGTCCGGCAGAACTCAGCCTGGCCCCTTTCGGCCCGGTGTACTTTCTGT-C---
GGACGGGTCAACATCAG-TTTT-GAC-
CGGTGGAAAAAGGCGAGGGGAATGTAGCGCCGTTTCGGCGGCGTG-TTATAGC-CCCTTGTT-
GTAT-ACATCGGTTGGGACTGAGGA-CGGCAGCACGCGCGCCTTCTT-T-
TGGCCGCGCCGGGGGTC----------------------TC---GG-CCCCACG-T-
CACGTGCTTCGGAT-
GTTGGCATAATGGCTTTAAGCGACCCGTCTTGAAACACGGACCAAGGAGTCTAACATGCTTGCG
AGTGTTCGGGTGGAAAACCCTTGCGCGTAATGAAAGTGAAA-GTTGGGAG-----CCTC-------
-----------CGCAA--GGGGGTGCACCGACGCCCGGCCCTGACG-----
TTCTCTGACGGTGCTGCGGTAGAGCAAGTATGCTGGG----
ACCCGAAAGATGGTGAACTATGCCTGAATAGGGCGAAGCCAGAGGAAACTCTG-
GTGGAGGCTCGTAGCGATTCTGACGTGCAAATCGATCGTCAAATTTGGG------------------
----

>Phellinus_mori_LWZ_20150825-1
ACGAGTTTGAA-----A----TCGATGG-------------------------------CTTGATGCTG--
GCGTT---------------ATAACG--CGCA-TGTG-CTCAGC------------------------------
--TCGTCGTA-----CTCAATCC--T--CTT-----------------AA-CCCC--TGTGCA-
CCTAATGG---GAG--------------------

----------------------------------TTTAGTAGT----------------C------
GGTGTGTTG--

----------------------TTGTATTAGTTC----------GGTCTATTGAT-------
CGAATATGCGCA---
CCGTCGACGAAACAC-TTCAA------TTTTTA-TTAT-------------CA---AC-GATTAC-
TTGTAATGTGGAAT-G-----T-AAT-GC-T-CCTTT----GTGAGCGATATAA-----
AGTTACAACTTTCAACAACGGATCTCTTGGCTCTCGCATCGATGAAGAACGCAGCGAAATGCGA
TAAGTAATGTGAATTGCAGAATTCAGTGAATCATCGAATCTTTGAACGCACCTTGCGCCCCTTGG
-TATTCCGAGGGGCATGCCTGTTTGAGTGTCATGTTAATCTCAACCCCC--------TTG-
TTATATTTCAT----------------TAACTTG-------GGACGTTGGAC-TTGGAGG-TAT----
TATATGCTGGC-TCCG---------------CTTTG--
GTTGTGGTCGGCTCCTCTTAAATGTATTAGC-TGGGCTTTGG----------------C-TC---GCG-
TTA-----------ACGGTGTAATAGTAACTTC-----------TACA-TTCATC------GGA-
CGCTTG-CCTAA-CAGGTCTGC-TTC-----TAACTGTCCG---------ATAACAA--AGGAC-AA-
-------GG-TCC--ATTAAC-AACGA--C-
CTTCATGACTTACTTGACCTCATCCCCTAGTAACTGCGAGTGAAGCGGGAAAAGCTCAAATTTTA
AATCT-G-GCG-GTCT-CCTGGCCGTCCGAGTTGTAATCTGGAGAAGCGTTTT-CCGCGTCG-
GACCGTGCACAAGTCTCCTGGAACGGAGCGTCATAGAGGGTGAGAATCCCG-
TCCATGGCACGG-ACGCGCCGATGC-
TTTGTGATACGCTCTCGAAGAGTCGAGTTGTTTGGGAATGCAGCTCAAAATGGGTGGTAAATTC
CATCTAAAGCTAAATATTGGCGAGAGACCGATAGCGAACAAGTACCGTGAGGGAAAGATGAAA
AGCACTTTGGAAAGAGAGTTAAACAGTACGTGAAATTGTTGAAAGGGAAACGCTTGAAGTCAG
TCGCGTCCGGCAGAACTCAGCCTTGC----TTCGGCCTGGTGTACTTTCTGT-C---
GGACGGGTCAACATCAG-TTTT-GAC-
CGGTGGAGAAAGGCGAGGGGAATGTAGCGCCGTTTCGGCGGCGTG-TTATAGC-CCCCCGTC-
AGAT-ACACCGGCTGGGACTGAGGA-CCGCAGCACGCCT------TT-T-TGGCC-------
GGGGG----------------------TT---CG-CCCCACG-T-ACCGTGCTTAGGAT-
GTTGGCATAATGGCTTTAAGCGACCCGTCTTGAAACACGGACCAAGGAGTCCAACATGCTTGCG
AGTGTTCGGGTGGAAAACCCTTGCGCGTAATGAAAGTGACA-GTTGGGAA-----CCTC-------
-----------CGCAA--GGGGGTGCACCGACGCCCGGCCCTGACG-----
TTCTCTGACGGTGCTGCGGTAGAGCAAGTATGCTGGG----
ACCCGAAAGATGGTGAACTATGCCTGAATAGGGCGAAGCCAGAGGAAACTCTG-
GTGGAGGCTCGTAGCGATTCTGACGTGCAAATCGATCGTCAAATTTGGG------------------
----

>Phellinus_piceicola_LWZ_20190921-5
ATGAATTTGAA-------AG-GCGGAGG------------------------------CTTGATGCTG--
GCGCG---------------GAAACG--CGGA-TGTG-CACGGC------------------------------
---CCT-CGTC-----CTCAATCC--A--TTC-----------------AA-CCCC--TGTGCA-
CCTTATGG---GAG--------------------
--------------------------------------TTACTAGT----------------C-----------------
----

----------------------------GGCAG--

------------TAATGTTAGTA---------------GTCGAAA-------GACAAAAGC-----------
-------------------------------TTACGC-ATTGTCGGCGAACAC-TTCAA---------CTA-
TTAT-------------CA---ACCTTTG---TTGTCATGTAGAAT-G-----T-AAT-GC-T-CC-TT---
-GTGGGCGAAAT-G-----
AAATACAACTTTCAACAACGGATCTCTTGGCTCTCGCATCGATGAAGAACGCAGCGAAATGCGA
TAAGTAATGTGAATTGCAGAATTCAGTGAATCATCGAATCTTTGAACGCACCTTGCGCCCCTTGG
-TATTCCGAGGGGCATGCCTGTTTGAGTGTCATGTTAATCTCAACTCCC--------TTG-TTTCTT-
----------------------AACTC--------GGGTGTTGGAC-TTGGAGG-T----------
TGCTGGC-CCCA----------------------TTTGTGGTCGGCTCTTCTTAAATGCATTAGC-
TGGGCTTTGG----------------C-TC---GCGTTGA-----------ACGGTGTAATAGTAATCT--
----------TACA-TTCACCT-----GAG-CGCTTG-CCTAA-CAAGTCTGC-TTC-----
TAATCGTCTT------------------CGGAC-AA--------GG------CCTTGT-TGC----C-

TTCACTTGACATCTTGACCTCATCCCCTAGTAACTGCGAGTGAAGAGGGAAGAGCTCAAATTTA
AAATCT-G-GCG-GGC--TCTGTCCGTCCGAGTTGTAATCTGGAGAAGCGTTTT-CCGCGTCG-
GACCGTGCATAAGTCTCCTGGAACGGAGCGTCATAGAGGGTGAGAATCCCG-
TCCATGGCACGG-AC-CGCCGATGC-
TTTGTGATACGTTTTCAAAGAGTCGAGTTGTTTGGGAATGCAGCTCAAAATGGGTGGTAAATTCC
ATCTAAAGCTAAATATTGGCGAGAGACCGATAGCGAACAAGTACCGTGAGGGAAAGATGAAAA
GCACTTTGGAAAGAGAGTTAAACAGTACGTGAAATTGTTGAAAGGGAAACGCTTGAAGTCAGTC
GCGTCCGACGGAACTCAGCCTTGC----TTCGGCCTGGTGTACTTTCTGT-C---
GGACGGGTCAACATCAG-TTTT-GAC-
CGGTGGAGAAAGGTAGGGGGAATGTAGCGCTGTTTCGGCGGCGTG-TTATAGC-CCCTTGCT-
GTAT-GCATCGGTTGGGACTGAGGA-CCGCAGCACGCCT------TT-T-TGGCC-------
GAGGG----------------------TT---CG-CCCTACG-T-ACCGTGCTTAGGAT-
GTTGGCATAATGGCTTTAAGCGACCCGTCTTGAAACACGGACCAAGGAGTCCAACATGCTTGCG
AGTGTTCGGGTGGAAAACCCTTGCGCGTAATGAAAGTAACA-GTTGGGAA-----CCTC-------
-----------CGCAA--GGGGGTGCACCGACGCCCGGCCCTGAAG-----
TTCTCTGACGGTGCTGCGGTAGAGCAAGTATGCTGGG----
ACCCGAAAGATGGTGAACTATGCCTGAATAGGGCGAAGCCAGAGGAAACTCTG-
GTGGAGGCTCGTAGCGATTCTGACGTGCAAATCGATCGTCAAATTTGGGTTGGTGTGCC--AT--
-CGAGGATAACGTAAGGGAAAAT-
AATGATGTTACCTTACTAGTAGTGTCGTCCAAGTGCTCGTGCCAGAAGACTCGGTAAGGCGAGA
GACGCAAACGTTATTCGTCTTGATCAGGCGTAAAGGGTTTGTAGGCGGCTTTAAGTATCCAA-
TTTTAAGTTTACTATTAT--------------------------------AATTTTATTT---------------
----

TTAATATTGTTATAATATTTATTAAAGGATCAATTAAAGCTAGAATCTAACAGGGGTTA----
AAACAATGCTTAAA--GTAGGAGTACGAACCATAGATATTAAGTAGAGTGT-
TAAGGGCGAAGGC-
TTTTATCCAATAAAGATTGACGCTGAGAAACGAAGGTGAGGACAGAAAAAAGGATTAGAGACC
CTTGTATCCCTCACTGTCAACGATGAATGGTAGTTACTAGGGAGAA-----------------------
----
ATAACCTAGGGACGATGTTAACACGAAAACCATTCCGCCTTGTGAGTACGACTGCAAAGTTGAA
AACAAAAAAATTAGTCGGTTTCGGAG--CAAACGAAGTGAAGC-ATGTTATTTA-------------
----

ATTTCCGTCGGCTCCACCTCAGCGCCAGTCATTGAATTCTTGGAGGAGTGGGGTTTGGAAAGTC
TTGAAGAGA---CACA---ATCCACGA------
ATCTGACCAAAGTGTTTGTTAACGGTGTGTGGCTAGGAGTGCACCGCGATCCATTGAACCTGGT
TAAGACTATCCGTATGCTTAGGAGAAAAG---
AGGATCTTGAAGCCGAAGTGTCTATTGTTCGTGACATTCGCGAGAAGGAAGTGCGCGTCTACAC

TGACGCAGGCCGTGTACTTCGACCACTCTTCATTGTCGAGGACAACAGGCTCAAGTTAAGGCGT
CAGCATCTGCGATGGCTTCAAGAGAAATGGAGGATC-------GAGACCAGACCTCTT--------
----------------GAAGGAGGGAACGATTCTGGTGGGGAAACGATAGCGGAG---
GAAGAGGATGGAAGGCCTATAGAAAGGGAGGGGGTTAGGATGAAAAAGAAGAGGAAGCCCTA
CAACTGGGCAGCATTGGTCAGAGATGGGGTTATCGAATACCTCGACGCCGAAGAAGAGGAAAC
GGTCATGATTTGCATGACGCCGGACGATCTCGTTGAGTCTCGACTGCTGAAGAAAGGCCTCAAC
CCACG---AGCTGGCGGTGATTTTGATCCTGCTGCTCGGTTGAGGCCATTACCGGCG------
GCGCATACTTGGACGCATTGCGAGATTCATCCTAGCATGATTTTGGGCATTTGCGCCAGTATTAT
ACCGTTCCC
>Phellinus_pomaceus_LWZ_20160908-1
ATGAATTTTGA-----A-AG-GCGAGGG------------------------------CTTGATGCTG--
GCGCG---------------GAGACG--CGGA-CGTG-CACGGC---------------------------
----CCT-TGTC-----CTCAATCC-AA--TTC-----------------AA-CCCC--TGTGGA-
CCTTATGG---GAG-------------------
------------------------------------TTATTAGC----------------C----------------
----

--T---------
---------------------GGCGGGAATG---

-------------TAATGTTAGTA---------------GTCGAGA-------GGCGAAGGC----------
-------------------------------TTACGG-ATCGTCGGTGAACRC-TTCAA-------CTTTA-
TTAT-------------CA---ACCTTTG---TTGTCGTGTAGAAT-G-----C-AAT-GC-T-CC-TC--
--GTGGGCGAAAT-G-----
AAATACAACTTTCAACAACGGATCTCTTGGCTCTCGCATCGATGAAGAACGCAGCGAAATGCGA
TAAGTAATGTGAATTGCAGAATTCAGTGAATCATCGAATCTTTGAACGCACCTTGCGCCCCTTGG
-TATTCCGAGGGGCATGCCTGTTTGAGTGTCATGTTAATCTCAATCCCC--------TTG-
TTTTCTT---------------------AACTC--------GGGCGTTGGAC-TTGGAAG-T----------
TGCTGGC-CCCT---------------------CTCGCCGGTCGGCTCTTCTTAAATGTATTAGC-
TGGGCTTTGG----------------C-TC---GCGTTGA-----------ACGGTGTAATAGTAATCT--
----------TACA-TTCACCT-----GAG-CGCTTG-CCTGA-CGAGTCTGC-TTC-----
TAATCGTCTT------------------CGGAC-AA--------GG-----TCTTTGT-TAC----C-
TTCACTTGACATCTTGACCTCATCCCCTAGTAACTGCGAGTGAAGAGGGAAAAGCTCAAATTTA
AAATCT-G-GCG-GGCT--CTGTCCGTCCGAGTTGTAATCTGGAGAAGCGTTTT-CCGCGTCG-
GACCGTGCACAAGTCTCCTGGAACGGAGCGTCATAGAGGGTGAGAATCCCG-
TCCATGGCACGG-AC-CGCCGATGC-
TCTGTGATACGTTTTCAAAGAGTCGAGTTGTTTGGGAATGCAGCTCAAAACGGGTGGTAAATTCC
ATCTAAAGCTAAATATTGGCGAGAGACCGATAGCGAACAAGTACCGTGAGGGAAAGATGAAAA
GCACTTTGGAAAGAGAGTTAAACAGTACGTGAAATTGTTGAAAGGGAAACGCTTGAAGTCAGTC
GCGTCCGACGGAACTCAGCCTTGC----TTCGGCCTGGTGTACTTTCCGT-C---
GGACGGGTCAACATCAG-TTTT-GAC-
CGGTGGAAAAAGGTGGGGGGAATGTAGCGCCGCTTCGGCGGCGTG-TTATAGC-CCCTCGCT-
GCAT-GCACCGATTGGGACTGAGGA-CCGCAGCACGCCT------TT-T-TGGCC-------

GGGGG----------------------CT---CG-C--

TTGATGTGTC--AT---CGAGGATAACGTAAGGGAAAAT-
AATGATAATACCTTACTAGTAGTGTCGTCCAAGTGCTCGTGCCAGAAGACTCGGTAAGGCGAGA
GACGCAAACGTTATTCGTCTTGATCAGGCGTAAAGGGTTTGTAGGCGGCTTTAAGTATCCAA-
TTTTAATTTTACCATTTT--------------------------------AAGATTATTT---------------
----

TTAATAACGTTAAAATACTTATTAAAGGATCAATTAAAGCTAGAATCTAACGGGGGTTA----
AAACAATGCTTAAA--GTAGGTGTACGAACCATAGATATTAAGTAGAGTGT-
TAAGGGCGAAGGC-
TTTTATCCAATAAAGATTGACGCTGAGAAACGAAGGTGAGGACAGAAAAAAGGATTAGAGACC
CTTGTATCCCTCACTGTCAACGATGAGTGGTAGTTATTAGGGAGAA-----------------------
----
ATAACCTAAAGACGATGTTAACACGAAAACCATTCCGCCTTGTGAGTACGACTGCAAAGTTGAA
AACAAAAAAATTAGTCGGTTTCGGAG--CAAACGAAGTGAAGC-ATGTTATTTAAT-----------
----

>Porodaedalea_alpicola_Cui_12272
----------------------CGGGGG------------------------------CTTGATGCTG--
GCGTG---------------GAAACG--CGCACTGTG-CTCGGC----------------------------
---CTTTCGTG-----CTTAATCC--A--CTC-----------------AA-CACC--TGTGCA-
CCTTATCG--AAG-------------------
------------------------------------TTAGTAGT---------------------------------
--TGGAGCCACCGGGGT----------

-----------TGATTTTGTTAGTA-------------GTGTTTCGA-------CGCGAAATTA--------
-----------TGGTCGGCCT------------TGGCT-GGGATCGGCGAGCAC-TTTGA-------
CTTCA-TCAT-------------AC---ACACTTTAA-TTGTCTTGTAGAAT-G-----T-AAT-GC-T-
CC-TT----GTGGGCGAAAT------
GAAATACAACTTTCAACAACGGATCTCTTGGCTCTCGCATCGATGAAGAACGCAGCGAAATGCG
ATAAGTAATGTGAATTGCAGAATTCAGTGAATCATCGAATCTTTGAACGCACCTTGCGCCCCTTG
G-TATTCCGAGGGGCATGCCTGTTTGAGTGTCATGTTAACATCAAACCCC--------TTG-
CTTGTAA--------------------AGGCTC--------GGGGCTTGGAT-TTGGAGG-T-T------
TGTGCCGGC-CTGC-------------TTGATTG--TCGGTTGTCGGCTCCTCTTAAATGGATTAGC-
TGGACTTTGG----------------T-CT---GCG--TG-----------TCGGTGTAATAG--------
---------TTTA-TTCACC------AAT-CGCTTG-CCTAA-TGGGTCTGC-TTC-----
TAATGGTCTT------------------CGGAC-AA--------GG-TCT---TTAAC-AGC------
CTTCTTTGACTCTTTGACCTCATCCCCTAGTAACTGCGAGTGAAGCGGGAAAAGCTCAAATTTAA
AATCT-G-GCG-GTCT-TATGGCCGTCCGAGTTGTAATCTGGAGAAGTGTTTT-CCGCGTCG-
GACCGTGTACAAGTCTCCTGGAACGGAGCGTCATAGAGGGTGAGAATCCCG-
TCCATGACACGG-AC-TGCCGATGC-
TTTGTGATACACTCTCGAAGAGTCGAGTTGTTTGGGAATGCAGCTCAAAATGGGTGGTAAATTCC
ATCTAAAGCTAAATATTGGCGAGAGACCGATAGCGAACAAGTACCGTGAGGGAAAGATGAAAA
GCACTTTGGAAAGAGAGTTAAACAGTACGTGAAATTGTTGAAAGGGAAACGCTTGAAGTCAGTC
GCGTCTTTCAGAACTCAGCCTTGC----TTCGGCCTGGTGTACTTTCTGT-T---
AGACGGGTCAACGTCAA-TTTC-GAT-
CGGCGGAGAAAGGTTGAGGGAATGTAGCGTTGTTTCGGCGACGTG-TTATAGCGCTCTTCTG-
GTAT-ACGTCGGCTGGGATTGAGGA-CCGCAGCGCGCCT------TTAA-TGGCC-------
AGGGG----------------------TT---CG-CCTCATG-T-ACCGCGCTTAGGAT-
GTTGGCATAATGGCTTTAAGCGACCCGTCTTGAAACACGGACCAAGGAGTCTAACATGCTTGCG
AGTATTCGGGTGGAAAACCCTTGTGCGTAATGAAAGTAAAA-GTTGGGAG-----CCTC-------
-----------CGCGA--GGGGGTGCACCGACGCCCGGCCCTGACG-----
TTCTCTGACGGTGCTGCGGTAGAGCAAGTATGTTGGG----
ACCCGAAAGATGGTGAACTATGCCTGAATAGGGTGAAGCCAGAGGAAACTCTG-
GTGGAGGCTCGTAGCGATTCTGACGTGCAAATCGATCGTCAAATTTGGG-----------------
----

GGTACTGGTGAGTTCGAGGCTGGTATCTCGAAGGACGGCCAGACCCGCGAGCATGCTCTCCTT

GCGTTCACCCTTGGCGTTCGTCAGCTGATCGTCGCTGTCAACAAAATGGACACCACCAAGGTTC
GCCTGCACTATTGGTC---GCATTTTATCCTTTTTTTTCC--
CTTTTTATTTATGAAATCTCTCAGTGGTCCGAGGATCGTTTCAATGAAATTGTCAAAGAGACGTC
CAACTTCATYAAGAAGGTCGGCTACAACCCTAAGGCTGTTGCTTTCGTCCCGATCTCTGGATGG
CATGGTGATAACATGTTGGAGGAATCCCCCAAGTAAATTAAATTA-CTATTAT---
ATTTGTTATCCGT-----GCTAATTTCGCCTGTGCC-
TGCAGCATGACGTGGTACAAGGGCTGGACAAAGGAGACGAAGG------------
CTGGTGTCGTC-
AAGGGCAAGACTCTCCTCGACGCCATCGACGCCATCGAGCCCCCAGTTCGTCCCGAGAACAAG
CCCCTCCGTCTTCCCCTTCAGGATGTGATTTCCGTTGGCTCGTATTCAGCGCCTGTCATCGAGTT
CCTGGAAGAGTGGGGTTTGGAAAGTCTTGAAGAGAATGCTCA---ATCCACGA------
ACCTTACCAAGGTCTTTGTAAATGGCGTATGGCTTGGGGTACACCGTGACCCATTCAATCTGGTG
AAGACCATTCGTACTCTCAGGAGAAGAG---
AGGATATCAGCTGCGAGGTGTCTGTTGTTCGTGATATCCGTGAGAAGGAACTTCGCATCTATAC
AGACGCCGGTCGGGTGCTTCGACCACTCTTCATTGTAGAGGATCACAAATTGAAGTTGAAGCGT
CAGCATCTGCGCTGGCTTCACGAAAAATGGAGAGTT-------
GAGGAAAGGCCTATCGTGAGGCCC------
GAAGAGAGGGAAGAAAGCGAAGTTTCTGAGGGAGAGACAATAGCGGAG---
GAGGAAAACGGCATGCCGATAATGAGGGAAAGGATTAAGATGAAGAAAAAGAGGAAGCCTTT
CACCTGGAGTACTCTGGTGAAAGATGGTGTTATCGAATACCTTGATGCTGAGGAGGAGGAGAC
AGTTATGATTTGCATGACGCCAGACGATCTAGTCGAGTCTCGGCTAATGCGTAAAGGCTTGGAT
CCACG---AGCAGACAGTGACTTTGATCCTGCTGCTCGATTAAAAGCGGTGCCGATG------
GCACACACTTGGACACACTGC---
>Porodaedalea_chrysoloma_Dai_12674
TCGAGTTTTTA-----A-AA-TCGAGGG-------------------------------CTTGATGCTG--
GCGTG---------------GAAACA--CGCACTGTG-CTCGGC-----------------------------
---CTTTCGTG-----CTTAATCC--A--CTC-----------------AA-CACC--TGTGCA-
CCTTATCG---AAG-------------------
-------------------------------------TTAGTAGT----------------C------TTTCCTC--
--CTTAGTTGGAGCCGCCGGGGT--------

-------------TGACTTTGTTAGTA-------------GTGTTTCGA-------CGCGAAAGCATA---
--------------TGGTCGGCCT------------TGGCT-GGGATCGGCGAACAC-TTTGA-------
CTTCA-TTAT-------------AC---ACACTTTAA-TTGTCTTGTAGAAT-G-----T-AAT-GC-T-
CC-TT----GTGGGCGAAAT------
GAAATACAACTTTCAACAACGGATCTCTTGGCTCTCGCATCGATGAAGAACGCAGCGAAATGCG
ATAAGTAATGTGAATTGCAGAATTCAGTGAATCATCGAATCTTTGAACGCACCTTGCGCCCCTTG
G-TATTCCGAGGGGCATGCCTGTTTGAGTGTCATGTTAACATCAAACCCC--------TTG-
CTTGTAA--------------------AGGCTC--------GGGGCTTGGAT-TTGGAGG-T-TG---
TGCTGGCTGGC-CTAC-------------TTCATTG--

TCGGTTGTCGGCTCCTCTTAAATGGATTAGC-TGGACTTTGG----------------T-CT---GCG-
-TG-----------TCGGTGTAATAG-------------------TTTA-TTCACC------AAT-CGCTTG-
CCTAA-TGGGTCTGC-TTC-----TAATAGTCTT-------------------TGGAC-AA--------GG-
TC----TTAAC-AGC-------
CTTCTTGACTCTTTGACCTCATCCCCTAGTAACTGCGAGTGAAGCGGGAAAAGCTCAAATTTAAA
ATCT-G-GCG-GTCT-TATGGTCGTCCGAGTTGTAATCTGGAGAAGTGTTTT-CCGCGTCG-
GACCGTGTACAAGTCTCCTGGAACGGAGCGTCATAGAGGGTGAGAATCCCG-
TCCATGACACGG-AC-CGCCGATGC-
TTTGTGATACACTCTCGAAGAGTCGAGTTGTTTGGGAATGCAGCTCAAAATGGGTGGTAAATTCC
ATCTAAAGCTAAATATTGGCGAGAGACCGATAGCGAACAAGTACCGTGAGGGAAAGATGAAAA
GCACTTTGGAAAGAGAGTTAAACAGTACGTGAAATTGTTGAAAGGGAAACGCTTGAAGTCAGTC
GCGTCTTTCAGAACTCAGCCTTGC----TTCGGCCTGGTGTACTTTCTGT-T---
AGACGGGTCAACGTCAA-TTTC-GAT-
CGGCGGAGAAAGGTTGAGGGAATGTAGCGTTGTTTCGGCGACGTG-TTATAGC-CCTTTTCT-
GTAT-ACGTCGGCTGGGATTGAGGA-CCGCAGCGCGCCT------TT-A-TGGCC-------
AGGGG-----------------------TT---CG-CCCCATG-T-ACCGCGCTTAGGAT-
GTTGGCATAATGGCTTTAAGCGACCCGTCTTGAAACACGGACCAAGGAGTCTAACATGCTTGCG
AGTATTCGGGTGGAAAACCCTTGTGCGTAATGAAAGTAAAA-GTTGGGAG-----CCTC-------
-----------CGCGA--GGGGGTGCACCGACGCCCGGCCCTGACG-----
TTCTCTGACGGTGCTGCGGTAGAGCAAGTATGTTGGG----
ACCCGAAAGATGGTGAACTATGCCTGAATAGGGTGAAGCCAGAGGAAACTCTG-
GTGGAGGCTCGTAGCGATTCTGACGTGCAAATCGATCGTCAAATTTGGG------------------
----

TCTCCTCGACGCCATCGACGCCATCGAGCCCCCAGTTCGTCCCGAGAACAAGCCCCTCCGTCTT
CCTCTTCAGGATGTGATTTCCGTCGGCTCGTATTCAGCGCCTGTCATCGAGTTCCTGGAGGAGTG
GGGTTTGGAAAGTCTTGAAGAGAATGCTCA---ATCCACGA------
ACCTTACCAAGGTCTTTGTTAATGGCGTATGGCTTGGGGTGCACCGTGACCCATTCAATCTGGTG
AAGACCATTCGTACTCTCAGGAGAAGAG---
AGGATATCAGCTGCGAGGTGTCTGTTGTTCGTGATATCCGTGAGAAGGAACTTCGCATCTATAC
AGACGCCGGTCGGGTGCTTCGACCACTCTTCATTGTAGAGGATCACAAATTGAAGTTGAAGCGT

CAGCATCTGCGCTGGCTTCACGAAAAATGGAGAGTG-------
GAGGAAAGGCCTATCGTGAGGCCC------
GAAGAGAGGGAAGAAGGCGAAGTTTCTGAGGGAGAGACAATAGCGGAG---
GAGGAAGACGGCATGCCGATAATGAGGGAAGGGATTAAGATGAAGAAAAAGAGGAAGCCTTT
CACCTGGAGCACTCTGGTGAAAGATGGTGTTATTGAATACCTTGATGCTGAGGAGGAGGAGAC
AGTTATGATTTGCATGACGCCAGACGATCTAGTCGAGTCTCGGCTAATGCGTAAAGGCTTGGAT
CCACG---AGCAGACAGTGACTTTGATCCTGCTGCTCGATTAAAAGCGGTGCCGATG------
GCACACACTTGGACACACTGCGAGATCCATCCTAGCATGATTTTAGGCATCTGTGCTAGTATCAT
TCCGTTCCC
>Porodaedalea_laricis_LWZ_20190724-9
TCGAGTTTTTA-----A-AA-TCGGGGG------------------------------CTTGATGCTG--
GCGTG---------------GAAACA--CGCACTGTG-CTCGGC----------------------------
---CTT-CGTG-----CTTAATCC--A--CTC-----------------AA-CACC--TGTGCA-
CCTTATCG---AAG-------------------
------------------------------------TTAGTAGT----------------C------TTTCCTC--
--CTTAGTTGGAGCCGCCGAGGT--------

-------------TGACTTTGTTGGTA-------------GTGTTTCGA-------CGCGAAAGCATA---
--------------TAGTCGGCCT------------TGGCT-GGGATTGACGAACAC-TTTGA-------
CTTCA-TCAT-------------AC---ACACTTTAA-TTGTCTTGTAGAAT-G-----T-AAT-GC-T-
CC-TT----GTGGGCGAAAT------
GAAATACAACTTTCAACAACGGATCTCTTGGCTCTCGCATCGATGAAGAACGCAGCGAAATGCG
ATAAGTAATGTGAATTGCAGAATTCAGTGAATCATCGAATCTTTGAACGCACCTTGCGCCCCTTG
G-TATTCCGAGGGGCATGCCTGTTTGAGTGTCATGTTAACATCAAACCCC--------TTG-
CTTGTAA--------------------AGGTTC--------GGGGCTTGGAT-TTGGAGG-T-T------
TATGCCGGC-CTGC-------------TTCATTG--TCAGTTGTCGGCTCCTCTTAAATGGATTAGC-
TGGACTTTGG----------------T-CT---GCG--TG-----------TCGGTGTGATAG---------
---------TTTA-TTCACC------AAT-CGCTTT-CCTAA-TGGGTCTGC-TTC-----
TAATGGTCTT------------------CGGAC-AA--------GG-TC----TTAAC-AGC-------
CTTCTTGACTCTTTGACCTCATCCCCTAGTAACTGCGAGTGAAGCGGGAAAAGCTCAAATTTAAA
ATCT-G-GCG-GTCT-TATGGCCGTCCGAGTTGTAATCTGGAGAAGTGTTTT-CCGCGTCG-
GACCGTGTACAAGTCTCCTGGAACGGAGCGTCATAGAGGGTGAGAATCCCG-
TCCATGACACGG-AC-CGCCGATGC-
TTTGTGATACACTCTCGAAGAGTCGAGTTGTTTGGGAATGCAGCTCAAAATGGGTGGTAAATTCC
ATCTAAAGCTAAATATTGGCGAGAGACCGATAGCGAACAAGTACCGTGAGGGAAAGATGAAAA
GCACTTTGGAAAGAGAGTTAAACAGTACGTGAAATTGTTGAAAGGGAAACGCTTGAAGTCAGTC
GCGTCTTTCAGAACTCAGCCTTGC----TTCGGCCTGGTGTACTTTCTGT-T---
AGACGGGTCAACGTCAA-TTTC-GAT-
CGGCGGAGAAAGGTTGAGGGAATGTAGCGTTGTTTCGGCGACGTG-TTATAGC-CCTCTTCT-
GTAT-ACGTCGGTTGGGATTGAGGA-CCGCAGCGCGCCT------TT-A-TGGCC-------

AGGGG----------------------TT---CG-CCCCATG-T-ACCGCGCTTAGGAT-
GTTGGCATAATGGCTTTAAGCGACCCGTCTTGAAACACGGACCAAGGAGTCTAACATGCTTGCG
AGTATTCGGGTGGAAAACCCTTGTGCGTAATGAAAGTAAAA-GTTGGGAG-----CCTC-------
-----------CGCGA--GGGGGTGCACCGACGCCCGGCCCTGACG-----
TTCTCTGACGGTGCTGCGGTAGAGCAAGTATGTTGGG----
ACCCGAAAGATGGTGAACTATGCCTGAATAGGGCGAAGCCAGAGGAAACTCTG-
GTGGAGGCTCGTAGCGATTCTGACGTGCAAATCGATCGTCAAATTTGGGTTGGTGTGTC--AT--
-CGAGGATAACGTAAGGGAAAAT-
AATGATACCACCTTACTATTAGTGTCGTCCAAATGCTCGTGCCAGAAGACTCGGTAAGGCGAGA
GACGCAAACGTTATTCGTCTTGATCAGGCGTAAAGGGTTTGTAGGCGGCTTTAAGTATCCAA-
TTTTAAGTTTACTATTAT-------------------------------AATGCTATAA--------------
----

GTGTTATAATATTTATTAAAGGGTCAATTAAAGCTAGAATCTAACAGAGGTTA----
AAACAATACTTAAG--GTAGGTGTACTAACCATAAATAATAAGCAGAGTGT-
TAAGGGCGAAGGC-
ATTTATCCACTGAAGATTGACGCTGAGAAACGAAGGTGAGGACAGAAAAAAGGATTAGAGACC
CTTGTATCCCTCACTGTCAACGATGAATGGTAGTTACTAGGGAGAA-----------------------
----
ATAACCTAGAGACGATGTTAACACGAAAACCATTCCGCCTTGTGAGTACGACTGCAAAGTTGAA
AACAAAAAAATTAGTCGGTTTCGGAG--CAAACGAAGTGAAGC-ATGTTATTTAT------------
----

ATTTCCGTTGGCTCGTATTCAGCGCCTGTCATCGAGTTCCTGGAAGAGTGGGGTTTGGAAAGTCT
TGAAGAGAATGCTCA---ATCCACGA------
ACCTTACCAAGGTCTTTGTTAATGGCGTATGGCTTGGGGTGCACCGTGACCCATTCAATCTGGTG
AAGACCATTCGTACTCTCAGGAGAAGAG---
AGGACATCAGCTGCGAAGTGTCTGTTGTTCGTGATATCCGTGAGAAGGAACTTCGCATCTATAC
AGACGCCGGTCGGGTGCTTCGACCACTCTTCATTGTAGAGGATCACAAATTGAAGTTGAAGCGT
CAGCATCTGCGCTGGCTTCACGAAAAATGGAGAGTG-------
GAGGAAAGGCCTATCGTGAGGCCT------
GAAGAGAGGGAAGAAGGCGAAGTTTCGGAGGGAGAGACAATAGCGGAG---
GAGGAAGACGGCATGCCGATAATGAGGGAAGGGATTAAGATGAAGAAAAAGAGGAAGCCTTT
CACCTGGAGTACTCTGGTGAAAGATGGTGTTATCGAATACCTTGATGCTGAGGAGGAGGAGAC
AGTTATGATTTGCATGACGCCAGACGATCTAGTCGAGTCTCGGCTAATGCGTAAAGGCTTGGAT
CCACG---AGCAGACAGTGACTTTGACCCTGCTGCTCGATTAAAAGCGGTGCCGATG------
GCACACACTTGGACACACTGCGAGATCCATCCTAGCATGATTTTAGGCATCTGTGCTAGTATCAT
TCCGTTTCC
>Porodaedalea_mongolica_Dai_19231

TCGAGTTTTTA-----A-AA-TCGGGGG------------------------------CTTGATGCTG--
GCGTG---------------GAAACA--CGCACTGTG-CTCGGC----------------------------
---CTT-CGTG-----CTTAATCC--A--CTC-----------------AA-CACC--TGTGCA-
CCTTATCG--AAG------------------
------------------------------------TTAGTAGT----------------C------TTTCCTC--
---CTTAGTTGGAGCCGCCGAGGT--------

-------------TGACTTTGTTAGTA-------------GTGTTTCGA-------CGCGAAAGCATA---
--------------TAGTCGGCCT------------TGGCT-GGGATTGACGAACAC-TTTGA-------
CTTCA-TCAT-------------AC---ACACTTTAA-TTGTCTTGTAGAAT-G-----T-AAT-GC-T-
CC-TT----GTGGGCGAAAT------
GAAATACAACTTTCAACAACGGATCTCTTGGCTCTCGCATCGATGAAGAACGCAGCGAAATGCG
ATAAGTAATGTGAATTGCAGAATTCAGTGAATCATCGAATCTTTGAACGCACCTTGCGCCCCTTG
G-TATTCCGAGGGGCATGCCTGTTTGAGTGTCATGTTAACATCAAACCCC--------TTG-
CTTGTAA--------------------AGGTTC--------GGGGCTTGGAT-TTGGAGG-T-T------
TATGCCGGC-CTGC-------------TTCATTG--TCAGTTGTCGGCTCCTCTTAAATGGATTAGC-
TGGACTTTGG----------------T-CT---GCG--TG-----------TCGGTGTGATAG---------
---------TTTA-TTCACC------AAT-CGCTTT-CCTAA-TGGGTCTGC-TTC-----
TAATGGTCTT------------------CGGAC-AA--------GG-TC----TTAAC-AGC-------
CTTCTTGACTCTTTGACCTCA-
CCCCTAGTAACTGCGAGTGAAGCGGGAAAAGCTCAAATTTAAAATCTCG-GCG-GTCT-
TATGGCCGTCCGAGTTGTAATCTGGAGAAGTGTTTT-CCGCGTCG-
GACCGTGTACAAGTCTCCTGGAACGGAGCGTCATAGAGGGTGAGAATCCCG-
TCCATGACACGG-AC-CGCCGATGC-
TTTGTGATACACTCTCGAAGAGTCGAGTTGTTTGGGAATGCAGCTCAAAATGGGTGGTAAATTCC
ATCTAAAGCTAAATATTGGCGAGAGACCGATAGCGAACAAGTACCGTGAGGGAAAGATGAAAA
GCACTTTGGAAAGAGAGTTAAACAGTACGTGAAATTGTTGAAAGGGAAACGCTTGAAGTCAGTC
GCGTCTTTCAGAACTCAGCCTTGC----TTCGGCCTGGTGTACTTTCTGT-T---
AGACGGGTCAACGTCAA-TTTC-GAT-
CGGCGGAGAAAGGTTGAGGGAATGTAGCGTTGTTTCGGCGACGTG-TTATAGC-CCTCTTCT-
GTAT-ACGTCGGTTGGGATTGAGGA-CCGCAGCGCGCCT------TT-A-TGGCC-------
AGGGG----------------------TT---CG-CCCCATG-T-ACCGCGCTTAGGAT-
GTTGGCATAATGGCTTTAAGCGACCCGTCTTGAAACACGGACCAAGGAGTCTAACATGCTTGCG
AGTATTCGGGTGGAAAACCCTTGTGCGTAATGAAAGTAAAA-GTTGGGAG-----CCTC-------
-----------CGCGA--GGGGGTGCACCGACGCCCGGCCCTGACG-----
TTCTCTGACGGTGCTGCGGTAGAGCAAGTATGTTGGG----
ACCCGAAAGATGGTGAACTATGCCTGAATAGGGCGAAGCCAGAGGAAACTCTG-
GTGGAGGCTCGTAGCGATTCTGACGTGCAAATCGATCGTCAAATTTGGG------------------
----

GAGGATCGTTTCAATGAAATTGTCAAAGAGACGTCCAACTTCATCAAGAAGGTCGGCTACAACC
CTAAGGCTGTTGCTTTCGTCCCGATCTCTGGTTGGCATGGTGATAACATGTTGGAGGAATCCCCC
AAGTAAGTTAATTTT-CTATTAT---ATTTGTTATCCGC-----TCTAATTTCGCCTGTGCC-
TGCAGCATGACGTGGTACAAGGGCTGGACAAAGGAGACGAAGG------------
CTGGTGTCGTC-
AAAGGCAAGACTCTCCTCGACGCCATCGACGCCATCGAGCCCCCAGTTCGTCCCGAGAACAAG
CCCCTCCGTCTTCCCCTTCAGGATGTGATTTCCGTTGGCTCGTATTCAGCGCCTGTCATCGAGTT
CCTGGAAGAGTGGGGTTTGGAAAGTCTTGAAGAGAAYGCTCA---ATCCACGA------
ACCTTACCAAGGTCTTTGTTAATGGCGTATGGCTTGGGGTGCACCGTGACCCATTCAATCTGGTG
AAGACCATTCGTACTCTCAGGAGAAGAG---
AGGACATCAGCTGCGAAGTGTCTGTTGTTCGTGATATCCGTGAGAAGGAACTTCGCATCTAYAC
AGACGCCGGTCGGGTGCTTCGACCACTCTTCATTGTAGAGGATCACAAATTGAAGTTGAAGCGT
CAGCATCTGCGCTGGCTTCACGAAAAATGGAGAGTR-------
GAGGAAAGGCCTATCGTGAGGCCC------
GAAGAGAGGGAAGAAGGCGAAGTTTCRGAGGGAGAGACAATAGCGGAG---
GAGGAAGACGGCATGCCGATAATGAGGGAAGGGATTAAGATGAAGAAAAAGAGGAAGCCTTT
CACCTGGAGTACTCTGGTGAAAGATGGTGTTATCGAATACCTTGATGCTGAGGAGGAGGAGAC
AGTTATGATTTGCATGACGCCAGACGATCTAGTCGAGTCTCGGCTAATGCGTAAAGGCTTGGAT
CCACG---AGCAGACAGTGACTTTGACCCTGCTGCTCGATTAAAAGCGGTGCCGATG------
GCACACACTTGGACACACTGC---
>Sanghuangporus_alpinus_Cui_12444

----------------------CA-TGTG-CACTGC---------------------------------CTT-
CGCG-----CTCAAATCCAA--CTC----------------AAA-CCCC--TGTGCA-CCTATATA----
--------------------------------TATCGCGAGTCGAAG--------------------------------
---------------------------TTAGTAGT-----------------CTGAG-----GTCT-------------
------------------------------------TGTAAGTAATTGGTAGGAAGGTGAA---------------
----

--------------AGCGGGTGT--
-----------------------------------TACTCGTTAGGTA--------------------------------
----
---------------------------------------GCCCTTCGAA-------AGTGAAAGC------------
---------------------------------GAGT-GCGTCGGGTGAAGACTTTCGG-----

CTTGTTGTTTAC-------------AA---ACACCTTATATTGTCTCTGTGAAT-G-----T-AAT-GC-
T-CC-TC----GTGGGCGAAAA------
TAAATACAACTTTCAACAACGGATCTCTTGGTTCTCGCATCGATGAAGAACGCAGCGAAATGCG
ATAAGTAATGTGAATTGCAGAATTCAGTGAATCATCGAATCTTTGAACGCACCTTGCGCCCCTTG
G-TATTCCGAGGGGCATGCCTGTTTGAGTGTCATGTTAATCTCAAACCGC--------TCG-
TCTTTCTTAACT---------TGAA--GGGCTT--------ACGGTTTGGAC-TTGGAGG-T-T------
ACTGCTGGC-GCCTTTC-------------------GAGGGGGTCGGCTCCTCTTAAATACATTAGC-
TGGGCTTTGG----------------C-TC---GCG-TTT-----------ACGGTGTAATAGTTGAT---
----------TCCG-TTCACC---AACGAG-CGCTTG-CCTGA-CGAGCTTGC-TTC-----
TAGTCGTC---
---------------------------------------GAAGAGCTCAAATTTAAAATCT-G-GCG-
GCCT-TCTGGACGTCCGAGTTGTAGTCTGGAGAAGTGTTAT-CCGCGTCG-
GACCGTGTACAAGTCTCCTGGAACGGAGCGTCGTAGAGGGTGAGAATCCCG-
TCCATGACACGG-AC-GCCCGATGC-
TATGTGAGGCACTCTCAAAGAGTCGAGTTGTTTGGGAATGCAGCTCAAAATGGGTGGTAAATTC
CATCTAAAGCTAAATATTGGCGAGAGACCGATAGCGAACAAGTACCGTGAGGGAAAGATGAAA
AGCACTTTGGAAAGAGAGTTAAACAGTACGTGAAATTGTTGAAAGGGAAACGCTTGAAGTCAG
TCGCGTCCCGCGGAACTCAGCCTGGT----TTCGACCTGGTGTACTTTCCAT-GT--
GGACGGGTCAACATCAA-TTTC-GGT-
CGGTGGACAAGGGCGAGGGGAATGTAGCGTTGCTTCGGCGACGTG-TTATAGC-CCCCCGTC-
GTAT-ACACTGGTTGGGATTGAGGA-CCGCAGCACGCCC------TT-G-TGGCC-------
GGGGG----------------------TT---CG-CCCCACG-T-AACGTGCTTAGGAT-
GTTGGCATAATGGCTTTAAGCGACCCGTCTTGAAACACGGACCAAGGAGTCTAACATGCTTGCG
AGTGTTCGGGTGGAAAACCCTTGCGCGTAATGAAAGTGAAA-GTTGGGCA-----CCTC-------
-----------CGCGA--GGAGGTGCACCGACGCCCGGCCCTGACG-----
TTCTCTGACGGTGCCGCGGTAGAGCACGTATGTTGGG----
ACCCGAAAGATGGTGAACTATGCCTGAATAGGGCGAAGCCAGAGGAAACTCTG-
GTGGAGGCTCGTAGCGATTCTGACGTGCAAATCGATCGTCAAATTTGGG------------------
----

CAAACTCGCGAGCACGCTCTTTTGGCCTTCACTCTCGGTGTACGTCAGCTTATCGTCGCGGTCAA
CAAAATGGATACCACCAAGGTTAGATTTGATCAATGAT-
CAATCACGAGCCAAAAGCTGATATA-TCTTTCA----------
CTTGTAGTGGTCTGAAGAGCGATTCAACGAAATCGTCAAGGAAACTTCCAACTTCATCAAGAAG
GTCGGCTACAACCCCAAGGCTGTACCTTTCGTCCCAATTTCCGGTTGGCACGGCGACAACATGC
TTGAGGAATCAAGCAAGTACGTATGACCA-TTAACTA---------CAGATAG-----

CTGTACTGACTTCTTGTT-TGCAGTATGACGTGGTACAAGGGCTGGACCAAGGAGACGAAGG--
----------CTGGTGTTGTG-AAGGGGAAGACTCTCCTCGACTCCAAC----------------------
----
ATTTCCGTGGGCTCTTACTCAGCCCCCGTGCTCGAGTTCTTGGAAGAATGGGGCTTGGAAAATTT
GGAGGACAACGCACA---GTCGCCAA------
ACCTAACCAAGGTGTTCGTTAACGGTGTTTGGCTTGGGGTGCACCGTGACCCGTTTAGTCTGGT
AAACACGCTCCGTAATCTTCGGCGAAAGG---
GCGATCTTGGCAGCGAAGTATCTATTGTACGGGATATCCGTGAAAAGGAGGTTCGCGTATATAC
GGACGCGGGTCGCGTGCTTCGACCTCTATTCATTGTCGAGAAGCAGCATCTGAAGTTGAAACGT
CAGCATCTTCGATGGCTTCAAGAGAAATGGAGAATT-------
GAAGAGAGACCTATCGAAAAGCCACCTGTAGATGACGAGCAGAACGTGGAGGAATCGGAAGG
AGAGACAATAGCCGAG---
GAAGAGGATGGCAAACCTATAATCCGAGACGGAATTAGGATGAAGAAGAAGCGGAAGCCTTTC
AATTGGGGCACTTTGGTTTCAGACGGCGTCATTGAATACCTTGACGCTGAAGAGGAAGAGACA
GTCATGATTTGTATGACTCCGGATGATTTGGTCGAGTCGCGGTTAATGAGTCGCGGACTCGATC
CCAG---AGCCGAT--

>Sanghuangporus_quercicola_Dai_21763
----------------------CGGGGG-------------------------------CTTGCTGCTG--
GCGCG---------------GATTTG--CGCA-TGTG-CACGGT------------------------------
--CTT-CGCG-----CTCAAATCCAA--CTCTC--------------AAA-CCCC--TGTGCA-
CCTTATTATTAT-------------------------------TATCGCGAGTCGAGG----------------
--TTAGTAGT----------------
CTGAGGGTCTGCCT--
TGTAAGTAATTAGTAGGAGGGCGAA--

--AGCGAGCGT---------

GGCTCGTTAGGTA---

---ACCCTTCGAA-------AATGAAAGC---
GGGGC-GCGTCGGGTGAAGAC-TTCGG-----CTTGTCG-TTATTACAAAA------AA---
ACCCCTTATACTGTCTTTGTGAAT-G-----T-AAT-GC-T-CC-TC----GTGGGCGAAAA------
TAAATACAACTTTCAACAACGGATCTCTTGGCTCTCGCATCGATGAAGAACGCAGCGAAATGCG
ATAAGTAATGTGAATTGCAGAATTCAGTGAATCATCGAATCTTTGAACGCACCTTGCGCCCCTTG
G-TATTCCGAGGGGCATGCCTGTTTGAGTGTCATGTTTATCTCAAACCGC--------TCG-
TCTTTCTTAAT----------TGAA--GGGCTT--------GAGGTTTGGAC-TTGGAGG-T-TT---
TTACTGCTGGC-GCCTCTTT----------CTCGAG--
AGGGGGGTCGGCTCCTCTTAAATACATTAGC-TGGGCTTTGG----------------C-TC---
GCG-TTT-----------ACGGTGTAATAGTTGAT-------------TCCG-TTCACC---AACGAG-
CGCTTG-CCTGA-GGAGCTCGC-TTC-----TAGCCGTCCGCGTCGT------------CGGAC-AA-
-------GGAGTTT-GTTTGT-
TACCTCCTTCATCTTTGACACCTTGACCTCATCCCCTAGTAACTGCGAGTGAAGCGGGAAGAGCT
CAAATTTAAAATCT-G-GCG-CCCT-TGTGGACGTCCGAGTTGTAGTCTGGAGAAGTGTTAT-

```
CCGCGTCG-GACCGTGCACAAGTCTCCTGGAACGGAGCGTCATAGAGGGTGAGAATCCCG-
TCCATGGCACGG-AC-
GCCCGATGCTTATGCGAGGCACTCTCAAAGAGTCGAGTTGTTTGGGAATGCAGCTCAAAATGGG
TGGTAAATTCCATCTAAAGCTAAATATTGGCGAGAGACCGATAGCGAACAAGTACCGTGAGGG
AAAGATGAAAAGCACTTTGGAAAGAGAGTTAAACAGTACGTGAAATTGTTGAAAGGGAAACGC
TTGAAGTCAGTCGCGTCCCGTGGAACTCAGCCTGGT----TTCGGCCTGGTGTACTTTCCAT-
TGCGGGACGGGTCAACGTCAA-TTTC-GGC-
CGGTGGACAAGGGCGAGGGGAATGTAGCGTTGCTTCGGCGACGTG-TTATAGC-CCCGCGTC-
GTAT-ACGCCGGCCGGGATTGAGGA-CCGCAGCACGCCC------TT-G-TGGCC-------
GGGGGGG-----------------TTCGCG---CC-CCCCACG-T-AACGTGCTTAGGAT-
GTTGGCATAATGGCTTTAAGCGACCCGTCTTGAAACACGGACCAAGGAGTCTAACATGCCTGCG
AGTGTTCGGGTGGAAAACCCTTGCGCGTAATG-AAGTGAAA-GTTGGGAA-----CCTC-------
-----------CGCGA--GGGGGTGCACCGACGCCCGGCCCTGACG-----
TTCTCTGACGGTGCCGCGGTAGAGCATGTATGTTGGG----
ACCCGAAAGATGGTGAACTATGCCTGAATAGGGCGAAGCCAGAGGAAACTCTG-
GTGGAGGCTCGTAGCGATTCTGACGTGCAAATCGATCGTCAAATTTGGG------------------
-------------------------------------------------------------------
-------------------------------------------------------------------
-------------------------------------------------------------------
-------------------------------------------------------------------
-------------------------------------------------------------------
-------------------------------------------------------------------
-------------------------------------------------------------------
-------------------------------------------------------------------
-------------------------------------------------------------------
GGTACTGGTGAATTCGAGGCTGGTATCTCCAAGGATGGTCAAACTCGCGAGCACGCTCTTTTGG
CCTTCACTCTCGGTGTACGTCAGCTTATCGTCGCGGTCAACAAAATGGATACCACCAAGGTTAG
ATTTGATCAATGAT-CAATCATGAGTCAAAAGCTGATATA-ACCTTAA----------
CTTGTAGTGGTCCGAAGAGCGATTCAATGAAATCGTCAAGGAAACTTCCAACTTCATCAAGAAG
GTCGGCTACAACCCCAAGGCTGTACCTTTCGTCCCAATTTCCGGCTGGCATGGCGACAACATGC
TTGAGGAATCAAGCAAGTACGTATGACCA-TTAACTA---------CAGATGG-----
CTGTACTGACTTCTTATT-TGCAGTATGACGTGGTACAAGGGCTGGACGAAGGAGACGAAGG--
----------CTGGTGTTGTT-
AAGGGGAAGACTCTTCTCGACGCCATCGACGCCATCGAACCTCCTGTCCGTCCCGAGAATAAG
CCTCTGCGTCTCCCGTTGCAGGATGTGATTTCTGTGGGCTCTTACTCAGCCCCAGTACTCGAGTT
CTTGGAAGAATGGGGCTTGGAGAATTTGGAGGACAATGCACA---GTCGCCAA------
ACCTAACCAAGGTGTTTGTTAACGGTGTTTGGCTTGGCGTGCATCGTGACCCGTTTAGTCTGGTA
AACACGCTCCGTAATCTTCGACGAAAGG---
GCGATCTTGTCAGCGAAGTATCTATTGTACGGGATATCCGCGAAAAAGAGGTCCGCGTATACAC
GGACGCGGGTCGCGTGCTTCGACCTCTATTCATCGTCGAGAAGCAGCATTTGAAGTTGAAACGT
CAGCATCTTCGATGGCTTCAAGAGAAATGGAGAATT-------
GAGGAGAGACCTATTGAAAAGCCACCCGTAGATGATGAGCAGAACGTGGAGGAATCGGAGGG
AGAGACAATAGCCGAG---
GAAGAGGATGGCAAGCCTATAATCCGAGACGGAATTAGGATGAAAAAGAAGCGGAAGCCCTT
```

CAATTGGGGCACTTTGGTTTCAGACGGTGTCATCGAATACCTTGACGCTGAAGAGGAAGAGACA
GTCATGATTTGTATGACTCCGGATGATTTGGTCGAGTCGCGGTTAATGAGTCGCGGACTGGATC
CCAG---AGCAGATGGTGACTTCGACCCGGCTGCTCGTCTCAAGGCAGTTCCAAAT------
GCTCACACATGGACACACTGCGAGATCCATCCTAGCATGATTTTAGGTATTTGCGCTAGTATTAT
CCCATTCCC
>Sanghuangporus_vaninii_Dai_8236

---------------------CA-TGTG-CACGGC-------------------------------CTT-
CGCG-----CTCAAATCCAA--CTCTC-------------AAAC-CCCC--TGTGCA-CCTTATTA----
------------------------------TATCGCGAGTCGAAG------------------------------
-------------------------TTAGTAGT----------------CTGAG-GTCTGTCT------------
----------------------------------TGTAAGTAATGAGTAGAAGGGCGAAAGC---------
----

--------------GAGCGAGTGT---
-----------------------------------TGCTCGTTAGGTA----------------------------
----
--------------------------------------ACCCTTCGAA-------AATGAAAGCGAG------
--C-----------------------------GGGAGT-GCGTCGGGTGAAGAC-TTGGG-----
CTTGTTG-TTAC-------------AAACCCCCCCTTATATTGTCTTTGTGAAT-G-----T-AAT-GC-
T-CC-TC----GTGGGCGAAAA------
TCAATACAACTTTCAACAACGGATCTCTTGGCTCTCGCATCGATGAAGAACGCAGCGAAATGCG
ATAAGTAATGTGAATTGCAGAATTCAGTGAATCATCGAATCTTTGAACGCACCTTGCGCCCCTTG
G-TATTCCGAGGGGCATGCCTGTTTGAGTGTCATGTTTATCTCAAACCGC--------TCG-
TCTTTCTTTAAT---------TGAA--GGGCTT--------GAGGTTTGGAC-TTGGAGG-T------
TTACTGCTGGCCGCCTTTCG------------------
AAGGGGGTCGGCTCCTCTTAAATACATTAGC-TGGGCTTTGG----------------C-TC---
GCG-CTT-----------ACGGTGTAATAGTTGAT-------------TCCG-TTCACC---AACGAG-
CGCTTG-CCTAA-CGAGCTTGC-TTC-----TAACGGTC---------------------------------
----
GAAGAGCTCAAATTTCAAATCT-G-GCG-CCCT-
TGTGGACGTCCGAGTTGTAGTCTGGAGAAGTGTTAT-CCGCGTCG-
GACCGTGTACAAGTCTCCTGGAACGGAGCGTCATAGAGGGTGAGAATCCCG-
TCCATGACACGG-AC-GCCCGATGC-
TATGTGAGGCACTCTCAAAGAGTCGAGTTGTTTGGGAATGCAGCTCAAAATGGGTGGTAAATTC
CATCTAAAGCTAAATATTGGCGAGAGACCGATAGCGAACAAGTACCGTGAGGGAAAGATGAAA
AGCACTTTGGAAAGAGAGTTAAACAGTACGTGAAATTGTTGAAAGGGAAACGCTTGAAGTCAG
TCGCGTCCCGTGGAACTCAGCCTGGT----TTCGACCTGGTGTACTTTCCAT-GC-
GGGACGGGTCAACGTCAA-TTTC-GGC-
CGGTGGACAAGGGCGAGGGGAATGTAGCGTTGCTTCGGCGACGTG-TTATAGC-CCCCCGTC-
GTAT-ACACCGGGCGGGATTGAGGA-CGGCAGCACGCCC------TT-G-TGGCC-------
GGGGGGG----------------TT--CG---CC-CCCCACG-T-AACGTGCTTAGGAT-
GTTGGCATAATGGCTTTAAGCGACCCGTCTTGAAACACGGACCAAGGAGTCTAACATGCTTGCG
AGTGTTCGGGTGGAAAACCCTTGCGCGTAATGAAAGTGAAA-GTTGGGAA-----CCTC-------

-----------CGCGA--GGGGGTGCAGCGACGCCCGGCCCTGACG-----
TTCTCTGACGGTGCCGCGGTAGAGCAGGTATGTTGGG----
ACCCGAAAGATGGTGAACTATGCCTGAATAGGGCGAAGCCAGAGGAAACTCTG-
GTGGAGGCTCGTAGCGATTCTGACGTGCAAATCGATCGTCAAATTTGGG------------------
----

CAAACTCGCGAGCACGCCCTTTTGGCCTTCACTCTCGGTGTACGTCAGCTTATCGTCGCGGTTAA
CAAAATGGATACCACCAAGGTTAGATTTGATCAATAAT-CAATCATGAGTCAAAAGCTGATATA-
GCCTTCA----------
CTTGTAGTGGTCCGAAGAGCGATTCAACGAAATCGTCAAGGAAACTTCTAACTTCATCAAGAAG
GTCGGCTACAACCCCAAGGCTGTACCTTTCGTCCCAATTTCCGGCTGGCATGGCGACAACATGC
TTGAGGAATCAAGCAAGTACGTATGACCA-TTAACTA---------CAGATGA-----
CTGTACTGACTTCTTATT-TGCAGTATGCCGTGGTACAAGGGTTGGACGAAGGAGACGAAGG--
----------CTGGTGTTGTT-AAAGGAAAGACTCTCCTGGACGCCATC---------------------
----
ATTTCTGTGGGCTCTTACTCAGCCCCAGTACTCGAGTTCTTGGAAGAATGGGGCTTGGAGAATTT
GGAGGACAATGCACA---GTCGCCAA------
ACCTAACCAAGGTGTTTGTTAACGGTGTTTGGCTTGGAGTGCATCGTGACCCGTTTAGTCTGGTA
AACACGCTCCGTAATCTTCGACGAAAGG---
GCGATCTTGTCAGTGAAGTATCTATTGTACGGGATATCCGCGAAAAAGAGGTCCGCGTATACAC
GGACGCGGGTCGCGTGCTTCGACCTCTATTCATCGTCGAGAAGCAGCATCTGAAGTTGAAACGT
CAGCATCTTCGATGGCTTCAAGAGAAATGGAGAATT-------
GAGGAGAGACCTATTGAAAAGCCACCCGTAGATGATGAGCAGAACGTGGAGGAATCGGAGGG
AGAGACAATAGCCGAG---
GAAGAGGATGGCAAGCCTATAATCCGAGACGGAATTAGGATGAAGAAGAAGCGGAAGCCCTT
CAATTGGGGCACTTTGGTTTCAGACGGTGTCATCGAATACCTTGACGCTGAAGAGGAAGAGACA
GTCATGATTTGTATGACTCCGGATGATTTGGTCGAGTCGCGGCTAATGAGTCGCGGACTGGATC
CCAG---AGCAGAT--

>Sanghuangporus_weigelae_LWZ_20210623-2a
TCGAGTTTTGA-----A-AG-CGAGAGA-------------------------------CTTGCTGCTG--
GCGTG---------------AAATCG--CGCA-TGTG-CACTGC------------------------------
--CTT-CGCG-----CTCAAATCCAA--CTC----------------AAA-CCCC--TGTGCA-
CCTATATA-------------------------------------TCGCGAGTCGAAG-----------------
---------------------------------------TTAGTAGT----------------CTGAG-----
GTCT---

CGTAAGTAATTGGTAGAAAGGTGAA---

--AGCGGTCT---------

TACTCGTTAGGTA---

---GCCTTTCGAA-------TATGAAAGC--
GAGT-GCGTCGGGTGAAGCCTTTCGG-----CTTGTTG-TTAC-------------AA---
ACACCTTATATTGTCTCTGTGAAT-G-----T-AAT-GC-T-CC-TC----GTGGGCGAAAA------
TAAATACAACTTTCAACAACGGATCTCTTGGTTCTCGCATCGATGAAGAACGCAGCGAAATGCG
ATAAGTAATGTGAATTGCAGAATTCAGTGAATCATCGAATCTTTGAACGCACCTTGCGCCCCTTG
G-TATTCCGAGGGGCATGCCTGTTTGAGTGTCATGTTAATCTCAAACCGC--------TCG-
TCTTTCTTAATTG--------TGAA--GGGCTT--------ACGGTTTGGAC-TTGGAGG-T-T------
ACTGCTGGC-GCCTTT--------------------CGAGGGGTCGGCTCCTCTTAAATACATTAGC-
CGGGCTTTGG----------------C-TC---GCG-TTT-----------ACGGTGTAATAGTTGAT---
----------TCCA-TTCACC---AACGAG-CGCTTG-CCTGA-CGAGCTTGC-TTC-----
CAGTCGTCCGCGTCGT------------CGGAC-AA--------GG------AGTTAT------
CTCCTTCTTTGACACCTTTGACCTCATCCCCTAGTAACTGCGAGTGAAGCGGGAAGAGCTCAAA
TTTAAAATCT-G-GCG-GCCT-TCTGGACGTCCGAGTTGTAGTCTGGAGAAGTGTTAT-
CCGCGTCG-GACCGTGTACAAGTCTCCTGGAACGGAGCGTCATAGAGGGTGAGAATCCCG-
TCCATGACACGG-AC-GCCCGATGC-
TATGTGAGGCACTCTCAAAGAGTCGAGTTGTTTGGGAATGCAGCTCAAAATGGGTGGTAAATTC
CATCTAAAGCTAAATATTGGCGAGAGACCGATAGCGAACAAGTACCGTGAGGGAAAGATGAAA
AGCACTTTGGAAAGAGAGTTAAACAGTACGTGAAATTGTTGAAAGGGAAACGCTTGAAGTCAG
TCGCGTCCTGCGGAACTCAGCCTGGT----TTCGACCTGGTGTACTTTCCAT-GT--
GGACGGGTCAACATCAA-TTTC-GGT-
CGGTGGACAAGGGCGAGGGGAATGTAGCGTTGCTTCGGCGACGTG-TTATAGC-CCCTTGTC-
GTAT-ACGCTGGTTGGGATTGAGGA-CCGCAGCACGCCC------TT-G-TGGCC-------
GGGGG----------------------TT---CG-CCCCACG-T-AACGTGCTTAGGAT-
GTTGGCATAATGGCTTTAAGCGACCCGTCTTGAAACACGGACCAAGGAGTCTAACATGCTTGCG
AGTGTTCGGGTGGAAAACCCTTGCGCGTAATGAAAGTGAAA-GTTGGGAA-----CCTC-------
-----------CGCGA--GGGGGTGCACCGACGCCCGGCCCTGACG-----
TTCTCTGACGGTGCCGCGGTAGAGCACGTATGTTGGG----
ACCCGAAAGATGGTGAACTATGCCTGAATAGGGCGAAGCCAGAGGAAACTCTG-
GTGGAGGCTCGTAGCGATTCTGACGTGCAAATCGATCGTCAAATTTGGGTCGATGTGTC--AT--
-CGGGGATAAGGTAAGGGAAAAT-
AATGATATTACCTTATTAATAGTGTCGTCCAACAACTCGTGCCAGAAGACTCGGTAAGGCGAGA
GACACAAACGTTAATCGTCTTGATCTGGCGTAAAGGGTTTGTAGGCGGCTTTAAGTATCCTTTTT
TTTAGTTTATTTTTAT--------------------------------AAAATTATAT-------------
ACTATATTT---

AGTAATAGTTTTGTATTATATATTAAAAGATCAATTAAAGCTAGAATCTAAAAGAGGTTA----
AAACAATGCTTGAT-TACAGGTGTACTAACCGTAAACAACTAGTAGAGTGT-
TAAGGGCGAAGGC-

TTTTTTCCAATAAAGATTGACGCTGAGAAACGAAGGTGAGGAAAGAAAAAAGGATTAGAGACC
CTTGTATCCCTCACTGTCAACGATGAATGGTAGTTACTAGAGAGAA-----------------------
----
ATAATCTAGAGACAATGTTAACACGAAAACCATTCCGCCTTGTGAGTACGACTGCAAAGTTGAA
AACAAAAAAATTAGTCGGTTTCGGAG--CAAACGAAGTGAAGC-ATGTTA-----------------
----
ACTCGCGAGCACGCCCTTTTGGCCTTCACTCTCGGTGTACGTCAGCTTATCGTCGCGGTCAACA
AAATGGATACCACCAAGGTTAGATTTGATCAATGAT-CAATCACGAGCCAAAAGCTGATATA-
TCTTTCA----------
CTTGTAGTGGTCTGAAGAGCGATTCAACGAAATCGTCAAGGAAACTTCCAACTTCATAAAGAAG
GTCGGCTACAACCCCAAGGCTGTACCTTTCGTCCCAATTTCCGGCTGGCACGGCGACAACATGC
TTGAGGAATCAAGCAAGTACGTATGACCA-TTAACTA---------CAGATGG-----
CTGTACTGACTTCTTATT-TGCAGTATGCCGTGGTACAAGGGCTGGACTAAGGAGACGAAGG---
---------CTGGTGTTGTTAAAGGGAAAGACTCTTCTCGACT----------------------------
----
ATTTCCGTGGGCTCTTACTCAGCTCCCGTGCTCGAGTTCTTGGAAGAATGGGGCTTGGAAAATTT
GGAGGACAACGCACA---GTCGCCAA------
ACCTAACCAAGGTGTTCGTTAACGGTGTTTGGCTTGGGGTGCATCGTGATCCGTTTAGTCTGGTA
AACACGCTCCGTAATCTTCGGCGAAAGG---
GCGATCTTGGCAGCGAAGTATCTATTGTACGGGACATCCGCGAAAAGGAAGTTCGCGTATACA
CGGACGCGGGTCGCGTGCTTCGACCTCTATTCATCGTCGAGAAGCAGCATCTGAAGTTGAAAC
GTCAGCATCTCCGATGGCTTCAAGAGAAATGGAGAGTC-------
GAGGAGAGACCTATCGAAAAGCCACCCGTAGATGACGAGCAGAACGTGGAGGAATCGGAAGG
AGAGACAATAGCCGAG---
GAAGAGGATGGCAAACCTATAATCCGAGACGGAACTAGGATGAAGAAGAAGCGGAAGCCCTT
CAACTGGGGCACTTTGGTTTCAGACGGTGTCATCGAATACCTTGACGCTGAAGAGGAAGAGAC
AGTCATGATTTGTATGACTCCAGATGATTTGGTCGAGTCGCGGTTAATGAGTCGCGGACTCGAT
CCCAG---AGCCGATGGTGACTTCGACCCGGCTGCTCGCCTCAAGGCAGTTCCAAAT------
GCTCACACATGGACGCACTGCGAGATTCATCCTAGCATGATTTTAGGTATTTGCGCTAGTATCAT
CCCATTCCC
