# Supplementary material for: Spatiotemporal Pattern of a Macrofungal Genus Phylloporia (Basidiomycota) Revealing Its Adaptive Evolution in China
Source: J Fungi (Basel). 2024 Nov 10;10(11):780. doi: 10.3390/jof10110780 (PMC11595563; doi:10.3390/jof10110780)
Supplement: Supplementary file 1 [file jof-10-00780-s001.zip › Table S1.pdf]

Table S1 Known records of *Phylloporia* used for predicting the current potential distribution of *Phylloporia* in China.

Species	Sampling location	Longitude	Latitude	Voucher No.	Host plant
P. alyxiae	Taiwan, Taitung, Orchid Island	121.5667	22.01667	GC 1604-28	*Alyxia insularis*
P. alyxiae	Taiwan, Taitung, Orchid Island	121.5667	22.01667	Chen 1182	*Alyxia insularis*
P. alyxiae	Taiwan, Taitung, Orchid Island	121.5667	22.01667	Chen 924	*Alyxia insularis*
P. alyxiae	Taiwan, Taitung, Orchid Island	121.5667	22.01667	Chen 943	*Alyxia insularis*
P. alyxiae	Taiwan, Taitung, Orchid Island	121.5667	22.01667	Chen 995	*Alyxia insularis*
P. alyxiae	Taiwan, Taitung, Orchid Island	121.5667	22.01667	13669	*Alyxia insularis*
P. alyxiae	Taiwan, Taitung	121.15	22.75	Chen 576	*Alyxia insularis*
P. alyxiae	Taiwan, Taitung	121.15	22.75	Chen 577	*Alyxia insularis*
P. alyxiae	Taiwan, Taitung	121.15	22.75	Wu 9705-7	*Alyxia insularis*
P. alyxiae	Taiwan, Taitung	121.15	22.75	Wu 9705-44	*Alyxia insularis*
P. alyxiae	Taiwan, Taitung	121.15	22.75	Wu 9705-46	*Alyxia insularis*
P. alyxiae	Taiwan, Taitung	121.15	22.75	Wu 9805-11	*Alyxia insularis*
P. alyxiae	Taiwan, Taitung	121.15	22.75	Wu 9805-13	*Alyxia insularis*
P. alyxiae	Taiwan, Taitung	121.15	22.75	Wu 9805-31	*Alyxia insularis*
P. clausenae	Hainan, Changjiang, Bawangling Nature Reserve	109.08	19.12	Dai 10831	*Clausena*
P. clausenae	Yunnan, Xishuangbanna, Menglun, Lvshilin park	101.2792	21.90663	Yuan 3528	*Clausena*
P. crataegi	Liaoning, Shenyang, Beiling Park	123.43	41.85	Dai 11014	*Crataegus*
P. crataegi	Liaoning, Shenyang, Beiling Park	123.43	41.85	Dai 11016	*Crataegus*
P. crataegi	Beijing, Xishan National Forest Park	116.1976	39.97171	IFP018096	*Crataegus*
P. crataegi	Liaoning, Anshan, Qianshan	122.96048	41.07507	IFP015196	*Crataegus*
P. crataegi	Liaoning, Tieling	123.844279	42.290585	IFP018095	*Crataegus*
P. cylindrispora	Guangxi, Longzhou, Nonggang Nature Reserve	106.96	22.47	Yuan 6144	Angiosperm
P. cylindrispora	Guangxi, Longzhou, Nonggang Nature Reserve	106.96	22.47	Yuan 6148	Angiosperm
P. cystidiolophora	Chongqing, Nanchuan, Jinfoshan Nature Reserve	107.14	29.04	Dai13945	*Crataegus*

P. cystidiolophora	Chongqing, Nanchuan, Jinfoshan Nature Reserve	107.14	29.04	Dai13953	*Crataegus*
P. dependens	Yunnan, Ruili	97.81153	24.037273	Dai 13167	Angiosperm
P. flacourtiae	Guangxi, Longzhou, Nonggang Nature Reserve	106.96	22.47	Zhou 140	*Flacourtia*
P. flacourtiae	Guangxi, Longzhou, Nonggang Nature Reserve	106.96	22.47	Zhou 165	*Flacourtia*
P. flacourtiae	Guangxi, Longzhou, Nonggang Nature Reserve	106.96	22.47	Yuan 6198	*Flacourtia*
P. flacourtiae	Guangxi, Longzhou, Nonggang Nature Reserve	106.96	22.47	Yuan 6204	*Flacourtia*
P. flacourtiae	Guangxi, Fangchenggang, Golden Camellia Nature Reserve	108.12	21.74	Yuan 6360	*Flacourtia*
P. flacourtiae	Guangxi, Fangchenggang, Golden Camellia Nature Reserve	108.12	21.74	Yuan 6362	*Flacourtia*
P. fontanesiae	Henan, Xinyang, Jigongshan Nature Reserve	114.11	31.82	Li 194	*Fontanesia*
P. fontanesiae	Henan, Xinyang, Jigongshan Nature Reserve	114.11	31.82	Li 199	*Fontanesia*
P. fontanesiae	Henan, Xin County, Liankang Mountain National Nature Reserve	114.8333	31.59167	BJFC017269	*Fontanesia*
P. fontanesiae	Henan, Xin County, Liankang Mountain National Nature Reserve	114.8333	31.59167	BJFC017270	*Fontanesia*
P. fontanesiae	Shandong, Jinan, Qianfo Mountain Scenic Area	117.07	36.63	LWZ 20191013-1	*Fontanesia*
P. fontanesiae	Shandong, Jinan, Qianfo Mountain Scenic Area	117.07	36.63	LWZ 20201024-3	*Fontanesia*
P. gutta	Sichuan, Aba, Jiuzhaigou Nature Reserve	103.92	33.26	Dai 4197	*Abelia*
P. gutta	Sichuan, Aba, Jiuzhaigou Nature Reserve	103.92	33.26	Dai 4103	*Abelia*
P. hainaniana	Hainan, Qiongzhong, Limushan Nature Reserve	109.86	19.04	Dai 9460	Angiosperm
P. hainaniana	Hainan, Ledong, Jianfengling Nature Reserve	108.8751	18.71495	Dai 16142	Angiosperm
P. homocarnica	Guangxi, Guilin, Xing'an, Maoershan Nature Reserve	110.48737	25.857773	Yuan 5750	Angiosperm
P. homocarnica	Guangxi, Guilin, Xing'an, Maoershan Nature Reserve	110.48737	25.857773	Yuan 5766	Angiosperm
P. lespedezae	Shanxi, Huguan, Baquanxia National Park	113.59416	35.94544	Dai 17067	*Lespedeza*
P. lespedezae	Shanxi, Huguan, Baquanxia National Park	113.59416	35.94544	Dai 17065	*Lespedeza*
P. lonicerae	Hubei, Huanggang, Luotian	115.27	30.83	LWZ 20200324-4	*Lonicera*
P. lonicerae	Shandong, Linyi, Pingyi, Liushangu, Xincheng	117.64	35.51	LWZ 20190809-1	*Lonicera*

P. lonicerae	Shandong, Linyi, Fei County	117.98	35.27	LWZ 20190809-2	*Lonicera*
P. lonicerae	Shandong, Linyi, Fei County	117.98	35.27	LWZ 20190809-3	*Lonicera*
P. lonicerae	Shandong, Linyi, Fei County	117.98	35.27	LWZ 20190819-1	*Lonicera*
P. manglietiae	Hainan, Baoting, Qixianling Forest Park	109.7	18.7	Cui 13709	*Manglietia*
P. minutipora	Hainan, Ledong, Jianfengling Nature Reserve	108.8751	18.71495	Dai 9257	Angiosperm
P. minutipora	Hainan, Shuiman, Wuzhishan Nature Reserve	109.516662	18.776921	LWZ 20150531-13	Angiosperm
P. minutipora	Hainan, Shuiman, Wuzhishan Nature Reserve	109.516662	18.776921	LWZ 20150531-14	Angiosperm
P. minutipora	Hainan, Shuiman, Wuzhishan Nature Reserve	109.516662	18.776921	LWZ 20150531-15	Angiosperm
P. minutissima	Yunnan, Mengla, Xishuangbanna Tropical Botanical Garden	101.461959	21.73667	Dai 21223	Angiosperm
P. mori	Taiwan, Taitung, Yanping	121.15	22.9	Wu 1105-2	*Morus*
P. mori	Taiwan, Taitung, Yanping	121.15	22.9	Wu 1105-3	*Morus*
P. mori	Taiwan, Taitung, Changpin	121.43	23.35	Wu 1206-1	*Morus*
P. mori	Taiwan, Taitung, Changpin	121.43	23.35	Wu 1206-2	*Morus*
P. mori	Taiwan, Taitung, Changpin	121.43	23.35	Wu 1206-3	*Morus*
P. mori	Taiwan, Taitung, Changpin	121.43	23.35	Wu 1206-4	*Morus*
P. mori	Taiwan, Taitung, Changpin	121.43	23.35	Wu 1206-5	*Morus*
P. moricola	Sichuan, Xichang, Puge, Luojishan	114.299322	30.586877	TNM F0034325	*Morus*
P. murrayae	Taiwan, Taitung, Changpin	121.43	23.35	Wu 1404-4	*Murraya*
P. murrayae	Taiwan, Taitung, Changpin	121.43	23.35	Wu 1404-5	*Murraya*
P. murrayae	Taiwan, Taitung, Changpin	121.43	23.35	Wu 1404-6	*Murraya*
P. murrayae	Taiwan, Taitung, Green Island (Lyudao Township), Across Mountain Ancient Trail	121.5	22.65	Chen 1708	*Murraya*
P. murrayae	Taiwan, Taitung, Green Island (Lyudao Township), Yutzuhu	121.5	22.66667	Chen 770	*Murraya*

P. nandinae	Jiangxi, Jinggangshan County, Jinggang Mountain	114.15	26.51	Dai 10588	*Nandina domestica*
P. nandinae	Jiangxi, Jinggangshan County, Jinggang Mountain	114.15	26.51	Dai 10625	*Nandina domestica*
P. oblongospora	Guangxi, Longzhou, Nonggang Nature Reserve	106.8585	22.431578	Zhou 179	Angiosperm
P. oblongospora	Guangdong, Zhaoqing, Dinghushan	112.540918	23.165922	BJFC007884	Angiosperm
P. oblongospora	Guangdong, Zhaoqing, Dinghushan	112.540918	23.165922	BJFC007895	Angiosperm
P. oreophila	Tibet, Bomi	95.77	29.86	Cui 9503	Angiosperm
P. oreophila	Gansu, Lanzhou, Xinglongshan Nature Reserve	104.04	35.79	Cui 2219	Angiosperm
P. oreophila	Sichuan, Liangshan, Luojishan Scenic Spot	114.299322	30.586877	LWZ 20190811-27a	Angiosperm
P. osmanthi	Guangxi, Guilin, Xing'an, Maoershan Nature Reserve	110.48737	25.857773	Yuan 5655	*Osmanthus*
P. pendula	Hainan, Shuiman, Wuzhishan Nature Reserve	109.516662	18.776921	Cui 13691	Angiosperm
P. pendula	Hainan, Changjiang, Bawangling Nature Reserve	109.08	19.12	Cui 13876	Angiosperm
P. perangusta	Guangdong, Renhua,Danxiashan Nature Reserve	114.15	24.7	Dai 18139	Angiosperm
P. pseudopectinata	Hainan, Ledong, Jianfengling Nature Reserve	108.8751	18.71495	Cui 13749	Angiosperm
P. pseudopectinata	Hainan, Ledong, Jianfengling Nature Reserve	108.8751	18.71495	Cui 13746	Angiosperm
P. pseudopectinata	Hainan, Ledong, Jianfengling Nature Reserve	108.8751	18.71495	LWZ 20170613-20	Angiosperm
P. pulla	Hainan, Shuiman, Wuzhishan Nature Reserve	109.516662	18.776921	Dai 9627	Angiosperm
P. pulla	Hainan, Ledong, Jianfengling Nature Reserve	108.8751	18.71495	Cui 5251	Angiosperm
P. pulla	Shandong, Zibo	117.84	36.48	LWZ 20190428-2	Angiosperm
P. pulla	Shandong, Zibo	117.84	36.48	LWZ 20190428-3	Angiosperm
P. pulla	Shandong, Zibo	117.84	36.48	LWZ 20190428-4	Angiosperm
P. radiata	Guizhou, Fanjingshan National Nature Reserve	108.77	27.84	LWZ 20141122-5	*Liana*
P. radiata	Guizhou, Fanjingshan National Nature Reserve	108.77	27.84	LWZ 20141122-6	*Liana*

P. radiata	Guizhou, Fanjingshan National Nature Reserve	108.77	27.84	LWZ 20141122-19	*Liana*
P. rattanicola	Fujian, Longyan, Liancheng, Guanzhaishan	116.821447	25.604176	Dai 18235	*Rattan*
P. rattanicola	Fujian, Longyan, Liancheng, Guanzhaishan	116.821447	25.604176	Dai 18232	*Rattan*
P. rattanicola	Fujian, Longyan, Liancheng, Guanzhaishan	116.821447	25.604176	Dai 18233	*Rattan*
P. rubiacearum	Taiwan, Taitung, Green Island, 0.6 km of Across Mountain Ancient Trail	121.4937	22.64971	Chen3580	*Wendlandia*
P. rubiacearum	Taiwan, Taitung, Green Island, 0.6 km of Across Mountain Ancient Trail	121.4937	22.64971	Chen 1707	*Wendlandia*
P. rubiacearum	Taiwan, Taitung, Green Island, 0.6 km of Across Mountain Ancient Trail	121.4937	22.64971	Chen 1714	*Wendlandia*
P. rubiacearum	Taiwan, Taitung, Green Island, 0.6 km of Across Mountain Ancient Trail	121.4937	22.64971	Chen 3579	*Wendlandia*
P. rubiacearum	Taiwan, Taitung, Green Island, 0.6 km of Across Mountain Ancient Trail	121.4937	22.64971	Chen 3583	*Wendlandia*
P. rubiacearum	Taiwan, Taitung, Green Island, 0.6 km of Across Mountain Ancient Trail	121.4937	22.64971	Chen 3584	*Wendlandia*
P. rubiacearum	Taiwan, Taitung, Green Island, 0.6 km of Across Mountain Ancient Trail	121.4937	22.64971	Chen 3585	*Wendlandia*
P. rubiacearum	Taiwan, Taitung, Orchid Island, Hsiaotienchih	121.55	22.04	GC 1805-19	*Wendlandia*
P. rubiacearum	Taiwan, Taitung, Orchid Island, Hsiaotienchih	121.55	22.04	GC 1805-20	*Wendlandia*
P. rubiacearum	Taiwan, Taitung, Orchid Island, Hsiaotienchih	121.55	22.04	Wu 9705-8	*Wendlandia*
P. splendida	Yunnan, Xishuangbanna, Menglun, Lvshilin park	101.2792	21.90663	Cui 8429	Angiosperm
P. splendida	Zhejiang, Hangzhou, Lin'an, Tianmushan Nature Reserve	119.3503	30.20768	Dai 6282	Angiosperm
P. subpulla	Hainan, Ledong, Jianfengling Nature Reserve	108.875116	18.714954	Cui 5251	Angiosperm
P. subpulla	Hainan, Shuiman, Wuzhishan Nature Reserve	109.516662	18.776921	Dai 9627	Angiosperm

P. subpulla	Hainan, Ledong, Diaoluoshan Nature Reserve	109.915968	18.659778	Dai 9313	Angiosperm
P. subpulla	Hainan, Qiongzhong, Limushan Nature Reserve	109.86	19.04	Dai 16272	Angiosperm
P. terrestris	Guangxi, Guilin, Xing'an, Maoershan Nature Reserve	110.601021	25.607031	Yuan 5738	Ground (under angiosperm)
P. terrestris	Guangxi, Guilin, Xing'an, Maoershan Nature Reserve	110.601021	25.607031	He 2359	Ground (under angiosperm)
P. tiliae	Hunan, Zhangjiajie, Tianmenshan National Forest Park	119.350295	30.207684	Yuan 5491	*Tilia*
P. sp.	Hainan, Ledong, Jianfengling Nature Reserve	108.875116	18.714954	Dai 9242	Angiosperm
P. sp.	Yunnan, Ruili	97.81153	24.037273	Cui 13763	Angiosperm
