# Supplementary material for: Spatiotemporal Pattern of a Macrofungal Genus Phylloporia (Basidiomycota) Revealing Its Adaptive Evolution in China
Source: J Fungi (Basel). 2024 Nov 10;10(11):780. doi: 10.3390/jof10110780 (PMC11595563; doi:10.3390/jof10110780)
Supplement: Supplementary file 1 [file jof-10-00780-s001.zip › Table S2.pdf]

Table S2 Information of collections used for generating the time-calibrated phylogenetic tree.

Species	Voucher	ITS	nLSU	mt-SSU	*tef1-a*	*rpb2*
Coltricia abieticola	Cui 10321	KX364785	KX364804	KY693823	KY693911	KX364876
Coltricia austrosinensis	Dai 13093	KU360670	KU360640	KY693826	KY693913	KX364872
Coltricia baoshanensis	Dai 13075	KX364800	KX364820	KY693870	KY693953	KX364912
Coltricia globosa	Cui 7545	KJ540930	KJ000226	KY693873	KY693954	KX364913
Fomitiporella chinensis	Cui 11230	KX181309	KY693759	KY693880	KY693958	KY693910
Fomitiporella sinica	LWZ 20140624-5	KX181302	KX181321			
Fomitiporella subinermis	Dai 15131	KX181307	KX181345			
Fomitiporella tenuissima	Dai 12365	KC456244	KC999901			
Fomitiporia alpina	Dai 15735	KX639627	KX639645		KX639664	KX639680
Fomitiporia gaoligongensis	Cui 16433	MG867712	MG867714		MG867720	MG867717
Fomitiporia punicata	Dai 10640	KX663825	KX639653		KX639667	KX663827
Fomitiporia subrobusta	Dai 13577	KX639618	KX639636		KX639656	KX639673
Fulvifomes dracaenicola	Dai 22097	MW559800	NG_075429			
Fulvifomes fastuosus	Dai 18292	MH390411	MH390381			
Fulvifomes hainanensis	Dai 11573	KC879263	JX866779			
Fulvifomes lloydii	Dai 9642	MH390429	MH390379			
Fuscoporia chinensis	Dai 15713	MN816721	MN810008		MN848846	MN848771
Fuscoporia contigua	Dai 16025	MG008401	MG008454		MH636386	MH079406
Fuscoporia ramulicola	Dai 15723	MH050749	MH050762		MN848824	MH079398
Fuscoporia rhabarbarina	Dai 16550	MN816744	MN810036		MN848836	MN848785
Hirschioporus acontextus	Dai 23793	OQ449141	OQ449200	OQ449123	OQ831439	
Hirschioporus chinensis	Dai 20264	OQ449101	OQ449204	OQ449127	OQ831443	
Hirschioporus pubescens	Dai 23710	OQ512026	OQ449059	OQ449169	OQ857930	
Hirschioporus tianschanicus	Dai 19067	OQ448960	OQ449067	OQ449177	OQ857947	

Hydnoporia gigasetosa	He 1442	KT828670	KT828674			
Hydnoporia lamellata	Cui 7629	JQ279603	JQ279617			
Hydnoporia tabacina	LWZ 20210924-26a	ON063651	ON063851	ON063720	ON089676	ON100685
Hydnoporia tabacinoides	LWZ 20190814-29b	ON063652	ON063852	ON063721		
Hymenochaete asetosa	Dai 10756	JQ279559	JQ279642	KU978829		
Hymenochaete cyclolamellata	Cui 7393	JQ279513	JQ279629	KU978838		
Hymenochaete huangshanensis	LWZ 20201014-27	ON063654	ON063854	ON063723		
Hymenochaete xerantica	LWZ 20190814-13b	ON063657	ON063856	ON063726	ON089680	ON100699
Inonotus griseus	Dai 13436	KX674583	KX364823	KY693881	KY693959	KX364919
Inonotus henanensis	Dai 13157	KX674581	KX832918		MF977776	MF973468
Inonotus obliquus	Dai 10715	MZ484606	OL413405			
Inonotus rickii	Dai 12996	KC479128	MH101019			
Lyomyces crustosus	LWZ 20170815-23	MT319465	MT319201	MT326446	MT326400	MT326275
Pallidohirschioporus biformis	Dai 14531	OQ453313	OQ453539	OQ453514	OQ874737	
Pallidohirschioporus brastagii	Dai 12316	OQ453235	OQ453289	OQ453268	OQ874749	
Pallidohirschioporus polycystidiatus	Dai 14686	OQ453377	OQ453300	OQ453278	OQ874756	
Pallidohirschioporus versicolor	Dai 19332	OQ453387	OQ474952	OQ534102	OQ874763	
Phellinopsis asetosa	Dai 13553	KJ425524	KJ425523			
Phellinopsis conchata	L-7601	KU139188	KU139257		KU139377	KU139315
Phellinopsis lonicericola	Dai 22911	OL473605	OL473618			
Phellinopsis tibetica	LWZ 20190808-31a	ON063660	ON063859	ON063730		
Phellinus castanopsidis	Cui 10153	NR_120108	NG_060099			
Phellinus mori	LWZ 20150825-1	ON063661	ON063861			
Phellinus piceicola	LWZ 20190921-5	ON063662	ON063862	ON063731		ON100695
Phellinus pomaceus	LWZ 20160908-1	ON063663	ON063863	ON063732		
Phylloporia alyxiae	GC 1604-28		LC514408			

Phylloporia alyxiae	Chen 1182		LC514407			
Phylloporia alyxiae	13669	MH151185				
Phylloporia clausenae	Yuan 3528		KJ787795			
Phylloporia clausenae	Cui8463	MH151186	MH165868		MH167424	MH161216
Phylloporia crataegi	Dai 11016		JF712923			
Phylloporia crataegi	Dai 18133	MH151191	MH165865	MH124754	MH167431	MH161224
Phylloporia cylindrispora	Yuan 6144		KJ787797			
Phylloporia cylindrispora	Yuan 6148		KJ787798			
Phylloporia cystidiolophora	Dai13945		MG738798			
Phylloporia cystidiolophora	Dai13953		MG738799	MH124760	MH167438	MH161227
Phylloporia dependens	Dai 13167		KP698746			
Phylloporia flacourtiae	Yuan 6360		KJ787800			
Phylloporia flacourtiae	Yuan 6362		KJ787801			
Phylloporia fontanesiae	Cui12356	MH151188	MH165871			
Phylloporia fontanesiae	LWZ 20201024-3	**PP068872**	**PP068875**			
Phylloporia gutta	Dai 16070	MH151183	MH165863	MH124749	MH167423	MH161215
Phylloporia gutta	Cui 6945	MH151182		MH124748	MH167422	MH161214
Phylloporia hainaniana	Dai 9460		JF712928			
Phylloporia hainaniana	Dai 16142		MH165866	MH124759	MH167437	
Phylloporia homocarnica	Yuan 5750	MH151195	KJ787803			
Phylloporia homocarnica	Yuan 5766		KJ787804			
Phylloporia lespedezae	Dai 17067	MH151180	KY242603	MH124747	MH167420	MH161212
Phylloporia lespedezae	Dai 17065	MH151179	KY242602	MH124746	MH167419	MH161211
Phylloporia lonicerae	LWZ 20190809-2	**PP068870**	MN893899			
Phylloporia lonicerae	LWZ 20190819-1	**PP068871**	MN893901			
Phylloporia manglietiae	Cui 13709	MF410324	KX242358	MH124753	MH167430	MH161223

Phylloporia manglietiae	Cui 5497		MH165872		MH167435		
Phylloporia minutipora	LWZ 20150531-13	MH151193	KU904465	MH124755	MH167432		
Phylloporia minutipora	Dai 9257		KU904464				
Phylloporia minutissima	Dai 21223		MZ437408				
Phylloporia mori	Wu 1105-2		LC514412				
Phylloporia mori	Wu 1105-3		LC514413				
Phylloporia moricola	Wu1807-1		LC589617				
Phylloporia moricola	Wu1807-5		LC589618				
Phylloporia murrayae	Wu 1404-4		LC514409				
Phylloporia murrayae	Wu 1404-5		LC514410				
Phylloporia nandinae	Dai 10588		JF712930				
Phylloporia nandinae	Dai 10625		JF712931		MH167439	MH161228	
Phylloporia oblongospora	Zhou 179	MH151197	JF712932				
Phylloporia oreophila	Cui 2219	MH151196	JF712933	MH124757			
Phylloporia oreophila	LWZ 20190811-27a	ON063665	ON063865	ON063733	ON089684	ON100694	
Phylloporia osmanthi	Yuan 5655		KF729938	MH124761			
Phylloporia pendula	Cui 13691	MF410320	KX242357	MH124751	MH167426	MH161218	
Phylloporia pendula	Cui 13876	MF410321	KX901670	MH124752	MF977777	MF973471	
Phylloporia perangusta	Cui 11306	MF410325	KX242354				
Phylloporia perangusta	Dai 18139	MH151169	MG738803	MH124739	MH167413	MH161204	
Phylloporia pseudopectinata	Cui 13749	MF410323	KX242356		MH167429	MH161222	
Phylloporia pseudopectinata	Cui 13746	MF410322	KX242355		MH167428	MF973472	
Phylloporia pulla	LWZ 20190428-2		MN893898				
Phylloporia pulla	LWZ 20190428-3		MN893897				
Phylloporia radiata	LWZ 20141122-5	MH151198	KU904470	MH124758	MH167434		
Phylloporia radiata	LWZ 20141122-6	MH151194	KU904471	MH124756	MH167433		

Phylloporia rattanicola	Dai 18235	MH151172	MG738808	MH124742	MH167415	MH161207
Phylloporia rattanicola	Dai 18232	MH151170	MG738806	MH124740	MH167414	MH161205
Phylloporia rubiacearum	Chen3580		LC514415			
Phylloporia rubiacearum	Chen 3583		LC514416			
Phylloporia sp.	Cui 13763	MH151190	KX242353			
Phylloporia sp.	Dai 9242	LC528151	JF712936			
Phylloporia sp.	Dai 18366	MH151192				
Phylloporia splendida	Cui 8429		MG738804			
Phylloporia splendida	Dai 6282		MG738805			
Phylloporia subpulla	Cui 5251		KU904468			
Phylloporia subpulla	Dai 9627		KU904469			
Phylloporia terrestris	Yuan 5738		KC778784			
Phylloporia terrestris	He 2359	MH151189	MH165869			MH161220
Phylloporia tiliae	Yuan 5491		KJ787805			
Porodaedalea alpicola	Cui 12272	MG585275	MH152375		MG585294	MH101492
Porodaedalea chrysoloma	Dai 12674	KY000004	MH152354		KY000009	MH101475
Porodaedalea laricis	LWZ 20190724-9	ON063668	ON063868	ON063735		ON100693
Porodaedalea mongolica	Dai 19231	MZ484567	ON529261		MZ514632	ON604673
Sanghuangporus alpinus	Cui 12444	MF772782	MF772800		MF977780	MF973474
Sanghuangporus quercicola	Dai 21763	MZ484583	MZ437413		OL436567	OL436571
Sanghuangporus vaninii	Dai 8236	MF772791	MF772812		MF977792	MF973485
Sanghuangporus weigelae	LWZ 20210623-2a	ON063671	ON063870	ON063736	ON089687	ON100697
Xylodon subflaviporus	Dai 16413	MT319567	MT319310	MT326547	MT326415	MT326309

The newly generated sequences are shown in bold.
