# Supplementary figures and images for: Spatiotemporal Pattern of a Macrofungal Genus Phylloporia (Basidiomycota) Revealing Its Adaptive Evolution in China
Source: J Fungi (Basel). 2024 Nov 10;10(11):780. doi: 10.3390/jof10110780 (PMC11595563; doi:10.3390/jof10110780)

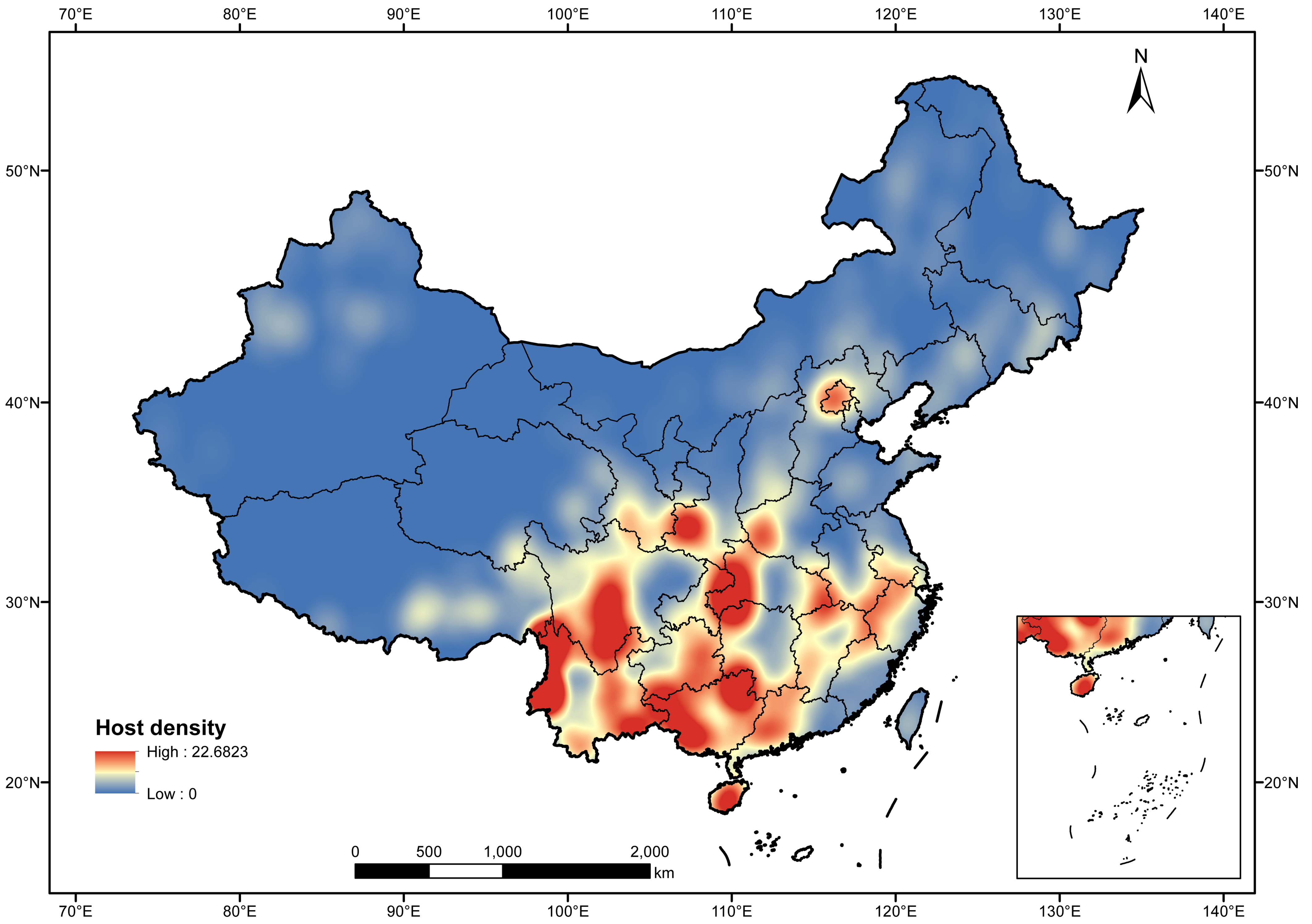

Supplement: Supplementary file 1 [file jof-10-00780-s001.zip › Figure S1.jpg]

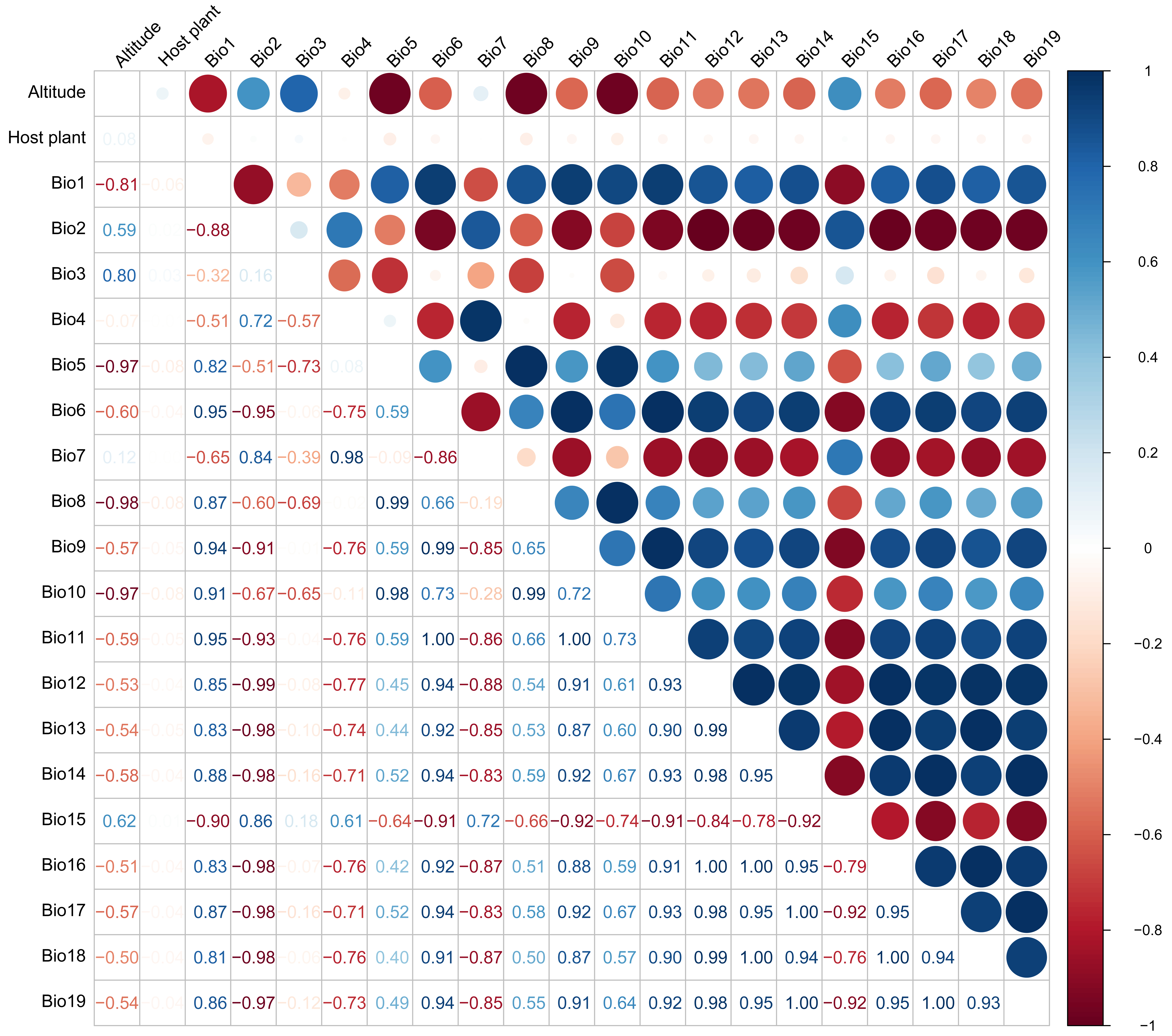

Supplement: Supplementary file 1 [file jof-10-00780-s001.zip › Figure S2.jpg]

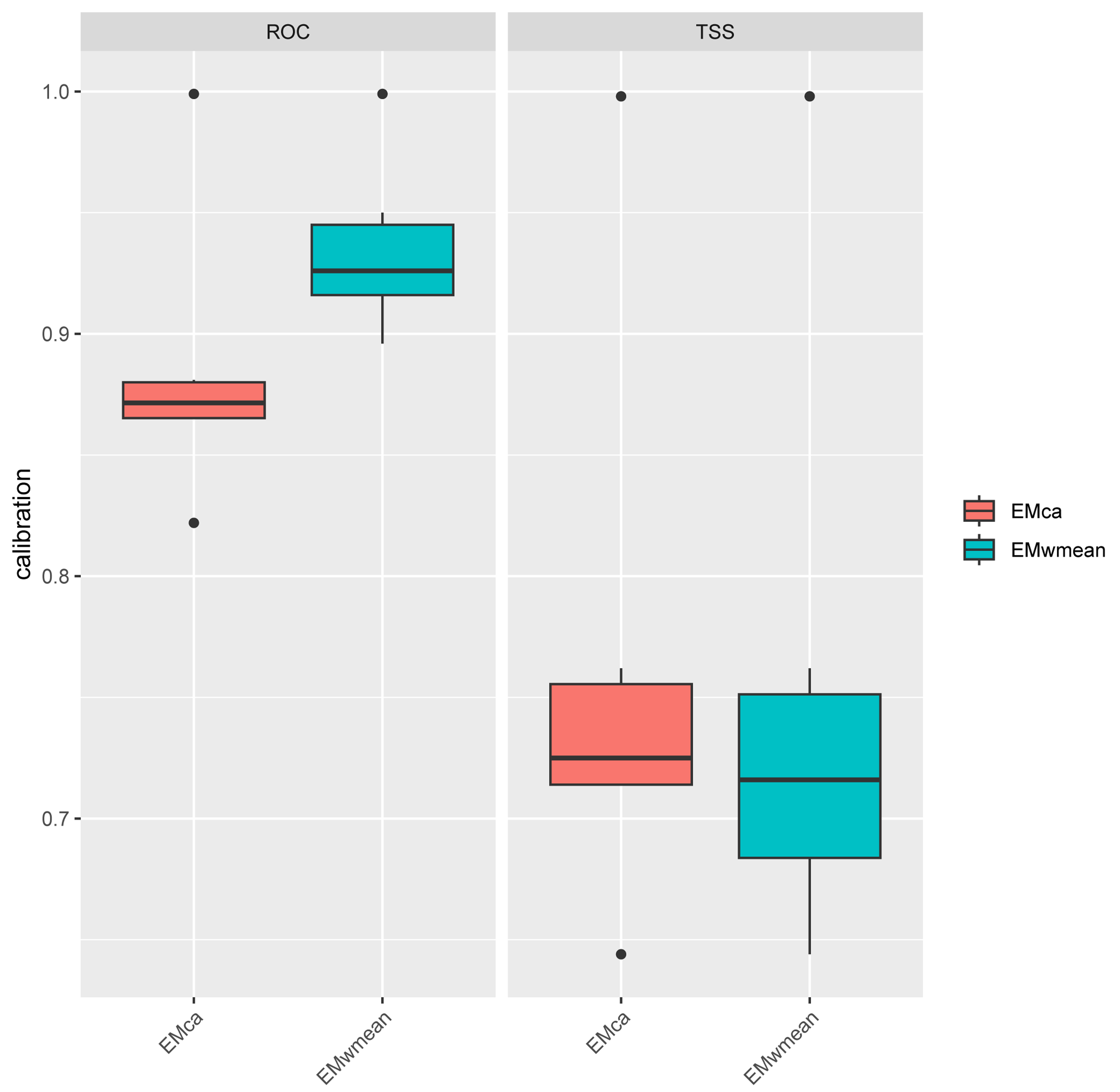

Supplement: Supplementary file 1 [file jof-10-00780-s001.zip › Figure S3.jpg]

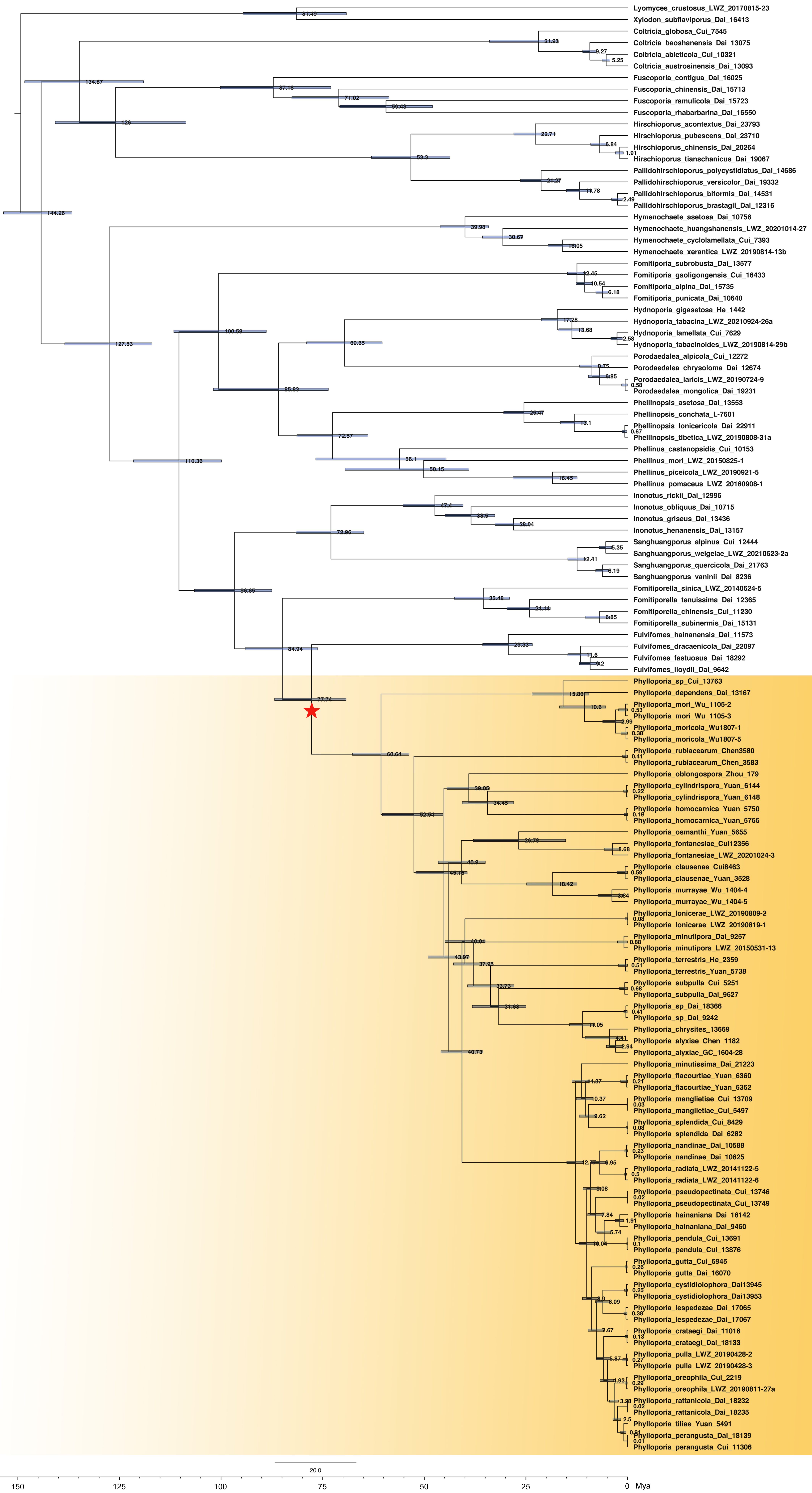

Supplement: Supplementary file 1 [file jof-10-00780-s001.zip › Figure S4.jpg]
